# Induction of cryptic pre-mRNA splice-switching by antisense oligonucleotides

**DOI:** 10.1038/s41598-021-94639-x

**Published:** 2021-07-23

**Authors:** Kristin A. Ham, Niall P. Keegan, Craig S. McIntosh, May T. Aung-Htut, Khine Zaw, Kane Greer, Sue Fletcher, Steve D. Wilton

**Affiliations:** 1 Centre for Molecular Medicine and Innovative Therapeutics, Health Futures Institute, Murdoch University, Perth, WA 6150 Australia; 2 Perron Institute for Neurological and Translational Science, Centre for Neuromuscular and Neurological Disorders, The University of Western Australia, Perth, WA 6009 Australia; 3 Department of Biochemistry, Faculty of Medicine Siriraj Hospital, Mahidol University, Bangkok, 10700 Thailand

**Keywords:** Molecular biology, RNA splicing

## Abstract

Antisense oligomers (AOs) are increasingly being used to modulate RNA splicing in live cells, both for research and for the development of therapeutics. While the most common intended effect of these AOs is to induce skipping of whole exons, rare examples are emerging of AOs that induce skipping of only part of an exon, through activation of an internal cryptic splice site. In this report, we examined seven AO-induced cryptic splice sites in six genes. Five of these cryptic splice sites were discovered through our own experiments, and two originated from other published reports. We modelled the predicted effects of AO binding on the secondary structure of each of the RNA targets, and how these alterations would in turn affect the accessibility of the RNA to splice factors. We observed that a common predicted effect of AO binding was disruption of the exon definition signal within the exon’s excluded segment.

## Introduction

The process of pre-mRNA splicing is a fundamental aspect of gene regulation and function in higher eukaryotes. Pre-mRNA consists of retained regions, termed exons, that are interspersed with regions destined for excision, termed introns^1^. During maturation into mRNA, the introns are removed and the exons are ligated together to form a continuous message, ready to be translated into a protein, or in some cases to serve other functions as a non-coding RNA. Pre-mRNA splicing involves a multitude of splicing factors that interact with numerous splicing motifs on the transcript^2^. A large multi-protein complex called the spliceosome is responsible for the coordination of this complex set of transesterification reactions^3^.

The major form of the spliceosome is composed of five small nuclear ribonucleoproteins (snRNPs; U1, U2, U5 and U4/U6), as well as numerous non-snRNP proteins^4,5^. The canonical 5′ splice site (5′ss) is defined by an AG|GURAGU sequence, while the 3′ splice site (3′ss) is denoted by a (Yn)-YAG| sequence (where; |= exon boundary; underlined sequence identifies invariant nucleotides; R = purine; Y = pyrimidine)^6^. The branchpoint sequence, typically located approximately 15 to 50 nucleotides (nt) upstream from the 3′ss, is required for U2 snRNA binding during spliceosome formation. This sequence is defined as YNC**U**R**A**Y (underlined sequence denotes branch formation region; bold nucleotides are highly conserved; N = any nucleotide)^6^. The major spliceosome (called spliceosome hereon), along with hundreds of associated splicing factors are responsible for over 95% of all splicing reactions, including the phenomenon known as alternative splicing^7–10^.

Alternative splicing is a process whereby multiple different transcripts and protein isoforms can arise from a single protein-coding gene and is an essential element in spatial and temporal regulation of gene expression in higher eukaryotes^7^. In order to achieve alternative splicing, the spliceosome must recognize and select a splice site amid a variety of alternative splice sites and branchpoints within the transcript. Typically, these splice sites are well defined and have evolutionarily conserved functions. However, on occasion, sequences usually ignored by the spliceosome can become activated as splice junctions. These are known as cryptic splice sites^11^ and are most often activated by mutations or errors during transcription^12^. According to DBASS, the mutations most commonly causative of cryptic splice site activation are those that weaken canonical exon splice sites, thus redirecting the spliceosome to utilize a viable cryptic site nearby^13^. However, this is a relatively rare outcome of such mutations, which are generally far more likely to induce whole exon skipping^14^. Cryptic splice sites may be found within both exonic and intronic regions and typically include or exclude a proportion of the exon or intron^12^. Interestingly, recent data has shown that cryptic splice sites can also be activated by synthetic molecules such as antisense oligonucleotides.

Antisense oligonucleotides (AOs) are small, single-stranded RNA or DNA-like synthetic molecules used to modify gene expression. These AOs can be used to downregulate gene expression through RNA silencing, redirection of pre-mRNA splicing patterns, intron retention, inhibiting translation, or RNase H-induced degradation of the target gene transcript^15^. The sequence of maturing gene transcripts can also be altered by using AOs to induce removal or inclusion of an exon, as demonstrated by current therapeutic strategies approved for the treatment of Duchenne muscular dystrophy and spinal muscular atrophy, respectively.

While most splice modulating AOs are designed with the intention to enhance exon selection or induce skipping of whole exons, the occasional activation of cryptic splice sites after in vitro AO treatment has also been observed. We have reported the activation of a cryptic donor splice site after treatment with an AO targeting *LMNA* pre-mRNA, promoting removal of 150 nt from the end of exon 11^16^. This precisely replicates the alternative *LMNA* transcript isoform that was reported to arise from recurrent pathogenic mutations within the cryptic splice motif^17^. Evers et al.^18^ observed that an AO targeting exon 9 in *ATXN3* promoted a partial exon 9 skip, activating an alternative 5′ss. A partial exon 12 skip in the *HTT* transcript was also detected after treatment with an AO (World Patent WO2015053624A2); once again activating a cryptic donor splice site^19,20^, this time one that was previously observed to be used at low levels (3.2% of full-length) in normal human embryonic stem cells^21^. Lastly, we recently reported activation of two cryptic donor splice sites by AOs containing several locked nucleic acid residues, designed to enhance efficiency of exon skipping from the dystrophin transcript^22^.

In addition to the established roles that splice site motifs and exon enhancer and silencer motifs play in directing RNA splicing, there is increasing evidence of a similar role for RNA secondary structure^23–26^, and of its effect on splice factor binding^27,28^. While modelling the interactions of these phenomena presents a highly complex challenge, a reasonable starting point may be to assume that RNA secondary structure is generally antagonistic to splice factor binding within closed regions.

In our laboratory’s quest to develop new therapeutics for debilitating genetic diseases, we have tested thousands of AOs targeted to numerous gene transcripts in a variety of cell types. We have confirmed AO-induced cryptic splicing events in the target transcripts in less than 0.2% of human cells, and only a single example in mouse cells^29^. In this study, we investigated the possible mechanisms by which AOs may induce cryptic splicing. We analyzed 12 AOs targeting six different human gene transcripts and found that changes to the accessibility of enhancer and silencer motifs within the transcript secondary structure appeared to play a role in many cases. The diverse nature of these changes indicates that there may be multiple pathways to inducing cryptic splicing, sometimes within a single exon.

## Results and discussion

To explore the possible mechanisms behind cryptic splice site activation, we analyzed AO-induced cryptic splicing events in six different human transcripts: *COL7A1, SRSF2, ATXN3, USH2A, HTT,* and *LMNA*. Data for *HTT* and *LMNA* were obtained from the literature and analyzed together with those from the remaining transcripts.

### Analysis of antisense oligonucleotide treatment

#### COL7A1 exon 15

**Figure 1:**
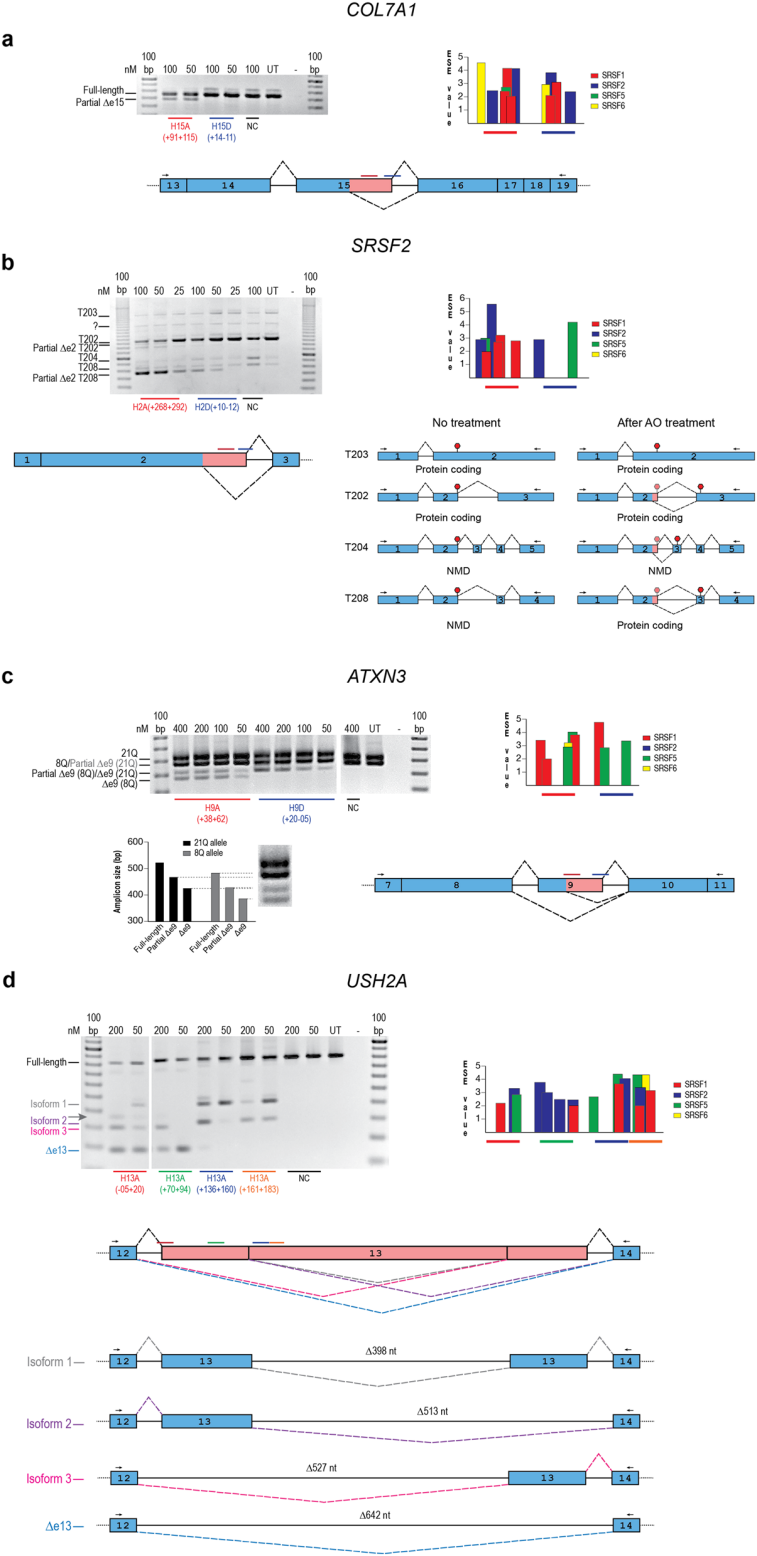
Activation of cryptic splice sites by AO-mediated splice switching in four different gene transcript targets. (**a**) *COL7A1* exon 15. (**b**) *SRSF2* exon 2*.* (**c**) *ATXN3* exon 9*.* (**d**) *USH2A* exon 13**.** Reverse transcription-PCR analysis after transfection with antisense oligonucleotides (AOs), at various nM concentrations indicated above the gel image. Exon splice enhancer (ESE) motifs, predicted by ESEfinder 3.0^34^. The color code indicates the putative binding sites for serine/arginine rich splicing factors (SRSFs). Blue boxes represent exons, lines between the boxes represent introns, dashed lines above and below represent various splicing events, pink boxes represent the portion of exon removed after the activation of a new cryptic splice site, black arrows indicate primer location and direction, coloured lines indicate AO target binding site, red polygons represent termination codons, pink polygons represent termination codons removed after cryptic splice site activation. Alternative transcript exon composition before and after *SRSF2* AO treatment. Multiple transcript isoforms noted as T### according to Ensembl. Grey arrow indicates an amplicon that could not be succesfully isolated and sequenced. NC, negative control sequence synthesized as 2′-OMe PS; UT, untreated; 100 bp, 100 base pair DNA ladder; nM, nanomolar. The gel images were cropped for presentation. Full-length gel images are presented in Supplementary Fig. S3.

Antisense oligonucleotides (2′-*O-*methyl modified bases on a phosphorothioate backbone, (2´-OMe PS)) were transfected into healthy human fibroblasts as cationic lipoplexes at concentrations of 100 and 50 nM to induce skipping of exon 15 from the *COL7A1* pre-mRNA transcript, removing 144 nt from the full-length transcript (Fig. 1a). Subsequent RT-PCR analysis from exons 13 to 19 revealed both the full-length transcript and an unanticipated amplicon, smaller than full-length but larger than would be expected as a result of complete exon 15 removal. The unexplained amplicon was isolated and identified by Sanger sequencing to be missing the last 64 nucleotides from the 3′ end of exon 15 (Supplementary Fig. S1). Removing 64 nt from the *COL7A1* transcript would render the cryptically spliced product out-of-frame, and therefore produce a premature termination codon in exon 16. This discovery highlights the importance of investigating unexpected splicing products after AO treatment. A new donor splice site was activated by treatment with an AO targeting *COL7A1* exon 15, H15A(+91+115), that resulted in cryptic splice site activation in 30% of the transcripts after transfection of the AO at both 100 nM and 50 nM. Treatment with this AO did not induce other aberrant splicing products. Transfection of cells with an AO covering the authentic donor splice site, H15D(+14–11), did not lead to cryptic donor site activation. Cryptic splice site activation was induced after the H15A(+91+115) AO was transfected into an immortalized human keratinocyte cell line (HaCaT) as cationic lipoplexes at concentrations of 400, 200, 100 and 50 nM, indicating that cryptic splice site activation after treatment with this AO is not cell-specific (Supplementary Fig. S2).

#### SRSF2 exon 2

Antisense oligonucleotides were transfected into healthy human fibroblasts as cationic lipoplexes at concentrations of 100, 50 and 25 nM to induce skipping of exon 2 from the *SRSF2* pre-mRNA transcript, removing 311 nt from the full-length transcript (Fig. 1b). Gel fractionation of the RT-PCR amplicons revealed several products confirmed by Sanger sequencing: full-length SRSF2-T204 (ENST00000452355.7); full-length SRSF2-T208 (ENST00000585202.5); and T208 missing 65 nt from the 3′ end of exon 2. Multiple amplicons larger than 1000 nt were present that correspond to the amplicon sizes of the transcripts SRSF2-T203 (ENST00000392485.2) and SRSF2-T202 (ENST00000359995.10) (Fig. 1b). The splicing of T202 appears to be influenced by the AOs in the same manner (Fig. 1b). However, we were unable to isolate and identify various amplicons to confirm this. The AOs did not appear to cause exon skipping or cryptic donor site activation within the T203 transcript, most likely due to the T203 isoform containing only two exons, making both “unskippable”^30^. Cryptic splice site activation was induced after both H2A(+268+292) and H2D(+10–12) AOs were transfected into HaCaT cells and a human neuroblastoma cell line (SH-SY5Y) as cationic lipoplexes at concentrations of 400, 200, 100 and 50 nM, indicating that cryptic splice site activation after treatment with these AOs is not cell-specific (Supplementary Fig. S2).

Under normal conditions, *SRSF2* transcript isoforms T202 and T203 code for proteins while T208 and T204 undergo nonsense mediated decay (NMD). After AO treatment, the expression of the cryptically spliced T208 increased with a concomitant decrease in the full-length T202. The cryptic splicing of exon 2 removes the natural termination codon from T202, T204, and T208 and exposes a new in-frame termination codon in the following exon of each transcript (Fig. 1b).

Mammalian NMD generally follows the ‘50 nucleotide rule’, whereby termination codons more than 50 nt upstream of the final exon are determined premature and result in a reduction in mRNA abundance^31^. Cryptic splice site activation appears to stabilize T208 as a new termination codon is created within 50 nt of the penultimate 3′ exon junction. Isoform T204 still appears to undergo NMD, as the new termination codon is exposed within the third exon of the five-exon isoform.

#### ATXN3 exon 9

Antisense oligonucleotides were transfected into healthy control human fibroblasts as cationic lipoplexes at concentrations of 400, 200, 100 and 50 nM to induce skipping of exon 9 from the *ATXN3* pre-mRNA, thereby removing 97 nt from the full-length transcript (Fig. 1c). Gel fractionation of the RT-PCR amplicons revealed two full-length product bands representing the two transcripts in the untreated sample: a larger product (533 nt) containing 21 CAG (21Q) repeats and a slightly smaller product (483 nt) containing eight CAG (8Q) repeats. Two additional smaller bands were observed in healthy human fibroblasts treated with H9A(+38+62) at all concentrations tested. The two bands were isolated and identified by Sanger sequencing (Supplementary Fig. S1). The smaller of the two amplicons was solely the result of complete exon 9 skipping from the 8Q transcript. The larger of the two amplicons is a similar size to complete exon 9 removal from the 21Q transcript. However, this amplicon was confirmed as resulting primarily from the activation of a cryptic donor site on position + 42 of exon 9, removing 55 nt from the 8Q transcript. Sanger sequencing revealed a minor secondary product with the removal of exon 9 entirely from the 21Q transcript. Treatment with H9D(+20–05) resulted in predominantly partial exon 9 skipping from the 8Q transcript and a low level of complete exon 9 skipping from the 21Q transcript.

Complete and partial exon 9 skipping was predominately observed in the 8Q compared with the 21Q transcript. Partial exon 9 skipping from the 8Q transcript and complete exon 9 skipping from the 21Q transcript produces products that differ by three nucleotides, and could not be distinguished on an agarose gel alone. Sanger sequencing confirmed that both transcripts were disproportionately represented, with lower levels of complete exon 9 skipping from the 21Q transcript. Partial exon 9 skipping from the 21Q transcript produces a product 16 nt smaller than the canonical 8Q transcript and could not be confirmed by the methods used in this study. Cryptic donor activation in the transcript with fewer CAG repeats dominates in some AO treatments but not others^32,33^. The CAG expansion occurs in the following exon 10, separated by a 10 kb intron from the AO target. Numerous studies assessing AO-mediated removal of exon 9 and/or exon 10 from the *ATXN3* transcript reported reduced exon skipping efficiencies the larger the expansion size. Although this phenomenon is directed more towards exon 10 removal, we speculate that the CAG repeat length may influence the cryptic splice site usage frequency. The nature of the CAG repeat allows for numerous consecutive potential serine/arginine-rich splicing factor (SRSF) 2 (AGCAG) and SRSF5 (ACAGC) splice motifs. The fact that these positive exon selection sites are heavily repeated may influence exon 10 and potentially exon 9 selection and, therefore, susceptibility to AO-mediated exon skipping.

As *ATXN3* is ubiquitously expressed, AO-mediated cryptic splice site activation was tested in both HaCaT and SH-SY5Y cells. The number of repeats for each cell line was determined via Sanger sequencing: heterozygous for 19Q and 18Q transcripts in the HaCaT cells and homozygous for 10Q transcript in the SH-SY5Y cells. Antisense oligonucleotides were transfected as cationic lipoplexes at concentrations of 400, 200, 100 and 50 nM (Supplementary Fig. S2). The H9D(+20–05) AO targeting the donor site activated the cryptic 3′ss in both cell lines, but cryptic splice site activation was not apparent after treatment with the H9A(+38+62) AO. Although, without testing both AOs in multiple cell types from the same healthy control donor, it cannot be determined if the discrepancy in cryptic splice site activation is due to the cell type or some other factors.

#### USH2A exon 13

Antisense oligonucleotides were transfected into a Huh7 cell line as cationic lipoplexes at concentrations of 200 and 50 nM to induce skipping of exon 13 from the *USH2A* pre-mRNA transcript (Fig. 1d). Subsequent RT-PCR analysis revealed multiple unanticipated amplicons larger than expected from the removal of exon 13 in its entirety. It was confirmed by Sanger sequencing that multiple splicing events occurred: removal of the complete exon 13 (Fig. 1d ∆e13); activation of a cryptic donor (Fig. 1d isoform 2); activation of a cryptic acceptor (isoform 3); or activation of both cryptic donor and acceptor sites within exon 13 (Fig. 1d isoform 1), after treatment with different AOs (Supplementary Fig. S1). Treatment with H13A(−05+20) and H13A(+70+94) resulted mainly in complete exon 13 exclusion, removing 642 nt from the full-length transcript (Fig. 1d ∆e13), and the activation of a cryptic acceptor site, removing 527 nt from the full-length transcript (Fig. 1d isoform 3). Treatment with H13A(+136+160) and H13A(+161+183) resulted in the activation of a cryptic donor site, both on its own (missing 513 nt from the 3′ end of exon 13; Fig. 1d isoform 2) and in conjunction with the cryptic acceptor site (missing 398 nt from the middle of exon 13; Fig. 1d isoform 1) but did not remove the entire exon 13. We were unable to isolate and identify one of the amplicons by Sanger sequencing (labelled with a grey arrow in Fig. 1d). We speculate that this amplicon is a heteroduplex, which would explain why it could not be isolated.

The *USH2A* expression profile is limited to a small subset of tissue types (eye, heart muscle, liver, and testis) that were not available for use at the time of this study. Thus AO-induced cryptic splicing was not investigated in additional cell types.

#### LMNA exon 11

Lou et al.^16^ sought to induce cryptic splicing through AO-mediated splice-switching by designing a panel of AOs to anneal across exon 11 of the *LMNA* gene transcript in human myogenic cells. Initially, 2′-OMe PS AOs were tested at concentrations of 400, 200 and 100 nM as cationic lipoplexes. The transfection of several different AOs resulted in the cryptically spliced ∆150 transcript and whole exon 11 removal. Transfection of the H11A(+221+245) AO sequence resulted predominantly in ∆150 transcript expression and was thus synthesized as a phosphorodiamidate morpholino oligomer, producing even more specific and potent cryptic splicing activation. This finding, along with the ability of AOs containing several locked nucleic acids to activate cryptic donor splice sites from the dystrophin transcript^22^, highlights that cryptic splicing can be activated by AOs comprised of various backbone chemistries and sugar modifications.

#### HTT exon 12

As a potential treatment for Huntington’s disease, a 2′-OMe PS AO was developed to reduce the levels of toxic huntingtin protein by activating a cryptic donor splice site, removing 135 nt from the 3′ end of exon 12^19,20^. Antisense oligonucleotides were transfected into Huntington’s disease patient-derived fibroblasts as cationic lipoplexes at various concentrations and resulted in a dose-dependent partial exon 12 skipping (150 nM 95% skipping; 25 nM 92% skipping, and 1 nM 16% skipping) except at the highest concentration where no exon skipping was evident (1000 nM 0% skipping)^20^.

### Analysis of splice site scores and exonic splicing enhancer motifs masked by the examined antisense oligonucleotides

Two models were employed to calculate the scores of both the canonical and cryptic splice sites activated after AO treatment: a weight matrix model, Human Splice Finder 3.1^34^, and a maximum entropy model, MaxEntScan^35^. No discernable pattern became evident using either model (Table 1), indicating splice site scores are not the only factor influencing splice site usage. Various cryptic splice site scores were higher when compared to canonical splice site scores, but again, with the small number of examples covered in this study, no pattern could be deduced. Included in Table 1 are the canonical and cryptic splice site sequences recognized by the spliceosome in the examples reported here. The CAG cryptic 3′ss is activated in the *USH2A* transcript after AO treatment. During U2-type canonical splicing of human transcripts, CAG 3′ss are more frequently used by the spliceosome than TAG 3′ss (64.55% versus 29.01%)^36^. Except for the *USH2A* transcript, all the studied activated cryptic 5′ss comprise the CAGgt sequence. Additionally, the canonical and cryptic 5′ss are strikingly similar in the *LMNA* example.

**Table 1:** Comparing canonical and cryptic splice site scores using two different modeling approaches.

Gene (exon)	Splice site	Canonical splice site sequence	Cryptic splice site sequence	HSF canonical splice site score	HSF cryptic splice site score	MaxEnt canonical splice site score	MaxEnt cryptic splice site score	Position relative to beginning of exon
*USH2A* (13)	Acceptor	ttttatctttagGG	caacactgccagAT	88.04	80.44	8.95	− 1.01	+ 527
	Donor	CAGgtaaga	AGTgtgagt	97.66	82.16	10.77	4.88	+ 129
*COL7A1* (15)	Donor	CGGgtcagg	CAGgtggct	88.19	78.49	4.01	2.97	+ 80
*ATXN3* (9)	Donor	AAAgtaaag	CAGgtacaa	74.37	**76.7**	1.6	**7.09**	+ 42
*SRSF2* (2)	Donor	TAAgtaatg	CAGgtcgcg	73.19	72.69	− 0.64	**5.46**	+ 246
HTT* (12)	Donor	ATTgtaagt	CAGgtcagc	83.43	**92.8**	7.16	**8.54**	+ 206
LMNA* (11)	Donor	CAGgtgagt	CAGgtgggc	98.84	88.33	8.07	2.93	+ 120

Cryptic splice site scores that are higher than the canonical splice site scores are highlighted in bold.

Exonic splicing enhancer (ESE) motifs masked by AO binding sites were tallied using ESEFinder 3.0^37^; (Fig. 1; Table 2). Motifs were considered when one or more motif nucleotides were masked by the targeting AO, as even partially covering a motif by two nucleotides influences splice outcome^38^. The examined AOs were found to consistently mask SRSF1 motifs, with exception of the AO H2D(+10–12) targeting the *SRSF2* exon 2 donor site.

**Table 2:** Exonic splicing enhancer motifs masked by the antisense oligonucleotides examined in this study.

Gene	AO nomenclature	SRSF1 (SF2)	SRSF2 (SC35)	SRSF5 (SRp40)	SRSF6 (SRp55)
*USH2A*	H13A(−05+20)	1	1	1	0
	H13A(+70+94)	1	4	0	0
	H13A(+136+160)	1	1	2	0
	H13A(+161+183)	2	2	1	1
*COL7A1*	H15A(+91+115)	3	2	1	1
	H15D(+14–11)	2	2	0	1
*ATXN3*	H9A(+38+62)	3	0	2	1
	H9D(+20–05)	1	0	2	0
*SRSF2*	H2A(+268+292)	4	2	1	0
	H2D(+10–12)	0	1	1	0
*HTT*	H12A(+269+297)^a^	3	4	3	0
*LMNA*	H11A(+221+245)^a^	3	3	1	1

^a^Not tested in this study; published results.

The splicing factor SRSF1 is necessary for several splicing processes, including lariat formation and 5′ss cleavage^39^. In addition, SRSF1 assists in modulating 5′ss selection^39^. The addition of purified SRSF1 to cultured cells favored 5′ss located more proximally to the 3′ss while lower levels of SRSF1 favored 5′ss located distal to the 3′ss^40^. In our study, AOs can mask the availability of ESE motif binding sites, therefore reducing the amount of SRSF1 that can bind to the pre-mRNA. Fewer SRSF1 binding sites may drive the 5′ss preference away from the canonical splice site towards a more distal cryptic splice site.

### Analysis of AO-induced changes to exonic splicing enhancer/silencer access within cryptically spliced exons

It is notable that all seven of the observed cryptic splice sites fell within the affected exons, between the canonical splice sites, rather than in the downstream or upstream introns. We suggest that this is a logical consequence of the ‘exon definition’ paradigm under which the human spliceosome is thought to operate, whereby transcript sequence between the first and last exons is processed as intron unless specifically defined as being part of an internal exon^41^. Because ‘intron’ is the default sequence identity under this paradigm, AO binding is therefore much more likely to diminish an existing exon signal than it is to spontaneously extend it.

Because four of the seven cryptic splice sites had MaxEnt scores lower than their canonical counterparts, it was clear that our analysis would need to encompass other variables in order to explain the activation of these sites—specifically, those variables that could plausibly be altered by AO binding. We therefore attempted to model the effect that AO binding would have on both the local secondary structure of the transcript, and the subsequent change in accessibility to ESE and exon splicing silencer (ESS) motifs.

Two of the eleven cryptic-splice inducing AOs, SRSF2 H2D(+10–12) and ATXN3 H9D(+20–05), were excluded from this modelling, as we reasoned that simple steric blocking of the target exon donor sites was the most likely explanatory mechanism in those cases.

**Figure 2:**
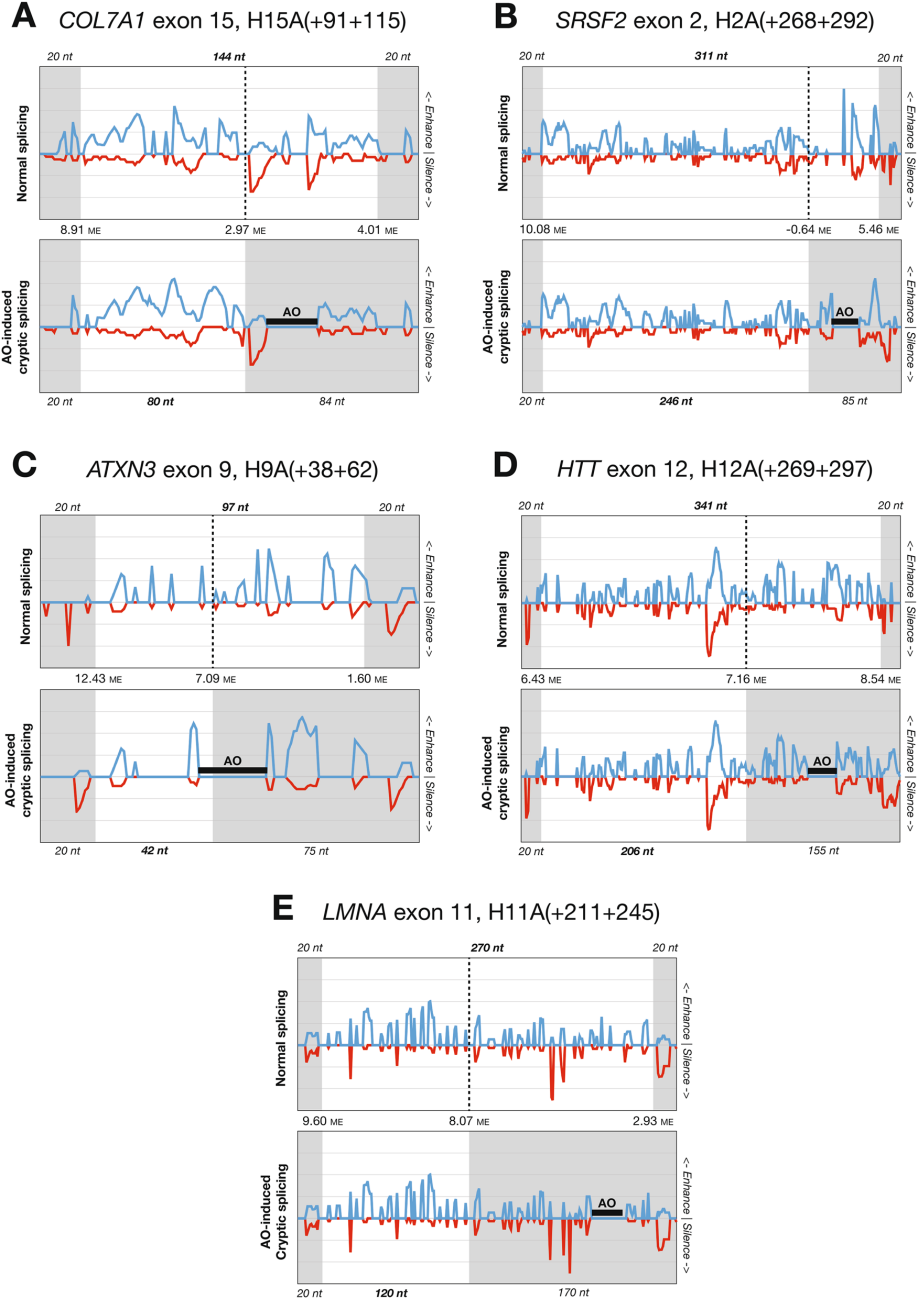
Changes to predicted exon splicing enhancer/silencer (ESE/ESS) access in five examples of antisense oligonucleotide (AO)-induced cryptic splicing of canonical exons. Blue lines indicate ESE access and red lines indicate ESS access. Grey shading indicates pre-mRNA sequence excluded from the mature transcript. Region sizes and Maximum Entropy scores for cryptic and canonical splice sites are also shown.

The ESE and ESS motifs for each cryptically spliced exon were overlaid to generate enhancer and silencer scores at each nucleotide position. These values were then “masked” by the predicted secondary structure for the exons, effectively resetting the ESE and ESS scores to zero for all nucleotides predicted to bind other nucleotides. This masking was repeated with the altered structures predicted for on-target AO binding, and the two plots were vertically aligned to allow comparison between them (Fig. 2A–E). Because the size of *USH2A* exon 13 (642 nt) made it impractical to visually compare changes in its ESE and ESS access in the same manner as for the other exons, we elected to present only the net changes in ESE and ESS access as a result of AO binding (Fig. 2F–G).

We acknowledge that there are impediments to the accuracy of this approach. Individually, HSF 3.1 and RNAfold are imperfect predictors that encompass only a fraction of the RNA interactions occurring within living cells, and neither account for more complex factors, such as RNA tertiary structure or local ribonucleoprotein context. However, despite their limitations, these two utilities have proven instrumental for numerous scientific reports over the past decade and have amassed a combined total of over 4000 citations. We therefore reasoned that integrating the predictions of these two well-tested programs might prove more informative than their individual outputs.

In *COL7A1* exon 15 (Fig. 2A), AO binding was predicted to increase ESE access in the retained 5′ segment, as well as directly competing with ESEs in the excised 3′ segment. The net effect was a much stronger exon signal from the 5′ segment that improved the profile of the cryptic donor site. This example demonstrates that blocking an authentic donor site does not automatically activate a cryptic donor site; additional elements, including secondary structure and exon and intron definition motifs, are necessary to define the exon boundary.

For *SRSF2* exon 2 (Fig. 2B), the AO directly obscured the strongest enhancer peak in the excised 3′ segment and induced a moderate increase in ESE access within the retained 5′ segment. We also observed that, in the absence of AO binding, the enhancer signal in the excised 3′ segment of the exon was substantially stronger than in the rest of the exon. This may be a positively selected feature to ensure inclusion of this segment and avoidance of the cryptic splice site, though it is not clear why the very poor MaxEnt score of the cryptic donor is not a sufficient deterrent alone.

In *ATXN3* exon 9 (Fig. 2C), the AO binding site overlapped the cryptic donor site and caused loss of ESE access 3′ of the cryptic donor and a slight increase of ESE access immediately 5′ of the cryptic donor. This, combined with the much stronger MaxEnt score of the cryptic site, may have been enough to shift exon definition to the 5′ region of the exon. Partial occlusion of the cryptic donor site by this AO may also explain why it induces whole exon skipping in some fibroblast transcripts (Fig. 1c), as this would sterically block spliceosome binding.

In *HTT* exon 12 (Fig. 2D), the changes in secondary structure did not clearly favor either enhancement or silencing of the excised segment. However, ESS access was increased both 5′ and 3′ of the canonical donor site, and this appears to have been sufficient to tip the balance towards the comparably strong cryptic donor splice site.

A similar change to *HTT* exon 12 appears to have occurred in *LMNA* exon 11 (Fig. 2E), with the exception that the cryptic donor site in this exon was much stronger than its canonical neighbor.

**Figure 3:**
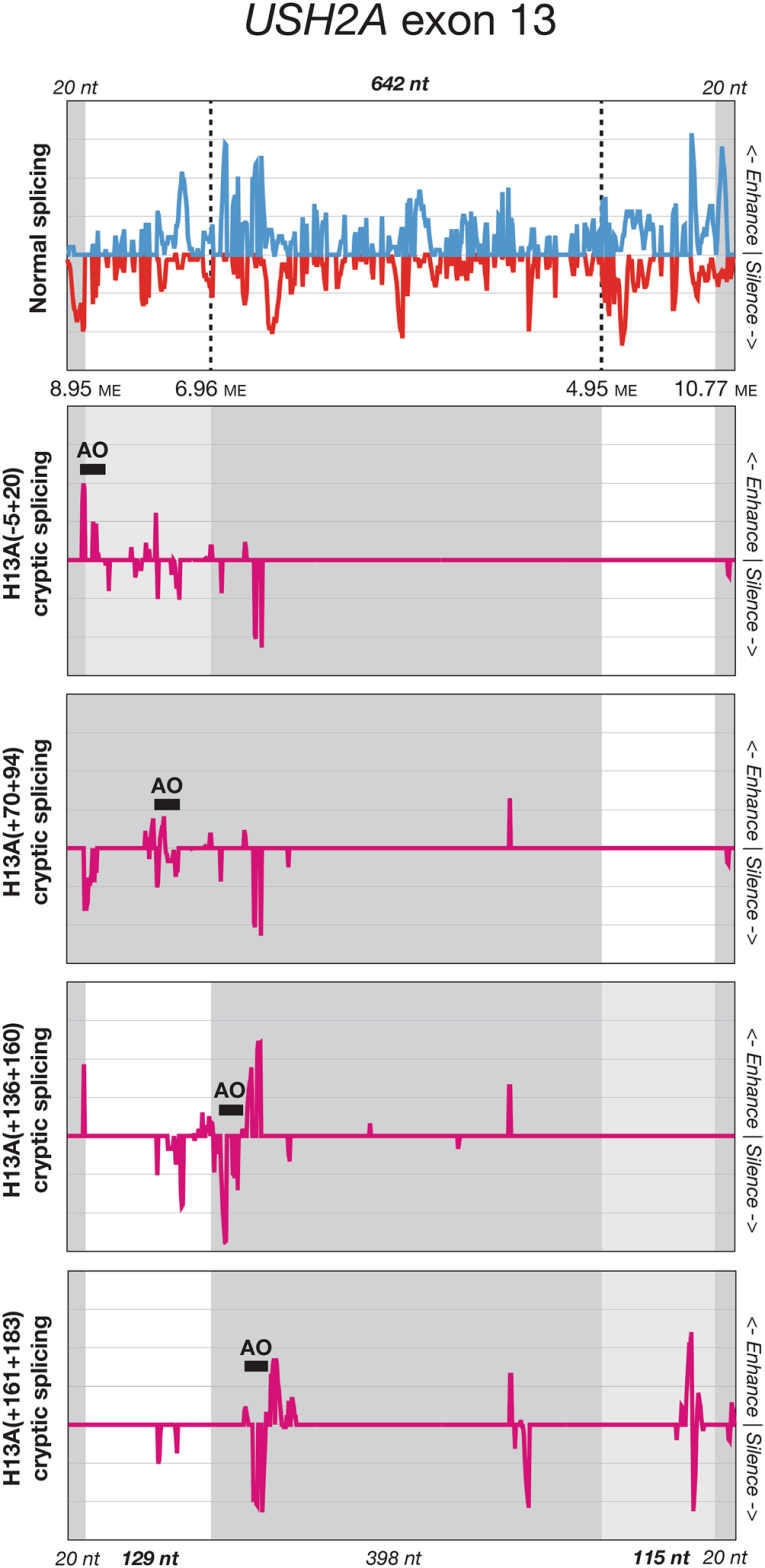
Net changes to predicted exon splicing enhancer/silencer (ESE/ESS) access in four examples of antisense oligonucleotide (AO)-induced cryptic splicing of *USH2A* exon 13. Blue lines indicate ESE access and red lines indicate ESS access, purple indicates the net change in ESE and ESS access as a result of AO binding. Grey shading indicates pre-mRNA sequence excluded from the mature transcript, and pale grey indicates regions with intermediate inclusion. Region sizes and Maximum Entropy scores for cryptic and canonical splice sites are also shown.

For *USH2A* exon 13 (Fig. 3), all four AOs induced use of varying combinations of the two canonical splice sites, an internal cryptic donor site, and an internal cryptic acceptor site. In examining the effects of the four AOs, we noted that they appeared to group together as two pairs. The first two AO sequences, H13A(−05+20) and H13A(+70+94), were targeted 5′ of the cryptic donor site and predominantly induced splice-switching from the canonical to the cryptic acceptor site. Conversely, the second two AOs, H13A(+136+160) and H13A(+161+183), were both targeted 3′ to the cryptic donor site and induced its activation, splice-switching away from the canonical donor site. This is consistent with our earlier observation that the orientation of the AO target site relative to the cryptic donor site appears to be a key determinant of the AO's effect. The second pair of AOs primarily act to enhance the 5′ cryptic donor site, in much the same way as the examples shown in Fig. 2, while the first pair of AOs act to silence the canonical acceptor site. Both these splicing effects are further complicated by the presence of the internal cryptic acceptor site that provides an alternative partner for the canonical donor site, and by the distance between the two cryptic sites (398 nt), which allows sufficient separation for both to be activated within the same transcript (see also Fig. 1d, isoform 1).

It appears that some facets of exon definition are unique to large internal exons and that these can only be properly understood by studying splicing in similarly sized exons from other genes. Exons longer than 500 nt, such as *USH2A* exon 13, typically rely on intron definition rather than exon definition in order to achieve correct splicing, but this intron-defined splicing can become inefficient when the intron size exceeds 500 nt^42,43^. It is possible that sporadic splice site activation in this larger exon is partly due to the inability of the spliceosome to utilize intron definition, and thus inefficiently creates exon isoforms of less than 500 nt by activating various internal splice sites, regardless of their strength.

There is accumulating evidence that long non-coding RNA (lncRNA) plays a role in post-transcriptional modification, including splicing^44^. In most cases, lncRNA contains sequence motifs or scaffolds that can recruit splicing factors to promote or restrict splicing^44^. We cannot rule out that the introduction of AOs to the cells may have caused a disturbance to the lncRNA and led to the observed cryptic splicing. It is also possible that the AOs have become part of the splicing complex as non-coding RNA and shifted the whole paradigm. At this stage, the results are inconclusive as only the AOs targeting *COL7A1*, *SRSF2* and *HTT* showed some similarity towards lncRNAs with no mention of splicing involvement.

## Conclusions

Despite the small number of examples of AO-induced cryptic splicing, we observed considerable diversity in the etiology of this phenomenon. However, a common feature appears to be disruption of the exon definition signal.

It is clear that canonical exon definition is achieved not by any single motif, but by the cumulative signal of multiple enhancers binding with regularity and consistency along the entire exon span. Furthermore, continuity of this enhancing signal appears to be just as important, if not more important, than its overall strength. This continuity is especially crucial when the exon contains a cryptic splice site, as this is often the only metric by which the spliceosome can distinguish the cryptic site from its canonical neighbor.

## Methods

All methods were carried out in accordance with relevant guidelines and regulations.

### Antisense oligonucleotides (AOs)

Antisense oligonucleotides (AOs) comprising of 2′-*O*-methyl modified bases on a phosphorothioate backbone (2′-OMe PS) were synthesized by TriLink BioTechnologies (San Diego, CA) or synthesized in-house on an Expedite 8909 Nucleic Acid synthesizer (Applied Biosystems, Melbourne, Australia) using the 1 µmol thioate synthesis protocol, as described previously^45^. After synthesis, the oligonucleotides were cleaved from the support following incubation in ammonium hydroxide for a minimum of 24 h at room temperature. The 2′-OMe PS AOs were subsequently desalted under sterile conditions on NAP-10 columns (GE Healthcare, Sydney, Australia) according to manufacturer’s instructions. The 2′-OMe PS AOs used in this study are listed in Table 3. Oligonucleotide nomenclature is based on that described by Aung-Htut et al.^46^ and Mann et al.^47^, indicating the intron:exon, exon or exon:intron annealing coordinates in the target gene pre-mRNA.

**Table 3:** Information for AOs.

Gene	AO nomenclature	Sequence (5′ to 3′)
*USH2A*	H13A(−05+20)	GCAAUGAUCACACCUAAGCCCUAAA
	H13A(+70+94)	GAGCCAUGGAGGUUACACUGGCAGG
	H13A(+136+160)	UGAAGUCCUUUGGCUUCUUUUUUGC
	H13A(+161+183)	AGUUUUCUCUGCAGGUGUCACAC
*COL7A1*	H15A(+91+115)	CCCUCCUCUCUGCCUCGCAGUACCG
	H15D(+14–11)	CAGGGCCUGACCCGUUCGAGCCACG
*ATXN3*	H9A(+38+62)	UUCUGAAGUAAGAUUUGUACCUGAU
	H9D(+20–05)	UUUACUUUUCAAAGUAGGCUUCUCG
*SRSF2*	H2A(+268+292)	CGCUCCUUCCUCUUCAGGAGACUUG
	H2D(+10–12)	CCCAGACAUUACCAUUUUCUUA
*HTT*	H12A(+269+297)^a^^20^	CGGUGGUGGUCUGGGAGCUGUCGCUGAUG
*LMNA*	H11A(+221+245)^a^^16^	AGGAGGUAGGAGCGGGUGACCAGAU
	Negative control	CCUCUUACCUCAGUUACAAUUUAUA

^a^Not tested in this study; published results.

### Cell culture and transfection

All cell culture reagents were purchased from Gibco, (Thermo Fisher Scientific, Scoresby, Australia), unless otherwise stated. Primary dermal fibroblasts were derived from a healthy volunteer after informed consent (The University of Western Australia Human Research Ethics Committee approval RA/4/1/2295; Murdoch University Human Research Ethics Committee approval 2013/156). The human hepatocarcinoma cell line, Huh7, was supplied by the Japanese Collection of Research Bioresources Cell Bank (Osaka, Japan) and purchased from CellBank Australia (Westmead, Australia). The human neuroblastoma cell line, SH-SY5Y, was supplied by ATCC (Gaithersburg, MD) and purchased from In Vitro Technologies (Canning Vale, Australia). HaCaT cells were purchased from AddexBio (San Diego, CA). Culture conditions and transfection seeding density are described in Table 4.

**Table 4:** Culture conditions for cell strains used in the study.

Cell strain	Propagation/seeding media	Transfection seeding density
Primary dermal fibroblasts	Dulbecco’s modified Eagle’s medium supplemented with 1% GlutaMax™-I and 10% FBS	1.8 × 10^4^ cells/well
Huh7	Dulbecco’s modified Eagle’s medium supplemented with 10% FBS	5 × 10^4^ cells/well
SH-SY5Y	1:1 mixture of Eagle’s Minimum Essential Medium and Ham’s F-12 medium supplemented with 10% FBS	7 × 10^4^ cells/well
HaCaT	Dulbecco’s modified Eagle’s medium supplemented with 10% FBS	3 × 10^4^ cells/well

All cells were maintained at 37 °C in a 5% CO_2_ atmosphere. Cells were seeded 24 h before transfection in a 24-well plate. Fetal bovine serum (FBS) (Scientifix, Cheltenham, Australia).

All cell strains were transfected with 2′-OMe PS AO-Lipofectamine 3000 (Thermo Fisher Scientific) lipoplexes in Opti-MEM (Gibco) according to the manufacturer’s instructions, at various concentrations in duplicate wells, and the cells were then incubated at 37 °C in a 5% CO_2_ atmosphere for 24 h before RNA extraction. The negative control oligomer (sequence from Gene Tools, LLC synthesized as a 2′-OMe PS AO) that targets a human beta-globin intron mutation was used as a negative transfection control.

### Molecular analysis

After harvesting the cells, total RNA was extracted using MagMax nucleic acid isolation kit (AM1830; Thermo Fisher Scientific) according to manufacturer’s instructions and included the DNase treatment step. Molecular analyses were accomplished using three different systems optimized for different gene targets. SuperScript III One-Step RT-PCR System with Platinum *Taq* DNA Polymerase (Thermo Fisher Scientific) was used to synthesize and amplify cDNA from 50 ng of total RNA in a single step. Nested PCR was necessary to amplify the *USH2A* transcripts. Briefly, after 20 cycles of amplification, 1 µl aliquot was removed and subjected to nested PCR for 25 cycles using AmpliTaq Gold (Thermo Fisher Scientific) and an inner primer set. For regions with a high GC-content that are more difficult to amplify, SuperScript IV First-Strand Synthesis System and random hexamers (Thermo Fisher Scientific) were used to synthesize cDNA from harvested total RNA, and approximately 50 ng of cDNA was used as a template for PCR amplification using the TaKaRa LA Taq DNA Polymerase with GC Buffer II system (Takara Bio USA, Inc., Clayton, Australia). PCR systems, conditions and primers used to assess splice modulation across the different gene transcripts are summarized in Table 5.

**Table 5:** List of primers, PCR system and conditions used in this study.

Gene target (accession numbers)	Primer orientation	Sequence (5′–3′)	Length (nt)	PCR system	Cycling conditions
*ATXN3* (NM_004993.6)	Exon 7F	GTCCAACAGATGCATCGACCAA	522 (21Q) 516 (19Q) 513 (18Q) 489 (10Q) 483 (8Q)	SSIII One-Step	55 °C (30 min) and 94 °C (2 min); 28 cycles of 94 °C (30 s), 55 °C (30 s) and 68 °C (1.5 min)
	Exon 11R	AGCTGCCTGAAGCATGTCTTCTT			
*COL7A1* (NM_000094.4)	Exon 13F	CTTAGCTACACTGTGCGGGT	765	SSIII One-Step	55 °C (30 min) and 94 °C (2 min); 30 cycles of 94 °C (30 s), 60 °C (30 s) and 68 °C (1.5 min)
	Exon 19R	TGGGAGTATCTGGTGCCTCA			
*SRSF2* (XR_429913.4)	Exon 1F	CCCAGAGCTGAGGAAGCC	850	SSIV TaKaRa GC I	94 °C (1 min); 32 cycles of 94 °C (30 s), 62 °C (30 s) and 72 °C (4 min)
	Exon 4R	CTCAACTGCTACACAACTGC			
*USH2A* (NM_206933.4)	Exon 12F	AAGAGTTGGATCCTGATGGCTGC	993	SSIII One-Step	55 °C (30 min) and 94 °C (2 min); 20 cycles of 94 °C (15 s), 60 °C (30 s) and 68 °C (1 min)
	Exon 15R	GACAGGTTTCATTCAAGGCTCC			
	Exon 12F	CTGTAACTGCAATACCTCTGG	837	AmpliTaq Gold	94 °C (5 min); 25 cycles of 94 °C (30 s), 60 °C (30 s) and 72 °C (1 min); 72 °C (5 min)
	Exon 14R	CAAACACACTGACCAGTCAGG			

Amplified RT-PCR products were resolved on 2% agarose gels by electrophoresis in Tris–acetate ethylenediaminetetraacetic acid buffer, compared to a 100 bp DNA size standard (Geneworks, Adelaide, Australia). Relative transcript abundance was estimated by densitometry on images captured by the Fusion FX system (Vilber Lourmat, Marne-la-Vallée, France) using Fusion-Capt software and ImageJ (version 1.8.0_112) software for densitometry analysis. To identify RT-PCR products, the amplicons were first isolated by bandstab^48^, followed by template preparation using Diffinity RapidTip for PCR Purification (Diffinity Genomics, Inc., West Henrietta, NY) and DNA sequencing, performed by the Australian Genome Research Facility Ltd. (Nedlands, Australia).

### In silico analysis

Basic Local Alignment Search Tool (BLAST)^49^ was used to compare amplicon sequences to the reference mRNA sequences (accession numbers: Table 5). ESEFinder 3.0^34^ was used to evaluate ESE motifs masked by AO binding sites. Motifs were considered when one or more motif nucleotides were masked by the targeting AO. Human Splice Finder 3.1^34^ and MaxEntScan^35^ were employed to calculate the scores of both the canonical and cryptic splice sites activated after treatment with each AO. Sequences for each cryptically spliced exon and ± 20 nt of flanking intron were input to Human Splice Finder 3.1^34^ which generated a JSON file with the locations of every detected ESE and ESS motif, as well as predicted acceptor and donor splice sites. Raw text from this JSON file was then imported into a custom-made spreadsheet (see Supplementary Material) that used this data to assign an ESE and an ESS score to each nucleotide of the sequence, under the following rationale:

- ESE score: + 1/n for each overlapping ESE motif, where n = ESE motif length.
- ESS score: − 1/n for each overlapping ESS motif, where n = ESS motif length.

**Figure 4:**
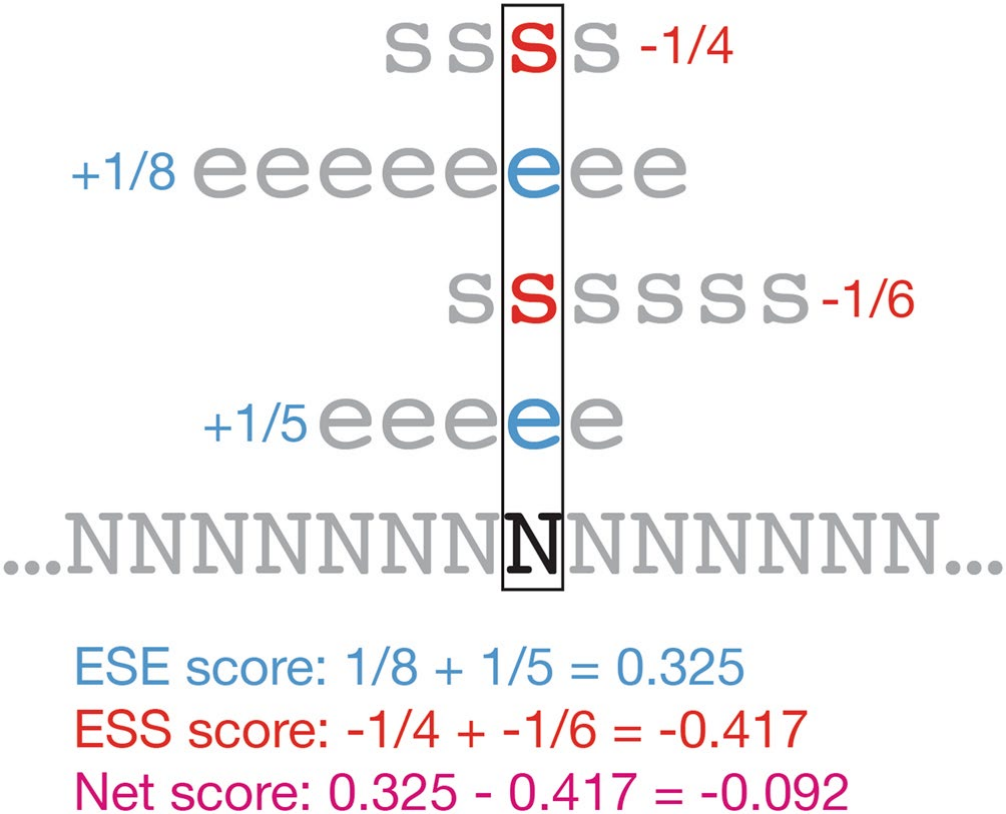
Example of exonic splicing enhancer (ESE) and exonic splicing silencer (ESS) score calculations for an RNA nucleotide. An RNA nucleotide, N, indicated with a rectangular box, is assigned an ESE score as the sum of its contributions to any overlapping enhancer motifs, indicated with ‘e’ characters and blue text, and an ESS score as the sum of its contributions to any overlapping silencer motifs, indicated with ‘s’ characters and red text. The ‘Net score,’ shown in purple text, is determined as the sum of the ESE and ESS scores**.**

For example, a nucleotide that fell within two six nt ESE motifs and one eight nt ESS motif would be assigned an ESE score of 0.333 (2 × 1/6) and an ESS score of − 0.125 (1 × − 1/8). An example diagram of this calculation is provided in Fig. 4.

Predicted centroid normal RNA folding was calculated for the sequence of each cryptically spliced exon with ± 70 nt flanking intron, using RNAfold^50^ with the “avoid isolated base pairs” option. Predicted centroid AO-induced folding was calculated for each exon using the same sequence and settings as for normal folding, but with an additional constraint mask that prohibited binding within the AO target sites.

## Supplementary Information

Supplementary Information 1.

Supplementary Information 2.

## Data Availability

All data generated or analyzed during this study are included in this published article (and its Supplementary Information file).

## Abbreviations

AO: Antisense oligonucleotide
SnRNP: Small nuclear ribonucleoproteins
5′ss: 5′ Splice site
3′ss: 3′ Splice site
Nt: Nucleotide
NMD: Nonsense mediated decay
NC: Negative control
SRSF: Serine/arginine-rich splicing factor
ESE: Exonic splicing enhancer
ESS: Exonic splicing silencer
lncRNA: Long non-coding RNA
2′-OMe PS: 2′-*O*-Methyl modified bases on a phosphorothioate backbone
FBS: Fetal bovine serum
BLAST: Basic local alignment search tool
