# Supplementary material for: Induction of cryptic pre-mRNA splice-switching by antisense oligonucleotides
Source: Sci Rep. 2021 Jul 23;11:15137. doi: 10.1038/s41598-021-94639-x (PMC8302632; doi:10.1038/s41598-021-94639-x)
Supplement: Supplementary file 1 — Supplementary Information 1. [file 41598_2021_94639_MOESM1_ESM.pdf]

Induction of cryptic pre-mRNA splice-switching by antisense oligonucleotides

Kristin A. Ham[1,2¶], Niall P. Keegan[1,2¶], Craig S. McIntosh[1,2], May T. Aung-Htut[1,2], Khine Zaw[1-3], Kane Greer[1,2], Sue Fletcher[1,2] and Steve D. Wilton[1,2*]

1 Centre for Molecular Medicine and Innovative Therapeutics, Health Futures Institute, Murdoch University, Perth, Western Australia, 6150, Australia
2 Perron Institute for Neurological and Translational Science, Centre for Neuromuscular and Neurological Disorders, The University of Western Australia, Perth, Western Australia, 6009, Australia
3 Department of Biochemistry, Faculty of Medicine Siriraj Hospital, Mahidol University, Bangkok, 10700, Thailand
* corresponding author s.wilton@murdoch.edu.au
¶ these authors contributed equally to this work

Supplementary materials

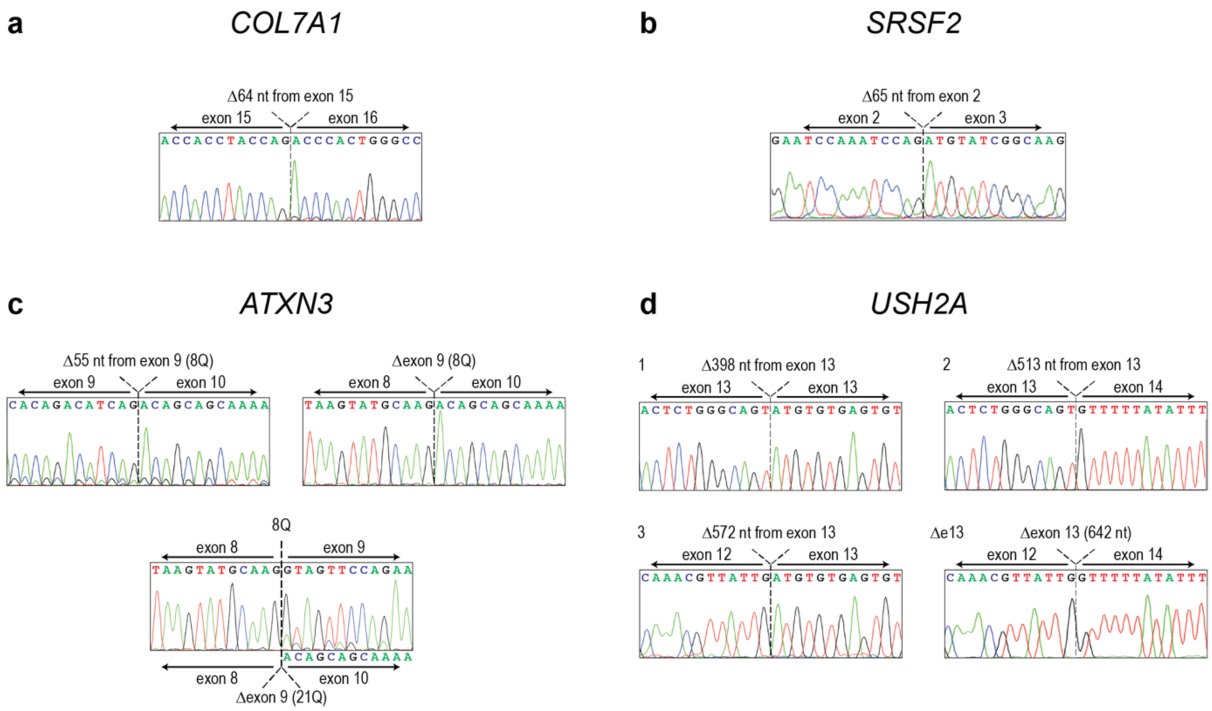

Supplementary Figure S1. **Sanger sequencing confirms transcripts resulting from AO treatment in four different gene transcript targets.** (**a**) *COL7A1* exon 15 new donor site activated. (**b**) *SRSF2* exon 2 new donor site activated. (**c**) *ATXN3* exon 9 new donor site activated in the 8Q transcript and complete exon 9 removal from the 8Q transcript. Sanger sequencing reveals a secondary transcript, complete removal of exon 9 from the 21Q transcript, as well as the full-length 8Q transcript. (**d**) *USH2A* exon 13 new donor (2), new acceptor (3), combination of a new donor and new acceptor (1), and complete exon 13 (Δe13) removal from the full-length transcript.

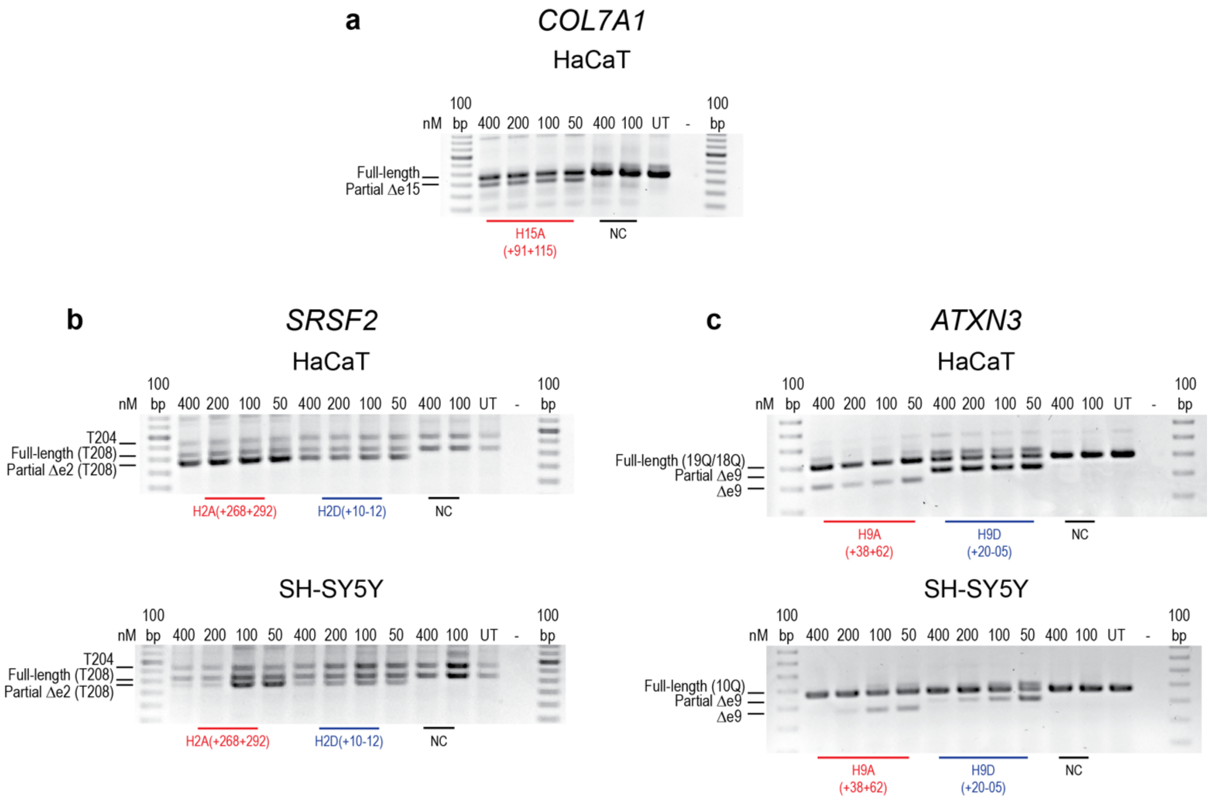

Supplementary Figure S2. **Activation of cryptic splice sites by AO-mediated splice switching in multiple cell lines.** Reverse transcription-PCR analysis after transfection with antisense oligonucleotides (AOs), at various nM concentrations indicated above the gel image for (**a**) *COL7A1* H15A(+91+115) tested in HaCaT, (**b**) *SRSF2* H2A(+268+292) and H2D(+10-12) tested in both HaCaT and SH-SY5Y, and (**c**) *ATXN3* H9A(+38+62) and H9D(+20-05) testing in both HaCaT and SH-SY5Y. Multiple transcript isoforms (noted as T204 and T208) according to Ensembl. NC, negative control sequence synthesized as 2´-OMe PS; UT, untreated; 100 bp, 100 base pair DNA ladder; nM, nanomolar. The gels were cropped for presentation. Full-length gel images are presented in Supplementary Figure S3.

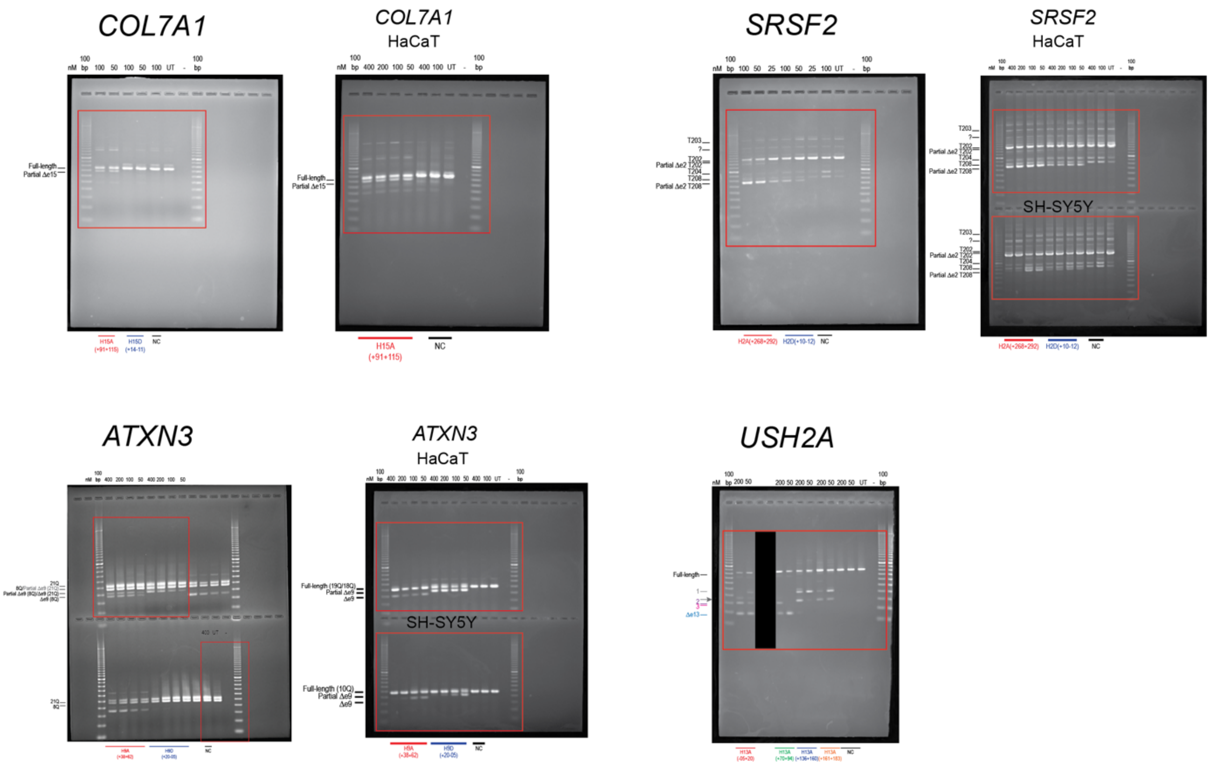

Supplementary Figure S3. **Full gel images for Figure 1 and Supplementary Figure S2.**

~~~ COL7A1 exon 15 ~~~

gggaccaatgagggtatgggtgccagaggggacaggcaggagccatgccagcatttccctctgacctcagGTCCGGAGT
CCAGCCAGACACTGCCCCCAGACTCTACTGCCACAGACATCACAGGGCTGCAGCCTGGAAC
CACCTACCAGGTGGCTGTGTCGGTACTGCGAGGCAGAGAGGAGGGCCCTGCTGCAGTCATC
GTGGCTCGAACGGgtcaggccctgcccccgtcccttggctctctgcctccattgctctttcagacccccatgccttcccttgc

2ry, no AO

.....(((((..(((..(((.((((..((((((.((((((.(((.....(((...........(((...)))............................((((.(((((.(((................((((((.........))))))......
.......)))...)))))))))..........)))..........................)))))))))...)))))))))).))).)))..)))))............................

H15A(+91+115) mask

....
......xxxxxxxxxxxxxxxxxxxxxxxxx...

2ry, H15A(+91+115)

.....(((((..((((.(((.((((..((((((.((((((.(((...((.........((((((((...)))).))))..(((.....)))......
..|||||||||||||||||||||||||............................))...)))))))))...)))))))))).)))))))..)))))............................

~~~ SRSF2 exon 2 ~~~

ggagcgcgcgggcggggccggccccgctgcctggaattaaccccgctgtgcttgctcgtcccgcccgcagCCCTAGGCG
GCGTCGCCGCAGCCGATCCCGGAGTCGGAGTCGTTCCAGGTCTCGCAGCCGATCTCGCTAC
AGCCGCTCGAAGTCTCGGTCCCGCACTCGTTCTCGATCTCGGTCGACCTCCAAGTCCAGATC
CGCACGAAGGTCCAAGTCCAAGTCCTCGTCGGTCTCCAGATCTCGTTCGCGGTCCAGGTCCC
GGTCTCGGTCCAGGAGTCCTCCCCCAGTGTCCAAGAGGGAATCCAAATCCAGGTCGCGATCG
AAGAGTCCCCCCAAGTCTCCTGAAGAGGAAGGAGCGGTGTCCTCTTAAGAAAATGgtaatgtctgg
gaatccgagacacataaccctaattcataaatgggatttggggtaggtctttttgagtc

2ry, no AO

.......((((((((((.(((..(((.((..((......))..)).)))...))).))))))))))........................(((.(((....))).)))..............................(((((.(((.((((.(((
((((...(((....)))...)))..)))).........(((.(((.(((..((.............)).)))))))))..))))).)))...)))))...((.............)).......(((............)))................
......((((((((.(((((((((((((((.(((((((..........))))))).)).....(((..(((((.((...)).)))))....)))............)))))))))))..)).))))))))...

H2A(+268+292) mask

....
....
..........................xxxxxxxxxxxxxxxxxxxxxxxxx...
....

2ry, H2A(+268+292)

.......((((((((((.(((..(((.((..............)).)))...))).))))))))))........................(((.(((....))).)))......................................(((((((
((((......................(((......))).(((.(((.(((..((.............)).)))))))))........((.((((((.((.((..(((....))).)).(((.(((((.............)))))..)))........))
))))))).))...........|||||||||||||||||||||||||............................))))..)))))))).....(((((((.((........)).)))))))...............

~~~ ATXN3 exon 9 ~~~

aacaaacatattgttgcttgaattggtaaatacagtgattggtttttgttgtgttgtgttttgttttcagGTAGTTCCAGAAACATAT
CTCAAGATATGACACAGACATCAGGTACAAATCTTACTTCAGAAGAGCTTCGGAAGAGACGAG
AAGCCTACTTTGAAAAgtaaagtagttggtacaagttaaagtagcatgtttaatatttgctttggctattttgtctatttgtaaat

2ry, no AO

.((((....((((.((((......))))....))))((((.((.((((.(((.(((((((.((((((.((((((((.....))).))))).)))))).)))))))))).)))).)).))))...((((.(((....))).))))((((((((....((((((
.......))))))..........((((((((((..(.......).))))))))))..))))))))..))))....

H9A(+38+62) mask

....xxxxxxxxxxxxxxxxxxxxxxxxx
....

2ry, H9A(+38+62)

..............((((......))))....................((((((((((((((.....((((((((.....))).)))))))))))))))))))...|||||||||||||||||||||||||..(((((...........)))))((((((((.
....))))))))........(((((((((((.(((.....))))))))))))))...................

~~~ HTT exon 12 ~~~

tgcaagcctgtgattccctattgaatgttttctctcgccatttgacaaatgagtgtttctctgtcttcagCCTCAGTGAAGGATGA
GATCAGTGGAGAGCTGGCTGCTTCTTCAGGGGTTTCCACTCCAGGGTCAGCAGGTCATGACA
TCATCACAGAACAGCCACGGTCACAGCACACACTGCAGGCGGACTCAGTGGATCTGGCCAGC
TGTGACTTGACAAGCTCTGCCACTGATGGGGATGAGGAGGATATCTTGAGCCACAGCTCCAG
CCAGGTCAGCGCCGTCCCATCTGACCCTGCCATGGACCTGAATGATGGGACCCAGGCCTCGT

CGCCCATCAGCGACAGCTCCCAGACCACCACCGAAGGGCCTGATTCAGCTGTTACCCCTTCA
GACAGTTCTGAAATTgtaagtgggcagaggggcctgacatcttttttttatttttatttgagacagagtctcactccatagtg

2ry, no AO

.......(((((...................................(((((...((((((((.((......((((((((((..(((((((((((((((((.((((...(((((....)))))...((((........))))........))))
)))))...((((((((...(((((.((.....)))))))..........))))))))......)))))).)))))))....................((((....))))................((((((((....((......))........)))
))))).((((((((.((((((((..(((((((((..(((................)))....))))))).....((((((....))))))...))..)))))).))))))))))............))))))))))).))))))))))...))))))))))
..

H12A(+269+297) mask

....
....
.............................xxxxxxxxxxxxxxxxxxxxxxxxxxxxx...
....

2ry, H12A(+269+297)

.......(((((...................................(((((...((((((((.((......((((((((((.((((((((((((((((((.((((...(((((....)))))...((((........))))........))))
)))))..(((((((((...(((((.((.....)))))))..........)))))))))).....)))))).))))))))....................((((....))))................((((((((....((......))........)))
)))))...............|||||||||||||||||||||||||||||....................((...((((((....))))))...))...............................))))))))))).))))))))))...))))))))))..

~~~ LMNA exon 11 ~~~

ggagcctggttgggcctgagtggtcagtcccagactcgccgtcccgcctgagccttgtctcccttcccagGGCTCCCACTG
CAGCAGCTCGGGGGACCCCGCTGAGTACAACCTGCGCTCGCGCACCGTGCTGTGCGGGAC
CTGCGGGCAGCCTGCCGACAAGGCATCTGCCAGCGGCTCAGGAGCCCAGGTGGGCGGACC
CATCTCCTCTGGCTCTTCTGCCTCCAGTGTCACGGTCACTCGCAGCTACCGCAGTGTGGGGG
GCAGTGGGGGTGGCAGCTTCGGGGACAATCTGGTCACCCGCTCCTACCTCCTGGGCAACTC
CAGCCCCCGAACCCAGgtgagttgtctctgctttgtctccaaatcctgcaggcgggtccctggtcatcgaggggtaggac
gaggtg

2ry, no AO

.((((((((..((((..((.((((.((((...)))).))))))..))))((((((((..........)))))))).((((((...((((((((.((((.(((((.......(((((((..((((...)))).)))))))....((.(((((..((((....
.)))).))))).))(((..(((((((.(((((((....)))))))).....)))))))..)))..)))))....)))).)))).))))...))))))..((((((...((((((((.(((...((((((......))).)))))).))))))))(((......
))))))))).....))))))))..........(((((.((.((((..((((................)))))))).)))))))).....

H11A(+211+245) mask
~~~

....
....xxxxxxxxx
xxxxxxxxxxxxxxxx..

2ry, H11A(+211+245)

.(((((((((.(((((.((.((((.((((...((.(((.(((((...((((((((..........))))))))..(((((...((((((((.((((.(((((.......(((((((..((((...)))).)))))))....((.(((((..((((....
.)))).))))).))(((..((((((((.(((((((....)))))))).....))))))))..)))..)))))....)))).)))).))))...)))))).))))))))))).)))).)))).))..))))....||||||||||||||||||||||||(((((.......)
))))).))).))))))))..........(((((.((.((((..((((.(((....))).....)))))))).)))))))).....

~~~ USH2A exon 13 ~~~

gtttgtgtctcgtctatcttgaatgaaatcattttcccatcctcaccttttaaatatattttatctttagGGCTTAGGTGTGATCATT
GCAATTTTGGATTTAAATTTCTCCGAAGCTTTAATGATGTTGGATGTGAGCCCTGCCAGTGTAAC
CTCCATGGCTCAGTGAACAAATTCTGCAATCCTCACTCTGGGCAGTGTGAGTGCAAAAAAGAA
GCCAAAGGACTTCAGTGTGACACCTGCAGAGAAAACTTTTATGGGTTAGATGTCACCAATTGTA
AGGCCTGTGACTGTGACACAGCTGGATCCCTCCCTGGGACTGTCTGTAATGCTAAGACAGGG
CAGTGCATCTGCAAGCCCAATGTTGAAGGGAGACAGTGCAATAAATGTTTGGAGGGAAACTTC
TACCTACGGCAAAATAATTCTTTCCTCTGTCTGCCTTGCAACTGTGATAAGACTGGGACAATAA
ATGGCTCTCTGCTGTGTAACAAATCAACAGGACAATGTCCTTGCAAATTAGGGGTAACAGGTCT
TCGCTGTAATCAGTGTGAGCCTCACAGGTACAATTTGACCATTGACAATTTTCAACACTGCCAG
ATGTGTGAGTGTGATTCCTTGGGGACATTACCTGGGACCATTTGTGACCCAATCAGTGGCCAG
TGCCTGTGTGTGCCTAATCGTCAAGGAAGAAGGTGTAATCAGTGTCAACCAGgtaagaaagaaatgt
attacattttcagtgcacaatgacattccttttgttaacttaggtaacttctccc

2ry, no AO

....(((((..((((((((.((.(.(((((((...(((((((..........)))))))...)))))))...).)).))))))))................
...................(((...(((((..........))))))))......((((((...)).))))....)))))......((((..........................((.....(((((((.(((.(((((((....((((.....)
)))((((((..........))))))(((..(((....))).)))((..((((((((.((((((...............((((....)))).........................)))))).)))).))))..))........(((.((.....)).))
)...))))))).)))..)).))))).))...((((((...((((((......(((.((((((((....))))).))))))(((((((((.....((.((((((((...........((((..((....))..))))...........................
..............))))))).))))))))))....))))))...))))))....................................((.....))..))))..

H13A(-5+20) mask

....xxxxxxxxxxxxxxxxxxxxxxxxx..
....
....
....
....
....
~~~

2ry, H13A(-5+20)

```
((..(((((.((........................(((((..................................................((((((..........))))))..............)))))((((((.((.............)
).))))))............((((...(((((.((....)).))))))))).....((((((...)).))))..)).))))).)).(((((............................((.....(((((((.(((.(((((((....((((.....)
)))((((((.........))))))(((..(((....))).)))((..((((((((.((((((...............((((....))))..........................)))))).)))).)))).)).........(((.((.....)).))
)...))))))).)))..)).))))).))...((((((...((((((......(((.((((((((....))))).))))))((((((((((....((.(((((((..........((((..((....))..))))............................
..............))))))).)))))))))))....))))))...))))))..................................................................................))))).
```

H13A(+70+94) mask

```
...............................................................................................................................xxxxxxxx
xxxxxxxxxxxxxxxxx...............................................................................................................
................................................................................................................................................
................................................................................................................................................
................................................................................................................................................
...............................
```

2ry, H13A(+70+94)

```
((..(((((.((........................(((((.........................................(((((((...(((((((..........)))))))...)))))))...)))))).....||||||||||||||||||||||
||.............((((...(((((.((....)).))))))))).....((((((...)).))))..)).)))))..)).(((((............................(.....(((((((.(((.(((((((....((((.....))))((((
(((.........))))))(((..(((....))).)))((..((((((((.((((((...............((((....))))..........................)))))).)))).)))).)).........(((.((.....)).)))...))
))))).)))..)).))))).)....((((((...((((((......(((.((((((((....))))).))))))((((((((((....((.(((((((..........((((..((....))..)))).................................
.........))))))).)))))))))))....))))))...))))))..................................................................................))))).
```

H13A(+136+160) mask

```
................................................................................................................................................
..............................................xxxxxxxxxxxxxxxxxxxxxxxxx..................................................
................................................................................................................................................
................................................................................................................................................
................................................................................................................................................
...............................
```

2ry, H13A(+136+160)

```
.............................................................((((((((.((.(.(((((((...(((((((..........)))))))...)))))))...).)).)))))))))..........(((((
(.((((..(((................)))....)))).))).))).|||||||||||||||||||||||......................................................(((((((.(((.(((((((....((((.....))))(
(((((.........))))))(((..(((....))).)))....((((((((.((((((...............((((....))))..........................)))))).)))).)))).............(((.((.....)).)))...
```

)))))))．)))..)).))))))......((((((...((((((......(((.((((((((....))))).))))))(((((((((....((.(((((((..........((((..((....))..))))..............................
..........))))))).))))))))))....))))))...))))))....................................((.....))..

H13A(+161+183) mask

....
....xxxxxxxxxxxxxxxxxxxxxxx.......................................
....
....
....
....

2ry, H13A(+161+183)

...((.((..((((((.....((((....))))..((((..............................((((((((.((.(.(((((((...(((((((..........)))))))...)))))))...).)).))))))))(((.............
)))................(((...(((((..........))))))))......((((((...)).))))...........................)))).))))))..)))).((((((((((((((.(((.(((((((....((((.....)))
)((((((.........))))))(((..(((....))).)))((..((((((((.((((((...............((((....))))..........................)))))).)))).))))..))........(((.((.....)).))).
..))))))).)))..)).))))).......))))))))............(((.((((((((....))))).))))))(((((((((....((.(((((((..........((((..((....))..))))...........................
.............))))))).)))))))))))...(((((............(((((((((((.............................)))))))))))..............................)))))..........

Supplementary Figure S4. **RNA secondary structures.**

ATXN Exon 9 HSF output (JSON)

{"infos": {"HSF Pro version": "4.1.4 - July 2020","Elapsed time": "0,8333s","Sequence": "GTGTTGTGTTTTGTTTTCAGGTAGTTCCAGAAACATATCTCAAGATATGACACAGACATC AGGTACAAATCTTACTTCAGAAGAGCTTCGGAAGAGACGAGAAGCCTACTTTGAAAAGT AAAGTAGTTGGTACAAG","Exon start": "21","Exon end": "117","Genomic start": "-20","Genomic end": "116"},"ESE/ESS signals":{"matrix": [{"name": "IIE (ESS Site)","sequence": "GTGTTG","CV": "NA","position": "1"},{"name": "IIE (ESS Site)","sequence": "TGTTGT","CV": "NA","position": "2"},{"name": "Sironi_motif2 (ESS Site)","sequence": "TGTTGTG","CV": "75.62","position": "2"},{"name": "IIE (ESS Site)","sequence": "GTTGTG","CV": "NA","position": "3"},{"name": "IIE (ESS Site)","sequence": "TTGTGT","CV": "NA","position": "4"},{"name": "PESS (ESS Site)","sequence": "TTGTGTTT","CV": "-40.96781819916","position": "4"},{"name": "IIE (ESS Site)","sequence": "TGTGTT","CV": "NA","position": "5"},{"name": "PESS (ESS Site)","sequence": "TGTGTTTT","CV": "-41.86952247832","position": "5"},{"name": "IIE (ESS Site)","sequence": "GTGTTT","CV": "NA","position": "6"},{"name": "IIE (ESS Site)","sequence": "TGTTTT","CV": "NA","position": "7"},{"name": "IIE (ESS Site)","sequence": "GTTTTG","CV": "NA","position": "8"},{"name": "Fas ESS (ESS Site)","sequence": "GTTTTG","CV": "NA","position": "8"},{"name": "IIE (ESS Site)","sequence": "TTTTGT","CV": "NA","position": "9"},{"name": "PESS (ESS Site)","sequence": "TTTTGTTT","CV": "-73.96754030502","position": "9"},{"name": "IIE (ESS Site)","sequence": "TTTGTT","CV": "NA","position": "10"},{"name": "Fas ESS (ESS Site)","sequence": "TTTGTT","CV": "NA","position": "10"},{"name": "PESS (ESS Site)","sequence": "TTTGTTTT","CV": "-69.08406919979","position": "10"},{"name": "IIE (ESS Site)","sequence": "TTGTTT","CV": "NA","position": "11"},{"name": "Fas ESS (ESS Site)","sequence": "TTGTTT","CV": "NA","position": "11"},{"name": "IIE (ESS Site)","sequence": "TGTTTT","CV": "NA","position": "12"},{"name": "IIE (ESS Site)","sequence": "GTTTTC","CV": "NA","position": "13"},{"name": "ESE_SC35 (ESE Site)","sequence": "GTTTTCAG","CV": "79.73","position": "13"},{"name": "IIE (ESS Site)","sequence": "TTTTCA","CV": "NA","position": "14"},{"name": "ESE_SRp40 (ESE Site)","sequence": "TTTCAGG","CV": "91.76","position": "15"},{"name": "ESS_hnRNPA1 (ESS Site)","sequence": "CAGGTA","CV": "73.46","position": "18"},{"name": "Fas ESS (ESS Site)","sequence": "AGGTAG","CV": "NA","position": "19"},{"name": "IIE (ESS Site)","sequence": "GGTAGT","CV": "NA","position": "20"},{"name": "Fas ESS (ESS Site)","sequence": "GGTAGT","CV": "NA","position": "20"},{"name": "IIE (ESS Site)","sequence": "GTAGTT","CV": "NA","position": "21"},{"name": "Fas ESS (ESS Site)","sequence": "GTAGTT","CV": "NA","position": "21"},{"name": "ESS_hnRNPA1 (ESS Site)","sequence": "TAGTTC","CV": "71.55","position": "22"},{"name": "ESE_SC35 (ESE Site)","sequence": "AGTTCCAG","CV": "77.64","position": "23"},{"name": "Sironi_motif3 (ESS Site)","sequence": "AGTTCCAG","CV": "61.03","position": "23"},{"name": "EIE (ESE Site)","sequence": "CCAGAA","CV": "NA","position": "27"},{"name": "Sironi_motif1 (ESS Site)","sequence": "CCAGAAAC","CV": "60.83","position": "27"},{"name": "ESS_hnRNPA1 (ESS Site)","sequence": "CAGAAA","CV": "65.36","position": "28"},{"name": "RESCUE ESE (ESE Site)","sequence": "CAGAAA","CV": "NA","position": "28"},{"name": "EIE (ESE Site)","sequence": "CAGAAA","CV": "NA","position": "28"},{"name": "RESCUE ESE (ESE Site)","sequence": "AGAAAC","CV": "NA","position": "29"},{"name": "EIE (ESE Site)","sequence": "AGAAAC","CV": "NA","position": "29"},{"name": "RESCUE ESE (ESE Site)","sequence": "GAAACA","CV": "NA","position": "30"},{"name": "EIE (ESE Site)","sequence": "GAAACA","CV": "NA","position": "30"},{"name": "ESE_Tra2 (ESE Site)","sequence": "AACAT","CV": "81.36","position": "32"},{"name": "ESE_SRp55 (ESE Site)","sequence": "AACATA","CV": "74.09","position": "32"},{"name": "Sironi_motif3 (ESS Site)","sequence": "TATCTCAA","CV": "74.29","position": "36"},{"name": "PESE (ESE Site)","sequence": "ATCTCAAG","CV": "28.17473320088","position": "37"},{"name": "ESE_SRp40 (ESE Site)","sequence": "TCTCAAG","CV": "90.2","position": "38"},{"name": "EIE (ESE Site)","sequence": "CTCAAG","CV": "NA","position": "39"},{"name": "ESE_ASF (ESE Site)","sequence": "CTCAAGA","CV": "79.18","position": "39"},{"name": "ESE_ASFB (ESE Site)","sequence": "CTCAAGA","CV": "79.62","position": "39"},{"name": "PESE (ESE Site)","sequence": "CTCAAGAT","CV": "33.63970816967","position": "39"},{"name": "RESCUE ESE (ESE Site)","sequence": "TCAAGA","CV": "NA","position": "40"},{"name": "EIE (ESE Site)","sequence":

"TCAAGA","CV": "NA","position": "40"},{"name": "ESE_9G8 (ESE Site)","sequence": "CAAGAT","CV": "67.39","position": "41"},{"name": "RESCUE ESE (ESE Site)","sequence": "CAAGAT","CV": "NA","position": "41"},{"name": "EIE (ESE Site)","sequence": "CAAGAT","CV": "NA","position": "41"},{"name": "Sironi_motif1 (ESS Site)","sequence": "CAAGATAT","CV": "69.54","position": "41"},{"name": "ESE_Tra2 (ESE Site)","sequence": "AAGAT","CV": "87.22","position": "42"},{"name": "EIE (ESE Site)","sequence": "AAGATA","CV": "NA","position": "42"},{"name": "EIE (ESE Site)","sequence": "AGATAT","CV": "NA","position": "43"},{"name": "RESCUE ESE (ESE Site)","sequence": "GATATG","CV": "NA","position": "44"},{"name": "EIE (ESE Site)","sequence": "GATATG","CV": "NA","position": "44"},{"name": "EIE (ESE Site)","sequence": "ATATGA","CV": "NA","position": "45"},{"name": "ESE_9G8 (ESE Site)","sequence": "TATGAC","CV": "73.83","position": "46"},{"name": "ESS_hnRNPA1 (ESS Site)","sequence": "TATGAC","CV": "70.84","position": "46"},{"name": "EIE (ESE Site)","sequence": "TATGAC","CV": "NA","position": "46"},{"name": "EIE (ESE Site)","sequence": "ATGACA","CV": "NA","position": "47"},{"name": "ESE_SRp40 (ESE Site)","sequence": "TGACACA","CV": "78.89","position": "48"},{"name": "PESE (ESE Site)","sequence": "GACACAGA","CV": "31.54596244696","position": "49"},{"name": "ESE_SRp40 (ESE Site)","sequence": "ACACAGA","CV": "79.37","position": "50"},{"name": "ESE_SRp55 (ESE Site)","sequence": "CACAGA","CV": "75.63","position": "51"},{"name": "ESE_ASFB (ESE Site)","sequence": "CACAGAC","CV": "71.38","position": "51"},{"name": "PESS (ESS Site)","sequence": "CACAGACA","CV": "-29.39041638497","position": "51"},{"name": "ESE_9G8 (ESE Site)","sequence": "ACAGAC","CV": "60.88","position": "52"},{"name": "EIE (ESE Site)","sequence": "ACAGAC","CV": "NA","position": "52"},{"name": "Sironi_motif1 (ESS Site)","sequence": "ACAGACAT","CV": "60.5","position": "52"},{"name": "EIE (ESE Site)","sequence": "CAGACA","CV": "NA","position": "53"},{"name": "ESE_ASF (ESE Site)","sequence": "CAGACAT","CV": "81.92","position": "53"},{"name": "ESE_ASFB (ESE Site)","sequence": "CAGACAT","CV": "79.23","position": "53"},{"name": "RESCUE ESE (ESE Site)","sequence": "AGACAT","CV": "NA","position": "54"},{"name": "EIE (ESE Site)","sequence": "AGACAT","CV": "NA","position": "54"},{"name": "PESE (ESE Site)","sequence": "AGACATCA","CV": "28.36967386841","position": "54"},{"name": "ESE_9G8 (ESE Site)","sequence": "GACATC","CV": "59.27","position": "55"},{"name": "EIE (ESE Site)","sequence": "GACATC","CV": "NA","position": "55"},{"name": "EIE (ESE Site)","sequence": "ACATCA","CV": "NA","position": "56"},{"name": "PESE (ESE Site)","sequence": "ACATCAGG","CV": "24.31449321648","position": "56"},{"name": "RESCUE ESE (ESE Site)","sequence": "CATCAG","CV": "NA","position": "57"},{"name": "Sironi_motif1 (ESS Site)","sequence": "CATCAGGT","CV": "66.69","position": "57"},{"name": "ESE_ASF (ESE Site)","sequence": "ATCAGGT","CV": "73.65","position": "58"},{"name": "ESS_hnRNPA1 (ESS Site)","sequence": "CAGGTA","CV": "73.46","position": "60"},{"name": "EIE (ESE Site)","sequence": "GTACAA","CV": "NA","position": "63"},{"name": "EIE (ESE Site)","sequence": "TACAAA","CV": "NA","position": "64"},{"name": "EIE (ESE Site)","sequence": "ACAAAT","CV": "NA","position": "65"},{"name": "EIE (ESE Site)","sequence": "ATCTTA","CV": "NA","position": "69"},{"name": "EIE (ESE Site)","sequence": "TCTTAC","CV": "NA","position": "70"},{"name": "EIE (ESE Site)","sequence": "CTTACT","CV": "NA","position": "71"},{"name": "IIE (ESS Site)","sequence": "TTACTT","CV": "NA","position": "72"},{"name": "ESE_SRp40 (ESE Site)","sequence": "TTACTTC","CV": "79.37","position": "72"},{"name": "ESE_SRp55 (ESE Site)","sequence": "TACTTC","CV": "77.68","position": "73"},{"name": "RESCUE ESE (ESE Site)","sequence": "ACTTCA","CV": "NA","position": "74"},{"name": "RESCUE ESE (ESE Site)","sequence": "CTTCAG","CV": "NA","position": "75"},{"name": "RESCUE ESE (ESE Site)","sequence": "TTCAGA","CV": "NA","position": "76"},{"name": "PESE (ESE Site)","sequence": "TTCAGAAG","CV": "28.47129187595","position": "76"},{"name": "RESCUE ESE (ESE Site)","sequence": "TCAGAA","CV": "NA","position": "77"},{"name": "EIE (ESE Site)","sequence": "TCAGAA","CV": "NA","position": "77"},{"name": "ESE_SRp40 (ESE Site)","sequence": "TCAGAAG","CV": "86.19","position": "77"},{"name": "Sironi_motif1 (ESS Site)","sequence": "TCAGAAGA","CV": "61.96","position": "77"},{"name": "PESE (ESE Site)","sequence": "TCAGAAGA","CV": "37.08974321752","position": "77"},{"name": "RESCUE ESE (ESE Site)","sequence": "CAGAAG","CV": "NA","position": "78"},{"name": "EIE (ESE Site)","sequence": "CAGAAG","CV": "NA","position": "78"},{"name":

"ESE_ASF (ESE Site)","sequence": "CAGAAGA","CV": "84.25","position": "78"},{"name": "ESE_ASFB (ESE Site)","sequence": "CAGAAGA","CV": "80","position": "78"},{"name": "PESE (ESE Site)","sequence": "CAGAAGAG","CV": "36.94208603105","position": "78"},{"name": "RESCUE ESE (ESE Site)","sequence": "AGAAGA","CV": "NA","position": "79"},{"name": "EIE (ESE Site)","sequence": "AGAAGA","CV": "NA","position": "79"},{"name": "PESE (ESE Site)","sequence": "AGAAGAGC","CV": "38.21376281113","position": "79"},{"name": "ESE_9G8 (ESE Site)","sequence": "GAAGAG","CV": "84.7","position": "80"},{"name": "RESCUE ESE (ESE Site)","sequence": "GAAGAG","CV": "NA","position": "80"},{"name": "EIE (ESE Site)","sequence": "GAAGAG","CV": "NA","position": "80"},{"name": "Sironi_motif1 (ESS Site)","sequence": "GAAGAGCT","CV": "80.78","position": "80"},{"name": "ESE_Tra2 (ESE Site)","sequence": "AAGAG","CV": "89.41","position": "81"},{"name": "ESS_hnRNPA1 (ESS Site)","sequence": "AAGAGC","CV": "71.79","position": "81"},{"name": "EIE (ESE Site)","sequence": "AAGAGC","CV": "NA","position": "81"},{"name": "IIE (ESS Site)","sequence": "GAGCTT","CV": "NA","position": "83"},{"name": "EIE (ESE Site)","sequence": "GCTTCG","CV": "NA","position": "85"},{"name": "ESE_ASFB (ESE Site)","sequence": "CTTCGGA","CV": "73.62","position": "86"},{"name": "ESE_SRp55 (ESE Site)","sequence": "TTCGGA","CV": "74.03","position": "87"},{"name": "ESE_ASF (ESE Site)","sequence": "CGGAAGA","CV": "81.63","position": "89"},{"name": "ESE_ASFB (ESE Site)","sequence": "CGGAAGA","CV": "82.23","position": "89"},{"name": "RESCUE ESE (ESE Site)","sequence": "GGAAGA","CV": "NA","position": "90"},{"name": "EIE (ESE Site)","sequence": "GGAAGA","CV": "NA","position": "90"},{"name": "Sironi_motif2 (ESS Site)","sequence": "GGAAGAG","CV": "62.31","position": "90"},{"name": "PESE (ESE Site)","sequence": "GGAAGAGA","CV": "35.27513594001","position": "90"},{"name": "ESE_9G8 (ESE Site)","sequence": "GAAGAG","CV": "84.7","position": "91"},{"name": "RESCUE ESE (ESE Site)","sequence": "GAAGAG","CV": "NA","position": "91"},{"name": "EIE (ESE Site)","sequence": "GAAGAG","CV": "NA","position": "91"},{"name": "Sironi_motif1 (ESS Site)","sequence": "GAAGAGAC","CV": "70.48","position": "91"},{"name": "ESE_Tra2 (ESE Site)","sequence": "AAGAG","CV": "89.41","position": "92"},{"name": "ESS_hnRNPA1 (ESS Site)","sequence": "AAGAGA","CV": "73.7","position": "92"},{"name": "RESCUE ESE (ESE Site)","sequence": "AAGAGA","CV": "NA","position": "92"},{"name": "EIE (ESE Site)","sequence": "AAGAGA","CV": "NA","position": "92"},{"name": "ESE_9G8 (ESE Site)","sequence": "AGAGAC","CV": "65.17","position": "93"},{"name": "EIE (ESE Site)","sequence": "AGAGAC","CV": "NA","position": "93"},{"name": "Sironi_motif1 (ESS Site)","sequence": "AGAGACGA","CV": "65.2","position": "93"},{"name": "EIE (ESE Site)","sequence": "GAGACG","CV": "NA","position": "94"},{"name": "ESE_ASF (ESE Site)","sequence": "GAGACGA","CV": "89.72","position": "94"},{"name": "ESE_ASFB (ESE Site)","sequence": "GAGACGA","CV": "72.38","position": "94"},{"name": "RESCUE ESE (ESE Site)","sequence": "AGACGA","CV": "NA","position": "95"},{"name": "ESE_9G8 (ESE Site)","sequence": "GACGAG","CV": "84.1","position": "96"},{"name": "EIE (ESE Site)","sequence": "GACGAG","CV": "NA","position": "96"},{"name": "EIE (ESE Site)","sequence": "ACGAGA","CV": "NA","position": "97"},{"name": "PESE (ESE Site)","sequence": "ACGAGAAG","CV": "38.27141547663","position": "97"},{"name": "EIE (ESE Site)","sequence": "CGAGAA","CV": "NA","position": "98"},{"name": "ESE_SRp40 (ESE Site)","sequence": "CGAGAAG","CV": "79.13","position": "98"},{"name": "Sironi_motif1 (ESS Site)","sequence": "CGAGAAGC","CV": "73.99","position": "98"},{"name": "ESE_9G8 (ESE Site)","sequence": "GAGAAG","CV": "59.33","position": "99"},{"name": "ESS_hnRNPA1 (ESS Site)","sequence": "GAGAAG","CV": "66.32","position": "99"},{"name": "RESCUE ESE (ESE Site)","sequence": "GAGAAG","CV": "NA","position": "99"},{"name": "EIE (ESE Site)","sequence": "GAGAAG","CV": "NA","position": "99"},{"name": "RESCUE ESE (ESE Site)","sequence": "AGAAGC","CV": "NA","position": "100"},{"name": "EIE (ESE Site)","sequence": "AGAAGC","CV": "NA","position": "100"},{"name": "PESE (ESE Site)","sequence": "AGAAGCCT","CV": "34.24236516949","position": "100"},{"name": "ESE_9G8 (ESE Site)","sequence": "GAAGCC","CV": "80.41","position": "101"},{"name": "EIE (ESE Site)","sequence": "GAAGCC","CV": "NA","position": "101"},{"name": "EIE (ESE Site)","sequence": "AAGCCT","CV": "NA","position": "102"},{"name": "EIE (ESE Site)","sequence": "AGCCTA","CV": "NA","position": "103"},{"name": "ESE_9G8 (ESE Site)","sequence": "GCCTAC","CV": "61.48","position": "104"},{"name": "EIE (ESE

Site)","sequence": "GCCTAC","CV": "NA","position": "104"},{"name": "EIE (ESE Site)","sequence": "CCTACT","CV": "NA","position": "105"},{"name": "EIE (ESE Site)","sequence": "CTACTT","CV": "NA","position": "106"},{"name": "IIE (ESS Site)","sequence": "TACTTT","CV": "NA","position": "107"},{"name": "EIE (ESE Site)","sequence": "ACTTTG","CV": "NA","position": "108"},{"name": "IIE (ESS Site)","sequence": "ACTTTG","CV": "NA","position": "108"},{"name": "EIE (ESE Site)","sequence": "CTTTGA","CV": "NA","position": "109"},{"name": "IIE (ESS Site)","sequence": "CTTTGA","CV": "NA","position": "109"},{"name": "EIE (ESE Site)","sequence": "TTTGAA","CV": "NA","position": "110"},{"name": "IIE (ESS Site)","sequence": "TTTGAA","CV": "NA","position": "110"},{"name": "EIE (ESE Site)","sequence": "TTGAAA","CV": "NA","position": "111"},{"name": "IIE (ESS Site)","sequence": "TTGAAA","CV": "NA","position": "111"},{"name": "EIE (ESE Site)","sequence": "TGAAAA","CV": "NA","position": "112"},{"name": "ESE_SRp40 (ESE Site)","sequence": "TGAAAAG","CV": "82.18","position": "112"},{"name": "ESE_9G8 (ESE Site)","sequence": "GAAAAG","CV": "64.17","position": "113"},{"name": "RESCUE ESE (ESE Site)","sequence": "GAAAAG","CV": "NA","position": "113"},{"name": "EIE (ESE Site)","sequence": "GAAAAG","CV": "NA","position": "113"},{"name": "ESE_Tra2 (ESE Site)","sequence": "AAAAG","CV": "83.54","position": "114"},{"name": "EIE (ESE Site)","sequence": "AAAAGT","CV": "NA","position": "114"},{"name": "ESS_hnRNPA1 (ESS Site)","sequence": "TAAAGT","CV": "70.84","position": "119"},{"name": "IIE (ESS Site)","sequence": "AGTAGT","CV": "NA","position": "122"},{"name": "IIE (ESS Site)","sequence": "GTAGTT","CV": "NA","position": "123"},{"name": "Fas ESS (ESS Site)","sequence": "GTAGTT","CV": "NA","position": "123"},{"name": "ESS_hnRNPA1 (ESS Site)","sequence": "TAGTTG","CV": "70.6","position": "124"},{"name": "IIE (ESS Site)","sequence": "TAGTTG","CV": "NA","position": "124"},{"name": "IIE (ESS Site)","sequence": "AGTTGG","CV": "NA","position": "125"},{"name": "Sironi_motif2 (ESS Site)","sequence": "AGTTGGT","CV": "73.64","position": "125"},{"name": "IIE (ESS Site)","sequence": "GTTGGT","CV": "NA","position": "126"},{"name": "IIE (ESS Site)","sequence": "TTGGTA","CV": "NA","position": "127"},{"name": "EIE (ESE Site)","sequence": "GTACAA","CV": "NA","position": "130"},{"name": "ESE_SRp40 (ESE Site)","sequence": "GTACAAG","CV": "79.66","position": "130"},{"name": "RESCUE ESE (ESE Site)","sequence": "TACAAG","CV": "NA","position": "131"},{"name": "EIE (ESE Site)","sequence": "TACAAG","CV": "NA","position": "131"}]},"Splice site signals":{"matrix": [{"signal": "Acceptor splice site","matrix": [{"name": "MaxEnt Acceptor site","sequence": "GTGTTGTGTTTTGTTTTCAGGTA","CV": "12.43","position": "1","WT": "true"}]},{"signal": "Acceptor splice site","matrix": [{"name": "HSF Acceptor site (matrix AG)","sequence": "TTTTGTTTTCAGGT","CV": "96.87","position": "9","WT": "true"}]},{"signal": "Acceptor splice site","matrix": [{"name": "HSF Acceptor site (matrix AG)","sequence": "GTTTTCAGGTAGTT","CV": "78.25","position": "13"}]},{"signal": "Donor splice site","matrix": [{"name": "HSF Donor site (matrix GT)","sequence": "CAGGTAGTT","CV": "83.1","position": "18"}]},{"signal": "Donor splice site","matrix": [{"name": "MaxEnt Donor site","sequence": "CAGGTAGTT","CV": "6.3","position": "18"}]},{"signal": "Acceptor splice site","matrix": [{"name": "HSF Acceptor site (matrix AG)","sequence": "AGGTAGTTCCAGAA","CV": "78.39","position": "19"}]},{"signal": "Acceptor splice site","matrix": [{"name": "HSF Acceptor site (matrix AG)","sequence": "ACATATCTCAAGAT","CV": "75.16","position": "33"}]},{"signal": "Acceptor splice site","matrix": [{"name": "HSF Acceptor site (matrix AG)","sequence": "GATATGACACAGAC","CV": "74.85","position": "44"}]},{"signal": "Acceptor splice site","matrix": [{"name": "HSF Acceptor site (matrix AG)","sequence": "CACAGACATCAGGT","CV": "74.81","position": "51"}]},{"signal": "Donor splice site","matrix": [{"name": "HSF Donor site (matrix GT)","sequence": "CAGGTACAA","CV": "80.04","position": "60"}]},{"signal": "Donor splice site","matrix": [{"name": "MaxEnt Donor site","sequence": "CAGGTACAA","CV": "7.09","position": "60"}]},{"signal": "Acceptor splice site","matrix": [{"name": "HSF Acceptor site (matrix AG)","sequence": "ATCTTACTTCAGAA","CV": "86.39","position": "69"}]},{"signal": "Acceptor splice site","matrix": [{"name": "HSF Acceptor site (matrix AG)","sequence": "TTACTTCAGAAGAG","CV": "77.32","position": "72"}]},{"signal": "Acceptor splice site","matrix": [{"name": "HSF Acceptor site (matrix AG)","sequence": "ACTTCAGAAGAGCT","CV": "69.16","position": "74"}]},{"signal": "Acceptor splice site","matrix": [{"name": "HSF Acceptor site (matrix AG)","sequence": "GAGCTTCGGAAGAG","CV": "68.58","position": "83"}]},{"signal":

"Acceptor splice site","matrix": [{"name": "HSF Acceptor site (matrix AG)","sequence": "GCTTCGGAAGAGAC","CV": "68.96","position": "85"}]},{"signal": "Acceptor splice site","matrix": [{"name": "HSF Acceptor site (matrix AG)","sequence": "TACTTTGAAAAGTA","CV": "73","position": "107"}]},{"signal": "Acceptor splice site","matrix": [{"name": "HSF Acceptor site (matrix AG)","sequence": "TGAAAAGTAAAGTA","CV": "66.28","position": "112"}]},{"signal": "Donor splice site","matrix": [{"name": "HSF Donor site (matrix GT)","sequence": "AAAGTAAAG","CV": "77.62","position": "115","WT": "true"}]},{"signal": "Donor splice site","matrix": [{"name": "HSF Donor site (matrix GT)","sequence": "AAAGTAGTT","CV": "72.88","position": "120"}]},{"signal": "Donor splice site","matrix": [{"name": "HSF Donor site (matrix GT)","sequence": "GTAGTTGGT","CV": "69.66","position": "123"}]},{"signal": "Donor splice site","matrix": [{"name": "HSF Donor site (matrix GT)","sequence": "TTGGTACAA","CV": "72.77","position": "127"}]}]},"Branch point signals":{"matrix": [{"name": "BP","sequence": "TTTTCAG","CV": "68.3","position": "14"},{"name": "BP","sequence": "TCCAGAA","CV": "71.6","position": "26"},{"name": "BP","sequence": "ATCTCAA","CV": "85.53","position": "37"},{"name": "BP","sequence": "GACACAG","CV": "73.45","position": "49"},{"name": "BP","sequence": "CACAGAC","CV": "72.87","position": "51"},{"name": "BP","sequence": "ACATCAG","CV": "67.06","position": "56"},{"name": "BP","sequence": "ATCTTAC","CV": "80.32","position": "69"},{"name": "BP","sequence": "ACTTCAG","CV": "68.95","position": "74"},{"name": "BP","sequence": "TTCAGAA","CV": "68.23","position": "76"},{"name": "BP","sequence": "TTCGGAA","CV": "68.23","position": "87"},{"name": "BP","sequence": "AGCCTAC","CV": "70.07","position": "103"}]}}

COL7A1 Exon 15 HSF output (JSON):

{"infos": {"HSF Pro version": "4.1.4 - July 2020","Elapsed time": "1,1667s","Sequence": "GCATTTCCCTCTGACCTCAGGTCCGGAGTCCAGCCAGACACTGCCCCCAGACTCTACTGC CACAGACATCACAGGGCTGCAGCCTGGAACCACCTACCAGGTGGCTGTGTCGGTACTGC GAGGCAGAGAGGAGGGCCCTGCTGCAGTCATCGTGGCTCGAACGGGTCAGGCCCTGCCC CCGTC","Exon start": "21","Exon end": "164","Genomic start": "-20","Genomic end": "162"},"ESE/ESS signals":{"matrix": [{"name": "Sironi_motif3 (ESS Site)","sequence": "TTTCCCTC","CV": "79.66","position": "4"},{"name": "Sironi_motif3 (ESS Site)","sequence": "CCTCTGAC","CV": "61.45","position": "8"},{"name": "PESE (ESE Site)","sequence": "TCTGACCT","CV": "27.66208072203","position": "10"},{"name": "EIE (ESE Site)","sequence": "CTGACC","CV": "NA","position": "11"},{"name": "ESE_ASF (ESE Site)","sequence": "CTGACCT","CV": "75.4","position": "11"},{"name": "ESE_ASFB (ESE Site)","sequence": "CTGACCT","CV": "76.92","position": "11"},{"name": "EIE (ESE Site)","sequence": "TGACCT","CV": "NA","position": "12"},{"name": "IIE (ESS Site)","sequence": "TGACCT","CV": "NA","position": "12"},{"name": "EIE (ESE Site)","sequence": "GACCTC","CV": "NA","position": "13"},{"name": "ESE_SC35 (ESE Site)","sequence": "GACCTCAG","CV": "86.43","position": "13"},{"name": "EIE (ESE Site)","sequence": "ACCTCA","CV": "NA","position": "14"},{"name": "ESE_SRp40 (ESE Site)","sequence": "CCTCAGG","CV": "91.46","position": "15"},{"name": "Sironi_motif1 (ESS Site)","sequence": "CCTCAGGT","CV": "64.47","position": "15"},{"name": "ESE_ASF (ESE Site)","sequence": "CTCAGGT","CV": "88.27","position": "16"},{"name": "ESE_ASFB (ESE Site)","sequence": "CTCAGGT","CV": "90.92","position": "16"},{"name": "ESS_hnRNPA1 (ESS Site)","sequence": "CAGGTC","CV": "71.55","position": "18"},{"name": "Sironi_motif2 (ESS Site)","sequence": "GGTCCGG","CV": "65.36","position": "20"},{"name": "ESE_ASF (ESE Site)","sequence": "GTCCGGA","CV": "75.34","position": "21"},{"name": "ESE_SC35 (ESE Site)","sequence": "GTCCGGAG","CV": "78.25","position": "21"},{"name": "ESE_SRp55 (ESE Site)","sequence": "TCCGGA","CV": "74.03","position": "22"},{"name": "Sironi_motif1 (ESS Site)","sequence": "CCGGAGTC","CV": "61.34","position": "23"},{"name": "ESE_ASFB (ESE Site)","sequence": "CGGAGTC","CV": "72.46","position": "24"},{"name": "ESE_9G8 (ESE Site)","sequence": "GGAGTC","CV": "67.52","position": "25"},{"name": "ESE_SC35 (ESE Site)","sequence": "GAGTCCAG","CV": "75.49","position": "26"},{"name": "Sironi_motif3 (ESS Site)","sequence": "CCAGCCAG","CV": "62.88","position": "30"},{"name": "ESE_ASF (ESE Site)","sequence": "CAGCCAG","CV": "73.94","position": "31"},{"name": "ESE_ASFB (ESE Site)","sequence": "CAGCCAG","CV": "74.54","position": "31"},{"name": "EIE (ESE Site)","sequence": "GCCAGA","CV": "NA","position": "33"},{"name": "ESE_9G8 (ESE Site)","sequence": "CCAGAC","CV": "63.29","position": "34"},{"name": "EIE (ESE Site)","sequence": "CCAGAC","CV": "NA","position": "34"},{"name": "Sironi_motif1 (ESS Site)","sequence": "CCAGACAC","CV": "60.83","position": "34"},{"name": "EIE (ESE Site)","sequence": "CAGACA","CV": "NA","position": "35"},{"name": "ESE_SC35 (ESE Site)","sequence": "AGACACTG","CV": "79.73","position": "36"},{"name": "ESE_ASF (ESE Site)","sequence": "GACACTG","CV": "74.58","position": "37"},{"name": "ESE_SRp40 (ESE Site)","sequence": "ACACTGC","CV": "87.03","position": "38"},{"name": "Sironi_motif3 (ESS Site)","sequence": "ACTGCCCC","CV": "64.67","position": "40"},{"name": "EIE (ESE Site)","sequence": "CTGCCC","CV": "NA","position": "41"},{"name": "EIE (ESE Site)","sequence": "TGCCCC","CV": "NA","position": "42"},{"name": "ESE_SC35 (ESE Site)","sequence": "TGCCCCCA","CV": "80.28","position": "42"},{"name": "Sironi_motif3 (ESS Site)","sequence": "TGCCCCCA","CV": "64.15","position": "42"},{"name": "EIE (ESE Site)","sequence": "GCCCCC","CV": "NA","position": "43"},{"name": "ESE_SC35 (ESE Site)","sequence": "GCCCCCAG","CV": "80.84","position": "43"},{"name": "Sironi_motif3 (ESS Site)","sequence": "GCCCCCAG","CV": "78.49","position": "43"},{"name": "EIE (ESE Site)","sequence": "CCCCCA","CV": "NA","position": "44"},{"name": "ESE_ASFB (ESE Site)","sequence": "CCCCCAG","CV": "72.23","position": "44"},{"name": "EIE (ESE Site)","sequence": "CCCCAG","CV": "NA","position": "45"},{"name": "ESE_ASFB (ESE Site)","sequence": "CCCCAGA","CV": "74.54","position": "45"},{"name": "EIE (ESE Site)","sequence": "CCCAGA","CV": "NA","position": "46"},{"name": "ESE_9G8 (ESE Site)","sequence":

"CCAGAC","CV": "63.29","position": "47"},{"name": "EIE (ESE Site)","sequence": "CCAGAC","CV": "NA","position": "47"},{"name": "Sironi_motif1 (ESS Site)","sequence": "CCAGACTC","CV": "60.04","position": "47"},{"name": "ESE_ASF (ESE Site)","sequence": "CAGACTC","CV": "73.76","position": "48"},{"name": "ESE_ASFB (ESE Site)","sequence": "CAGACTC","CV": "71.85","position": "48"},{"name": "ESE_SC35 (ESE Site)","sequence": "AGACTCTA","CV": "75.18","position": "49"},{"name": "EIE (ESE Site)","sequence": "ACTCTA","CV": "NA","position": "51"},{"name": "EIE (ESE Site)","sequence": "CTCTAC","CV": "NA","position": "52"},{"name": "ESE_SC35 (ESE Site)","sequence": "CTCTACTG","CV": "78.81","position": "52"},{"name": "EIE (ESE Site)","sequence": "TCTACT","CV": "NA","position": "53"},{"name": "EIE (ESE Site)","sequence": "CTACTG","CV": "NA","position": "54"},{"name": "ESE_SRp40 (ESE Site)","sequence": "CTACTGC","CV": "89.31","position": "54"},{"name": "Sironi_motif3 (ESS Site)","sequence": "ACTGCCAC","CV": "75.78","position": "56"},{"name": "PESE (ESE Site)","sequence": "CTGCCACA","CV": "27.84997034413","position": "57"},{"name": "ESE_SC35 (ESE Site)","sequence": "TGCCACAG","CV": "77.82","position": "58"},{"name": "ESE_SRp40 (ESE Site)","sequence": "CCACAGA","CV": "83.5","position": "60"},{"name": "Sironi_motif1 (ESS Site)","sequence": "CCACAGAC","CV": "60.83","position": "60"},{"name": "ESE_SRp55 (ESE Site)","sequence": "CACAGA","CV": "75.63","position": "61"},{"name": "ESE_ASFB (ESE Site)","sequence": "CACAGAC","CV": "71.38","position": "61"},{"name": "PESS (ESS Site)","sequence": "CACAGACA","CV": "-29.39041638497","position": "61"},{"name": "ESE_9G8 (ESE Site)","sequence": "ACAGAC","CV": "60.88","position": "62"},{"name": "EIE (ESE Site)","sequence": "ACAGAC","CV": "NA","position": "62"},{"name": "Sironi_motif1 (ESS Site)","sequence": "ACAGACAT","CV": "60.5","position": "62"},{"name": "EIE (ESE Site)","sequence": "CAGACA","CV": "NA","position": "63"},{"name": "ESE_ASF (ESE Site)","sequence": "CAGACAT","CV": "81.92","position": "63"},{"name": "ESE_ASFB (ESE Site)","sequence": "CAGACAT","CV": "79.23","position": "63"},{"name": "RESCUE ESE (ESE Site)","sequence": "AGACAT","CV": "NA","position": "64"},{"name": "EIE (ESE Site)","sequence": "AGACAT","CV": "NA","position": "64"},{"name": "PESE (ESE Site)","sequence": "AGACATCA","CV": "28.36967386841","position": "64"},{"name": "ESE_9G8 (ESE Site)","sequence": "GACATC","CV": "59.27","position": "65"},{"name": "EIE (ESE Site)","sequence": "GACATC","CV": "NA","position": "65"},{"name": "EIE (ESE Site)","sequence": "ACATCA","CV": "NA","position": "66"},{"name": "Sironi_motif3 (ESS Site)","sequence": "CATCACAG","CV": "64.76","position": "67"},{"name": "ESE_SRp40 (ESE Site)","sequence": "TCACAGG","CV": "99.9","position": "69"},{"name": "Sironi_motif1 (ESS Site)","sequence": "TCACAGGG","CV": "62.01","position": "69"},{"name": "ESE_ASF (ESE Site)","sequence": "CACAGGG","CV": "92.58","position": "70"},{"name": "ESE_ASFB (ESE Site)","sequence": "CACAGGG","CV": "92.69","position": "70"},{"name": "Sironi_motif2 (ESS Site)","sequence": "ACAGGGC","CV": "63.68","position": "71"},{"name": "Sironi_motif1 (ESS Site)","sequence": "ACAGGGCT","CV": "65.6","position": "71"},{"name": "ESE_9G8 (ESE Site)","sequence": "CAGGGC","CV": "59.67","position": "72"},{"name": "ESS_hnRNPA1 (ESS Site)","sequence": "CAGGGC","CV": "83.22","position": "72"},{"name": "IIE (ESS Site)","sequence": "GGGCTG","CV": "NA","position": "74"},{"name": "ESE_SC35 (ESE Site)","sequence": "GGCTGCAG","CV": "86.43","position": "75"},{"name": "ESE_SRp55 (ESE Site)","sequence": "TGCAGC","CV": "87.22","position": "78"},{"name": "ESE_9G8 (ESE Site)","sequence": "GCAGCC","CV": "63.23","position": "79"},{"name": "EIE (ESE Site)","sequence": "CAGCCT","CV": "NA","position": "80"},{"name": "ESE_ASF (ESE Site)","sequence": "CAGCCTG","CV": "76.56","position": "80"},{"name": "ESE_ASFB (ESE Site)","sequence": "CAGCCTG","CV": "76.31","position": "80"},{"name": "EIE (ESE Site)","sequence": "AGCCTG","CV": "NA","position": "81"},{"name": "PESE (ESE Site)","sequence": "AGCCTGGA","CV": "30.49411237707","position": "81"},{"name": "EIE (ESE Site)","sequence": "GCCTGG","CV": "NA","position": "82"},{"name": "PESE (ESE Site)","sequence": "GCCTGGAA","CV": "29.30414477041","position": "82"},{"name": "EIE (ESE Site)","sequence": "CCTGGA","CV": "NA","position": "83"},{"name": "PESE (ESE Site)","sequence": "CCTGGAAC","CV": "44.5464311341","position": "83"},{"name": "EIE (ESE Site)","sequence": "CTGGAA","CV": "NA","position": "84"},{"name": "RESCUE ESE (ESE Site)","sequence": "TGGAAC","CV": "NA","position": "85"},{"name": "EIE (ESE Site)","sequence": "TGGAAC","CV": "NA","position":

"85"},{"name": "EIE (ESE Site)","sequence": "GGAACC","CV": "NA","position": "86"},{"name": "RESCUE ESE (ESE Site)","sequence": "GAACCA","CV": "NA","position": "87"},{"name": "EIE (ESE Site)","sequence": "GAACCA","CV": "NA","position": "87"},{"name": "EIE (ESE Site)","sequence": "ACCACC","CV": "NA","position": "89"},{"name": "Sironi_motif3 (ESS Site)","sequence": "CCACCTAC","CV": "63.3","position": "90"},{"name": "EIE (ESE Site)","sequence": "CACCTA","CV": "NA","position": "91"},{"name": "EIE (ESE Site)","sequence": "ACCTAC","CV": "NA","position": "92"},{"name": "EIE (ESE Site)","sequence": "CCTACC","CV": "NA","position": "93"},{"name": "Sironi_motif3 (ESS Site)","sequence": "CCTACCAG","CV": "78.21","position": "93"},{"name": "EIE (ESE Site)","sequence": "CTACCA","CV": "NA","position": "94"},{"name": "ESE_SRp40 (ESE Site)","sequence": "CTACCAG","CV": "82.06","position": "94"},{"name": "EIE (ESE Site)","sequence": "TACCAG","CV": "NA","position": "95"},{"name": "ESS_hnRNPA1 (ESS Site)","sequence": "CAGGTG","CV": "70.6","position": "98"},{"name": "Sironi_motif1 (ESS Site)","sequence": "CAGGTGGC","CV": "61.49","position": "98"},{"name": "IIE (ESS Site)","sequence": "AGGTGG","CV": "NA","position": "99"},{"name": "Fas ESS (ESS Site)","sequence": "AGGTGG","CV": "NA","position": "99"},{"name": "Sironi_motif2 (ESS Site)","sequence": "AGGTGGC","CV": "63.13","position": "99"},{"name": "ESE_9G8 (ESE Site)","sequence": "GGTGGC","CV": "66.72","position": "100"},{"name": "IIE (ESS Site)","sequence": "GGTGGC","CV": "NA","position": "100"},{"name": "Sironi_motif2 (ESS Site)","sequence": "GGTGGCT","CV": "68.45","position": "100"},{"name": "IIE (ESS Site)","sequence": "GTGGCT","CV": "NA","position": "101"},{"name": "IIE (ESS Site)","sequence": "TGGCTG","CV": "NA","position": "102"},{"name": "IIE (ESS Site)","sequence": "GGCTGT","CV": "NA","position": "103"},{"name": "Sironi_motif2 (ESS Site)","sequence": "GGCTGTG","CV": "60.01","position": "103"},{"name": "EIE (ESE Site)","sequence": "GCTGTG","CV": "NA","position": "104"},{"name": "IIE (ESS Site)","sequence": "GCTGTG","CV": "NA","position": "104"},{"name": "IIE (ESS Site)","sequence": "CTGTGT","CV": "NA","position": "105"},{"name": "ESE_SRp55 (ESE Site)","sequence": "TGTGTC","CV": "86.39","position": "106"},{"name": "IIE (ESS Site)","sequence": "TGTGTC","CV": "NA","position": "106"},{"name": "Sironi_motif2 (ESS Site)","sequence": "TGTGTCG","CV": "66.68","position": "106"},{"name": "RESCUE ESE (ESE Site)","sequence": "GTGTCG","CV": "NA","position": "107"},{"name": "Sironi_motif2 (ESS Site)","sequence": "TGTCGGT","CV": "73.28","position": "108"},{"name": "ESE_SC35 (ESE Site)","sequence": "GTACTGCG","CV": "75.18","position": "113"},{"name": "EIE (ESE Site)","sequence": "ACTGCG","CV": "NA","position": "115"},{"name": "EIE (ESE Site)","sequence": "CTGCGA","CV": "NA","position": "116"},{"name": "EIE (ESE Site)","sequence": "TGCGAG","CV": "NA","position": "117"},{"name": "Sironi_motif2 (ESS Site)","sequence": "TGCGAGG","CV": "71.04","position": "117"},{"name": "Sironi_motif1 (ESS Site)","sequence": "TGCGAGGC","CV": "65.65","position": "117"},{"name": "EIE (ESE Site)","sequence": "CGAGGC","CV": "NA","position": "119"},{"name": "ESE_9G8 (ESE Site)","sequence": "GAGGCA","CV": "63.49","position": "120"},{"name": "ESS_hnRNPA1 (ESS Site)","sequence": "GAGGCA","CV": "78.93","position": "120"},{"name": "ESE_9G8 (ESE Site)","sequence": "GCAGAG","CV": "67.52","position": "123"},{"name": "EIE (ESE Site)","sequence": "GCAGAG","CV": "NA","position": "123"},{"name": "Sironi_motif1 (ESS Site)","sequence": "GCAGAGAG","CV": "66.36","position": "123"},{"name": "ESS_hnRNPA1 (ESS Site)","sequence": "CAGAGA","CV": "73.7","position": "124"},{"name": "EIE (ESE Site)","sequence": "CAGAGA","CV": "NA","position": "124"},{"name": "ESE_ASF (ESE Site)","sequence": "CAGAGAG","CV": "76.15","position": "124"},{"name": "ESE_ASFB (ESE Site)","sequence": "CAGAGAG","CV": "76.08","position": "124"},{"name": "EIE (ESE Site)","sequence": "AGAGAG","CV": "NA","position": "125"},{"name": "ESE_SRp40 (ESE Site)","sequence": "AGAGAGG","CV": "78.41","position": "125"},{"name": "Sironi_motif2 (ESS Site)","sequence": "AGAGAGG","CV": "73.7","position": "125"},{"name": "Sironi_motif1 (ESS Site)","sequence": "AGAGAGGA","CV": "82.1","position": "125"},{"name": "PESE (ESE Site)","sequence": "AGAGAGGA","CV": "43.89814972273","position": "125"},{"name": "ESS_hnRNPA1 (ESS Site)","sequence": "GAGAGG","CV": "74.65","position": "126"},{"name": "ESE_ASF (ESE Site)","sequence": "GAGAGGA","CV": "86.17","position": "126"},{"name": "RESCUE ESE (ESE Site)","sequence": "AGAGGA","CV": "NA","position": "127"},{"name": "EIE (ESE Site)","sequence": "AGAGGA","CV": "NA","position": "127"},{"name": "Sironi_motif2 (ESS Site)","sequence":

"AGAGGAG","CV": "73.7","position": "127"},{"name": "ESE_9G8 (ESE Site)","sequence": "GAGGAG","CV": "79.87","position": "128"},{"name": "ESS_hnRNPA1 (ESS Site)","sequence": "GAGGAG","CV": "77.74","position": "128"},{"name": "RESCUE ESE (ESE Site)","sequence": "GAGGAG","CV": "NA","position": "128"},{"name": "EIE (ESE Site)","sequence": "GAGGAG","CV": "NA","position": "128"},{"name": "Sironi_motif1 (ESS Site)","sequence": "GAGGAGGG","CV": "65.72","position": "128"},{"name": "EIE (ESE Site)","sequence": "AGGAGG","CV": "NA","position": "129"},{"name": "ESE_ASF (ESE Site)","sequence": "AGGAGGG","CV": "73.88","position": "129"},{"name": "Sironi_motif2 (ESS Site)","sequence": "AGGAGGG","CV": "65.97","position": "129"},{"name": "IIE (ESS Site)","sequence": "GGAGGG","CV": "NA","position": "130"},{"name": "Fas ESS (ESS Site)","sequence": "GGAGGG","CV": "NA","position": "130"},{"name": "Sironi_motif2 (ESS Site)","sequence": "GGAGGGC","CV": "84.33","position": "130"},{"name": "ESE_9G8 (ESE Site)","sequence": "GAGGGC","CV": "79","position": "131"},{"name": "ESS_hnRNPA1 (ESS Site)","sequence": "GAGGGC","CV": "87.03","position": "131"},{"name": "ESE_SC35 (ESE Site)","sequence": "GGGCCCTG","CV": "85.38","position": "133"},{"name": "EIE (ESE Site)","sequence": "GGCCCT","CV": "NA","position": "134"},{"name": "PESE (ESE Site)","sequence": "GGCCCTGC","CV": "13.27628899332","position": "134"},{"name": "EIE (ESE Site)","sequence": "GCCCTG","CV": "NA","position": "135"},{"name": "EIE (ESE Site)","sequence": "CCCTGC","CV": "NA","position": "136"},{"name": "EIE (ESE Site)","sequence": "CCTGCT","CV": "NA","position": "137"},{"name": "IIE (ESS Site)","sequence": "CTGCTG","CV": "NA","position": "138"},{"name": "ESE_SRp55 (ESE Site)","sequence": "TGCTGC","CV": "76.4","position": "139"},{"name": "ESE_ASFB (ESE Site)","sequence": "CTGCAGT","CV": "71.77","position": "141"},{"name": "IIE (ESS Site)","sequence": "TGCAGT","CV": "NA","position": "142"},{"name": "ESE_9G8 (ESE Site)","sequence": "GCAGTC","CV": "63.23","position": "143"},{"name": "EIE (ESE Site)","sequence": "TCATCG","CV": "NA","position": "147"},{"name": "EIE (ESE Site)","sequence": "CATCGT","CV": "NA","position": "148"},{"name": "EIE (ESE Site)","sequence": "ATCGTG","CV": "NA","position": "149"},{"name": "EIE (ESE Site)","sequence": "TCGTGG","CV": "NA","position": "150"},{"name": "ESE_SRp55 (ESE Site)","sequence": "CGTGGC","CV": "75.89","position": "151"},{"name": "Sironi_motif2 (ESS Site)","sequence": "CGTGGCT","CV": "61.65","position": "151"},{"name": "IIE (ESS Site)","sequence": "GTGGCT","CV": "NA","position": "152"},{"name": "ESE_ASF (ESE Site)","sequence": "GGCTCGA","CV": "74.29","position": "154"},{"name": "ESE_SC35 (ESE Site)","sequence": "GGCTCGAA","CV": "83.54","position": "154"},{"name": "ESE_ASF (ESE Site)","sequence": "CGAACGG","CV": "80.05","position": "158"},{"name": "ESE_ASFB (ESE Site)","sequence": "CGAACGG","CV": "83","position": "158"},{"name": "EIE (ESE Site)","sequence": "GAACGG","CV": "NA","position": "159"},{"name": "Sironi_motif2 (ESS Site)","sequence": "GAACGGG","CV": "62.31","position": "159"},{"name": "EIE (ESE Site)","sequence": "AACGGG","CV": "NA","position": "160"},{"name": "Sironi_motif2 (ESS Site)","sequence": "GGTCAGG","CV": "65.36","position": "164"},{"name": "ESS_hnRNPA1 (ESS Site)","sequence": "CAGGCC","CV": "73.22","position": "167"},{"name": "EIE (ESE Site)","sequence": "GGCCCT","CV": "NA","position": "169"},{"name": "PESE (ESE Site)","sequence": "GGCCCTGC","CV": "13.27628899332","position": "169"},{"name": "EIE (ESE Site)","sequence": "GCCCTG","CV": "NA","position": "170"},{"name": "EIE (ESE Site)","sequence": "CCCTGC","CV": "NA","position": "171"},{"name": "EIE (ESE Site)","sequence": "CCTGCC","CV": "NA","position": "172"},{"name": "Sironi_motif3 (ESS Site)","sequence": "CCTGCCCC","CV": "67.52","position": "172"},{"name": "EIE (ESE Site)","sequence": "CTGCCC","CV": "NA","position": "173"},{"name": "EIE (ESE Site)","sequence": "TGCCCC","CV": "NA","position": "174"},{"name": "ESE_SC35 (ESE Site)","sequence": "TGCCCCCG","CV": "83.05","position": "174"},{"name": "Sironi_motif3 (ESS Site)","sequence": "TGCCCCCG","CV": "60.44","position": "174"},{"name": "EIE (ESE Site)","sequence": "GCCCCC","CV": "NA","position": "175"},{"name": "Sironi_motif3 (ESS Site)","sequence": "GCCCCCGT","CV": "61.69","position": "175"},{"name": "ESE_ASF (ESE Site)","sequence": "CCCCCGT","CV": "82.56","position": "176"},{"name": "ESE_ASFB (ESE Site)","sequence": "CCCCCGT","CV": "87.46","position": "176"},{"name": "ESE_SRp55 (ESE Site)","sequence": "CCCGTC","CV": "76.27","position": "178"}]},"Splice site signals":{"matrix": [{"signal": "Acceptor splice site","matrix": [{"name": "MaxEnt Acceptor site","sequence":

"GCATTTCCCTCTGACCTCAGGTC","CV": "8.91","position": "1","WT": "true"}]},{"signal": "Acceptor splice site","matrix": [{"name": "HSF Acceptor site (matrix AG)","sequence": "CTCTGACCTCAGGT","CV": "85.15","position": "9","WT": "true"}]},{"signal": "Acceptor splice site","matrix": [{"name": "HSF Acceptor site (matrix AG)","sequence": "TCAGGTCCGGAGTC","CV": "69.75","position": "17"}]},{"signal": "Donor splice site","matrix": [{"name": "HSF Donor site (matrix GT)","sequence": "CAGGTCCGG","CV": "83.46","position": "18"}]},{"signal": "Donor splice site","matrix": [{"name": "MaxEnt Donor site","sequence": "CAGGTCCGG","CV": "4.73","position": "18"}]},{"signal": "Acceptor splice site","matrix": [{"name": "HSF Acceptor site (matrix AG)","sequence": "TCCGGAGTCCAGCC","CV": "76.89","position": "22"}]},{"signal": "Acceptor splice site","matrix": [{"name": "HSF Acceptor site (matrix AG)","sequence": "GAGTCCAGCCAGAC","CV": "73.04","position": "26"}]},{"signal": "Acceptor splice site","matrix": [{"name": "HSF Acceptor site (matrix AG)","sequence": "CACTGCCCCCAGAC","CV": "79.17","position": "39"}]},{"signal": "Acceptor splice site","matrix": [{"name": "MaxEnt Acceptor site","sequence": "CCCAGACTCTACTGCCACAGACA","CV": "3.47","position": "46"}]},{"signal": "Acceptor splice site","matrix": [{"name": "HSF Acceptor site (matrix AG)","sequence": "CTACTGCCACAGAC","CV": "81.1","position": "54"}]},{"signal": "Acceptor splice site","matrix": [{"name": "HSF Acceptor site (matrix AG)","sequence": "CAGACATCACAGGG","CV": "77.26","position": "63"}]},{"signal": "Acceptor splice site","matrix": [{"name": "HSF Acceptor site (matrix AG)","sequence": "ACAGGGCTGCAGCC","CV": "72.99","position": "71"}]},{"signal": "Acceptor splice site","matrix": [{"name": "HSF Acceptor site (matrix AG)","sequence": "ACCACCTACCAGGT","CV": "80.01","position": "89"}]},{"signal": "Donor splice site","matrix": [{"name": "HSF Donor site (matrix GT)","sequence": "CAGGTGGCT","CV": "79.95","position": "98"}]},{"signal": "Donor splice site","matrix": [{"name": "HSF Donor site (matrix GT)","sequence": "TGTGTCGGT","CV": "68.46","position": "106"}]},{"signal": "Donor splice site","matrix": [{"name": "HSF Donor site (matrix GT)","sequence": "TCGGTACTG","CV": "72.3","position": "110"}]},{"signal": "Donor splice site","matrix": [{"name": "MaxEnt Donor site","sequence": "TCGGTACTG","CV": "4.52","position": "110"}]},{"signal": "Acceptor splice site","matrix": [{"name": "HSF Acceptor site (matrix AG)","sequence": "CGGTACTGCGAGGC","CV": "71.21","position": "111"}]},{"signal": "Acceptor splice site","matrix": [{"name": "HSF Acceptor site (matrix AG)","sequence": "ACTGCGAGGCAGAG","CV": "72.83","position": "115"}]},{"signal": "Acceptor splice site","matrix": [{"name": "HSF Acceptor site (matrix AG)","sequence": "TGCGAGGCAGAGAG","CV": "65.79","position": "117"}]},{"signal": "Acceptor splice site","matrix": [{"name": "HSF Acceptor site (matrix AG)","sequence": "GCCCTGCTGCAGTC","CV": "79.44","position": "135"}]},{"signal": "Acceptor splice site","matrix": [{"name": "HSF Acceptor site (matrix AG)","sequence": "CGAACGGGTCAGGC","CV": "70.76","position": "158"}]},{"signal": "Donor splice site","matrix": [{"name": "HSF Donor site (matrix GT)","sequence": "CGGGTCAGG","CV": "85.08","position": "162","WT": "true"}]},{"signal": "Donor splice site","matrix": [{"name": "MaxEnt Donor site","sequence": "CGGGTCAGG","CV": "4.01","position": "162","WT": "true"}]}]},"Branch point signals":{"matrix": [{"name": "BP","sequence": "CCCTCTG","CV": "67.41","position": "7"},{"name": "BP","sequence": "CTCTGAC","CV": "91.46","position": "9"},{"name": "BP","sequence": "ACCTCAG","CV": "92.79","position": "14"},{"name": "BP","sequence": "TCCGGAG","CV": "75.49","position": "22"},{"name": "BP","sequence": "GCCAGAC","CV": "78.11","position": "33"},{"name": "BP","sequence": "CCCCCAG","CV": "88","position": "44"},{"name": "BP","sequence": "CCCAGAC","CV": "79.74","position": "46"},{"name": "BP","sequence": "GCCACAG","CV": "80.32","position": "59"},{"name": "BP","sequence": "CACAGAC","CV": "72.87","position": "61"},{"name": "BP","sequence": "ACATCAC","CV": "69.79","position": "66"},{"name": "BP","sequence": "ATCACAG","CV": "74.33","position": "68"},{"name": "BP","sequence": "CACCTAC","CV": "72.03","position": "91"},{"name": "BP","sequence": "GGCAGAG","CV": "70.81","position": "122"},{"name": "BP","sequence": "CTCGAAC","CV": "69.48","position": "156"},{"name": "BP","sequence": "GGGTCAG","CV": "65.1","position": "163"},{"name": "BP","sequence": "CCCTGCC","CV": "65.2","position": "171"}]}}

HTT Exon 12 HSF Output (JSON):

{"infos": {"HSF Pro version": "4.1.4 - July 2020","Elapsed time": "2,4667s","Sequence": "GAGTGTTTCTCTGTCTTCAGCCTCAGTGAAGGATGAGATCAGTGGAGAGCTGGCTGCTT
CTTCAGGGGTTTCCACTCCAGGGTCAGCAGGTCATGACATCATCACAGAACAGCCACGGT
CACAGCACACACTGCAGGCGGACTCAGTGGATCTGGCCAGCTGTGACTTGACAAGCTCTG
CCACTGATGGGGATGAGGAGGATATCTTGAGCCACAGCTCCAGCCAGGTCAGCGCCGTC
CCATCTGACCCTGCCATGGACCTGAATGATGGGACCCAGGCCTCGTCGCCCATCAGCGAC
AGCTCCCAGACCACCACCGAAGGGCCTGATTCAGCTGTTACCCCTTCAGACAGTTCTGAA
ATTGTAAGTGGGCAGAGGGGCCT","Exon start": "21","Exon end": "361","Genomic start": "-20","Genomic end": "361"},"ESE/ESS signals":{"matrix": [{"name": "ESS_hnRNPA1 (ESS Site)","sequence": "GAGTGT","CV": "72.74","position": "1"},{"name": "EIE (ESE Site)","sequence": "GAGTGT","CV": "NA","position": "1"},{"name": "IIE (ESS Site)","sequence": "GAGTGT","CV": "NA","position": "1"},{"name": "IIE (ESS Site)","sequence": "AGTGTT","CV": "NA","position": "2"},{"name": "Fas ESS (ESS Site)","sequence": "AGTGTT","CV": "NA","position": "2"},{"name": "IIE (ESS Site)","sequence": "GTGTTT","CV": "NA","position": "3"},{"name": "IIE (ESS Site)","sequence": "TGTTTC","CV": "NA","position": "4"},{"name": "Fas ESS (ESS Site)","sequence": "TGTTTC","CV": "NA","position": "4"},{"name": "IIE (ESS Site)","sequence": "GTTTCT","CV": "NA","position": "5"},{"name": "Fas ESS (ESS Site)","sequence": "GTTTCT","CV": "NA","position": "5"},{"name": "PESS (ESS Site)","sequence": "GTTTCTCT","CV": "-34.300017835","position": "5"},{"name": "IIE (ESS Site)","sequence": "TTTCTC","CV": "NA","position": "6"},{"name": "Sironi_motif3 (ESS Site)","sequence": "TTTCTCTG","CV": "62.05","position": "6"},{"name": "IIE (ESS Site)","sequence": "TTCTCT","CV": "NA","position": "7"},{"name": "IIE (ESS Site)","sequence": "TCTCTG","CV": "NA","position": "8"},{"name": "IIE (ESS Site)","sequence": "CTCTGT","CV": "NA","position": "9"},{"name": "IIE (ESS Site)","sequence": "TCTGTC","CV": "NA","position": "10"},{"name": "IIE (ESS Site)","sequence": "CTGTCT","CV": "NA","position": "11"},{"name": "IIE (ESS Site)","sequence": "TGTCTT","CV": "NA","position": "12"},{"name": "IIE (ESS Site)","sequence": "GTCTTC","CV": "NA","position": "13"},{"name": "ESE_SC35 (ESE Site)","sequence": "GTCTTCAG","CV": "83.23","position": "13"},{"name": "PESE (ESE Site)","sequence": "GTCTTCAG","CV": "36.91637045363","position": "13"},{"name": "RESCUE ESE (ESE Site)","sequence": "TCTTCA","CV": "NA","position": "14"},{"name": "RESCUE ESE (ESE Site)","sequence": "CTTCAG","CV": "NA","position": "15"},{"name": "ESE_SRp40 (ESE Site)","sequence": "CTTCAGC","CV": "84.51","position": "15"},{"name": "EIE (ESE Site)","sequence": "CAGCCT","CV": "NA","position": "18"},{"name": "ESE_SC35 (ESE Site)","sequence": "AGCCTCAG","CV": "77.89","position": "19"},{"name": "EIE (ESE Site)","sequence": "CTCAGT","CV": "NA","position": "22"},{"name": "ESE_ASF (ESE Site)","sequence": "CTCAGTG","CV": "73.71","position": "22"},{"name": "ESE_ASFB (ESE Site)","sequence": "CTCAGTG","CV": "77.46","position": "22"},{"name": "EIE (ESE Site)","sequence": "TCAGTG","CV": "NA","position": "23"},{"name": "IIE (ESS Site)","sequence": "TCAGTG","CV": "NA","position": "23"},{"name": "ESS_hnRNPA1 (ESS Site)","sequence": "CAGTGA","CV": "69.89","position": "24"},{"name": "EIE (ESE Site)","sequence": "CAGTGA","CV": "NA","position": "24"},{"name": "PESE (ESE Site)","sequence": "CAGTGAAG","CV": "40.77785474017","position": "24"},{"name": "RESCUE ESE (ESE Site)","sequence": "AGTGAA","CV": "NA","position": "25"},{"name": "EIE (ESE Site)","sequence": "AGTGAA","CV": "NA","position": "25"},{"name": "RESCUE ESE (ESE Site)","sequence": "GTGAAG","CV": "NA","position": "26"},{"name": "EIE (ESE Site)","sequence": "GTGAAG","CV": "NA","position": "26"},{"name": "PESE (ESE Site)","sequence": "GTGAAGGA","CV": "36.92673963807","position": "26"},{"name": "RESCUE ESE (ESE Site)","sequence": "TGAAGG","CV": "NA","position": "27"},{"name": "EIE (ESE Site)","sequence": "TGAAGG","CV": "NA","position": "27"},{"name": "Sironi_motif2 (ESS Site)","sequence": "TGAAGGA","CV": "71.46","position": "27"},{"name": "ESE_9G8 (ESE Site)","sequence": "GAAGGA","CV": "71.75","position": "28"},{"name": "ESS_hnRNPA1 (ESS Site)","sequence": "GAAGGA","CV": "71.79","position": "28"},{"name": "RESCUE ESE (ESE Site)","sequence": "GAAGGA","CV": "NA","position": "28"},{"name": "EIE (ESE Site)","sequence": "GAAGGA","CV": "NA","position": "28"},{"name": "ESE_9G8 (ESE Site)","sequence":

"AAGGAT","CV": "60.14","position": "29"},{"name": "ESS_hnRNPA1 (ESS Site)","sequence": "AAGGAT","CV": "75.84","position": "29"},{"name": "RESCUE ESE (ESE Site)","sequence": "AAGGAT","CV": "NA","position": "29"},{"name": "EIE (ESE Site)","sequence": "AAGGAT","CV": "NA","position": "29"},{"name": "EIE (ESE Site)","sequence": "GGATGA","CV": "NA","position": "31"},{"name": "Sironi_motif2 (ESS Site)","sequence": "GGATGAG","CV": "69.5","position": "31"},{"name": "ESE_9G8 (ESE Site)","sequence": "GATGAG","CV": "80.47","position": "32"},{"name": "RESCUE ESE (ESE Site)","sequence": "GATGAG","CV": "NA","position": "32"},{"name": "EIE (ESE Site)","sequence": "GATGAG","CV": "NA","position": "32"},{"name": "Sironi_motif1 (ESS Site)","sequence": "GATGAGAT","CV": "61.39","position": "32"},{"name": "RESCUE ESE (ESE Site)","sequence": "ATGAGA","CV": "NA","position": "33"},{"name": "EIE (ESE Site)","sequence": "ATGAGA","CV": "NA","position": "33"},{"name": "IIE (ESS Site)","sequence": "TGAGAT","CV": "NA","position": "34"},{"name": "EIE (ESE Site)","sequence": "GAGATC","CV": "NA","position": "35"},{"name": "EIE (ESE Site)","sequence": "AGATCA","CV": "NA","position": "36"},{"name": "ESE_SC35 (ESE Site)","sequence": "GATCAGTG","CV": "85.38","position": "37"},{"name": "EIE (ESE Site)","sequence": "TCAGTG","CV": "NA","position": "39"},{"name": "IIE (ESS Site)","sequence": "TCAGTG","CV": "NA","position": "39"},{"name": "ESE_SRp40 (ESE Site)","sequence": "TCAGTGG","CV": "88.11","position": "39"},{"name": "Sironi_motif2 (ESS Site)","sequence": "TCAGTGG","CV": "63.63","position": "39"},{"name": "Sironi_motif1 (ESS Site)","sequence": "TCAGTGGA","CV": "63.26","position": "39"},{"name": "ESS_hnRNPA1 (ESS Site)","sequence": "CAGTGG","CV": "67.03","position": "40"},{"name": "EIE (ESE Site)","sequence": "CAGTGG","CV": "NA","position": "40"},{"name": "ESE_ASF (ESE Site)","sequence": "CAGTGGA","CV": "81.1","position": "40"},{"name": "ESE_ASFB (ESE Site)","sequence": "CAGTGGA","CV": "80.31","position": "40"},{"name": "PESE (ESE Site)","sequence": "CAGTGGAG","CV": "28.1689264576","position": "40"},{"name": "EIE (ESE Site)","sequence": "AGTGGA","CV": "NA","position": "41"},{"name": "Sironi_motif2 (ESS Site)","sequence": "AGTGGAG","CV": "76.75","position": "41"},{"name": "ESE_9G8 (ESE Site)","sequence": "GTGGAG","CV": "62.69","position": "42"},{"name": "EIE (ESE Site)","sequence": "GTGGAG","CV": "NA","position": "42"},{"name": "IIE (ESS Site)","sequence": "GTGGAG","CV": "NA","position": "42"},{"name": "EIE (ESE Site)","sequence": "TGGAGA","CV": "NA","position": "43"},{"name": "ESE_9G8 (ESE Site)","sequence": "GGAGAG","CV": "71.82","position": "44"},{"name": "EIE (ESE Site)","sequence": "GGAGAG","CV": "NA","position": "44"},{"name": "Sironi_motif2 (ESS Site)","sequence": "GGAGAGC","CV": "67.43","position": "44"},{"name": "Sironi_motif1 (ESS Site)","sequence": "GGAGAGCT","CV": "78.99","position": "44"},{"name": "ESS_hnRNPA1 (ESS Site)","sequence": "GAGAGC","CV": "75.6","position": "45"},{"name": "EIE (ESE Site)","sequence": "GAGAGC","CV": "NA","position": "45"},{"name": "EIE (ESE Site)","sequence": "GAGCTG","CV": "NA","position": "47"},{"name": "PESE (ESE Site)","sequence": "GAGCTGGC","CV": "35.17890990838","position": "47"},{"name": "EIE (ESE Site)","sequence": "AGCTGG","CV": "NA","position": "48"},{"name": "Sironi_motif2 (ESS Site)","sequence": "AGCTGGC","CV": "63.13","position": "48"},{"name": "ESE_9G8 (ESE Site)","sequence": "GCTGGC","CV": "62.42","position": "49"},{"name": "PESE (ESE Site)","sequence": "GCTGGCTG","CV": "42.19677393934","position": "49"},{"name": "PESE (ESE Site)","sequence": "CTGGCTGC","CV": "39.60364829385","position": "50"},{"name": "IIE (ESS Site)","sequence": "TGGCTG","CV": "NA","position": "51"},{"name": "ESE_SC35 (ESE Site)","sequence": "GGCTGCTT","CV": "78.81","position": "52"},{"name": "IIE (ESS Site)","sequence": "CTGCTT","CV": "NA","position": "54"},{"name": "ESE_SRp55 (ESE Site)","sequence": "TGCTTC","CV": "81.58","position": "55"},{"name": "IIE (ESS Site)","sequence": "GCTTCT","CV": "NA","position": "56"},{"name": "IIE (ESS Site)","sequence": "CTTCTT","CV": "NA","position": "57"},{"name": "IIE (ESS Site)","sequence": "TTCTTC","CV": "NA","position": "58"},{"name": "RESCUE ESE (ESE Site)","sequence": "TCTTCA","CV": "NA","position": "59"},{"name": "PESE (ESE Site)","sequence": "TCTTCAGG","CV": "29.59074902841","position": "59"},{"name": "RESCUE ESE (ESE Site)","sequence": "CTTCAG","CV": "NA","position": "60"},{"name": "ESE_SRp40 (ESE Site)","sequence": "CTTCAGG","CV": "89.6","position": "60"},{"name": "Sironi_motif2 (ESS Site)","sequence": "TCAGGGG","CV": "80.53","position": "62"},{"name": "Sironi_motif1 (ESS Site)","sequence": "TCAGGGGT","CV": "71.73","position": "62"},{"name": "ESS_hnRNPA1 (ESS

Site)","sequence": "CAGGGG","CV": "82.27","position": "63"},{"name": "ESE_ASF (ESE Site)","sequence": "CAGGGGT","CV": "76.44","position": "63"},{"name": "ESE_ASFB (ESE Site)","sequence": "CAGGGGT","CV": "78.08","position": "63"},{"name": "IIE (ESS Site)","sequence": "AGGGGT","CV": "NA","position": "64"},{"name": "Fas ESS (ESS Site)","sequence": "AGGGGT","CV": "NA","position": "64"},{"name": "IIE (ESS Site)","sequence": "GGGGTT","CV": "NA","position": "65"},{"name": "Fas ESS (ESS Site)","sequence": "GGGGTT","CV": "NA","position": "65"},{"name": "IIE (ESS Site)","sequence": "GGGTTT","CV": "NA","position": "66"},{"name": "Fas ESS (ESS Site)","sequence": "GGGTTT","CV": "NA","position": "66"},{"name": "IIE (ESS Site)","sequence": "GGTTTC","CV": "NA","position": "67"},{"name": "ESE_SC35 (ESE Site)","sequence": "GGTTTCCA","CV": "83.29","position": "67"},{"name": "Fas ESS (ESS Site)","sequence": "GTTTCC","CV": "NA","position": "68"},{"name": "Sironi_motif3 (ESS Site)","sequence": "GTTTCCAC","CV": "64.49","position": "68"},{"name": "ESE_SRp40 (ESE Site)","sequence": "CCACTCC","CV": "89.31","position": "72"},{"name": "ESE_ASFB (ESE Site)","sequence": "CACTCCA","CV": "72.15","position": "73"},{"name": "ESE_SC35 (ESE Site)","sequence": "CACTCCAG","CV": "77.21","position": "73"},{"name": "Sironi_motif3 (ESS Site)","sequence": "ACTCCAGG","CV": "61.74","position": "74"},{"name": "ESE_SRp40 (ESE Site)","sequence": "CTCCAGG","CV": "81.52","position": "75"},{"name": "ESE_ASFB (ESE Site)","sequence": "CTCCAGG","CV": "73.15","position": "75"},{"name": "Sironi_motif1 (ESS Site)","sequence": "CCAGGGTC","CV": "61.34","position": "77"},{"name": "ESS_hnRNPA1 (ESS Site)","sequence": "CAGGGT","CV": "84.17","position": "78"},{"name": "ESE_SC35 (ESE Site)","sequence": "GGTCAGCA","CV": "80.9","position": "81"},{"name": "PESE (ESE Site)","sequence": "GTCAGCAG","CV": "31.51485489363","position": "82"},{"name": "ESE_ASF (ESE Site)","sequence": "CAGCAGG","CV": "74.23","position": "84"},{"name": "ESE_ASFB (ESE Site)","sequence": "CAGCAGG","CV": "73.54","position": "84"},{"name": "Sironi_motif1 (ESS Site)","sequence": "CAGCAGGT","CV": "66.69","position": "84"},{"name": "ESE_ASF (ESE Site)","sequence": "AGCAGGT","CV": "77.55","position": "85"},{"name": "ESE_ASFB (ESE Site)","sequence": "AGCAGGT","CV": "72.38","position": "85"},{"name": "ESS_hnRNPA1 (ESS Site)","sequence": "CAGGTC","CV": "71.55","position": "87"},{"name": "PESS (ESS Site)","sequence": "AGGTCATG","CV": "-27.29998880128","position": "88"},{"name": "IIE (ESS Site)","sequence": "GTCATG","CV": "NA","position": "90"},{"name": "ESE_9G8 (ESE Site)","sequence": "CATGAC","CV": "76.25","position": "92"},{"name": "EIE (ESE Site)","sequence": "ATGACA","CV": "NA","position": "93"},{"name": "ESE_9G8 (ESE Site)","sequence": "GACATC","CV": "59.27","position": "95"},{"name": "EIE (ESE Site)","sequence": "GACATC","CV": "NA","position": "95"},{"name": "EIE (ESE Site)","sequence": "ACATCA","CV": "NA","position": "96"},{"name": "RESCUE ESE (ESE Site)","sequence": "TCATCA","CV": "NA","position": "99"},{"name": "Sironi_motif3 (ESS Site)","sequence": "CATCACAG","CV": "64.76","position": "100"},{"name": "ESE_SRp40 (ESE Site)","sequence": "TCACAGA","CV": "85.65","position": "102"},{"name": "ESE_SRp55 (ESE Site)","sequence": "CACAGA","CV": "75.63","position": "103"},{"name": "ESE_ASF (ESE Site)","sequence": "CACAGAA","CV": "81.86","position": "103"},{"name": "ESE_ASFB (ESE Site)","sequence": "CACAGAA","CV": "82.31","position": "103"},{"name": "RESCUE ESE (ESE Site)","sequence": "ACAGAA","CV": "NA","position": "104"},{"name": "EIE (ESE Site)","sequence": "ACAGAA","CV": "NA","position": "104"},{"name": "EIE (ESE Site)","sequence": "CAGAAC","CV": "NA","position": "105"},{"name": "RESCUE ESE (ESE Site)","sequence": "AGAACA","CV": "NA","position": "106"},{"name": "EIE (ESE Site)","sequence": "AGAACA","CV": "NA","position": "106"},{"name": "PESE (ESE Site)","sequence": "AGAACAGC","CV": "27.60028038275","position": "106"},{"name": "ESE_9G8 (ESE Site)","sequence": "GAACAG","CV": "62.96","position": "107"},{"name": "EIE (ESE Site)","sequence": "GAACAG","CV": "NA","position": "107"},{"name": "PESE (ESE Site)","sequence": "GAACAGCC","CV": "50.38386720808","position": "107"},{"name": "ESE_Tra2 (ESE Site)","sequence": "AACAG","CV": "83.54","position": "108"},{"name": "EIE (ESE Site)","sequence": "AACAGC","CV": "NA","position": "108"},{"name": "Sironi_motif3 (ESS Site)","sequence": "ACAGCCAC","CV": "60.45","position": "109"},{"name": "ESE_ASF (ESE Site)","sequence": "GCCACGG","CV": "76.91","position": "112"},{"name": "ESE_ASFB (ESE Site)","sequence": "CCACGGT","CV": "72.31","position": "113"},{"name": "ESE_9G8 (ESE

Site)","sequence": "GGTCAC","CV": "60.94","position": "117"},{"name": "ESE_SC35 (ESE Site)","sequence": "GGTCACAG","CV": "86.92","position": "117"},{"name": "Sironi_motif3 (ESS Site)","sequence": "GGTCACAG","CV": "62.3","position": "117"},{"name": "ESE_SRp40 (ESE Site)","sequence": "TCACAGC","CV": "94.81","position": "119"},{"name": "ESE_SRp55 (ESE Site)","sequence": "CACAGC","CV": "78","position": "120"},{"name": "ESE_ASF (ESE Site)","sequence": "CACAGCA","CV": "81.86","position": "120"},{"name": "ESE_ASFB (ESE Site)","sequence": "CACAGCA","CV": "83.31","position": "120"},{"name": "ESE_9G8 (ESE Site)","sequence": "GCACAC","CV": "60.88","position": "124"},{"name": "Sironi_motif3 (ESS Site)","sequence": "GCACACAC","CV": "61.72","position": "124"},{"name": "ESE_ASFB (ESE Site)","sequence": "CACACAC","CV": "73","position": "125"},{"name": "PESS (ESS Site)","sequence": "CACACACT","CV": "-35.88442921787","position": "125"},{"name": "ESE_ASF (ESE Site)","sequence": "CACACTG","CV": "83.78","position": "127"},{"name": "ESE_ASFB (ESE Site)","sequence": "CACACTG","CV": "82.38","position": "127"},{"name": "ESE_SRp40 (ESE Site)","sequence": "ACACTGC","CV": "87.03","position": "128"},{"name": "ESE_SRp40 (ESE Site)","sequence": "CTGCAGG","CV": "80.26","position": "131"},{"name": "Sironi_motif1 (ESS Site)","sequence": "CTGCAGGC","CV": "61.32","position": "131"},{"name": "ESE_SRp55 (ESE Site)","sequence": "TGCAGG","CV": "75.89","position": "132"},{"name": "Sironi_motif2 (ESS Site)","sequence": "TGCAGGC","CV": "62.77","position": "132"},{"name": "ESE_9G8 (ESE Site)","sequence": "GCAGGC","CV": "66.65","position": "133"},{"name": "Sironi_motif2 (ESS Site)","sequence": "GCAGGCG","CV": "60.55","position": "133"},{"name": "ESS_hnRNPA1 (ESS Site)","sequence": "CAGGCG","CV": "72.27","position": "134"},{"name": "ESE_ASF (ESE Site)","sequence": "CAGGCGG","CV": "77.78","position": "134"},{"name": "ESE_ASFB (ESE Site)","sequence": "CAGGCGG","CV": "78.15","position": "134"},{"name": "PESE (ESE Site)","sequence": "GGCGGACT","CV": "44.46886963447","position": "136"},{"name": "ESE_9G8 (ESE Site)","sequence": "GCGGAC","CV": "77.79","position": "137"},{"name": "ESE_ASFB (ESE Site)","sequence": "CGGACTC","CV": "74.08","position": "138"},{"name": "ESE_SC35 (ESE Site)","sequence": "GGACTCAG","CV": "82.43","position": "139"},{"name": "EIE (ESE Site)","sequence": "GACTCA","CV": "NA","position": "140"},{"name": "EIE (ESE Site)","sequence": "CTCAGT","CV": "NA","position": "142"},{"name": "ESE_ASF (ESE Site)","sequence": "CTCAGTG","CV": "73.71","position": "142"},{"name": "ESE_ASFB (ESE Site)","sequence": "CTCAGTG","CV": "77.46","position": "142"},{"name": "EIE (ESE Site)","sequence": "TCAGTG","CV": "NA","position": "143"},{"name": "IIE (ESS Site)","sequence": "TCAGTG","CV": "NA","position": "143"},{"name": "ESE_SRp40 (ESE Site)","sequence": "TCAGTGG","CV": "88.11","position": "143"},{"name": "Sironi_motif2 (ESS Site)","sequence": "TCAGTGG","CV": "63.63","position": "143"},{"name": "Sironi_motif1 (ESS Site)","sequence": "TCAGTGGA","CV": "63.26","position": "143"},{"name": "ESS_hnRNPA1 (ESS Site)","sequence": "CAGTGG","CV": "67.03","position": "144"},{"name": "EIE (ESE Site)","sequence": "CAGTGG","CV": "NA","position": "144"},{"name": "ESE_ASF (ESE Site)","sequence": "CAGTGGA","CV": "81.1","position": "144"},{"name": "ESE_ASFB (ESE Site)","sequence": "CAGTGGA","CV": "80.31","position": "144"},{"name": "EIE (ESE Site)","sequence": "AGTGGA","CV": "NA","position": "145"},{"name": "Sironi_motif2 (ESS Site)","sequence": "AGTGGAT","CV": "64.69","position": "145"},{"name": "ESE_9G8 (ESE Site)","sequence": "GTGGAT","CV": "64.7","position": "146"},{"name": "EIE (ESE Site)","sequence": "GTGGAT","CV": "NA","position": "146"},{"name": "IIE (ESS Site)","sequence": "GTGGAT","CV": "NA","position": "146"},{"name": "RESCUE ESE (ESE Site)","sequence": "TGGATC","CV": "NA","position": "147"},{"name": "EIE (ESE Site)","sequence": "CTGGCC","CV": "NA","position": "152"},{"name": "Sironi_motif1 (ESS Site)","sequence": "CCAGCTGT","CV": "64.47","position": "156"},{"name": "PESE (ESE Site)","sequence": "CCAGCTGT","CV": "27.5173269072","position": "156"},{"name": "ESE_ASF (ESE Site)","sequence": "CAGCTGT","CV": "76.44","position": "157"},{"name": "ESE_ASFB (ESE Site)","sequence": "CAGCTGT","CV": "75.85","position": "157"},{"name": "PESE (ESE Site)","sequence": "CAGCTGTG","CV": "40.95661947996","position": "157"},{"name": "EIE (ESE Site)","sequence": "GCTGTG","CV": "NA","position": "159"},{"name": "IIE (ESS Site)","sequence": "GCTGTG","CV": "NA","position": "159"},{"name": "EIE (ESE Site)","sequence": "CTGTGA","CV": "NA","position": "160"},{"name": "IIE (ESS Site)","sequence": "CTGTGA","CV": "NA","position":

"160"},{"name": "ESE_9G8 (ESE Site)","sequence": "TGTGAC","CV": "60.94","position": "161"},{"name": "EIE (ESE Site)","sequence": "TGTGAC","CV": "NA","position": "161"},{"name": "IIE (ESS Site)","sequence": "TGTGAC","CV": "NA","position": "161"},{"name": "IIE (ESS Site)","sequence": "GTGACT","CV": "NA","position": "162"},{"name": "IIE (ESS Site)","sequence": "TGACTT","CV": "NA","position": "163"},{"name": "ESE_SRp40 (ESE Site)","sequence": "TGACTTG","CV": "81.4","position": "163"},{"name": "IIE (ESS Site)","sequence": "GACTTG","CV": "NA","position": "164"},{"name": "PESE (ESE Site)","sequence": "GACTTGAC","CV": "33.66252037545","position": "164"},{"name": "ESE_9G8 (ESE Site)","sequence": "CTTGAC","CV": "59.06","position": "166"},{"name": "EIE (ESE Site)","sequence": "TGACAA","CV": "NA","position": "168"},{"name": "ESE_SRp40 (ESE Site)","sequence": "TGACAAG","CV": "91.58","position": "168"},{"name": "ESE_9G8 (ESE Site)","sequence": "GACAAG","CV": "63.56","position": "169"},{"name": "EIE (ESE Site)","sequence": "GACAAG","CV": "NA","position": "169"},{"name": "PESE (ESE Site)","sequence": "GACAAGCT","CV": "40.45599525506","position": "169"},{"name": "EIE (ESE Site)","sequence": "ACAAGC","CV": "NA","position": "170"},{"name": "EIE (ESE Site)","sequence": "CAAGCT","CV": "NA","position": "171"},{"name": "PESE (ESE Site)","sequence": "AGCTCTGC","CV": "44.92926142373","position": "173"},{"name": "PESE (ESE Site)","sequence": "CTCTGCCA","CV": "36.23449288467","position": "175"},{"name": "Sironi_motif3 (ESS Site)","sequence": "TCTGCCAC","CV": "84.44","position": "176"},{"name": "ESE_SC35 (ESE Site)","sequence": "TGCCACTG","CV": "84.09","position": "178"},{"name": "ESE_SRp40 (ESE Site)","sequence": "CCACTGA","CV": "82","position": "180"},{"name": "EIE (ESE Site)","sequence": "CTGATG","CV": "NA","position": "183"},{"name": "IIE (ESS Site)","sequence": "CTGATG","CV": "NA","position": "183"},{"name": "ESE_ASF (ESE Site)","sequence": "CTGATGG","CV": "73.47","position": "183"},{"name": "ESE_ASFB (ESE Site)","sequence": "CTGATGG","CV": "74.15","position": "183"},{"name": "IIE (ESS Site)","sequence": "TGATGG","CV": "NA","position": "184"},{"name": "Sironi_motif2 (ESS Site)","sequence": "TGATGGG","CV": "89.48","position": "184"},{"name": "ESE_9G8 (ESE Site)","sequence": "GATGGG","CV": "64.5","position": "185"},{"name": "ESS_hnRNPA1 (ESS Site)","sequence": "GATGGG","CV": "66.79","position": "185"},{"name": "IIE (ESS Site)","sequence": "GATGGG","CV": "NA","position": "185"},{"name": "Fas ESS (ESS Site)","sequence": "GATGGG","CV": "NA","position": "185"},{"name": "Sironi_motif2 (ESS Site)","sequence": "GATGGGG","CV": "80.51","position": "185"},{"name": "IIE (ESS Site)","sequence": "ATGGGG","CV": "NA","position": "186"},{"name": "Fas ESS (ESS Site)","sequence": "ATGGGG","CV": "NA","position": "186"},{"name": "EIE (ESE Site)","sequence": "TGGGGA","CV": "NA","position": "187"},{"name": "IIE (ESS Site)","sequence": "TGGGGA","CV": "NA","position": "187"},{"name": "Fas ESS (ESS Site)","sequence": "TGGGGA","CV": "NA","position": "187"},{"name": "PESS (ESS Site)","sequence": "TGGGGATG","CV": "-28.84914495705","position": "187"},{"name": "ESE_9G8 (ESE Site)","sequence": "GGGGAT","CV": "69","position": "188"},{"name": "IIE (ESS Site)","sequence": "GGGGAT","CV": "NA","position": "188"},{"name": "Fas ESS (ESS Site)","sequence": "GGGGAT","CV": "NA","position": "188"},{"name": "IIE (ESS Site)","sequence": "GGGATG","CV": "NA","position": "189"},{"name": "Fas ESS (ESS Site)","sequence": "GGGATG","CV": "NA","position": "189"},{"name": "EIE (ESE Site)","sequence": "GGATGA","CV": "NA","position": "190"},{"name": "Sironi_motif2 (ESS Site)","sequence": "GGATGAG","CV": "69.5","position": "190"},{"name": "ESE_9G8 (ESE Site)","sequence": "GATGAG","CV": "80.47","position": "191"},{"name": "RESCUE ESE (ESE Site)","sequence": "GATGAG","CV": "NA","position": "191"},{"name": "EIE (ESE Site)","sequence": "GATGAG","CV": "NA","position": "191"},{"name": "Sironi_motif2 (ESS Site)","sequence": "GATGAGG","CV": "63.6","position": "191"},{"name": "Sironi_motif1 (ESS Site)","sequence": "GATGAGGA","CV": "65.66","position": "191"},{"name": "PESE (ESE Site)","sequence": "GATGAGGA","CV": "34.77409694773","position": "191"},{"name": "ESE_ASF (ESE Site)","sequence": "ATGAGGA","CV": "74.23","position": "192"},{"name": "ESE_SRp55 (ESE Site)","sequence": "TGAGGA","CV": "75.05","position": "193"},{"name": "EIE (ESE Site)","sequence": "TGAGGA","CV": "NA","position": "193"},{"name": "Sironi_motif2 (ESS Site)","sequence": "TGAGGAG","CV": "80.53","position": "193"},{"name": "ESE_9G8 (ESE Site)","sequence": "GAGGAG","CV": "79.87","position": "194"},{"name": "ESS_hnRNPA1 (ESS Site)","sequence": "GAGGAG","CV": "77.74","position": "194"},{"name":

"RESCUE ESE (ESE Site)","sequence": "GAGGAG","CV": "NA","position": "194"},{"name": "EIE (ESE Site)","sequence": "GAGGAG","CV": "NA","position": "194"},{"name": "Sironi_motif1 (ESS Site)","sequence": "GAGGAGGA","CV": "65.66","position": "194"},{"name": "PESE (ESE Site)","sequence": "GAGGAGGA","CV": "79.46154899025","position": "194"},{"name": "EIE (ESE Site)","sequence": "AGGAGG","CV": "NA","position": "195"},{"name": "ESE_ASF (ESE Site)","sequence": "AGGAGGA","CV": "78.13","position": "195"},{"name": "ESE_ASFB (ESE Site)","sequence": "AGGAGGA","CV": "71.23","position": "195"},{"name": "RESCUE ESE (ESE Site)","sequence": "GGAGGA","CV": "NA","position": "196"},{"name": "EIE (ESE Site)","sequence": "GGAGGA","CV": "NA","position": "196"},{"name": "Sironi_motif2 (ESS Site)","sequence": "GGAGGAT","CV": "65.4","position": "196"},{"name": "ESE_9G8 (ESE Site)","sequence": "GAGGAT","CV": "81.88","position": "197"},{"name": "ESS_hnRNPA1 (ESS Site)","sequence": "GAGGAT","CV": "79.65","position": "197"},{"name": "RESCUE ESE (ESE Site)","sequence": "GAGGAT","CV": "NA","position": "197"},{"name": "EIE (ESE Site)","sequence": "GGATAT","CV": "NA","position": "199"},{"name": "RESCUE ESE (ESE Site)","sequence": "GATATC","CV": "NA","position": "200"},{"name": "IIE (ESS Site)","sequence": "TATCTT","CV": "NA","position": "202"},{"name": "IIE (ESS Site)","sequence": "ATCTTG","CV": "NA","position": "203"},{"name": "IIE (ESS Site)","sequence": "TCTTGA","CV": "NA","position": "204"},{"name": "IIE (ESS Site)","sequence": "CTTGAG","CV": "NA","position": "205"},{"name": "Sironi_motif1 (ESS Site)","sequence": "CTTGAGCC","CV": "69.27","position": "205"},{"name": "IIE (ESS Site)","sequence": "TTGAGC","CV": "NA","position": "206"},{"name": "ESE_SC35 (ESE Site)","sequence": "AGCCACAG","CV": "79.67","position": "209"},{"name": "ESE_SRp40 (ESE Site)","sequence": "CCACAGC","CV": "92.66","position": "211"},{"name": "Sironi_motif1 (ESS Site)","sequence": "CCACAGCT","CV": "71.12","position": "211"},{"name": "ESE_SRp55 (ESE Site)","sequence": "CACAGC","CV": "78","position": "212"},{"name": "ESE_ASF (ESE Site)","sequence": "CACAGCT","CV": "79.82","position": "212"},{"name": "ESE_ASFB (ESE Site)","sequence": "CACAGCT","CV": "81.54","position": "212"},{"name": "ESE_SC35 (ESE Site)","sequence": "AGCTCCAG","CV": "81.14","position": "215"},{"name": "Sironi_motif3 (ESS Site)","sequence": "GCTCCAGC","CV": "63.44","position": "216"},{"name": "Sironi_motif3 (ESS Site)","sequence": "CCAGCCAG","CV": "62.88","position": "219"},{"name": "ESE_ASF (ESE Site)","sequence": "CAGCCAG","CV": "73.94","position": "220"},{"name": "ESE_ASFB (ESE Site)","sequence": "CAGCCAG","CV": "74.54","position": "220"},{"name": "ESE_ASF (ESE Site)","sequence": "GCCAGGT","CV": "75.57","position": "222"},{"name": "ESS_hnRNPA1 (ESS Site)","sequence": "CAGGTC","CV": "71.55","position": "224"},{"name": "ESE_SC35 (ESE Site)","sequence": "GGTCAGCG","CV": "83.66","position": "226"},{"name": "ESS_hnRNPA1 (ESS Site)","sequence": "CAGCGC","CV": "67.98","position": "229"},{"name": "ESE_ASFB (ESE Site)","sequence": "CGCCGTC","CV": "72.23","position": "232"},{"name": "PESS (ESS Site)","sequence": "CGCCGTCC","CV": "-5.812964798693","position": "232"},{"name": "ESE_9G8 (ESE Site)","sequence": "GCCGTC","CV": "62.62","position": "233"},{"name": "Sironi_motif3 (ESS Site)","sequence": "CGTCCCAT","CV": "77.88","position": "235"},{"name": "PESE (ESE Site)","sequence": "CATCTGAC","CV": "33.49868726125","position": "240"},{"name": "PESE (ESE Site)","sequence": "ATCTGACC","CV": "43.11672798311","position": "241"},{"name": "EIE (ESE Site)","sequence": "CTGACC","CV": "NA","position": "243"},{"name": "EIE (ESE Site)","sequence": "TGACCC","CV": "NA","position": "244"},{"name": "ESE_SC35 (ESE Site)","sequence": "TGACCCTG","CV": "80.77","position": "244"},{"name": "Sironi_motif3 (ESS Site)","sequence": "TGACCCTG","CV": "62.14","position": "244"},{"name": "EIE (ESE Site)","sequence": "GACCCT","CV": "NA","position": "245"},{"name": "EIE (ESE Site)","sequence": "ACCCTG","CV": "NA","position": "246"},{"name": "PESE (ESE Site)","sequence": "ACCCTGCC","CV": "35.45555974931","position": "246"},{"name": "EIE (ESE Site)","sequence": "CCCTGC","CV": "NA","position": "247"},{"name": "EIE (ESE Site)","sequence": "CCTGCC","CV": "NA","position": "248"},{"name": "Sironi_motif3 (ESS Site)","sequence": "CCTGCCAT","CV": "75.03","position": "248"},{"name": "ESE_ASFB (ESE Site)","sequence": "CTGCCAT","CV": "72.77","position": "249"},{"name": "ESS_hnRNPA1 (ESS Site)","sequence": "CATGGA","CV": "65.84","position": "253"},{"name": "EIE (ESE Site)","sequence": "CATGGA","CV": "NA","position": "253"},{"name": "EIE (ESE Site)","sequence": "ATGGAC","CV": "NA","position":

"254"},{"name": "EIE (ESE Site)","sequence": "TGGACC","CV": "NA","position": "255"},{"name": "PESE (ESE Site)","sequence": "TGGACCTG","CV": "26.9627829232","position": "255"},{"name": "EIE (ESE Site)","sequence": "GGACCT","CV": "NA","position": "256"},{"name": "PESE (ESE Site)","sequence": "GGACCTGA","CV": "53.7430682002","position": "256"},{"name": "EIE (ESE Site)","sequence": "GACCTG","CV": "NA","position": "257"},{"name": "ESE_SC35 (ESE Site)","sequence": "GACCTGAA","CV": "78.07","position": "257"},{"name": "PESE (ESE Site)","sequence": "GACCTGAA","CV": "50.52737672077","position": "257"},{"name": "RESCUE ESE (ESE Site)","sequence": "ACCTGA","CV": "NA","position": "258"},{"name": "EIE (ESE Site)","sequence": "ACCTGA","CV": "NA","position": "258"},{"name": "RESCUE ESE (ESE Site)","sequence": "CCTGAA","CV": "NA","position": "259"},{"name": "EIE (ESE Site)","sequence": "CCTGAA","CV": "NA","position": "259"},{"name": "EIE (ESE Site)","sequence": "TGAATG","CV": "NA","position": "261"},{"name": "IIE (ESS Site)","sequence": "TGAATG","CV": "NA","position": "261"},{"name": "EIE (ESE Site)","sequence": "GAATGA","CV": "NA","position": "262"},{"name": "ESE_SC35 (ESE Site)","sequence": "GAATGATG","CV": "76.47","position": "262"},{"name": "ESE_9G8 (ESE Site)","sequence": "AATGAT","CV": "60.74","position": "263"},{"name": "RESCUE ESE (ESE Site)","sequence": "ATGATG","CV": "NA","position": "264"},{"name": "EIE (ESE Site)","sequence": "ATGATG","CV": "NA","position": "264"},{"name": "IIE (ESS Site)","sequence": "TGATGG","CV": "NA","position": "265"},{"name": "Sironi_motif2 (ESS Site)","sequence": "TGATGGG","CV": "89.48","position": "265"},{"name": "ESE_9G8 (ESE Site)","sequence": "GATGGG","CV": "64.5","position": "266"},{"name": "ESS_hnRNPA1 (ESS Site)","sequence": "GATGGG","CV": "66.79","position": "266"},{"name": "IIE (ESS Site)","sequence": "GATGGG","CV": "NA","position": "266"},{"name": "Fas ESS (ESS Site)","sequence": "GATGGG","CV": "NA","position": "266"},{"name": "Sironi_motif2 (ESS Site)","sequence": "GATGGGA","CV": "69.67","position": "266"},{"name": "EIE (ESE Site)","sequence": "ATGGGA","CV": "NA","position": "267"},{"name": "ESE_9G8 (ESE Site)","sequence": "TGGGAC","CV": "60.34","position": "268"},{"name": "EIE (ESE Site)","sequence": "TGGGAC","CV": "NA","position": "268"},{"name": "IIE (ESS Site)","sequence": "TGGGAC","CV": "NA","position": "268"},{"name": "EIE (ESE Site)","sequence": "GGGACC","CV": "NA","position": "269"},{"name": "EIE (ESE Site)","sequence": "GGACCC","CV": "NA","position": "270"},{"name": "ESE_SC35 (ESE Site)","sequence": "GGACCCAG","CV": "87.11","position": "270"},{"name": "Sironi_motif3 (ESS Site)","sequence": "GGACCCAG","CV": "64.16","position": "270"},{"name": "EIE (ESE Site)","sequence": "GACCCA","CV": "NA","position": "271"},{"name": "ESE_SC35 (ESE Site)","sequence": "GACCCAGG","CV": "75.3","position": "271"},{"name": "PESE (ESE Site)","sequence": "GACCCAGG","CV": "44.42822243145","position": "271"},{"name": "EIE (ESE Site)","sequence": "ACCCAG","CV": "NA","position": "272"},{"name": "ESE_SRp40 (ESE Site)","sequence": "ACCCAGG","CV": "79.25","position": "272"},{"name": "ESE_ASF (ESE Site)","sequence": "CCCAGGC","CV": "74","position": "273"},{"name": "ESE_ASFB (ESE Site)","sequence": "CCCAGGC","CV": "79.85","position": "273"},{"name": "ESS_hnRNPA1 (ESS Site)","sequence": "CAGGCC","CV": "73.22","position": "275"},{"name": "ESE_SRp55 (ESE Site)","sequence": "CTCGTC","CV": "76.27","position": "280"},{"name": "RESCUE ESE (ESE Site)","sequence": "TCGTCG","CV": "NA","position": "281"},{"name": "RESCUE ESE (ESE Site)","sequence": "CGTCGC","CV": "NA","position": "282"},{"name": "ESE_9G8 (ESE Site)","sequence": "GTCGCC","CV": "62.62","position": "283"},{"name": "Sironi_motif3 (ESS Site)","sequence": "TCGCCCAT","CV": "82.7","position": "284"},{"name": "ESE_ASF (ESE Site)","sequence": "CGCCCAT","CV": "74.99","position": "285"},{"name": "ESE_ASFB (ESE Site)","sequence": "CGCCCAT","CV": "81.23","position": "285"},{"name": "RESCUE ESE (ESE Site)","sequence": "CATCAG","CV": "NA","position": "289"},{"name": "ESS_hnRNPA1 (ESS Site)","sequence": "CAGCGA","CV": "69.89","position": "292"},{"name": "EIE (ESE Site)","sequence": "CAGCGA","CV": "NA","position": "292"},{"name": "ESE_9G8 (ESE Site)","sequence": "AGCGAC","CV": "64.57","position": "293"},{"name": "EIE (ESE Site)","sequence": "AGCGAC","CV": "NA","position": "293"},{"name": "ESE_SRp40 (ESE Site)","sequence": "CGACAGC","CV": "87.75","position": "295"},{"name": "Sironi_motif1 (ESS Site)","sequence": "CGACAGCT","CV": "71.54","position": "295"},{"name": "ESE_9G8 (ESE Site)","sequence": "GACAGC","CV": "62.69","position": "296"},{"name": "EIE (ESE Site)","sequence": "GACAGC","CV": "NA","position":

"296"},{"name": "ESE_SC35 (ESE Site)","sequence": "AGCTCCCA","CV": "80.71","position": "299"},{"name": "ESE_SC35 (ESE Site)","sequence": "GCTCCCAG","CV": "77.33","position": "300"},{"name": "Sironi_motif3 (ESS Site)","sequence": "GCTCCCAG","CV": "93.81","position": "300"},{"name": "ESE_ASFB (ESE Site)","sequence": "CTCCCAG","CV": "74.15","position": "301"},{"name": "EIE (ESE Site)","sequence": "CCCAGA","CV": "NA","position": "303"},{"name": "ESE_9G8 (ESE Site)","sequence": "CCAGAC","CV": "63.29","position": "304"},{"name": "EIE (ESE Site)","sequence": "CCAGAC","CV": "NA","position": "304"},{"name": "ESE_SRp40 (ESE Site)","sequence": "CCAGACC","CV": "80.5","position": "304"},{"name": "Sironi_motif1 (ESS Site)","sequence": "CCAGACCA","CV": "62.65","position": "304"},{"name": "PESE (ESE Site)","sequence": "CCAGACCA","CV": "49.17938274319","position": "304"},{"name": "EIE (ESE Site)","sequence": "CAGACC","CV": "NA","position": "305"},{"name": "ESE_ASF (ESE Site)","sequence": "CAGACCA","CV": "83.96","position": "305"},{"name": "ESE_ASFB (ESE Site)","sequence": "CAGACCA","CV": "82","position": "305"},{"name": "EIE (ESE Site)","sequence": "AGACCA","CV": "NA","position": "306"},{"name": "ESE_9G8 (ESE Site)","sequence": "GACCAC","CV": "77.45","position": "307"},{"name": "ESE_SC35 (ESE Site)","sequence": "GACCACCA","CV": "87.78","position": "307"},{"name": "EIE (ESE Site)","sequence": "ACCACC","CV": "NA","position": "308"},{"name": "Sironi_motif3 (ESS Site)","sequence": "ACCACCAC","CV": "60.45","position": "308"},{"name": "ESE_SRp40 (ESE Site)","sequence": "CCACCAC","CV": "78.83","position": "309"},{"name": "ESE_SC35 (ESE Site)","sequence": "CACCACCG","CV": "78.07","position": "310"},{"name": "EIE (ESE Site)","sequence": "ACCACC","CV": "NA","position": "311"},{"name": "ESE_ASFB (ESE Site)","sequence": "CCACCGA","CV": "75.69","position": "312"},{"name": "Sironi_motif3 (ESS Site)","sequence": "CCACCGAA","CV": "66.59","position": "312"},{"name": "EIE (ESE Site)","sequence": "CACCGA","CV": "NA","position": "313"},{"name": "ESE_ASF (ESE Site)","sequence": "CACCGAA","CV": "76.09","position": "313"},{"name": "ESE_ASFB (ESE Site)","sequence": "CACCGAA","CV": "79.15","position": "313"},{"name": "PESE (ESE Site)","sequence": "CACCGAAG","CV": "48.96038556775","position": "313"},{"name": "EIE (ESE Site)","sequence": "ACCGAA","CV": "NA","position": "314"},{"name": "PESE (ESE Site)","sequence": "ACCGAAGG","CV": "52.03712997565","position": "314"},{"name": "ESE_ASFB (ESE Site)","sequence": "CCGAAGG","CV": "71.46","position": "315"},{"name": "ESE_ASF (ESE Site)","sequence": "CGAAGGG","CV": "76.5","position": "316"},{"name": "ESE_ASFB (ESE Site)","sequence": "CGAAGGG","CV": "81.38","position": "316"},{"name": "Sironi_motif2 (ESS Site)","sequence": "CGAAGGG","CV": "72.42","position": "316"},{"name": "ESE_9G8 (ESE Site)","sequence": "GAAGGG","CV": "68.73","position": "317"},{"name": "ESS_hnRNPA1 (ESS Site)","sequence": "GAAGGG","CV": "68.94","position": "317"},{"name": "Sironi_motif2 (ESS Site)","sequence": "GAAGGGC","CV": "67.43","position": "317"},{"name": "ESS_hnRNPA1 (ESS Site)","sequence": "AAGGGC","CV": "83.22","position": "318"},{"name": "IIE (ESS Site)","sequence": "GGGCCT","CV": "NA","position": "320"},{"name": "EIE (ESE Site)","sequence": "GCCTGA","CV": "NA","position": "322"},{"name": "EIE (ESE Site)","sequence": "CCTGAT","CV": "NA","position": "323"},{"name": "IIE (ESS Site)","sequence": "CTGATT","CV": "NA","position": "324"},{"name": "RESCUE ESE (ESE Site)","sequence": "GATTCA","CV": "NA","position": "326"},{"name": "PESE (ESE Site)","sequence": "GATTCAGC","CV": "32.82800841148","position": "326"},{"name": "RESCUE ESE (ESE Site)","sequence": "ATTCAG","CV": "NA","position": "327"},{"name": "EIE (ESE Site)","sequence": "ATTCAG","CV": "NA","position": "327"},{"name": "ESE_SRp40 (ESE Site)","sequence": "ATTCAGC","CV": "80.38","position": "327"},{"name": "PESE (ESE Site)","sequence": "TCAGCTGT","CV": "33.27388334253","position": "329"},{"name": "ESE_ASF (ESE Site)","sequence": "CAGCTGT","CV": "76.44","position": "330"},{"name": "ESE_ASFB (ESE Site)","sequence": "CAGCTGT","CV": "75.85","position": "330"},{"name": "PESE (ESE Site)","sequence": "CAGCTGTT","CV": "30.60402573217","position": "330"},{"name": "IIE (ESS Site)","sequence": "GCTGTT","CV": "NA","position": "332"},{"name": "IIE (ESS Site)","sequence": "CTGTTA","CV": "NA","position": "333"},{"name": "EIE (ESE Site)","sequence": "TGTTAC","CV": "NA","position": "334"},{"name": "EIE (ESE Site)","sequence": "GTTACC","CV": "NA","position": "335"},{"name": "EIE (ESE Site)","sequence": "TTACCC","CV": "NA","position": "336"},{"name":

"ESE_SRp40 (ESE Site)","sequence": "TTACCCC","CV": "80.68","position": "336"},{"name": "EIE (ESE Site)","sequence": "TACCCC","CV": "NA","position": "337"},{"name": "EIE (ESE Site)","sequence": "ACCCCT","CV": "NA","position": "338"},{"name": "PESE (ESE Site)","sequence": "CCTTCAGA","CV": "31.32032899348","position": "341"},{"name": "RESCUE ESE (ESE Site)","sequence": "CTTCAG","CV": "NA","position": "342"},{"name": "PESE (ESE Site)","sequence": "CTTCAGAC","CV": "28.7960547327","position": "342"},{"name": "RESCUE ESE (ESE Site)","sequence": "TTCAGA","CV": "NA","position": "343"},{"name": "ESE_9G8 (ESE Site)","sequence": "TCAGAC","CV": "60.88","position": "344"},{"name": "EIE (ESE Site)","sequence": "TCAGAC","CV": "NA","position": "344"},{"name": "EIE (ESE Site)","sequence": "CAGACA","CV": "NA","position": "345"},{"name": "ESE_ASF (ESE Site)","sequence": "CAGACAG","CV": "79.71","position": "345"},{"name": "ESE_ASFB (ESE Site)","sequence": "CAGACAG","CV": "77.69","position": "345"},{"name": "EIE (ESE Site)","sequence": "AGACAG","CV": "NA","position": "346"},{"name": "Sironi_motif1 (ESS Site)","sequence": "AGACAGTT","CV": "60.13","position": "346"},{"name": "EIE (ESE Site)","sequence": "GACAGT","CV": "NA","position": "347"},{"name": "ESE_ASF (ESE Site)","sequence": "GACAGTT","CV": "73.24","position": "347"},{"name": "Fas ESS (ESS Site)","sequence": "AGTTCT","CV": "NA","position": "350"},{"name": "IIE (ESS Site)","sequence": "GTTCTG","CV": "NA","position": "351"},{"name": "Fas ESS (ESS Site)","sequence": "GTTCTG","CV": "NA","position": "351"},{"name": "IIE (ESS Site)","sequence": "TTCTGA","CV": "NA","position": "352"},{"name": "RESCUE ESE (ESE Site)","sequence": "CTGAAA","CV": "NA","position": "354"},{"name": "EIE (ESE Site)","sequence": "CTGAAA","CV": "NA","position": "354"},{"name": "EIE (ESE Site)","sequence": "TGAAAT","CV": "NA","position": "355"},{"name": "EIE (ESE Site)","sequence": "GAAATT","CV": "NA","position": "356"},{"name": "EIE (ESE Site)","sequence": "AAATTG","CV": "NA","position": "357"},{"name": "IIE (ESS Site)","sequence": "AAATTG","CV": "NA","position": "357"},{"name": "IIE (ESS Site)","sequence": "AATTGT","CV": "NA","position": "358"},{"name": "IIE (ESS Site)","sequence": "ATTGTA","CV": "NA","position": "359"},{"name": "IIE (ESS Site)","sequence": "TTGTAA","CV": "NA","position": "360"},{"name": "Fas ESS (ESS Site)","sequence": "GTAAGT","CV": "NA","position": "362"},{"name": "ESS_hnRNPA1 (ESS Site)","sequence": "TAAGTG","CV": "68.7","position": "363"},{"name": "Fas ESS (ESS Site)","sequence": "TAAGTG","CV": "NA","position": "363"},{"name": "Sironi_motif2 (ESS Site)","sequence": "TAAGTGG","CV": "63.63","position": "363"},{"name": "Sironi_motif1 (ESS Site)","sequence": "TAAGTGGG","CV": "65.53","position": "363"},{"name": "ESS_hnRNPA1 (ESS Site)","sequence": "AAGTGG","CV": "67.03","position": "364"},{"name": "EIE (ESE Site)","sequence": "AAGTGG","CV": "NA","position": "364"},{"name": "IIE (ESS Site)","sequence": "AGTGGG","CV": "NA","position": "365"},{"name": "Sironi_motif2 (ESS Site)","sequence": "AGTGGGC","CV": "83.63","position": "365"},{"name": "ESE_9G8 (ESE Site)","sequence": "GTGGGC","CV": "61.82","position": "366"},{"name": "IIE (ESS Site)","sequence": "GTGGGC","CV": "NA","position": "366"},{"name": "IIE (ESS Site)","sequence": "GGGCAG","CV": "NA","position": "368"},{"name": "ESE_9G8 (ESE Site)","sequence": "GCAGAG","CV": "67.52","position": "370"},{"name": "EIE (ESE Site)","sequence": "GCAGAG","CV": "NA","position": "370"},{"name": "Sironi_motif2 (ESS Site)","sequence": "GCAGAGG","CV": "60.55","position": "370"},{"name": "Sironi_motif1 (ESS Site)","sequence": "GCAGAGGG","CV": "79.1","position": "370"},{"name": "ESS_hnRNPA1 (ESS Site)","sequence": "CAGAGG","CV": "70.84","position": "371"},{"name": "RESCUE ESE (ESE Site)","sequence": "CAGAGG","CV": "NA","position": "371"},{"name": "EIE (ESE Site)","sequence": "CAGAGG","CV": "NA","position": "371"},{"name": "ESE_ASF (ESE Site)","sequence": "CAGAGGG","CV": "91.12","position": "371"},{"name": "ESE_ASFB (ESE Site)","sequence": "CAGAGGG","CV": "89.77","position": "371"},{"name": "Sironi_motif2 (ESS Site)","sequence": "AGAGGGG","CV": "90.61","position": "372"},{"name": "Sironi_motif1 (ESS Site)","sequence": "AGAGGGGC","CV": "68.47","position": "372"},{"name": "ESE_9G8 (ESE Site)","sequence": "GAGGGG","CV": "63.9","position": "373"},{"name": "ESS_hnRNPA1 (ESS Site)","sequence": "GAGGGG","CV": "86.08","position": "373"},{"name": "IIE (ESS Site)","sequence": "GAGGGG","CV": "NA","position": "373"},{"name": "ESE_9G8 (ESE Site)","sequence": "GGGGCC","CV": "62.69","position": "375"},{"name": "IIE (ESS Site)","sequence": "GGGGCC","CV": "NA","position": "375"},{"name": "IIE (ESS Site)","sequence": "GGGCCT","CV": "NA","position": "376"}]},"Splice site

signals":{"matrix": [{"signal": "Acceptor splice site","matrix": [{"name": "MaxEnt Acceptor site","sequence": "GAGTGTTTCTCTGTCTTCAGCCT","CV": "6.43","position": "1","WT": "true"}]},{"signal": "Acceptor splice site","matrix": [{"name": "HSF Acceptor site (matrix AG)","sequence": "CTCTGTCTTCAGCC","CV": "86.58","position": "9","WT": "true"}]},{"signal": "Acceptor splice site","matrix": [{"name": "HSF Acceptor site (matrix AG)","sequence": "CTTCAGCCTCAGTG","CV": "81.11","position": "15"}]},{"signal": "Acceptor splice site","matrix": [{"name": "HSF Acceptor site (matrix AG)","sequence": "GCCTCAGTGAAGGA","CV": "74.2","position": "20"}]},{"signal": "Donor splice site","matrix": [{"name": "HSF Donor site (matrix GT)","sequence": "TCAGTGAAG","CV": "67.24","position": "23"}]},{"signal": "Acceptor splice site","matrix": [{"name": "HSF Acceptor site (matrix AG)","sequence": "GGATGAGATCAGTG","CV": "68.08","position": "31"}]},{"signal": "Donor splice site","matrix": [{"name": "HSF Donor site (matrix GT)","sequence": "GAGATCAGT","CV": "65.53","position": "35"}]},{"signal": "Acceptor splice site","matrix": [{"name": "HSF Acceptor site (matrix AG)","sequence": "AGATCAGTGGAGAG","CV": "67.7","position": "36"}]},{"signal": "Acceptor splice site","matrix": [{"name": "HSF Acceptor site (matrix AG)","sequence": "ATCAGTGGAGAGCT","CV": "67.06","position": "38"}]},{"signal": "Acceptor splice site","matrix": [{"name": "MaxEnt Acceptor site","sequence": "AGAGCTGGCTGCTTCTTCAGGGG","CV": "4.08","position": "46"}]},{"signal": "Acceptor splice site","matrix": [{"name": "HSF Acceptor site (matrix AG)","sequence": "CTGCTTCTTCAGGG","CV": "89.14","position": "54"}]},{"signal": "Acceptor splice site","matrix": [{"name": "MaxEnt Acceptor site","sequence": "TTCAGGGGTTTCCACTCCAGGGT","CV": "5.96","position": "61"}]},{"signal": "Donor splice site","matrix": [{"name": "HSF Donor site (matrix GT)","sequence": "GGGGTTTCC","CV": "66.46","position": "65"}]},{"signal": "Acceptor splice site","matrix": [{"name": "HSF Acceptor site (matrix AG)","sequence": "TTTCCACTCCAGGG","CV": "91.15","position": "69"}]},{"signal": "Acceptor splice site","matrix": [{"name": "HSF Acceptor site (matrix AG)","sequence": "CTCCAGGGTCAGCA","CV": "74.54","position": "75"}]},{"signal": "Acceptor splice site","matrix": [{"name": "HSF Acceptor site (matrix AG)","sequence": "CAGGGTCAGCAGGT","CV": "75.83","position": "78"}]},{"signal": "Donor splice site","matrix": [{"name": "HSF Donor site (matrix GT)","sequence": "AGGGTCAGC","CV": "84.61","position": "79"}]},{"signal": "Donor splice site","matrix": [{"name": "MaxEnt Donor site","sequence": "AGGGTCAGC","CV": "3.64","position": "79"}]},{"signal": "Donor splice site","matrix": [{"name": "HSF Donor site (matrix GT)","sequence": "CAGGTCATG","CV": "81.48","position": "87"}]},{"signal": "Donor splice site","matrix": [{"name": "MaxEnt Donor site","sequence": "CAGGTCATG","CV": "3.35","position": "87"}]},{"signal": "Acceptor splice site","matrix": [{"name": "HSF Acceptor site (matrix AG)","sequence": "ACATCATCACAGAA","CV": "79.09","position": "96"}]},{"signal": "Acceptor splice site","matrix": [{"name": "HSF Acceptor site (matrix AG)","sequence": "ATCACAGAACAGCC","CV": "72.71","position": "101"}]},{"signal": "Acceptor splice site","matrix": [{"name": "HSF Acceptor site (matrix AG)","sequence": "CCACGGTCACAGCA","CV": "75.94","position": "113"}]},{"signal": "Donor splice site","matrix": [{"name": "HSF Donor site (matrix GT)","sequence": "ACGGTCACA","CV": "74.78","position": "115"}]},{"signal": "Acceptor splice site","matrix": [{"name": "HSF Acceptor site (matrix AG)","sequence": "CACACACTGCAGGC","CV": "79.74","position": "125"}]},{"signal": "Acceptor splice site","matrix": [{"name": "HSF Acceptor site (matrix AG)","sequence": "AGGCGGACTCAGTG","CV": "68.4","position": "135"}]},{"signal": "Acceptor splice site","matrix": [{"name": "HSF Acceptor site (matrix AG)","sequence": "GGATCTGGCCAGCT","CV": "73.99","position": "148"}]},{"signal": "Donor splice site","matrix": [{"name": "HSF Donor site (matrix GT)","sequence": "GCTGTGACT","CV": "69.35","position": "159"}]},{"signal": "Acceptor splice site","matrix": [{"name": "HSF Acceptor site (matrix AG)","sequence": "TGACTTGACAAGCT","CV": "70.65","position": "163"}]},{"signal": "Acceptor splice site","matrix": [{"name": "HSF Acceptor site (matrix AG)","sequence": "GGATATCTTGAGCC","CV": "70.91","position": "199"}]},{"signal": "Acceptor splice site","matrix": [{"name": "HSF Acceptor site (matrix AG)","sequence": "CTTGAGCCACAGCT","CV": "80.7","position": "205"}]},{"signal": "Acceptor splice site","matrix": [{"name": "HSF Acceptor site (matrix AG)","sequence": "CCACAGCTCCAGCC","CV": "76.76","position": "211"}]},{"signal": "Acceptor splice site","matrix": [{"name": "HSF Acceptor site (matrix AG)","sequence": "AGCTCCAGCCAGGT","CV": "77.8","position": "215"}]},{"signal":

"Acceptor splice site","matrix": [{"name": "HSF Acceptor site (matrix AG)","sequence": "CAGCCAGGTCAGCG","CV": "69.8","position": "220"}]},{"signal": "Donor splice site","matrix": [{"name": "HSF Donor site (matrix GT)","sequence": "CAGGTCAGC","CV": "90.85","position": "224"}]},{"signal": "Donor splice site","matrix": [{"name": "MaxEnt Donor site","sequence": "CAGGTCAGC","CV": "7.16","position": "224"}]},{"signal": "Acceptor splice site","matrix": [{"name": "HSF Acceptor site (matrix AG)","sequence": "GATGGGACCCAGGC","CV": "73.92","position": "266"}]},{"signal": "Acceptor splice site","matrix": [{"name": "HSF Acceptor site (matrix AG)","sequence": "GTCGCCCATCAGCG","CV": "77.59","position": "283"}]},{"signal": "Acceptor splice site","matrix": [{"name": "HSF Acceptor site (matrix AG)","sequence": "CATCAGCGACAGCT","CV": "75.29","position": "289"}]},{"signal": "Acceptor splice site","matrix": [{"name": "HSF Acceptor site (matrix AG)","sequence": "GACAGCTCCCAGAC","CV": "75.51","position": "296"}]},{"signal": "Acceptor splice site","matrix": [{"name": "HSF Acceptor site (matrix AG)","sequence": "CCACCACCGAAGGG","CV": "71.79","position": "309"}]},{"signal": "Acceptor splice site","matrix": [{"name": "HSF Acceptor site (matrix AG)","sequence": "GGCCTGATTCAGCT","CV": "77.31","position": "321"}]},{"signal": "Acceptor splice site","matrix": [{"name": "MaxEnt Acceptor site","sequence": "TTCAGCTGTTACCCCTTCAGACA","CV": "6.96","position": "328"}]},{"signal": "Acceptor splice site","matrix": [{"name": "HSF Acceptor site (matrix AG)","sequence": "TTACCCCTTCAGAC","CV": "86.95","position": "336"}]},{"signal": "Acceptor splice site","matrix": [{"name": "HSF Acceptor site (matrix AG)","sequence": "CCCTTCAGACAGTT","CV": "79.08","position": "340"}]},{"signal": "Acceptor splice site","matrix": [{"name": "HSF Acceptor site (matrix AG)","sequence": "TGAAATTGTAAGTG","CV": "68.34","position": "355"}]},{"signal": "Donor splice site","matrix": [{"name": "HSF Donor site (matrix GT)","sequence": "ATTGTAAGT","CV": "83.59","position": "359","WT": "true"}]},{"signal": "Donor splice site","matrix": [{"name": "MaxEnt Donor site","sequence": "ATTGTAAGT","CV": "8.54","position": "359","WT": "true"}]},{"signal": "Acceptor splice site","matrix": [{"name": "HSF Acceptor site (matrix AG)","sequence": "GTAAGTGGGCAGAG","CV": "72.66","position": "362"}]},{"signal": "Donor splice site","matrix": [{"name": "HSF Donor site (matrix GT)","sequence": "TAAGTGGGC","CV": "75.44","position": "363"}]},{"signal": "Acceptor splice site","matrix": [{"name": "HSF Acceptor site (matrix AG)","sequence": "AAGTGGGCAGAGGG","CV": "65.22","position": "364"}]}]},"Branch point signals":{"matrix": [{"name": "BP","sequence": "TCTTCAG","CV": "71.67","position": "14"},{"name": "BP","sequence": "GCCTCAG","CV": "95.41","position": "20"},{"name": "BP","sequence": "TCTTCAG","CV": "71.67","position": "59"},{"name": "BP","sequence": "GGGTCAG","CV": "65.1","position": "80"},{"name": "BP","sequence": "TCATGAC","CV": "67.58","position": "91"},{"name": "BP","sequence": "ACATCAT","CV": "65.9","position": "96"},{"name": "BP","sequence": "TCATCAC","CV": "72.51","position": "99"},{"name": "BP","sequence": "ATCACAG","CV": "74.33","position": "101"},{"name": "BP","sequence": "CACAGAA","CV": "66.25","position": "103"},{"name": "BP","sequence": "CGGTCAC","CV": "69.46","position": "116"},{"name": "BP","sequence": "GTCACAG","CV": "76.95","position": "118"},{"name": "BP","sequence": "AGCACAC","CV": "75.85","position": "123"},{"name": "BP","sequence": "CACACAC","CV": "77.81","position": "125"},{"name": "BP","sequence": "GGCGGAC","CV": "73.53","position": "136"},{"name": "BP","sequence": "GACTCAG","CV": "88.54","position": "140"},{"name": "BP","sequence": "CTGTGAC","CV": "65.74","position": "160"},{"name": "BP","sequence": "ACTTGAC","CV": "66.74","position": "165"},{"name": "BP","sequence": "CACTGAT","CV": "84.07","position": "181"},{"name": "BP","sequence": "TCTTGAG","CV": "66.74","position": "204"},{"name": "BP","sequence": "GCCACAG","CV": "80.32","position": "210"},{"name": "BP","sequence": "GTCCCAT","CV": "81.84","position": "236"},{"name": "BP","sequence": "ATCTGAC","CV": "87.21","position": "241"},{"name": "BP","sequence": "CCCTGCC","CV": "65.2","position": "247"},{"name": "BP","sequence": "ACCTGAA","CV": "83.96","position": "258"},{"name": "BP","sequence": "GACCCAG","CV": "79.51","position": "271"},{"name": "BP","sequence": "CGCCCAT","CV": "82.26","position": "285"},{"name": "BP","sequence": "CCATCAG","CV": "71.31","position": "288"},{"name": "BP","

","CV": "70.81","position": "369"}]}}

LMNA Exon 11 HSF Output (JSON):

{"infos": {"HSF Pro version": "4.1.4 - July 2020","Elapsed time": "1,9833s","Sequence": "AGCCTTGTCTCCCTTCCCAGGGCTCCCACTGCAGCAGCTCGGGGGACCCCGCTGAGTACA ACCTGCGCTCGCGCACCGTGCTGTGCGGGACCTGCGGGCAGCCTGCCGACAAGGCATCT GCCAGCGGCTCAGGAGCCCAGGTGGGCGGACCCATCTCCTCTGGCTCTTCTGCCTCCAGT GTCACGGTCACTCGCAGCTACCGCAGTGTGGGGGGCAGTGGGGGTGGCAGCTTCGGGG ACAATCTGGTCACCCGCTCCTACCTCCTGGGCAACTCCAGCCCCCGAACCCAGGTGAGTT GTCTCTGCTTTGT","Exon start": "21","Exon end": "290","Genomic start": "-20","Genomic end": "290"},"ESE/ESS signals":{"matrix": [{"name": "IIE (ESS Site)","sequence": "GCCTTG","CV": "NA","position": "2"},{"name": "IIE (ESS Site)","sequence": "CCTTGT","CV": "NA","position": "3"},{"name": "IIE (ESS Site)","sequence": "CTTGTC","CV": "NA","position": "4"},{"name": "IIE (ESS Site)","sequence": "TTGTCT","CV": "NA","position": "5"},{"name": "IIE (ESS Site)","sequence": "TGTCTC","CV": "NA","position": "6"},{"name": "ESE_SRp40 (ESE Site)","sequence": "TGTCTCC","CV": "80.26","position": "6"},{"name": "ESE_SC35 (ESE Site)","sequence": "GTCTCCCT","CV": "76.35","position": "7"},{"name": "Sironi_motif3 (ESS Site)","sequence": "TCTCCCTT","CV": "88.61","position": "8"},{"name": "ESE_ASF (ESE Site)","sequence": "CTCCCTT","CV": "73.71","position": "9"},{"name": "ESE_ASFB (ESE Site)","sequence": "CTCCCTT","CV": "77.46","position": "9"},{"name": "Sironi_motif3 (ESS Site)","sequence": "CCTTCCCA","CV": "70.81","position": "12"},{"name": "Sironi_motif3 (ESS Site)","sequence": "CTTCCCAG","CV": "82.83","position": "13"},{"name": "ESE_SRp40 (ESE Site)","sequence": "TCCCAGG","CV": "85.53","position": "15"},{"name": "ESE_ASF (ESE Site)","sequence": "CCCAGGG","CV": "82.56","position": "16"},{"name": "ESE_ASFB (ESE Site)","sequence": "CCCAGGG","CV": "87.46","position": "16"},{"name": "PESE (ESE Site)","sequence": "CCCAGGGC","CV": "7.127777386053","position": "16"},{"name": "Sironi_motif2 (ESS Site)","sequence": "CCAGGGC","CV": "60.64","position": "17"},{"name": "Sironi_motif1 (ESS Site)","sequence": "CCAGGGCT","CV": "72.43","position": "17"},{"name": "ESE_9G8 (ESE Site)","sequence": "CAGGGC","CV": "59.67","position": "18"},{"name": "ESS_hnRNPA1 (ESS Site)","sequence": "CAGGGC","CV": "83.22","position": "18"},{"name": "ESE_SC35 (ESE Site)","sequence": "GGCTCCCA","CV": "91.47","position": "21"},{"name": "Sironi_motif3 (ESS Site)","sequence": "GCTCCCAC","CV": "94.24","position": "22"},{"name": "ESE_ASF (ESE Site)","sequence": "CCCACTG","CV": "73.76","position": "25"},{"name": "ESE_ASFB (ESE Site)","sequence": "CCCACTG","CV": "77.15","position": "25"},{"name": "ESE_SRp40 (ESE Site)","sequence": "CCACTGC","CV": "91.16","position": "26"},{"name": "ESE_SRp55 (ESE Site)","sequence": "TGCAGC","CV": "87.22","position": "30"},{"name": "PESE (ESE Site)","sequence": "GCAGCAGC","CV": "11.59855495046","position": "31"},{"name": "ESE_SRp55 (ESE Site)","sequence": "AGCAGC","CV": "75.18","position": "33"},{"name": "PESE (ESE Site)","sequence": "AGCAGCTC","CV": "37.85001182087","position": "33"},{"name": "Sironi_motif2 (ESS Site)","sequence": "GCTCGGG","CV": "65.36","position": "37"},{"name": "ESE_ASFB (ESE Site)","sequence": "CTCGGGG","CV": "76.15","position": "38"},{"name": "Sironi_motif2 (ESS Site)","sequence": "CTCGGGG","CV": "61.17","position": "38"},{"name": "Sironi_motif1 (ESS Site)","sequence": "CTCGGGGG","CV": "60.71","position": "38"},{"name": "IIE (ESS Site)","sequence": "TCGGGG","CV": "NA","position": "39"},{"name": "Sironi_motif2 (ESS Site)","sequence": "TCGGGGG","CV": "71.04","position": "39"},{"name": "IIE (ESS Site)","sequence": "CGGGGG","CV": "NA","position": "40"},{"name": "Fas ESS (ESS Site)","sequence": "CGGGGG","CV": "NA","position": "40"},{"name": "ESE_ASF (ESE Site)","sequence": "CGGGGGA","CV": "75.86","position": "40"},{"name": "ESE_ASFB (ESE Site)","sequence": "CGGGGGA","CV": "82.08","position": "40"},{"name": "Sironi_motif2 (ESS Site)","sequence": "CGGGGGA","CV": "67.24","position": "40"},{"name": "IIE (ESS Site)","sequence": "GGGGGA","CV": "NA","position": "41"},{"name": "Fas ESS (ESS Site)","sequence": "GGGGGA","CV": "NA","position": "41"},{"name": "ESE_9G8 (ESE Site)","sequence": "GGGGAC","CV": "82.08","position": "42"},{"name": "EIE (ESE Site)","sequence": "GGGGAC","CV": "NA","position": "42"},{"name": "IIE (ESS Site)","sequence": "GGGGAC","CV": "NA","position": "42"},{"name": "EIE (ESE Site)","sequence": "GGGACC","CV": "NA","position":

"43"},{"name": "EIE (ESE Site)","sequence": "GGACCC","CV": "NA","position": "44"},{"name": "ESE_SC35 (ESE Site)","sequence": "GGACCCCG","CV": "89.44","position": "44"},{"name": "EIE (ESE Site)","sequence": "GACCCC","CV": "NA","position": "45"},{"name": "EIE (ESE Site)","sequence": "ACCCCG","CV": "NA","position": "46"},{"name": "ESE_ASFB (ESE Site)","sequence": "CCCCGCT","CV": "73.15","position": "47"},{"name": "ESE_ASFB (ESE Site)","sequence": "CCGCTGA","CV": "72.38","position": "49"},{"name": "ESE_9G8 (ESE Site)","sequence": "GCTGAG","CV": "63.29","position": "51"},{"name": "PESE (ESE Site)","sequence": "GCTGAGTA","CV": "26.34975673893","position": "51"},{"name": "IIE (ESS Site)","sequence": "CTGAGT","CV": "NA","position": "52"},{"name": "ESE_ASF (ESE Site)","sequence": "CTGAGTA","CV": "76.5","position": "52"},{"name": "ESE_ASFB (ESE Site)","sequence": "CTGAGTA","CV": "77.85","position": "52"},{"name": "ESE_SRp55 (ESE Site)","sequence": "TGAGTA","CV": "80.24","position": "53"},{"name": "ESE_9G8 (ESE Site)","sequence": "GAGTAC","CV": "74.43","position": "54"},{"name": "EIE (ESE Site)","sequence": "GAGTAC","CV": "NA","position": "54"},{"name": "EIE (ESE Site)","sequence": "AGTACA","CV": "NA","position": "55"},{"name": "PESE (ESE Site)","sequence": "AGTACAAC","CV": "36.78779256654","position": "55"},{"name": "EIE (ESE Site)","sequence": "GTACAA","CV": "NA","position": "56"},{"name": "EIE (ESE Site)","sequence": "TACAAC","CV": "NA","position": "57"},{"name": "EIE (ESE Site)","sequence": "ACAACC","CV": "NA","position": "58"},{"name": "EIE (ESE Site)","sequence": "CAACCT","CV": "NA","position": "59"},{"name": "EIE (ESE Site)","sequence": "AACCTG","CV": "NA","position": "60"},{"name": "EIE (ESE Site)","sequence": "ACCTGC","CV": "NA","position": "61"},{"name": "EIE (ESE Site)","sequence": "CCTGCG","CV": "NA","position": "62"},{"name": "EIE (ESE Site)","sequence": "CTGCGC","CV": "NA","position": "63"},{"name": "ESE_ASFB (ESE Site)","sequence": "CTGCGCT","CV": "72.15","position": "63"},{"name": "PESE (ESE Site)","sequence": "GCGCTCGC","CV": "2.636261452764","position": "65"},{"name": "ESE_ASFB (ESE Site)","sequence": "CGCTCGC","CV": "76.15","position": "66"},{"name": "ESE_SC35 (ESE Site)","sequence": "CGCTCGCG","CV": "76.16","position": "66"},{"name": "Sironi_motif3 (ESS Site)","sequence": "GCTCGCGC","CV": "63.44","position": "67"},{"name": "Sironi_motif3 (ESS Site)","sequence": "TCGCGCAC","CV": "69.12","position": "69"},{"name": "ESE_ASF (ESE Site)","sequence": "CGCACCG","CV": "78.54","position": "72"},{"name": "ESE_ASFB (ESE Site)","sequence": "CGCACCG","CV": "83.85","position": "72"},{"name": "ESE_SC35 (ESE Site)","sequence": "GCACCGTG","CV": "75.3","position": "73"},{"name": "ESE_ASF (ESE Site)","sequence": "CACCGTG","CV": "74.46","position": "74"},{"name": "ESE_ASFB (ESE Site)","sequence": "CACCGTG","CV": "77.62","position": "74"},{"name": "EIE (ESE Site)","sequence": "ACCGTG","CV": "NA","position": "75"},{"name": "PESE (ESE Site)","sequence": "CCGTGCTG","CV": "47.1001538787","position": "76"},{"name": "EIE (ESE Site)","sequence": "GTGCTG","CV": "NA","position": "78"},{"name": "IIE (ESS Site)","sequence": "GTGCTG","CV": "NA","position": "78"},{"name": "IIE (ESS Site)","sequence": "TGCTGT","CV": "NA","position": "79"},{"name": "Sironi_motif2 (ESS Site)","sequence": "TGCTGTG","CV": "63.08","position": "79"},{"name": "PESE (ESE Site)","sequence": "TGCTGTGC","CV": "37.21707680248","position": "79"},{"name": "EIE (ESE Site)","sequence": "GCTGTG","CV": "NA","position": "80"},{"name": "IIE (ESS Site)","sequence": "GCTGTG","CV": "NA","position": "80"},{"name": "PESE (ESE Site)","sequence": "GCTGTGCG","CV": "36.95369951763","position": "80"},{"name": "EIE (ESE Site)","sequence": "CTGTGC","CV": "NA","position": "81"},{"name": "IIE (ESS Site)","sequence": "CTGTGC","CV": "NA","position": "81"},{"name": "PESE (ESE Site)","sequence": "CTGTGCGG","CV": "56.57427032049","position": "81"},{"name": "EIE (ESE Site)","sequence": "TGTGCG","CV": "NA","position": "82"},{"name": "Sironi_motif2 (ESS Site)","sequence": "TGTGCGG","CV": "83.58","position": "82"},{"name": "ESE_SRp55 (ESE Site)","sequence": "TGCGGG","CV": "79.79","position": "84"},{"name": "EIE (ESE Site)","sequence": "TGCGGG","CV": "NA","position": "84"},{"name": "Sironi_motif2 (ESS Site)","sequence": "TGCGGGA","CV": "77.11","position": "84"},{"name": "EIE (ESE Site)","sequence": "GCGGGA","CV": "NA","position": "85"},{"name": "ESE_9G8 (ESE Site)","sequence": "CGGGAC","CV": "62.76","position": "86"},{"name": "EIE (ESE Site)","sequence": "CGGGAC","CV": "NA","position": "86"},{"name": "EIE (ESE Site)","sequence": "GGGACC","CV": "NA","position": "87"},{"name": "EIE (ESE Site)","sequence": "GGACCT","CV":

"NA","position": "88"},{"name": "PESE (ESE Site)","sequence": "GGACCTGC","CV": "51.06781861393","position": "88"},{"name": "EIE (ESE Site)","sequence": "GACCTG","CV": "NA","position": "89"},{"name": "ESE_SC35 (ESE Site)","sequence": "GACCTGCG","CV": "83.17","position": "89"},{"name": "EIE (ESE Site)","sequence": "ACCTGC","CV": "NA","position": "90"},{"name": "EIE (ESE Site)","sequence": "CCTGCG","CV": "NA","position": "91"},{"name": "EIE (ESE Site)","sequence": "CTGCGG","CV": "NA","position": "92"},{"name": "ESE_ASF (ESE Site)","sequence": "CTGCGGG","CV": "78.83","position": "92"},{"name": "ESE_ASFB (ESE Site)","sequence": "CTGCGGG","CV": "83.31","position": "92"},{"name": "ESE_SRp55 (ESE Site)","sequence": "TGCGGG","CV": "79.79","position": "93"},{"name": "EIE (ESE Site)","sequence": "TGCGGG","CV": "NA","position": "93"},{"name": "Sironi_motif2 (ESS Site)","sequence": "TGCGGGC","CV": "77.92","position": "93"},{"name": "ESE_9G8 (ESE Site)","sequence": "GCGGGC","CV": "61.82","position": "94"},{"name": "IIE (ESS Site)","sequence": "GGGCAG","CV": "NA","position": "96"},{"name": "PESE (ESE Site)","sequence": "GGCAGCCT","CV": "41.05574888324","position": "97"},{"name": "ESE_9G8 (ESE Site)","sequence": "GCAGCC","CV": "63.23","position": "98"},{"name": "EIE (ESE Site)","sequence": "CAGCCT","CV": "NA","position": "99"},{"name": "ESE_ASF (ESE Site)","sequence": "CAGCCTG","CV": "76.56","position": "99"},{"name": "ESE_ASFB (ESE Site)","sequence": "CAGCCTG","CV": "76.31","position": "99"},{"name": "EIE (ESE Site)","sequence": "AGCCTG","CV": "NA","position": "100"},{"name": "EIE (ESE Site)","sequence": "GCCTGC","CV": "NA","position": "101"},{"name": "ESE_SC35 (ESE Site)","sequence": "GCCTGCCG","CV": "76.29","position": "101"},{"name": "EIE (ESE Site)","sequence": "CCTGCC","CV": "NA","position": "102"},{"name": "Sironi_motif3 (ESS Site)","sequence": "CCTGCCGA","CV": "68.31","position": "102"},{"name": "PESE (ESE Site)","sequence": "CCTGCCGA","CV": "59.56474311382","position": "102"},{"name": "ESE_ASF (ESE Site)","sequence": "CTGCCGA","CV": "86.64","position": "103"},{"name": "ESE_ASFB (ESE Site)","sequence": "CTGCCGA","CV": "88.23","position": "103"},{"name": "ESE_SRp55 (ESE Site)","sequence": "TGCCGA","CV": "74.03","position": "104"},{"name": "ESE_9G8 (ESE Site)","sequence": "GCCGAC","CV": "82.02","position": "105"},{"name": "EIE (ESE Site)","sequence": "GCCGAC","CV": "NA","position": "105"},{"name": "ESE_ASF (ESE Site)","sequence": "CCGACAA","CV": "73.94","position": "106"},{"name": "ESE_ASFB (ESE Site)","sequence": "CCGACAA","CV": "75.77","position": "106"},{"name": "EIE (ESE Site)","sequence": "CGACAA","CV": "NA","position": "107"},{"name": "ESE_SRp40 (ESE Site)","sequence": "CGACAAG","CV": "89.43","position": "107"},{"name": "ESE_9G8 (ESE Site)","sequence": "GACAAG","CV": "63.56","position": "108"},{"name": "EIE (ESE Site)","sequence": "GACAAG","CV": "NA","position": "108"},{"name": "EIE (ESE Site)","sequence": "ACAAGG","CV": "NA","position": "109"},{"name": "ESE_9G8 (ESE Site)","sequence": "CAAGGC","CV": "64.5","position": "110"},{"name": "ESS_hnRNPA1 (ESS Site)","sequence": "CAAGGC","CV": "66.08","position": "110"},{"name": "ESS_hnRNPA1 (ESS Site)","sequence": "AAGGCA","CV": "75.13","position": "111"},{"name": "ESE_SRp55 (ESE Site)","sequence": "GGCATC","CV": "74.48","position": "113"},{"name": "ESE_SC35 (ESE Site)","sequence": "GGCATCTG","CV": "78.38","position": "113"},{"name": "PESE (ESE Site)","sequence": "GCATCTGC","CV": "46.71690882169","position": "114"},{"name": "PESE (ESE Site)","sequence": "CATCTGCC","CV": "26.96112385369","position": "115"},{"name": "Sironi_motif3 (ESS Site)","sequence": "TCTGCCAG","CV": "84.02","position": "117"},{"name": "ESE_ASFB (ESE Site)","sequence": "CTGCCAG","CV": "71.23","position": "118"},{"name": "PESE (ESE Site)","sequence": "CTGCCAGC","CV": "50.79697551628","position": "118"},{"name": "Sironi_motif1 (ESS Site)","sequence": "CCAGCGGC","CV": "74.87","position": "121"},{"name": "ESS_hnRNPA1 (ESS Site)","sequence": "CAGCGG","CV": "67.03","position": "122"},{"name": "ESE_ASF (ESE Site)","sequence": "CAGCGGC","CV": "76.79","position": "122"},{"name": "ESE_ASFB (ESE Site)","sequence": "CAGCGGC","CV": "79","position": "122"},{"name": "ESE_SRp55 (ESE Site)","sequence": "AGCGGC","CV": "79.09","position": "123"},{"name": "EIE (ESE Site)","sequence": "AGCGGC","CV": "NA","position": "123"},{"name": "ESE_SC35 (ESE Site)","sequence": "GGCTCAGG","CV": "76.1","position": "126"},{"name": "ESE_SRp40 (ESE Site)","sequence": "GCTCAGG","CV": "78.65","position": "127"},{"name": "ESE_ASF (ESE Site)","sequence": "CTCAGGA","CV": "90.31","position": "128"},{"name": "ESE_ASFB (ESE Site)","sequence":

"CTCAGGA","CV": "92.69","position": "128"},{"name": "RESCUE ESE (ESE Site)","sequence": "TCAGGA","CV": "NA","position": "129"},{"name": "Sironi_motif2 (ESS Site)","sequence": "TCAGGAG","CV": "63.63","position": "129"},{"name": "ESE_9G8 (ESE Site)","sequence": "CAGGAG","CV": "60.54","position": "130"},{"name": "ESS_hnRNPA1 (ESS Site)","sequence": "CAGGAG","CV": "73.94","position": "130"},{"name": "Sironi_motif1 (ESS Site)","sequence": "CAGGAGCC","CV": "68.14","position": "130"},{"name": "EIE (ESE Site)","sequence": "AGGAGC","CV": "NA","position": "131"},{"name": "PESE (ESE Site)","sequence": "AGGAGCCC","CV": "69.66474352859","position": "131"},{"name": "ESE_9G8 (ESE Site)","sequence": "GGAGCC","CV": "67.52","position": "132"},{"name": "EIE (ESE Site)","sequence": "GGAGCC","CV": "NA","position": "132"},{"name": "EIE (ESE Site)","sequence": "GAGCCC","CV": "NA","position": "133"},{"name": "ESE_SC35 (ESE Site)","sequence": "GAGCCCAG","CV": "76.9","position": "133"},{"name": "Sironi_motif3 (ESS Site)","sequence": "GAGCCCAG","CV": "65.04","position": "133"},{"name": "ESE_ASF (ESE Site)","sequence": "CCCAGGT","CV": "84.77","position": "136"},{"name": "ESE_ASFB (ESE Site)","sequence": "CCCAGGT","CV": "89","position": "136"},{"name": "ESS_hnRNPA1 (ESS Site)","sequence": "CAGGTG","CV": "70.6","position": "138"},{"name": "IIE (ESS Site)","sequence": "AGGTGG","CV": "NA","position": "139"},{"name": "Fas ESS (ESS Site)","sequence": "AGGTGG","CV": "NA","position": "139"},{"name": "Sironi_motif2 (ESS Site)","sequence": "AGGTGGG","CV": "73.16","position": "139"},{"name": "IIE (ESS Site)","sequence": "GGTGGG","CV": "NA","position": "140"},{"name": "Fas ESS (ESS Site)","sequence": "GGTGGG","CV": "NA","position": "140"},{"name": "Sironi_motif2 (ESS Site)","sequence": "GGTGGGC","CV": "87.38","position": "140"},{"name": "ESE_9G8 (ESE Site)","sequence": "GTGGGC","CV": "61.82","position": "141"},{"name": "IIE (ESS Site)","sequence": "GTGGGC","CV": "NA","position": "141"},{"name": "IIE (ESS Site)","sequence": "TGGGCG","CV": "NA","position": "142"},{"name": "Sironi_motif2 (ESS Site)","sequence": "TGGGCGG","CV": "71.04","position": "142"},{"name": "IIE (ESS Site)","sequence": "GGGCGG","CV": "NA","position": "143"},{"name": "ESE_ASF (ESE Site)","sequence": "GGGCGGA","CV": "77.78","position": "143"},{"name": "ESE_9G8 (ESE Site)","sequence": "GCGGAC","CV": "77.79","position": "145"},{"name": "EIE (ESE Site)","sequence": "CGGACC","CV": "NA","position": "146"},{"name": "ESE_ASFB (ESE Site)","sequence": "CGGACCC","CV": "73.31","position": "146"},{"name": "EIE (ESE Site)","sequence": "GGACCC","CV": "NA","position": "147"},{"name": "Sironi_motif3 (ESS Site)","sequence": "GGACCCAT","CV": "60.98","position": "147"},{"name": "PESE (ESE Site)","sequence": "GGACCCAT","CV": "36.32034973185","position": "147"},{"name": "EIE (ESE Site)","sequence": "GACCCA","CV": "NA","position": "148"},{"name": "ESE_SC35 (ESE Site)","sequence": "GACCCATC","CV": "75.67","position": "148"},{"name": "EIE (ESE Site)","sequence": "ACCCAT","CV": "NA","position": "149"},{"name": "Sironi_motif3 (ESS Site)","sequence": "TCTCCTCT","CV": "69.73","position": "154"},{"name": "ESE_SC35 (ESE Site)","sequence": "CTCCTCTG","CV": "78.44","position": "155"},{"name": "ESE_SRp40 (ESE Site)","sequence": "CCTCTGG","CV": "89.96","position": "157"},{"name": "IIE (ESS Site)","sequence": "GCTCTT","CV": "NA","position": "163"},{"name": "ESE_SC35 (ESE Site)","sequence": "CTCTTCTG","CV": "77.03","position": "164"},{"name": "IIE (ESS Site)","sequence": "TCTTCT","CV": "NA","position": "165"},{"name": "IIE (ESS Site)","sequence": "CTTCTG","CV": "NA","position": "166"},{"name": "ESE_SRp40 (ESE Site)","sequence": "CTTCTGC","CV": "83.02","position": "166"},{"name": "Sironi_motif3 (ESS Site)","sequence": "TCTGCCTC","CV": "75.03","position": "168"},{"name": "EIE (ESE Site)","sequence": "CTGCCT","CV": "NA","position": "169"},{"name": "ESE_SRp55 (ESE Site)","sequence": "TGCCTC","CV": "81.58","position": "170"},{"name": "ESE_SC35 (ESE Site)","sequence": "TGCCTCCA","CV": "75.61","position": "170"},{"name": "ESE_SC35 (ESE Site)","sequence": "GCCTCCAG","CV": "79.42","position": "171"},{"name": "Sironi_motif3 (ESS Site)","sequence": "GCCTCCAG","CV": "61.3","position": "171"},{"name": "Sironi_motif3 (ESS Site)","sequence": "CCTCCAGT","CV": "61.41","position": "172"},{"name": "ESE_ASFB (ESE Site)","sequence": "CTCCAGT","CV": "74.69","position": "173"},{"name": "EIE (ESE Site)","sequence": "CCAGTG","CV": "NA","position": "175"},{"name": "Sironi_motif1 (ESS Site)","sequence": "CCAGTGTC","CV": "61.34","position": "175"},{"name": "ESS_hnRNPA1 (ESS Site)","sequence": "CAGTGT","CV": "68.94","position": "176"},{"name": "IIE (ESS Site)","sequence": "CAGTGT","CV":

"NA","position": "176"},{"name": "ESE_SRp55 (ESE Site)","sequence": "AGTGTC","CV": "74.35","position": "177"},{"name": "IIE (ESS Site)","sequence": "GTGTCA","CV": "NA","position": "178"},{"name": "ESE_SRp40 (ESE Site)","sequence": "TGTCACG","CV": "86.85","position": "179"},{"name": "ESE_ASF (ESE Site)","sequence": "GTCACGG","CV": "80.4","position": "180"},{"name": "ESE_9G8 (ESE Site)","sequence": "GGTCAC","CV": "60.94","position": "185"},{"name": "ESE_SC35 (ESE Site)","sequence": "GGTCACTC","CV": "79.3","position": "185"},{"name": "ESE_SRp40 (ESE Site)","sequence": "TCACTCG","CV": "96.55","position": "187"},{"name": "ESE_ASF (ESE Site)","sequence": "CACTCGC","CV": "73.3","position": "188"},{"name": "ESE_ASFB (ESE Site)","sequence": "CACTCGC","CV": "73.92","position": "188"},{"name": "Sironi_motif3 (ESS Site)","sequence": "ACTCGCAG","CV": "75.36","position": "189"},{"name": "ESE_SRp40 (ESE Site)","sequence": "TCGCAGC","CV": "79.19","position": "191"},{"name": "ESE_SRp55 (ESE Site)","sequence": "CGCAGC","CV": "81.9","position": "192"},{"name": "ESE_ASF (ESE Site)","sequence": "CGCAGCT","CV": "77.2","position": "192"},{"name": "ESE_ASFB (ESE Site)","sequence": "CGCAGCT","CV": "83.77","position": "192"},{"name": "EIE (ESE Site)","sequence": "CAGCTA","CV": "NA","position": "194"},{"name": "EIE (ESE Site)","sequence": "AGCTAC","CV": "NA","position": "195"},{"name": "ESE_SC35 (ESE Site)","sequence": "AGCTACCG","CV": "80.59","position": "195"},{"name": "EIE (ESE Site)","sequence": "GCTACC","CV": "NA","position": "196"},{"name": "Sironi_motif3 (ESS Site)","sequence": "GCTACCGC","CV": "63.44","position": "196"},{"name": "PESS (ESS Site)","sequence": "GCTACCGC","CV": "-0.3090016963986","position": "196"},{"name": "EIE (ESE Site)","sequence": "CTACCG","CV": "NA","position": "197"},{"name": "ESE_SRp40 (ESE Site)","sequence": "CTACCGC","CV": "80.38","position": "197"},{"name": "EIE (ESE Site)","sequence": "TACCGC","CV": "NA","position": "198"},{"name": "EIE (ESE Site)","sequence": "ACCGCA","CV": "NA","position": "199"},{"name": "ESE_ASF (ESE Site)","sequence": "CGCAGTG","CV": "77.61","position": "201"},{"name": "ESE_ASFB (ESE Site)","sequence": "CGCAGTG","CV": "83","position": "201"},{"name": "EIE (ESE Site)","sequence": "GCAGTG","CV": "NA","position": "202"},{"name": "ESS_hnRNPA1 (ESS Site)","sequence": "CAGTGT","CV": "68.94","position": "203"},{"name": "IIE (ESS Site)","sequence": "CAGTGT","CV": "NA","position": "203"},{"name": "EIE (ESE Site)","sequence": "AGTGTG","CV": "NA","position": "204"},{"name": "IIE (ESS Site)","sequence": "AGTGTG","CV": "NA","position": "204"},{"name": "Sironi_motif2 (ESS Site)","sequence": "AGTGTGG","CV": "76.75","position": "204"},{"name": "EIE (ESE Site)","sequence": "GTGTGG","CV": "NA","position": "205"},{"name": "IIE (ESS Site)","sequence": "GTGTGG","CV": "NA","position": "205"},{"name": "Fas ESS (ESS Site)","sequence": "GTGTGG","CV": "NA","position": "205"},{"name": "Sironi_motif2 (ESS Site)","sequence": "GTGTGGG","CV": "60.01","position": "205"},{"name": "IIE (ESS Site)","sequence": "TGTGGG","CV": "NA","position": "206"},{"name": "Sironi_motif2 (ESS Site)","sequence": "TGTGGGG","CV": "100.48","position": "206"},{"name": "IIE (ESS Site)","sequence": "GTGGGG","CV": "NA","position": "207"},{"name": "Fas ESS (ESS Site)","sequence": "GTGGGG","CV": "NA","position": "207"},{"name": "Sironi_motif2 (ESS Site)","sequence": "GTGGGGG","CV": "67.96","position": "207"},{"name": "IIE (ESS Site)","sequence": "TGGGGG","CV": "NA","position": "208"},{"name": "Fas ESS (ESS Site)","sequence": "TGGGGG","CV": "NA","position": "208"},{"name": "Sironi_motif2 (ESS Site)","sequence": "TGGGGGG","CV": "87.94","position": "208"},{"name": "IIE (ESS Site)","sequence": "GGGGGG","CV": "NA","position": "209"},{"name": "Fas ESS (ESS Site)","sequence": "GGGGGG","CV": "NA","position": "209"},{"name": "Sironi_motif2 (ESS Site)","sequence": "GGGGGGC","CV": "74.84","position": "209"},{"name": "ESE_9G8 (ESE Site)","sequence": "GGGGGC","CV": "66.11","position": "210"},{"name": "IIE (ESS Site)","sequence": "GGGGGC","CV": "NA","position": "210"},{"name": "IIE (ESS Site)","sequence": "GGGCAG","CV": "NA","position": "212"},{"name": "ESE_SC35 (ESE Site)","sequence": "GGGCAGTG","CV": "76.9","position": "212"},{"name": "EIE (ESE Site)","sequence": "GCAGTG","CV": "NA","position": "214"},{"name": "Sironi_motif2 (ESS Site)","sequence": "GCAGTGG","CV": "60.55","position": "214"},{"name": "Sironi_motif1 (ESS Site)","sequence": "GCAGTGGG","CV": "63.5","position": "214"},{"name": "ESS_hnRNPA1 (ESS Site)","sequence": "CAGTGG","CV": "67.03","position": "215"},{"name": "EIE (ESE Site)","sequence": "CAGTGG","CV": "NA","position": "215"},{"name": "ESE_ASF (ESE Site)","sequence": "CAGTGGG","CV": "76.85","position": "215"},{"name": "ESE_ASFB (ESE

Site)","sequence": "CAGTGGG","CV": "77","position": "215"},{"name": "IIE (ESS Site)","sequence": "AGTGGG","CV": "NA","position": "216"},{"name": "Sironi_motif2 (ESS Site)","sequence": "AGTGGGG","CV": "93.66","position": "216"},{"name": "IIE (ESS Site)","sequence": "GTGGGG","CV": "NA","position": "217"},{"name": "Fas ESS (ESS Site)","sequence": "GTGGGG","CV": "NA","position": "217"},{"name": "Sironi_motif2 (ESS Site)","sequence": "GTGGGGG","CV": "67.96","position": "217"},{"name": "IIE (ESS Site)","sequence": "TGGGGG","CV": "NA","position": "218"},{"name": "Fas ESS (ESS Site)","sequence": "TGGGGG","CV": "NA","position": "218"},{"name": "Sironi_motif2 (ESS Site)","sequence": "TGGGGGT","CV": "75.88","position": "218"},{"name": "IIE (ESS Site)","sequence": "GGGGGT","CV": "NA","position": "219"},{"name": "Fas ESS (ESS Site)","sequence": "GGGGGT","CV": "NA","position": "219"},{"name": "Sironi_motif2 (ESS Site)","sequence": "GGGGGTG","CV": "67.96","position": "219"},{"name": "IIE (ESS Site)","sequence": "GGGGTG","CV": "NA","position": "220"},{"name": "Fas ESS (ESS Site)","sequence": "GGGGTG","CV": "NA","position": "220"},{"name": "Sironi_motif2 (ESS Site)","sequence": "GGGGTGG","CV": "67.96","position": "220"},{"name": "IIE (ESS Site)","sequence": "GGGTGG","CV": "NA","position": "221"},{"name": "Fas ESS (ESS Site)","sequence": "GGGTGG","CV": "NA","position": "221"},{"name": "Sironi_motif2 (ESS Site)","sequence": "GGGTGGC","CV": "66.88","position": "221"},{"name": "ESE_9G8 (ESE Site)","sequence": "GGTGGC","CV": "66.72","position": "222"},{"name": "IIE (ESS Site)","sequence": "GGTGGC","CV": "NA","position": "222"},{"name": "Sironi_motif2 (ESS Site)","sequence": "GGTGGCA","CV": "69.67","position": "222"},{"name": "Fas ESS (ESS Site)","sequence": "GTGGCA","CV": "NA","position": "223"},{"name": "PESE (ESE Site)","sequence": "TGGCAGCT","CV": "31.69154579654","position": "224"},{"name": "PESE (ESE Site)","sequence": "GCAGCTTC","CV": "32.49246160291","position": "226"},{"name": "EIE (ESE Site)","sequence": "GCTTCG","CV": "NA","position": "229"},{"name": "Sironi_motif2 (ESS Site)","sequence": "TTCGGGG","CV": "71.04","position": "231"},{"name": "IIE (ESS Site)","sequence": "TCGGGG","CV": "NA","position": "232"},{"name": "Sironi_motif2 (ESS Site)","sequence": "TCGGGGA","CV": "60.21","position": "232"},{"name": "IIE (ESS Site)","sequence": "CGGGGA","CV": "NA","position": "233"},{"name": "ESE_9G8 (ESE Site)","sequence": "GGGGAC","CV": "82.08","position": "234"},{"name": "EIE (ESE Site)","sequence": "GGGGAC","CV": "NA","position": "234"},{"name": "IIE (ESS Site)","sequence": "GGGGAC","CV": "NA","position": "234"},{"name": "EIE (ESE Site)","sequence": "GGACAA","CV": "NA","position": "236"},{"name": "ESE_9G8 (ESE Site)","sequence": "GACAAT","CV": "65.57","position": "237"},{"name": "RESCUE ESE (ESE Site)","sequence": "GACAAT","CV": "NA","position": "237"},{"name": "EIE (ESE Site)","sequence": "GACAAT","CV": "NA","position": "237"},{"name": "IIE (ESS Site)","sequence": "TCTGGT","CV": "NA","position": "242"},{"name": "PESE (ESE Site)","sequence": "TGGTCACC","CV": "25.09384111921","position": "244"},{"name": "ESE_9G8 (ESE Site)","sequence": "GGTCAC","CV": "60.94","position": "245"},{"name": "ESE_SC35 (ESE Site)","sequence": "GGTCACCC","CV": "75.37","position": "245"},{"name": "ESE_SC35 (ESE Site)","sequence": "GTCACCCG","CV": "75.12","position": "246"},{"name": "EIE (ESE Site)","sequence": "TCACCC","CV": "NA","position": "247"},{"name": "ESE_SRp40 (ESE Site)","sequence": "TCACCCG","CV": "87.63","position": "247"},{"name": "Sironi_motif3 (ESS Site)","sequence": "TCACCCGC","CV": "72.69","position": "247"},{"name": "ESE_ASF (ESE Site)","sequence": "CACCCGC","CV": "81.8","position": "248"},{"name": "ESE_ASFB (ESE Site)","sequence": "CACCCGC","CV": "83.54","position": "248"},{"name": "EIE (ESE Site)","sequence": "ACCCGC","CV": "NA","position": "249"},{"name": "PESE (ESE Site)","sequence": "CCCGCTCC","CV": "5.894673972103","position": "250"},{"name": "ESE_ASFB (ESE Site)","sequence": "CGCTCCT","CV": "72.61","position": "252"},{"name": "ESE_SC35 (ESE Site)","sequence": "CGCTCCTA","CV": "82.93","position": "252"},{"name": "Sironi_motif3 (ESS Site)","sequence": "GCTCCTAC","CV": "77.05","position": "253"},{"name": "EIE (ESE Site)","sequence": "CTCCTA","CV": "NA","position": "254"},{"name": "EIE (ESE Site)","sequence": "TCCTAC","CV": "NA","position": "255"},{"name": "EIE (ESE Site)","sequence": "CCTACC","CV": "NA","position": "256"},{"name": "Sironi_motif3 (ESS Site)","sequence": "CCTACCTC","CV": "69.22","position": "256"},{"name": "EIE (ESE Site)","sequence": "CTACCT","CV": "NA","position": "257"},{"name": "ESE_SRp55 (ESE Site)","sequence": "TACCTC","CV": "77.68","position": "258"},{"name": "EIE (ESE Site)","sequence": "TACCTC","CV": "NA","position": "258"},{"name": "Sironi_motif3 (ESS

Site)","sequence": "CCTCCTGG","CV": "64.59","position": "260"},{"name": "PESE (ESE Site)","sequence": "CCTCCTGG","CV": "28.6658177761","position": "260"},{"name": "ESE_SRp40 (ESE Site)","sequence": "CTCCTGG","CV": "80.02","position": "261"},{"name": "ESE_ASFB (ESE Site)","sequence": "CTCCTGG","CV": "73.92","position": "261"},{"name": "IIE (ESS Site)","sequence": "TCCTGG","CV": "NA","position": "262"},{"name": "Fas ESS (ESS Site)","sequence": "TCCTGG","CV": "NA","position": "262"},{"name": "Sironi_motif2 (ESS Site)","sequence": "TCCTGGG","CV": "63.08","position": "262"},{"name": "IIE (ESS Site)","sequence": "CCTGGG","CV": "NA","position": "263"},{"name": "Fas ESS (ESS Site)","sequence": "CCTGGG","CV": "NA","position": "263"},{"name": "Sironi_motif2 (ESS Site)","sequence": "CCTGGGC","CV": "63.69","position": "263"},{"name": "IIE (ESS Site)","sequence": "CTGGGC","CV": "NA","position": "264"},{"name": "ESE_9G8 (ESE Site)","sequence": "GGCAAC","CV": "65.78","position": "267"},{"name": "EIE (ESE Site)","sequence": "GGCAAC","CV": "NA","position": "267"},{"name": "EIE (ESE Site)","sequence": "GCAACT","CV": "NA","position": "268"},{"name": "ESE_SC35 (ESE Site)","sequence": "AACTCCAG","CV": "78.93","position": "270"},{"name": "Sironi_motif3 (ESS Site)","sequence": "ACTCCAGC","CV": "62.17","position": "271"},{"name": "EIE (ESE Site)","sequence": "CAGCCC","CV": "NA","position": "275"},{"name": "EIE (ESE Site)","sequence": "AGCCCC","CV": "NA","position": "276"},{"name": "ESE_SC35 (ESE Site)","sequence": "AGCCCCCG","CV": "84.89","position": "276"},{"name": "EIE (ESE Site)","sequence": "GCCCCC","CV": "NA","position": "277"},{"name": "Sironi_motif3 (ESS Site)","sequence": "GCCCCCGA","CV": "68.59","position": "277"},{"name": "ESE_ASF (ESE Site)","sequence": "CCCCCGA","CV": "84.6","position": "278"},{"name": "ESE_ASFB (ESE Site)","sequence": "CCCCCGA","CV": "89.23","position": "278"},{"name": "Sironi_motif3 (ESS Site)","sequence": "CCCCCGAA","CV": "66.59","position": "278"},{"name": "ESE_ASFB (ESE Site)","sequence": "CCCCGAA","CV": "73.92","position": "279"},{"name": "EIE (ESE Site)","sequence": "GAACCC","CV": "NA","position": "283"},{"name": "ESE_SC35 (ESE Site)","sequence": "GAACCCAG","CV": "84.89","position": "283"},{"name": "Sironi_motif3 (ESS Site)","sequence": "GAACCCAG","CV": "65.04","position": "283"},{"name": "EIE (ESE Site)","sequence": "AACCCA","CV": "NA","position": "284"},{"name": "PESE (ESE Site)","sequence": "AACCCAGG","CV": "35.27638024214","position": "284"},{"name": "EIE (ESE Site)","sequence": "ACCCAG","CV": "NA","position": "285"},{"name": "ESE_SRp40 (ESE Site)","sequence": "ACCCAGG","CV": "79.25","position": "285"},{"name": "ESE_ASF (ESE Site)","sequence": "CCCAGGT","CV": "84.77","position": "286"},{"name": "ESE_ASFB (ESE Site)","sequence": "CCCAGGT","CV": "89","position": "286"},{"name": "ESS_hnRNPA1 (ESS Site)","sequence": "CAGGTG","CV": "70.6","position": "288"},{"name": "ESE_9G8 (ESE Site)","sequence": "GGTGAG","CV": "67.59","position": "290"},{"name": "Sironi_motif2 (ESS Site)","sequence": "GGTGAGT","CV": "68.45","position": "290"},{"name": "IIE (ESS Site)","sequence": "GTGAGT","CV": "NA","position": "291"},{"name": "IIE (ESS Site)","sequence": "TGAGTT","CV": "NA","position": "292"},{"name": "Sironi_motif2 (ESS Site)","sequence": "TGAGTTG","CV": "63.63","position": "292"},{"name": "IIE (ESS Site)","sequence": "GAGTTG","CV": "NA","position": "293"},{"name": "IIE (ESS Site)","sequence": "AGTTGT","CV": "NA","position": "294"},{"name": "Fas ESS (ESS Site)","sequence": "AGTTGT","CV": "NA","position": "294"},{"name": "ESE_9G8 (ESE Site)","sequence": "GTTGTC","CV": "59","position": "295"},{"name": "IIE (ESS Site)","sequence": "GTTGTC","CV": "NA","position": "295"},{"name": "IIE (ESS Site)","sequence": "TTGTCT","CV": "NA","position": "296"},{"name": "IIE (ESS Site)","sequence": "TGTCTC","CV": "NA","position": "297"},{"name": "ESE_SC35 (ESE Site)","sequence": "TGTCTCTG","CV": "78.81","position": "297"},{"name": "Sironi_motif3 (ESS Site)","sequence": "TGTCTCTG","CV": "60.28","position": "297"},{"name": "IIE (ESS Site)","sequence": "GTCTCT","CV": "NA","position": "298"},{"name": "IIE (ESS Site)","sequence": "TCTCTG","CV": "NA","position": "299"},{"name": "ESE_SRp40 (ESE Site)","sequence": "TCTCTGC","CV": "87.03","position": "299"},{"name": "IIE (ESS Site)","sequence": "TCTGCT","CV": "NA","position": "301"},{"name": "IIE (ESS Site)","sequence": "CTGCTT","CV": "NA","position": "302"},{"name": "IIE (ESS Site)","sequence": "TGCTTT","CV": "NA","position": "303"},{"name": "IIE (ESS Site)","sequence": "GCTTTG","CV": "NA","position": "304"},{"name": "IIE (ESS Site)","sequence": "CTTTGT","CV": "NA","position": "305"}]},"Splice site signals":{"matrix": [{"signal": "Acceptor splice site","matrix": [{"name": "MaxEnt Acceptor site","sequence":

"AGCCTTGTCTCCCTTCCCAGGGC","CV": "9.6","position": "1","WT": "true"}]},{"signal": "Acceptor splice site","matrix": [{"name": "HSF Acceptor site (matrix AG)","sequence": "CTCCCTTCCCAGGG","CV": "88.49","position": "9","WT": "true"}]},{"signal": "Acceptor splice site","matrix": [{"name": "MaxEnt Acceptor site","sequence": "TCCCAGGGCTCCCACTGCAGCAG","CV": "3.87","position": "15"}]},{"signal": "Acceptor splice site","matrix": [{"name": "HSF Acceptor site (matrix AG)","sequence": "CTCCCACTGCAGCA","CV": "83.62","position": "23"}]},{"signal": "Acceptor splice site","matrix": [{"name": "HSF Acceptor site (matrix AG)","sequence": "CCACTGCAGCAGCT","CV": "76.03","position": "26"}]},{"signal": "Acceptor splice site","matrix": [{"name": "HSF Acceptor site (matrix AG)","sequence": "GACCCCGCTGAGTA","CV": "66.44","position": "45"}]},{"signal": "Donor splice site","matrix": [{"name": "HSF Donor site (matrix GT)","sequence": "GCTGTGCGG","CV": "69.04","position": "80"}]},{"signal": "Acceptor splice site","matrix": [{"name": "HSF Acceptor site (matrix AG)","sequence": "ACCTGCGGGCAGCC","CV": "72.88","position": "90"}]},{"signal": "Acceptor splice site","matrix": [{"name": "HSF Acceptor site (matrix AG)","sequence": "CCTGCCGACAAGGC","CV": "71.92","position": "102"}]},{"signal": "Acceptor splice site","matrix": [{"name": "HSF Acceptor site (matrix AG)","sequence": "GGCATCTGCCAGCG","CV": "75.44","position": "113"}]},{"signal": "Acceptor splice site","matrix": [{"name": "HSF Acceptor site (matrix AG)","sequence": "CCAGCGGCTCAGGA","CV": "75.19","position": "121"}]},{"signal": "Acceptor splice site","matrix": [{"name": "HSF Acceptor site (matrix AG)","sequence": "GCGGCTCAGGAGCC","CV": "66.04","position": "124"}]},{"signal": "Acceptor splice site","matrix": [{"name": "HSF Acceptor site (matrix AG)","sequence": "TCAGGAGCCCAGGT","CV": "76.56","position": "129"}]},{"signal": "Donor splice site","matrix": [{"name": "HSF Donor site (matrix GT)","sequence": "CAGGTGGGC","CV": "87.03","position": "138"}]},{"signal": "Donor splice site","matrix": [{"name": "MaxEnt Donor site","sequence": "CAGGTGGGC","CV": "8.07","position": "138"}]},{"signal": "Acceptor splice site","matrix": [{"name": "MaxEnt Acceptor site","sequence": "TCTGGCTCTTCTGCCTCCAGTGT","CV": "8.95","position": "159"}]},{"signal": "Acceptor splice site","matrix": [{"name": "HSF Acceptor site (matrix AG)","sequence": "TTCTGCCTCCAGTG","CV": "87.44","position": "167"}]},{"signal": "Acceptor splice site","matrix": [{"name": "MaxEnt Acceptor site","sequence": "AGTGTCACGGTCACTCGCAGCTA","CV": "3.47","position": "177"}]},{"signal": "Donor splice site","matrix": [{"name": "HSF Donor site (matrix GT)","sequence": "ACGGTCACT","CV": "77.46","position": "183"}]},{"signal": "Acceptor splice site","matrix": [{"name": "HSF Acceptor site (matrix AG)","sequence": "GGTCACTCGCAGCT","CV": "79.53","position": "185"}]},{"signal": "Acceptor splice site","matrix": [{"name": "HSF Acceptor site (matrix AG)","sequence": "CAGCTACCGCAGTG","CV": "75.31","position": "194"}]},{"signal": "Donor splice site","matrix": [{"name": "HSF Donor site (matrix GT)","sequence": "GCAGTGTGG","CV": "70","position": "202"}]},{"signal": "Donor splice site","matrix": [{"name": "HSF Donor site (matrix GT)","sequence": "AGTGTGGGG","CV": "70.61","position": "204"}]},{"signal": "Acceptor splice site","matrix": [{"name": "HSF Acceptor site (matrix AG)","sequence": "TGTGGGGGGCAGTG","CV": "71.9","position": "206"}]},{"signal": "Donor splice site","matrix": [{"name": "HSF Donor site (matrix GT)","sequence": "GCAGTGGGG","CV": "70","position": "214"}]},{"signal": "Acceptor splice site","matrix": [{"name": "HSF Acceptor site (matrix AG)","sequence": "TGGGGGTGGCAGCT","CV": "73.47","position": "218"}]},{"signal": "Donor splice site","matrix": [{"name": "HSF Donor site (matrix GT)","sequence": "GGGGTGGCA","CV": "70.06","position": "220"}]},{"signal": "Donor splice site","matrix": [{"name": "HSF Donor site (matrix GT)","sequence": "CTGGTCACC","CV": "75.08","position": "243"}]},{"signal": "Acceptor splice site","matrix": [{"name": "HSF Acceptor site (matrix AG)","sequence": "GGGCAACTCCAGCC","CV": "73.88","position": "266"}]},{"signal": "Acceptor splice site","matrix": [{"name": "MaxEnt Acceptor site","sequence": "ACTCCAGCCCCCGAACCCAGGTG","CV": "3.33","position": "271"}]},{"signal": "Acceptor splice site","matrix": [{"name": "HSF Acceptor site (matrix AG)","sequence": "CCCCGAACCCAGGT","CV": "77.97","position": "279"}]},{"signal": "Donor splice site","matrix": [{"name": "HSF Donor site (matrix GT)","sequence": "CAGGTGAGT","CV": "97.16","position": "288","WT": "true"}]},{"signal": "Donor splice site","matrix": [{"name": "MaxEnt Donor site","sequence": "CAGGTGAGT","CV": "10.67","position": "288","WT": "true"}]}]},"Branch point signals":{"matrix": [{"name": "BP","sequence":

"GTCTCCC","CV": "65.14","position": "7"},{"name": "BP","sequence": "TTCCCAG","CV": "83.11","position": "14"},{"name": "BP","sequence": "CTCCCAC","CV": "87.36","position": "23"},{"name": "BP","sequence": "CGCTGAG","CV": "87.52","position": "50"},{"name": "BP","sequence": "CGCTCGC","CV": "65.56","position": "66"},{"name": "BP","sequence": "CGCGCAC","CV": "80.1","position": "70"},{"name": "BP","sequence": "TGCCGAC","CV": "79.69","position": "104"},{"name": "BP","sequence": "GGCTCAG","CV": "90.83","position": "126"},{"name": "BP","sequence": "AGCCCAG","CV": "79.18","position": "134"},{"name": "BP","sequence": "GGCGGAC","CV": "73.53","position": "144"},{"name": "BP","sequence": "GACCCAT","CV": "78.34","position": "148"},{"name": "BP","sequence": "TCCTCTG","CV": "65.88","position": "156"},{"name": "BP","sequence": "GTGTCAC","CV": "69.04","position": "178"},{"name": "BP","sequence": "CGGTCAC","CV": "69.46","position": "184"},{"name": "BP","sequence": "CTCGCAG","CV": "78.58","position": "190"},{"name": "BP","sequence": "ACCGCAG","CV": "77.7","position": "199"},{"name": "BP","sequence": "TGGTCAC","CV": "67.93","position": "244"},{"name": "BP","sequence": "CTCCTAC","CV": "75.54","position": "254"},{"name": "BP","sequence": "CCCCGAA","CV": "79.18","position": "279"},{"name": "BP","sequence": "CCCGAAC","CV": "72.85","position": "280"},{"name": "BP","sequence": "AACCCAG","CV": "76.89","position": "284"}]}}

SRSF2 Exon 2 HSF Output (JSON):

{"infos": {"HSF Pro version": "4.1.4 - July 2020","Elapsed time": "2,2333s","Sequence":
"CTTGCTCGTCCCGCCCGCAGCCCTAGGCGGCGTCGCCGCAGCCGATCCCGGAGTCGGAG
TCGTTCCAGGTCTCGCAGCCGATCTCGCTACAGCCGCTCGAAGTCTCGGTCCCGCACTCGT
TCTCGATCTCGGTCGACCTCCAAGTCCAGATCCGCACGAAGGTCCAAGTCCAAGTCCTCG
TCGGTCTCCAGATCTCGTTCGCGGTCCAGGTCCCGGTCTCGGTCCAGGAGTCCTCCCCCA
GTGTCCAAGAGGGAATCCAAATCCAGGTCGCGATCGAAGAGTCCCCCCAAGTCTCCTGAA
GAGGAAGGAGCGGTGTCCTCTTAAGAAAATGGTAATGTCTGGGAATCCGA","Exon start":
"21","Exon end": "331","Genomic start": "-20","Genomic end": "331"},"ESE/ESS signals":{"matrix":
[{"name": "IIE (ESS Site)","sequence": "CTTGCT","CV": "NA","position": "1"},{"name": "IIE (ESS
Site)","sequence": "TTGCTC","CV": "NA","position": "2"},{"name": "ESE_SRp40 (ESE
Site)","sequence": "TTGCTCG","CV": "79.07","position": "2"},{"name": "ESE_SRp55 (ESE
Site)","sequence": "CTCGTC","CV": "76.27","position": "5"},{"name": "Sironi_motif3 (ESS
Site)","sequence": "CGTCCCGC","CV": "67.87","position": "7"},{"name": "EIE (ESE Site)","sequence":
"CCCGCC","CV": "NA","position": "10"},{"name": "PESE (ESE Site)","sequence": "CCCGCCCG","CV":
"20.40240730986","position": "10"},{"name": "EIE (ESE Site)","sequence": "CCGCCC","CV":
"NA","position": "11"},{"name": "Sironi_motif3 (ESS Site)","sequence": "CCGCCCGC","CV":
"66.88","position": "11"},{"name": "PESE (ESE Site)","sequence": "CCGCCCGC","CV":
"0.7581947664652","position": "11"},{"name": "ESE_ASF (ESE Site)","sequence": "CGCCCGC","CV":
"79.18","position": "12"},{"name": "ESE_ASFB (ESE Site)","sequence": "CGCCCGC","CV":
"85.77","position": "12"},{"name": "PESS (ESS Site)","sequence": "CGCCCGCA","CV": "-
5.762363178611","position": "12"},{"name": "ESE_SC35 (ESE Site)","sequence": "GCCCGCAG","CV":
"75.37","position": "13"},{"name": "Sironi_motif3 (ESS Site)","sequence": "GCCCGCAG","CV":
"61.3","position": "13"},{"name": "PESE (ESE Site)","sequence": "GCCCGCAG","CV":
"10.83994541661","position": "13"},{"name": "ESE_SRp55 (ESE Site)","sequence": "CGCAGC","CV":
"81.9","position": "16"},{"name": "ESE_ASFB (ESE Site)","sequence": "CGCAGCC","CV":
"74.61","position": "16"},{"name": "ESE_9G8 (ESE Site)","sequence": "GCAGCC","CV":
"63.23","position": "17"},{"name": "EIE (ESE Site)","sequence": "CAGCCC","CV": "NA","position":
"18"},{"name": "ESE_ASF (ESE Site)","sequence": "CAGCCCT","CV": "76.15","position": "18"},{"name":
"ESE_ASFB (ESE Site)","sequence": "CAGCCCT","CV": "77.08","position": "18"},{"name":
"Sironi_motif3 (ESS Site)","sequence": "CAGCCCTA","CV": "60.93","position": "18"},{"name": "EIE
(ESE Site)","sequence": "AGCCCT","CV": "NA","position": "19"},{"name": "EIE (ESE Site)","sequence":
"GCCCTA","CV": "NA","position": "20"},{"name": "EIE (ESE Site)","sequence": "CCCTAG","CV":
"NA","position": "21"},{"name": "ESS_hnRNPA1 (ESS Site)","sequence": "TAGGCG","CV":
"87.51","position": "24"},{"name": "PESE (ESE Site)","sequence": "AGGCGGCG","CV":
"13.92125226567","position": "25"},{"name": "ESE_9G8 (ESE Site)","sequence": "GGCGGC","CV":
"70.34","position": "26"},{"name": "Sironi_motif2 (ESS Site)","sequence": "GGCGGCG","CV":
"67.96","position": "26"},{"name": "PESS (ESS Site)","sequence": "GGCGGCGT","CV": "-
3.528840849609","position": "26"},{"name": "PESE (ESE Site)","sequence": "GCGGCGTC","CV":
"7.381200253838","position": "27"},{"name": "ESE_9G8 (ESE Site)","sequence": "GGCGTC","CV":
"66.92","position": "29"},{"name": "ESE_SRp55 (ESE Site)","sequence": "GGCGTC","CV":
"78.38","position": "29"},{"name": "RESCUE ESE (ESE Site)","sequence": "CGTCGC","CV":
"NA","position": "31"},{"name": "PESE (ESE Site)","sequence": "CGTCGCCG","CV":
"21.75828186761","position": "31"},{"name": "ESE_9G8 (ESE Site)","sequence": "GTCGCC","CV":
"62.62","position": "32"},{"name": "PESE (ESE Site)","sequence": "GTCGCCGC","CV":
"4.922044471358","position": "32"},{"name": "Sironi_motif3 (ESS Site)","sequence":
"TCGCCGCA","CV": "61.29","position": "33"},{"name": "ESE_ASF (ESE Site)","sequence":
"CGCCGCA","CV": "73.47","position": "34"},{"name": "ESE_ASFB (ESE Site)","sequence":
"CGCCGCA","CV": "82.38","position": "34"},{"name": "ESE_SC35 (ESE Site)","sequence":
"CGCCGCAG","CV": "75.37","position": "34"},{"name": "PESE (ESE Site)","sequence":
"GCCGCAGC","CV": "2.9203771065","position": "35"},{"name": "ESE_SRp55 (ESE Site)","sequence":
"CGCAGC","CV": "81.9","position": "37"},{"name": "ESE_ASFB (ESE Site)","sequence":

"CGCAGCC","CV": "74.61","position": "37"},{"name": "PESS (ESS Site)","sequence": "CGCAGCCG","CV": "-2.363344518227","position": "37"},{"name": "ESE_9G8 (ESE Site)","sequence": "GCAGCC","CV": "63.23","position": "38"},{"name": "ESE_ASF (ESE Site)","sequence": "CAGCCGA","CV": "93.16","position": "39"},{"name": "ESE_ASFB (ESE Site)","sequence": "CAGCCGA","CV": "91.54","position": "39"},{"name": "ESE_9G8 (ESE Site)","sequence": "GCCGAT","CV": "68.93","position": "41"},{"name": "ESE_SC35 (ESE Site)","sequence": "CGATCCCG","CV": "75.55","position": "43"},{"name": "ESE_SC35 (ESE Site)","sequence": "GATCCCGG","CV": "79.61","position": "44"},{"name": "Sironi_motif3 (ESS Site)","sequence": "GATCCCGG","CV": "66.76","position": "44"},{"name": "Sironi_motif1 (ESS Site)","sequence": "CCGGAGTC","CV": "61.34","position": "48"},{"name": "ESE_ASFB (ESE Site)","sequence": "CGGAGTC","CV": "72.46","position": "49"},{"name": "ESE_9G8 (ESE Site)","sequence": "GGAGTC","CV": "67.52","position": "50"},{"name": "Sironi_motif2 (ESS Site)","sequence": "GGAGTCG","CV": "60.55","position": "50"},{"name": "Sironi_motif2 (ESS Site)","sequence": "AGTCGGA","CV": "67.68","position": "52"},{"name": "ESE_ASFB (ESE Site)","sequence": "CGGAGTC","CV": "72.46","position": "55"},{"name": "ESE_9G8 (ESE Site)","sequence": "GGAGTC","CV": "67.52","position": "56"},{"name": "Sironi_motif2 (ESS Site)","sequence": "GGAGTCG","CV": "60.55","position": "56"},{"name": "ESE_ASF (ESE Site)","sequence": "GAGTCGT","CV": "73.42","position": "57"},{"name": "Fas ESS (ESS Site)","sequence": "AGTCGT","CV": "NA","position": "58"},{"name": "Fas ESS (ESS Site)","sequence": "GTCGTT","CV": "NA","position": "59"},{"name": "Fas ESS (ESS Site)","sequence": "TCGTTC","CV": "NA","position": "60"},{"name": "Fas ESS (ESS Site)","sequence": "CGTTCC","CV": "NA","position": "61"},{"name": "ESE_SC35 (ESE Site)","sequence": "CGTTCCAG","CV": "75.92","position": "61"},{"name": "Sironi_motif3 (ESS Site)","sequence": "CGTTCCAG","CV": "63.88","position": "61"},{"name": "ESE_SRp40 (ESE Site)","sequence": "TTCCAGG","CV": "83.68","position": "63"},{"name": "ESS_hnRNPA1 (ESS Site)","sequence": "CAGGTC","CV": "71.55","position": "66"},{"name": "ESE_SC35 (ESE Site)","sequence": "GTCTCGCA","CV": "81.88","position": "69"},{"name": "Sironi_motif3 (ESS Site)","sequence": "TCTCGCAG","CV": "84.02","position": "70"},{"name": "ESE_SRp40 (ESE Site)","sequence": "TCGCAGC","CV": "79.19","position": "72"},{"name": "ESE_SRp55 (ESE Site)","sequence": "CGCAGC","CV": "81.9","position": "73"},{"name": "ESE_ASFB (ESE Site)","sequence": "CGCAGCC","CV": "74.61","position": "73"},{"name": "PESS (ESS Site)","sequence": "CGCAGCCG","CV": "-2.363344518227","position": "73"},{"name": "ESE_9G8 (ESE Site)","sequence": "GCAGCC","CV": "63.23","position": "74"},{"name": "ESE_ASF (ESE Site)","sequence": "CAGCCGA","CV": "93.16","position": "75"},{"name": "ESE_ASFB (ESE Site)","sequence": "CAGCCGA","CV": "91.54","position": "75"},{"name": "ESE_9G8 (ESE Site)","sequence": "GCCGAT","CV": "68.93","position": "77"},{"name": "Sironi_motif3 (ESS Site)","sequence": "TCTCGCTA","CV": "78.32","position": "82"},{"name": "EIE (ESE Site)","sequence": "TCGCTA","CV": "NA","position": "84"},{"name": "EIE (ESE Site)","sequence": "CGCTAC","CV": "NA","position": "85"},{"name": "ESE_SC35 (ESE Site)","sequence": "CGCTACAG","CV": "76.53","position": "85"},{"name": "EIE (ESE Site)","sequence": "GCTACA","CV": "NA","position": "86"},{"name": "EIE (ESE Site)","sequence": "CTACAG","CV": "NA","position": "87"},{"name": "ESE_SRp40 (ESE Site)","sequence": "CTACAGC","CV": "90.8","position": "87"},{"name": "Sironi_motif1 (ESS Site)","sequence": "CTACAGCC","CV": "67.96","position": "87"},{"name": "ESE_SRp55 (ESE Site)","sequence": "TACAGC","CV": "83.31","position": "88"},{"name": "ESS_hnRNPA1 (ESS Site)","sequence": "TACAGC","CV": "67.75","position": "88"},{"name": "EIE (ESE Site)","sequence": "TACAGC","CV": "NA","position": "88"},{"name": "ESE_ASF (ESE Site)","sequence": "CAGCCGC","CV": "80.35","position": "90"},{"name": "ESE_ASFB (ESE Site)","sequence": "CAGCCGC","CV": "80.62","position": "90"},{"name": "PESE (ESE Site)","sequence": "GCCGCTCG","CV": "1.884702964342","position": "92"},{"name": "ESE_SRp40 (ESE Site)","sequence": "CCGCTCG","CV": "78.77","position": "93"},{"name": "ESE_ASF (ESE Site)","sequence": "CGCTCGA","CV": "83.49","position": "94"},{"name": "ESE_ASFB (ESE Site)","sequence": "CGCTCGA","CV": "87.08","position": "94"},{"name": "Sironi_motif1 (ESS Site)","sequence": "CTCGAAGT","CV": "67.82","position": "96"},{"name": "ESE_9G8 (ESE Site)","sequence": "GAAGTC","CV": "80.41","position": "99"},{"name": "ESE_SRp40 (ESE Site)","sequence":

"AGTCTCG","CV": "79.07","position": "101"},{"name": "PESE (ESE Site)","sequence": "CGGTCCCG","CV": "9.403605987582","position": "106"},{"name": "Sironi_motif3 (ESS Site)","sequence": "GGTCCCGC","CV": "66.3","position": "107"},{"name": "ESE_SC35 (ESE Site)","sequence": "GTCCCGCA","CV": "83.29","position": "108"},{"name": "Sironi_motif3 (ESS Site)","sequence": "TCCCGCAC","CV": "69.12","position": "109"},{"name": "ESE_ASFB (ESE Site)","sequence": "CGCACTC","CV": "77","position": "112"},{"name": "ESE_SRp40 (ESE Site)","sequence": "GCACTCG","CV": "81.58","position": "113"},{"name": "ESE_ASF (ESE Site)","sequence": "CACTCGT","CV": "84.07","position": "114"},{"name": "ESE_ASFB (ESE Site)","sequence": "CACTCGT","CV": "83.08","position": "114"},{"name": "Fas ESS (ESS Site)","sequence": "TCGTTC","CV": "NA","position": "117"},{"name": "Fas ESS (ESS Site)","sequence": "CGTTCT","CV": "NA","position": "118"},{"name": "ESE_ASF (ESE Site)","sequence": "CGGTCGA","CV": "82.04","position": "130"},{"name": "ESE_ASFB (ESE Site)","sequence": "CGGTCGA","CV": "84.15","position": "130"},{"name": "ESE_9G8 (ESE Site)","sequence": "GTCGAC","CV": "82.02","position": "132"},{"name": "RESCUE ESE (ESE Site)","sequence": "GTCGAC","CV": "NA","position": "132"},{"name": "EIE (ESE Site)","sequence": "TCGACC","CV": "NA","position": "133"},{"name": "EIE (ESE Site)","sequence": "CGACCT","CV": "NA","position": "134"},{"name": "EIE (ESE Site)","sequence": "GACCTC","CV": "NA","position": "135"},{"name": "ESE_SC35 (ESE Site)","sequence": "GACCTCCA","CV": "86","position": "135"},{"name": "Sironi_motif3 (ESS Site)","sequence": "ACCTCCAA","CV": "63.74","position": "136"},{"name": "Sironi_motif3 (ESS Site)","sequence": "CCTCCAAG","CV": "78.21","position": "137"},{"name": "ESE_SRp40 (ESE Site)","sequence": "CTCCAAG","CV": "78.11","position": "138"},{"name": "ESE_9G8 (ESE Site)","sequence": "CAAGTC","CV": "61.08","position": "141"},{"name": "EIE (ESE Site)","sequence": "CCAGAT","CV": "NA","position": "146"},{"name": "Sironi_motif1 (ESS Site)","sequence": "CCAGATCC","CV": "64.62","position": "146"},{"name": "RESCUE ESE (ESE Site)","sequence": "CAGATC","CV": "NA","position": "147"},{"name": "EIE (ESE Site)","sequence": "CAGATC","CV": "NA","position": "147"},{"name": "EIE (ESE Site)","sequence": "AGATCC","CV": "NA","position": "148"},{"name": "ESE_SC35 (ESE Site)","sequence": "GATCCGCA","CV": "81.57","position": "149"},{"name": "ESE_SRp40 (ESE Site)","sequence": "CCGCACG","CV": "80.26","position": "152"},{"name": "ESE_ASF (ESE Site)","sequence": "CGCACGA","CV": "97.76","position": "153"},{"name": "ESE_ASFB (ESE Site)","sequence": "CGCACGA","CV": "99.85","position": "153"},{"name": "ESE_9G8 (ESE Site)","sequence": "CACGAA","CV": "67.79","position": "155"},{"name": "RESCUE ESE (ESE Site)","sequence": "ACGAAG","CV": "NA","position": "156"},{"name": "ESE_ASF (ESE Site)","sequence": "CGAAGGT","CV": "78.72","position": "157"},{"name": "ESE_ASFB (ESE Site)","sequence": "CGAAGGT","CV": "82.92","position": "157"},{"name": "Sironi_motif2 (ESS Site)","sequence": "CGAAGGT","CV": "60.36","position": "157"},{"name": "ESE_9G8 (ESE Site)","sequence": "GAAGGT","CV": "70.74","position": "158"},{"name": "ESS_hnRNPA1 (ESS Site)","sequence": "GAAGGT","CV": "70.84","position": "158"},{"name": "ESS_hnRNPA1 (ESS Site)","sequence": "AAGGTC","CV": "71.55","position": "159"},{"name": "ESE_SC35 (ESE Site)","sequence": "GGTCCAAG","CV": "82","position": "161"},{"name": "Sironi_motif3 (ESS Site)","sequence": "GGTCCAAG","CV": "62.3","position": "161"},{"name": "ESE_9G8 (ESE Site)","sequence": "CAAGTC","CV": "61.08","position": "165"},{"name": "Sironi_motif3 (ESS Site)","sequence": "AGTCCAAG","CV": "61.03","position": "167"},{"name": "ESE_9G8 (ESE Site)","sequence": "CAAGTC","CV": "61.08","position": "171"},{"name": "ESE_SRp55 (ESE Site)","sequence": "CTCGTC","CV": "76.27","position": "177"},{"name": "RESCUE ESE (ESE Site)","sequence": "TCGTCG","CV": "NA","position": "178"},{"name": "ESE_ASFB (ESE Site)","sequence": "CGTCGGT","CV": "77.38","position": "179"},{"name": "Sironi_motif2 (ESS Site)","sequence": "CGTCGGT","CV": "63.41","position": "179"},{"name": "ESE_SC35 (ESE Site)","sequence": "GGTCTCCA","CV": "84.71","position": "183"},{"name": "ESE_SC35 (ESE Site)","sequence": "GTCTCCAG","CV": "87.9","position": "184"},{"name": "ESE_SRp40 (ESE Site)","sequence": "TCTCCAG","CV": "79.78","position": "185"},{"name": "Sironi_motif3 (ESS Site)","sequence": "TCTCCAGA","CV": "74.12","position": "185"},{"name": "ESE_ASF (ESE Site)","sequence": "CTCCAGA","CV": "73.42","position": "186"},{"name": "ESE_ASFB (ESE Site)","sequence": "CTCCAGA","CV":

"76.46","position": "186"},{"name": "EIE (ESE Site)","sequence": "CCAGAT","CV": "NA","position": "188"},{"name": "Sironi_motif1 (ESS Site)","sequence": "CCAGATCT","CV": "71.12","position": "188"},{"name": "RESCUE ESE (ESE Site)","sequence": "CAGATC","CV": "NA","position": "189"},{"name": "EIE (ESE Site)","sequence": "CAGATC","CV": "NA","position": "189"},{"name": "Fas ESS (ESS Site)","sequence": "TCGTTC","CV": "NA","position": "195"},{"name": "Fas ESS (ESS Site)","sequence": "CGTTCG","CV": "NA","position": "196"},{"name": "PESE (ESE Site)","sequence": "CGCGGTCC","CV": "10.84160448612","position": "200"},{"name": "ESE_ASFB (ESE Site)","sequence": "CGGTCCA","CV": "71.46","position": "202"},{"name": "ESS_hnRNPA1 (ESS Site)","sequence": "CAGGTC","CV": "71.55","position": "207"},{"name": "ESE_SC35 (ESE Site)","sequence": "GGTCCCGG","CV": "81.82","position": "209"},{"name": "Sironi_motif3 (ESS Site)","sequence": "GGTCCCGG","CV": "65.87","position": "209"},{"name": "ESE_ASF (ESE Site)","sequence": "GTCCCGG","CV": "74.64","position": "210"},{"name": "ESE_SC35 (ESE Site)","sequence": "GGTCTCGG","CV": "77.15","position": "215"},{"name": "ESE_ASFB (ESE Site)","sequence": "CGGTCCA","CV": "71.46","position": "220"},{"name": "PESE (ESE Site)","sequence": "TCCAGGAG","CV": "43.95870575987","position": "223"},{"name": "Sironi_motif1 (ESS Site)","sequence": "CCAGGAGT","CV": "64.47","position": "224"},{"name": "ESE_9G8 (ESE Site)","sequence": "CAGGAG","CV": "60.54","position": "225"},{"name": "ESS_hnRNPA1 (ESS Site)","sequence": "CAGGAG","CV": "73.94","position": "225"},{"name": "Sironi_motif1 (ESS Site)","sequence": "CAGGAGTC","CV": "63.55","position": "225"},{"name": "PESE (ESE Site)","sequence": "AGGAGTCC","CV": "41.70859273576","position": "226"},{"name": "ESE_9G8 (ESE Site)","sequence": "GGAGTC","CV": "67.52","position": "227"},{"name": "Sironi_motif3 (ESS Site)","sequence": "CCTCCCCC","CV": "84.7","position": "232"},{"name": "ESE_SC35 (ESE Site)","sequence": "CTCCCCCA","CV": "76.41","position": "233"},{"name": "Sironi_motif3 (ESS Site)","sequence": "CTCCCCCA","CV": "60.11","position": "233"},{"name": "Sironi_motif3 (ESS Site)","sequence": "TCCCCCAG","CV": "85.88","position": "234"},{"name": "EIE (ESE Site)","sequence": "CCCCCA","CV": "NA","position": "235"},{"name": "ESE_ASFB (ESE Site)","sequence": "CCCCCAG","CV": "72.23","position": "235"},{"name": "EIE (ESE Site)","sequence": "CCCCAG","CV": "NA","position": "236"},{"name": "ESE_ASFB (ESE Site)","sequence": "CCCCAGT","CV": "72.77","position": "236"},{"name": "ESE_ASFB (ESE Site)","sequence": "CCCAGTG","CV": "75.54","position": "237"},{"name": "EIE (ESE Site)","sequence": "CCAGTG","CV": "NA","position": "238"},{"name": "Sironi_motif1 (ESS Site)","sequence": "CCAGTGTC","CV": "61.34","position": "238"},{"name": "ESS_hnRNPA1 (ESS Site)","sequence": "CAGTGT","CV": "68.94","position": "239"},{"name": "IIE (ESS Site)","sequence": "CAGTGT","CV": "NA","position": "239"},{"name": "ESE_SRp55 (ESE Site)","sequence": "AGTGTC","CV": "74.35","position": "240"},{"name": "IIE (ESS Site)","sequence": "GTGTCC","CV": "NA","position": "241"},{"name": "Sironi_motif3 (ESS Site)","sequence": "TGTCCAAG","CV": "69.69","position": "242"},{"name": "EIE (ESE Site)","sequence": "CCAAGA","CV": "NA","position": "245"},{"name": "ESE_9G8 (ESE Site)","sequence": "CAAGAG","CV": "65.37","position": "246"},{"name": "EIE (ESE Site)","sequence": "CAAGAG","CV": "NA","position": "246"},{"name": "Sironi_motif1 (ESS Site)","sequence": "CAAGAGGG","CV": "90.77","position": "246"},{"name": "ESE_Tra2 (ESE Site)","sequence": "AAGAG","CV": "89.41","position": "247"},{"name": "ESS_hnRNPA1 (ESS Site)","sequence": "AAGAGG","CV": "70.84","position": "247"},{"name": "RESCUE ESE (ESE Site)","sequence": "AAGAGG","CV": "NA","position": "247"},{"name": "EIE (ESE Site)","sequence": "AAGAGG","CV": "NA","position": "247"},{"name": "ESE_ASF (ESE Site)","sequence": "AAGAGGG","CV": "76.5","position": "247"},{"name": "Sironi_motif2 (ESS Site)","sequence": "AGAGGGA","CV": "79.77","position": "248"},{"name": "ESE_9G8 (ESE Site)","sequence": "GAGGGA","CV": "66.92","position": "249"},{"name": "ESS_hnRNPA1 (ESS Site)","sequence": "GAGGGA","CV": "88.93","position": "249"},{"name": "IIE (ESS Site)","sequence": "GAGGGA","CV": "NA","position": "249"},{"name": "EIE (ESE Site)","sequence": "GGGAAT","CV": "NA","position": "251"},{"name": "IIE (ESS Site)","sequence": "GGGAAT","CV": "NA","position": "251"},{"name": "ESE_9G8 (ESE Site)","sequence": "GAATCC","CV": "59.87","position": "253"},{"name": "ESE_SC35 (ESE Site)","sequence": "GAATCCAA","CV": "80.71","position": "253"},{"name": "RESCUE ESE (ESE Site)","sequence": "AATCCA","CV": "NA","position": "254"},{"name": "Sironi_motif3 (ESS

Site)","sequence": "AATCCAAA","CV": "65.63","position": "254"},{"name": "RESCUE ESE (ESE Site)","sequence": "ATCCAA","CV": "NA","position": "255"},{"name": "RESCUE ESE (ESE Site)","sequence": "AAATCC","CV": "NA","position": "259"},{"name": "RESCUE ESE (ESE Site)","sequence": "AATCCA","CV": "NA","position": "260"},{"name": "Sironi_motif1 (ESS Site)","sequence": "ATCCAGGT","CV": "60.99","position": "261"},{"name": "ESS_hnRNPA1 (ESS Site)","sequence": "CAGGTC","CV": "71.55","position": "264"},{"name": "Sironi_motif2 (ESS Site)","sequence": "GGTCGCG","CV": "65.36","position": "266"},{"name": "ESE_ASFB (ESE Site)","sequence": "CGATCGA","CV": "73.54","position": "271"},{"name": "EIE (ESE Site)","sequence": "GATCGA","CV": "NA","position": "272"},{"name": "RESCUE ESE (ESE Site)","sequence": "CGAAGA","CV": "NA","position": "275"},{"name": "ESE_9G8 (ESE Site)","sequence": "GAAGAG","CV": "84.7","position": "276"},{"name": "RESCUE ESE (ESE Site)","sequence": "GAAGAG","CV": "NA","position": "276"},{"name": "EIE (ESE Site)","sequence": "GAAGAG","CV": "NA","position": "276"},{"name": "Sironi_motif1 (ESS Site)","sequence": "GAAGAGTC","CV": "69.7","position": "276"},{"name": "ESE_Tra2 (ESE Site)","sequence": "AAGAG","CV": "89.41","position": "277"},{"name": "ESS_hnRNPA1 (ESS Site)","sequence": "AAGAGT","CV": "72.74","position": "277"},{"name": "Sironi_motif3 (ESS Site)","sequence": "AGTCCCCC","CV": "67.53","position": "280"},{"name": "ESE_SC35 (ESE Site)","sequence": "GTCCCCCC","CV": "77.76","position": "281"},{"name": "Sironi_motif3 (ESS Site)","sequence": "TCCCCCCA","CV": "78.48","position": "282"},{"name": "EIE (ESE Site)","sequence": "CCCCCC","CV": "NA","position": "283"},{"name": "ESE_ASFB (ESE Site)","sequence": "CCCCCCA","CV": "76.54","position": "283"},{"name": "Sironi_motif3 (ESS Site)","sequence": "CCCCCCAA","CV": "83.78","position": "283"},{"name": "EIE (ESE Site)","sequence": "CCCCCA","CV": "NA","position": "284"},{"name": "ESE_ASFB (ESE Site)","sequence": "CCCCCAA","CV": "75.54","position": "284"},{"name": "Sironi_motif3 (ESS Site)","sequence": "CCCCCAAG","CV": "62.88","position": "284"},{"name": "EIE (ESE Site)","sequence": "CCCCAA","CV": "NA","position": "285"},{"name": "ESE_SRp40 (ESE Site)","sequence": "CCCCAAG","CV": "79.96","position": "285"},{"name": "EIE (ESE Site)","sequence": "CCCAAG","CV": "NA","position": "286"},{"name": "ESE_ASF (ESE Site)","sequence": "CCCAAGT","CV": "73.65","position": "286"},{"name": "ESE_ASFB (ESE Site)","sequence": "CCCAAGT","CV": "75.92","position": "286"},{"name": "ESE_9G8 (ESE Site)","sequence": "CAAGTC","CV": "61.08","position": "288"},{"name": "ESE_SC35 (ESE Site)","sequence": "GTCTCCTG","CV": "94.17","position": "291"},{"name": "Sironi_motif3 (ESS Site)","sequence": "TCTCCTGA","CV": "74.12","position": "292"},{"name": "ESE_ASF (ESE Site)","sequence": "CTCCTGA","CV": "73.42","position": "293"},{"name": "ESE_ASFB (ESE Site)","sequence": "CTCCTGA","CV": "77.23","position": "293"},{"name": "EIE (ESE Site)","sequence": "TCCTGA","CV": "NA","position": "294"},{"name": "PESE (ESE Site)","sequence": "TCCTGAAG","CV": "42.05741210042","position": "294"},{"name": "RESCUE ESE (ESE Site)","sequence": "CCTGAA","CV": "NA","position": "295"},{"name": "EIE (ESE Site)","sequence": "CCTGAA","CV": "NA","position": "295"},{"name": "PESE (ESE Site)","sequence": "CCTGAAGA","CV": "64.8679588053","position": "295"},{"name": "RESCUE ESE (ESE Site)","sequence": "CTGAAG","CV": "NA","position": "296"},{"name": "EIE (ESE Site)","sequence": "CTGAAG","CV": "NA","position": "296"},{"name": "ESE_ASF (ESE Site)","sequence": "CTGAAGA","CV": "77.73","position": "296"},{"name": "ESE_ASFB (ESE Site)","sequence": "CTGAAGA","CV": "76.69","position": "296"},{"name": "PESE (ESE Site)","sequence": "CTGAAGAG","CV": "77.07829563789","position": "296"},{"name": "RESCUE ESE (ESE Site)","sequence": "TGAAGA","CV": "NA","position": "297"},{"name": "EIE (ESE Site)","sequence": "TGAAGA","CV": "NA","position": "297"},{"name": "Sironi_motif2 (ESS Site)","sequence": "TGAAGAG","CV": "65.39","position": "297"},{"name": "PESE (ESE Site)","sequence": "TGAAGAGG","CV": "45.09516837482","position": "297"},{"name": "ESE_9G8 (ESE Site)","sequence": "GAAGAG","CV": "84.7","position": "298"},{"name": "RESCUE ESE (ESE Site)","sequence": "GAAGAG","CV": "NA","position": "298"},{"name": "EIE (ESE Site)","sequence": "GAAGAG","CV": "NA","position": "298"},{"name": "Sironi_motif2 (ESS Site)","sequence": "GAAGAGG","CV": "60.55","position": "298"},{"name": "Sironi_motif1 (ESS Site)","sequence": "GAAGAGGA","CV": "81.26","position": "298"},{"name": "PESE (ESE Site)","sequence": "GAAGAGGA","CV": "70.03347172738","position": "298"},{"name": "ESE_Tra2 (ESE Site)","sequence":

"AAGAG","CV": "89.41","position": "299"},{"name": "ESS_hnRNPA1 (ESS Site)","sequence": "AAGAGG","CV": "70.84","position": "299"},{"name": "RESCUE ESE (ESE Site)","sequence": "AAGAGG","CV": "NA","position": "299"},{"name": "EIE (ESE Site)","sequence": "AAGAGG","CV": "NA","position": "299"},{"name": "ESE_ASF (ESE Site)","sequence": "AAGAGGA","CV": "80.75","position": "299"},{"name": "PESE (ESE Site)","sequence": "AAGAGGAA","CV": "37.18970215555","position": "299"},{"name": "RESCUE ESE (ESE Site)","sequence": "AGAGGA","CV": "NA","position": "300"},{"name": "EIE (ESE Site)","sequence": "AGAGGA","CV": "NA","position": "300"},{"name": "Sironi_motif2 (ESS Site)","sequence": "AGAGGAA","CV": "62.87","position": "300"},{"name": "PESE (ESE Site)","sequence": "AGAGGAAG","CV": "34.37757933463","position": "300"},{"name": "ESE_9G8 (ESE Site)","sequence": "GAGGAA","CV": "82.89","position": "301"},{"name": "ESS_hnRNPA1 (ESS Site)","sequence": "GAGGAA","CV": "80.6","position": "301"},{"name": "RESCUE ESE (ESE Site)","sequence": "GAGGAA","CV": "NA","position": "301"},{"name": "EIE (ESE Site)","sequence": "GAGGAA","CV": "NA","position": "301"},{"name": "RESCUE ESE (ESE Site)","sequence": "AGGAAG","CV": "NA","position": "302"},{"name": "EIE (ESE Site)","sequence": "AGGAAG","CV": "NA","position": "302"},{"name": "PESE (ESE Site)","sequence": "AGGAAGGA","CV": "34.85041414523","position": "302"},{"name": "Sironi_motif2 (ESS Site)","sequence": "GGAAGGA","CV": "68.38","position": "303"},{"name": "ESE_9G8 (ESE Site)","sequence": "GAAGGA","CV": "71.75","position": "304"},{"name": "ESS_hnRNPA1 (ESS Site)","sequence": "GAAGGA","CV": "71.79","position": "304"},{"name": "RESCUE ESE (ESE Site)","sequence": "GAAGGA","CV": "NA","position": "304"},{"name": "EIE (ESE Site)","sequence": "GAAGGA","CV": "NA","position": "304"},{"name": "Sironi_motif2 (ESS Site)","sequence": "GAAGGAG","CV": "60.55","position": "304"},{"name": "PESE (ESE Site)","sequence": "GAAGGAGC","CV": "72.20643801924","position": "304"},{"name": "ESS_hnRNPA1 (ESS Site)","sequence": "AAGGAG","CV": "73.94","position": "305"},{"name": "RESCUE ESE (ESE Site)","sequence": "AAGGAG","CV": "NA","position": "305"},{"name": "EIE (ESE Site)","sequence": "AAGGAG","CV": "NA","position": "305"},{"name": "EIE (ESE Site)","sequence": "AGGAGC","CV": "NA","position": "306"},{"name": "EIE (ESE Site)","sequence": "GGAGCG","CV": "NA","position": "307"},{"name": "Sironi_motif2 (ESS Site)","sequence": "GGAGCGG","CV": "77.46","position": "307"},{"name": "Sironi_motif1 (ESS Site)","sequence": "GGAGCGGT","CV": "72.34","position": "307"},{"name": "ESS_hnRNPA1 (ESS Site)","sequence": "GAGCGG","CV": "70.84","position": "308"},{"name": "EIE (ESE Site)","sequence": "GAGCGG","CV": "NA","position": "308"},{"name": "ESE_ASF (ESE Site)","sequence": "GAGCGGT","CV": "78.37","position": "308"},{"name": "Sironi_motif2 (ESS Site)","sequence": "AGCGGTG","CV": "64.21","position": "309"},{"name": "ESE_9G8 (ESE Site)","sequence": "GGTGTC","CV": "63.29","position": "312"},{"name": "IIE (ESS Site)","sequence": "GGTGTC","CV": "NA","position": "312"},{"name": "IIE (ESS Site)","sequence": "GTGTCC","CV": "NA","position": "313"},{"name": "IIE (ESS Site)","sequence": "TGTCCT","CV": "NA","position": "314"},{"name": "ESE_SC35 (ESE Site)","sequence": "GTCCTCTT","CV": "77.03","position": "315"},{"name": "IIE (ESS Site)","sequence": "CCTCTT","CV": "NA","position": "317"},{"name": "Sironi_motif3 (ESS Site)","sequence": "CCTCTTAA","CV": "64.74","position": "317"},{"name": "ESE_9G8 (ESE Site)","sequence": "TAAGAA","CV": "65.98","position": "322"},{"name": "ESS_hnRNPA1 (ESS Site)","sequence": "TAAGAA","CV": "74.89","position": "322"},{"name": "EIE (ESE Site)","sequence": "TAAGAA","CV": "NA","position": "322"},{"name": "ESE_Tra2 (ESE Site)","sequence": "AAGAA","CV": "100.34","position": "323"},{"name": "ESS_hnRNPA1 (ESS Site)","sequence": "AAGAAA","CV": "65.36","position": "323"},{"name": "RESCUE ESE (ESE Site)","sequence": "AAGAAA","CV": "NA","position": "323"},{"name": "EIE (ESE Site)","sequence": "AAGAAA","CV": "NA","position": "323"},{"name": "RESCUE ESE (ESE Site)","sequence": "AGAAAA","CV": "NA","position": "324"},{"name": "EIE (ESE Site)","sequence": "AGAAAA","CV": "NA","position": "324"},{"name": "ESE_9G8 (ESE Site)","sequence": "GAAAAT","CV": "66.18","position": "325"},{"name": "RESCUE ESE (ESE Site)","sequence": "GAAAAT","CV": "NA","position": "325"},{"name": "EIE (ESE Site)","sequence": "GAAAAT","CV": "NA","position": "325"},{"name": "ESE_Tra2 (ESE Site)","sequence": "AAAAT","CV": "81.36","position": "326"},{"name": "EIE (ESE Site)","sequence": "AAAATG","CV": "NA","position": "326"},{"name": "PESS (ESS Site)","sequence": "AAAATGGT","CV": "-32.15318188794","position": "326"},{"name": "EIE (ESE

Site)","sequence": "AAATGG","CV": "NA","position": "327"},{"name": "PESS (ESS Site)","sequence": "AAATGGTA","CV": "-29.37921766577","position": "327"},{"name": "IIE (ESS Site)","sequence": "GTAATG","CV": "NA","position": "332"},{"name": "ESS_hnRNPA1 (ESS Site)","sequence": "TAATGT","CV": "67.03","position": "333"},{"name": "IIE (ESS Site)","sequence": "TAATGT","CV": "NA","position": "333"},{"name": "PESS (ESS Site)","sequence": "TAATGTCT","CV": "-32.21747083148","position": "333"},{"name": "IIE (ESS Site)","sequence": "ATGTCT","CV": "NA","position": "335"},{"name": "IIE (ESS Site)","sequence": "TGTCTG","CV": "NA","position": "336"},{"name": "ESE_SRp40 (ESE Site)","sequence": "TGTCTGG","CV": "87.21","position": "336"},{"name": "Sironi_motif2 (ESS Site)","sequence": "TGTCTGG","CV": "68.44","position": "336"},{"name": "IIE (ESS Site)","sequence": "GTCTGG","CV": "NA","position": "337"},{"name": "Sironi_motif2 (ESS Site)","sequence": "GTCTGGG","CV": "60.01","position": "337"},{"name": "IIE (ESS Site)","sequence": "TCTGGG","CV": "NA","position": "338"},{"name": "Sironi_motif2 (ESS Site)","sequence": "TCTGGGA","CV": "72.75","position": "338"},{"name": "IIE (ESS Site)","sequence": "CTGGGA","CV": "NA","position": "339"},{"name": "EIE (ESE Site)","sequence": "TGGGAA","CV": "NA","position": "340"},{"name": "IIE (ESS Site)","sequence": "TGGGAA","CV": "NA","position": "340"},{"name": "EIE (ESE Site)","sequence": "GGGAAT","CV": "NA","position": "341"},{"name": "IIE (ESS Site)","sequence": "GGGAAT","CV": "NA","position": "341"},{"name": "ESE_9G8 (ESE Site)","sequence": "GAATCC","CV": "59.87","position": "343"}]},"Splice site signals":{"matrix": [{"signal": "Acceptor splice site","matrix": [{"name": "MaxEnt Acceptor site","sequence": "CTTGCTCGTCCCGCCCGCAGCCC","CV": "10.08","position": "1","WT": "true"}]},{"signal": "Acceptor splice site","matrix": [{"name": "HSF Acceptor site (matrix AG)","sequence": "TCCCGCCCGCAGCC","CV": "80.01","position": "9","WT": "true"}]},{"signal": "Acceptor splice site","matrix": [{"name": "HSF Acceptor site (matrix AG)","sequence": "CCGCAGCCCTAGGC","CV": "73.06","position": "15"}]},{"signal": "Acceptor splice site","matrix": [{"name": "HSF Acceptor site (matrix AG)","sequence": "GCGTCGCCGCAGCC","CV": "76.03","position": "30"}]},{"signal": "Acceptor splice site","matrix": [{"name": "HSF Acceptor site (matrix AG)","sequence": "CCGATCCCGGAGTC","CV": "69.52","position": "42"}]},{"signal": "Acceptor splice site","matrix": [{"name": "HSF Acceptor site (matrix AG)","sequence": "CCGGAGTCGGAGTC","CV": "66.09","position": "48"}]},{"signal": "Donor splice site","matrix": [{"name": "HSF Donor site (matrix GT)","sequence": "GGAGTCGGA","CV": "66.61","position": "50"}]},{"signal": "Acceptor splice site","matrix": [{"name": "HSF Acceptor site (matrix AG)","sequence": "GAGTCGTTCCAGGT","CV": "82.85","position": "57"}]},{"signal": "Donor splice site","matrix": [{"name": "HSF Donor site (matrix GT)","sequence": "CAGGTCTCG","CV": "73.98","position": "66"}]},{"signal": "Acceptor splice site","matrix": [{"name": "HSF Acceptor site (matrix AG)","sequence": "CAGGTCTCGCAGCC","CV": "77.38","position": "66"}]},{"signal": "Acceptor splice site","matrix": [{"name": "MaxEnt Acceptor site","sequence": "CGCAGCCGATCTCGCTACAGCCG","CV": "5.32","position": "73"}]},{"signal": "Acceptor splice site","matrix": [{"name": "HSF Acceptor site (matrix AG)","sequence": "ATCTCGCTACAGCC","CV": "83.29","position": "81"}]},{"signal": "Acceptor splice site","matrix": [{"name": "HSF Acceptor site (matrix AG)","sequence": "AGCCGCTCGAAGTC","CV": "68.62","position": "91"}]},{"signal": "Donor splice site","matrix": [{"name": "HSF Donor site (matrix GT)","sequence": "TCGGTCCCG","CV": "65.77","position": "105"}]},{"signal": "Donor splice site","matrix": [{"name": "HSF Donor site (matrix GT)","sequence": "TCGGTCGAC","CV": "66.41","position": "129"}]},{"signal": "Acceptor splice site","matrix": [{"name": "HSF Acceptor site (matrix AG)","sequence": "TCGACCTCCAAGTC","CV": "73.09","position": "133"}]},{"signal": "Acceptor splice site","matrix": [{"name": "HSF Acceptor site (matrix AG)","sequence": "CTCCAAGTCCAGAT","CV": "81.37","position": "138"}]},{"signal": "Acceptor splice site","matrix": [{"name": "HSF Acceptor site (matrix AG)","sequence": "ATCCGCACGAAGGT","CV": "73.63","position": "150"}]},{"signal": "Acceptor splice site","matrix": [{"name": "HSF Acceptor site (matrix AG)","sequence": "CGAAGGTCCAAGTC","CV": "65.1","position": "157"}]},{"signal": "Donor splice site","matrix": [{"name": "HSF Donor site (matrix GT)","sequence": "AAGGTCCAA","CV": "73.68","position": "159"}]},{"signal": "Acceptor splice site","matrix": [{"name": "HSF Acceptor site (matrix AG)","sequence": "TCCAAGTCCAAGTC","CV": "70.91","position": "163"}]},{"signal": "Acceptor splice site","matrix": [{"name": "MaxEnt Acceptor site","sequence":

"AAGTCCTCGTCGGTCTCCAGATC","CV": "5.25","position": "172"}]},{"signal": "Donor splice site","matrix": [{"name": "HSF Donor site (matrix GT)","sequence": "CTCGTCGGT","CV": "69.67","position": "177"}]},{"signal": "Acceptor splice site","matrix": [{"name": "HSF Acceptor site (matrix AG)","sequence": "GTCGGTCTCCAGAT","CV": "83.6","position": "180"}]},{"signal": "Donor splice site","matrix": [{"name": "HSF Donor site (matrix GT)","sequence": "TCGGTCTCC","CV": "65.97","position": "181"}]},{"signal": "Acceptor splice site","matrix": [{"name": "MaxEnt Acceptor site","sequence": "AGATCTCGTTCGCGGTCCAGGTC","CV": "4.74","position": "190"}]},{"signal": "Donor splice site","matrix": [{"name": "HSF Donor site (matrix GT)","sequence": "CTCGTTCGC","CV": "66.24","position": "194"}]},{"signal": "Acceptor splice site","matrix": [{"name": "HSF Acceptor site (matrix AG)","sequence": "TTCGCGGTCCAGGT","CV": "85.59","position": "198"}]},{"signal": "Donor splice site","matrix": [{"name": "HSF Donor site (matrix GT)","sequence": "GCGGTCCAG","CV": "66.61","position": "201"}]},{"signal": "Donor splice site","matrix": [{"name": "HSF Donor site (matrix GT)","sequence": "CAGGTCCCG","CV": "73.45","position": "207"}]},{"signal": "Acceptor splice site","matrix": [{"name": "MaxEnt Acceptor site","sequence": "AGGTCCCGGTCTCGGTCCAGGAG","CV": "5.29","position": "208"}]},{"signal": "Donor splice site","matrix": [{"name": "HSF Donor site (matrix GT)","sequence": "CCGGTCTCG","CV": "67.8","position": "213"}]},{"signal": "Acceptor splice site","matrix": [{"name": "HSF Acceptor site (matrix AG)","sequence": "GTCTCGGTCCAGGA","CV": "84.31","position": "216"}]},{"signal": "Donor splice site","matrix": [{"name": "HSF Donor site (matrix GT)","sequence": "TCGGTCCAG","CV": "66.22","position": "219"}]},{"signal": "Acceptor splice site","matrix": [{"name": "HSF Acceptor site (matrix AG)","sequence": "TCGGTCCAGGAGTC","CV": "69.89","position": "219"}]},{"signal": "Acceptor splice site","matrix": [{"name": "HSF Acceptor site (matrix AG)","sequence": "GTCCTCCCCCAGTG","CV": "82.21","position": "230"}]},{"signal": "Acceptor splice site","matrix": [{"name": "HSF Acceptor site (matrix AG)","sequence": "CCAGTGTCCAAGAG","CV": "71.26","position": "238"}]},{"signal": "Acceptor splice site","matrix": [{"name": "HSF Acceptor site (matrix AG)","sequence": "AGTGTCCAAGAGGG","CV": "71.6","position": "240"}]},{"signal": "Acceptor splice site","matrix": [{"name": "HSF Acceptor site (matrix AG)","sequence": "ATCCAAATCCAGGT","CV": "81.76","position": "255"}]},{"signal": "Donor splice site","matrix": [{"name": "HSF Donor site (matrix GT)","sequence": "CAGGTCGCG","CV": "73.98","position": "264"}]},{"signal": "Acceptor splice site","matrix": [{"name": "HSF Acceptor site (matrix AG)","sequence": "TCGCGATCGAAGAG","CV": "72.08","position": "268"}]},{"signal": "Acceptor splice site","matrix": [{"name": "HSF Acceptor site (matrix AG)","sequence": "AGTCCCCCCAAGTC","CV": "71.57","position": "280"}]},{"signal": "Acceptor splice site","matrix": [{"name": "HSF Acceptor site (matrix AG)","sequence": "AGTCTCCTGAAGAG","CV": "76.37","position": "290"}]},{"signal": "Acceptor splice site","matrix": [{"name": "HSF Acceptor site (matrix AG)","sequence": "TCTCCTGAAGAGGA","CV": "76.54","position": "292"}]},{"signal": "Acceptor splice site","matrix": [{"name": "HSF Acceptor site (matrix AG)","sequence": "CTGAAGAGGAAGGA","CV": "66.9","position": "296"}]},{"signal": "Donor splice site","matrix": [{"name": "HSF Donor site (matrix GT)","sequence": "GCGGTGTCC","CV": "69.73","position": "310"}]},{"signal": "Acceptor splice site","matrix": [{"name": "HSF Acceptor site (matrix AG)","sequence": "TGTCCTCTTAAGAA","CV": "81.06","position": "314"}]},{"signal": "Donor splice site","matrix": [{"name": "HSF Donor site (matrix GT)","sequence": "ATGGTAATG","CV": "81.8","position": "329","WT": "true"}]},{"signal": "Donor splice site","matrix": [{"name": "MaxEnt Donor site","sequence": "ATGGTAATG","CV": "5.46","position": "329","WT": "true"}]},{"signal": "Donor splice site","matrix": [{"name": "HSF Donor site (matrix GT)","sequence": "AATGTCTGG","CV": "73.34","position": "334"}]}]},"Branch point signals":{"matrix": [{"name": "BP","sequence": "CCCGCAG","CV": "81.95","position": "14"},{"name": "BP","sequence": "GCCCTAG","CV": "74.55","position": "20"},{"name": "BP","sequence": "GCCGCAG","CV": "80.32","position": "35"},{"name": "BP","sequence": "AGCCGAT","CV": "73.07","position": "40"},{"name": "BP","sequence": "CCCGGAG","CV": "77.01","position": "47"},{"name": "BP","sequence": "GTCGGAG","CV": "72.02","position": "53"},{"name": "BP","sequence": "GTCTCGC","CV": "65.14","position": "69"},{"name": "BP","sequence": "CTCGCAG","CV": "78.58","position": "71"},{"name": "BP","sequence": "AGCCGAT","CV": "73.07","position": "76"},{"name": "BP","sequence":

"CTCGAAG","CV": "66.76","position": "96"},{"name": "BP","sequence": "CCCGCAC","CV": "84.67","position": "110"},{"name": "BP","sequence": "CTCCAAG","CV": "72.81","position": "138"},{"name": "BP","sequence": "TCCAGAT","CV": "74.32","position": "145"},{"name": "BP","sequence": "TCCGCAC","CV": "83.15","position": "151"},{"name": "BP","sequence": "GTCCAAG","CV": "71.18","position": "162"},{"name": "BP","sequence": "GTCCAAG","CV": "71.18","position": "168"},{"name": "BP","sequence": "TCCAGAT","CV": "74.32","position": "187"},{"name": "BP","sequence": "TCCTCCC","CV": "68.61","position": "231"},{"name": "BP","sequence": "CCCCCAG","CV": "88","position": "235"},{"name": "BP","sequence": "GTCCAAG","CV": "71.18","position": "243"},{"name": "BP","sequence": "TCCAAAT","CV": "67.43","position": "256"},{"name": "BP","sequence": "CCCCCAA","CV": "84.11","position": "284"},{"name": "BP","sequence": "CCCCAAG","CV": "76.18","position": "285"},{"name": "BP","sequence": "TCCTGAA","CV": "86.68","position": "294"},{"name": "BP","sequence": "CTCTTAA","CV": "77.95","position": "318"}]}}

USH2A Exon 13 HSF Output (JSON):

{"infos": {"HSF Pro version": "4.1.4 - July 2020","Elapsed time": "4,4s","Sequence": "TAAATATATTTTATCTTTAGGGCTTAGGTGTGATCATTGCAATTTTGGATTTAAATTTCTC
CGAAGCTTTAATGATGTTGGATGTGAGCCCTGCCAGTGTAACCTCCATGGCTCAGTGAAC
AAATTCTGCAATCCTCACTCTGGGCAGTGTGAGTGCAAAAAAGAAGCCAAAGGACTTCAG
TGTGACACCTGCAGAGAAAACTTTTATGGGTTAGATGTCACCAATTGTAAGGCCTGTGAC
TGTGACACAGCTGGATCCCTCCCTGGGACTGTCTGTAATGCTAAGACAGGGCAGTGCATC
TGCAAGCCCAATGTTGAAGGGAGACAGTGCAATAAATGTTTGGAGGGAAACTTCTACCT
ACGGCAAAATAATTCTTTCCTCTGTCTGCCTTGCAACTGTGATAAGACTGGGACAATAAAT
GGCTCTCTGCTGTGTAACAAATCAACAGGACAATGTCCTTGCAAATTAGGGGTAACAGGT
CTTCGCTGTAATCAGTGTGAGCCTCACAGGTACAATTTGACCATTGACAATTTTCAACACT
GCCAGATGTGTGAGTGTGATTCCTTGGGGACATTACCTGGGACCATTTGTGACCCAATCA
GTGGCCAGTGCCTGTGTGTGCCTAATCGTCAAGGAAGAAGGTGTAATCAGTGTCAACCA
GGTAAGAAAGAAATGTATTA","Exon start": "21","Exon end": "662","Genomic start": "-20","Genomic end": "662"},"ESE/ESS signals":{"matrix": [{"name": "PESS (ESS Site)","sequence": "TAAATATA","CV": "-40.68577638232","position": "1"},{"name": "PESS (ESS Site)","sequence": "AAATATAT","CV": "-37.29629737162","position": "2"},{"name": "ESE_Tra2 (ESE Site)","sequence": "AATAT","CV": "85.76","position": "3"},{"name": "PESS (ESS Site)","sequence": "ATATATTT","CV": "-52.43779526253","position": "4"},{"name": "IIE (ESS Site)","sequence": "TATATT","CV": "NA","position": "5"},{"name": "PESS (ESS Site)","sequence": "TATATTTT","CV": "-57.32085160038","position": "5"},{"name": "IIE (ESS Site)","sequence": "ATATTT","CV": "NA","position": "6"},{"name": "PESS (ESS Site)","sequence": "ATATTTTA","CV": "-37.79609206177","position": "6"},{"name": "IIE (ESS Site)","sequence": "TATTTT","CV": "NA","position": "7"},{"name": "PESS (ESS Site)","sequence": "TATTTTAT","CV": "-50.45271859278","position": "7"},{"name": "IIE (ESS Site)","sequence": "ATTTTA","CV": "NA","position": "8"},{"name": "IIE (ESS Site)","sequence": "TTTTAT","CV": "NA","position": "9"},{"name": "IIE (ESS Site)","sequence": "TTTATC","CV": "NA","position": "10"},{"name": "PESS (ESS Site)","sequence": "TTTATCTT","CV": "-32.26102140614","position": "10"},{"name": "IIE (ESS Site)","sequence": "TTATCT","CV": "NA","position": "11"},{"name": "PESS (ESS Site)","sequence": "TTATCTTT","CV": "-34.8193065919","position": "11"},{"name": "IIE (ESS Site)","sequence": "TATCTT","CV": "NA","position": "12"},{"name": "IIE (ESS Site)","sequence": "ATCTTT","CV": "NA","position": "13"},{"name": "IIE (ESS Site)","sequence": "TCTTTA","CV": "NA","position": "14"},{"name": "IIE (ESS Site)","sequence": "CTTTAG","CV": "NA","position": "15"},{"name": "IIE (ESS Site)","sequence": "TTTAGG","CV": "NA","position": "16"},{"name": "Fas ESS (ESS Site)","sequence": "TTTAGG","CV": "NA","position": "16"},{"name": "Sironi_motif2 (ESS Site)","sequence": "TTTAGGG","CV": "68.44","position": "16"},{"name": "IIE (ESS Site)","sequence": "TTAGGG","CV": "NA","position": "17"},{"name": "Fas ESS (ESS Site)","sequence": "TTAGGG","CV": "NA","position": "17"},{"name": "Sironi_motif2 (ESS Site)","sequence": "TTAGGGC","CV": "70.51","position": "17"},{"name": "Sironi_motif1 (ESS Site)","sequence": "TTAGGGCT","CV": "66.13","position": "17"},{"name": "ESS_hnRNPA1 (ESS Site)","sequence": "TAGGGC","CV": "98.46","position": "18"},{"name": "IIE (ESS Site)","sequence": "GGGCTT","CV": "NA","position": "20"},{"name": "Fas ESS (ESS Site)","sequence": "GGCTTA","CV": "NA","position": "21"},{"name": "Fas ESS (ESS Site)","sequence": "GCTTAG","CV": "NA","position": "22"},{"name": "Fas ESS (ESS Site)","sequence": "CTTAGG","CV": "NA","position": "23"},{"name": "ESE_ASF (ESE Site)","sequence": "CTTAGGT","CV": "74.81","position": "23"},{"name": "ESE_ASFB (ESE Site)","sequence": "CTTAGGT","CV": "75","position": "23"},{"name": "Fas ESS (ESS Site)","sequence": "TTAGGT","CV": "NA","position": "24"},{"name": "Sironi_motif2 (ESS Site)","sequence": "TTAGGTG","CV": "63.63","position": "24"},{"name": "ESS_hnRNPA1 (ESS Site)","sequence": "TAGGTG","CV": "85.84","position": "25"},{"name": "IIE (ESS Site)","sequence": "GGTGTG","CV": "NA","position": "27"},{"name": "Fas ESS (ESS Site)","sequence": "GGTGTG","CV": "NA","position": "27"},{"name": "Sironi_motif2 (ESS Site)","sequence": "GGTGTGA","CV": "69.67","position": "27"},{"name": "EIE (ESE Site)","sequence": "GTGTGA","CV": "NA","position": "28"},{"name": "IIE (ESS Site)","sequence":

"GTGTGA","CV": "NA","position": "28"},{"name": "IIE (ESS Site)","sequence": "TGTGAT","CV": "NA","position": "29"},{"name": "ESE_9G8 (ESE Site)","sequence": "GATCAT","CV": "60.74","position": "32"},{"name": "RESCUE ESE (ESE Site)","sequence": "GATCAT","CV": "NA","position": "32"},{"name": "ESE_SC35 (ESE Site)","sequence": "GATCATTG","CV": "80.41","position": "32"},{"name": "IIE (ESS Site)","sequence": "TCATTG","CV": "NA","position": "34"},{"name": "ESE_SRp40 (ESE Site)","sequence": "TCATTGC","CV": "79.07","position": "34"},{"name": "IIE (ESS Site)","sequence": "AATTTT","CV": "NA","position": "41"},{"name": "IIE (ESS Site)","sequence": "ATTTTG","CV": "NA","position": "42"},{"name": "IIE (ESS Site)","sequence": "TTTTGG","CV": "NA","position": "43"},{"name": "Fas ESS (ESS Site)","sequence": "TTTTGG","CV": "NA","position": "43"},{"name": "Sironi_motif2 (ESS Site)","sequence": "TTTTGGA","CV": "64.79","position": "43"},{"name": "RESCUE ESE (ESE Site)","sequence": "TTTGGA","CV": "NA","position": "44"},{"name": "EIE (ESE Site)","sequence": "TTTGGA","CV": "NA","position": "44"},{"name": "IIE (ESS Site)","sequence": "TTTGGA","CV": "NA","position": "44"},{"name": "RESCUE ESE (ESE Site)","sequence": "TTGGAT","CV": "NA","position": "45"},{"name": "IIE (ESS Site)","sequence": "TTGGAT","CV": "NA","position": "45"},{"name": "EIE (ESE Site)","sequence": "TGGATT","CV": "NA","position": "46"},{"name": "IIE (ESS Site)","sequence": "TGGATT","CV": "NA","position": "46"},{"name": "IIE (ESS Site)","sequence": "GGATTT","CV": "NA","position": "47"},{"name": "EIE (ESE Site)","sequence": "GATTTA","CV": "NA","position": "48"},{"name": "IIE (ESS Site)","sequence": "GATTTA","CV": "NA","position": "48"},{"name": "IIE (ESS Site)","sequence": "TTTCTC","CV": "NA","position": "56"},{"name": "ESE_SRp40 (ESE Site)","sequence": "TTTCTCC","CV": "83.32","position": "56"},{"name": "Sironi_motif3 (ESS Site)","sequence": "TTTCTCCG","CV": "60.35","position": "56"},{"name": "PESE (ESE Site)","sequence": "TTTCTCCG","CV": "32.80229283406","position": "56"},{"name": "PESE (ESE Site)","sequence": "TTCTCCGA","CV": "31.14114948631","position": "57"},{"name": "Sironi_motif3 (ESS Site)","sequence": "TCTCCGAA","CV": "87.73","position": "58"},{"name": "PESE (ESE Site)","sequence": "TCTCCGAA","CV": "28.48871210582","position": "58"},{"name": "ESE_ASFB (ESE Site)","sequence": "CTCCGAA","CV": "75.85","position": "59"},{"name": "ESE_9G8 (ESE Site)","sequence": "GAAGCT","CV": "67.32","position": "63"},{"name": "EIE (ESE Site)","sequence": "GAAGCT","CV": "NA","position": "63"},{"name": "IIE (ESS Site)","sequence": "TTTAAT","CV": "NA","position": "68"},{"name": "PESS (ESS Site)","sequence": "TTTAATGA","CV": "-33.36015495709","position": "68"},{"name": "IIE (ESS Site)","sequence": "TTAATG","CV": "NA","position": "69"},{"name": "ESS_hnRNPA1 (ESS Site)","sequence": "TAATGA","CV": "67.98","position": "70"},{"name": "EIE (ESE Site)","sequence": "TAATGA","CV": "NA","position": "70"},{"name": "ESE_9G8 (ESE Site)","sequence": "AATGAT","CV": "60.74","position": "71"},{"name": "RESCUE ESE (ESE Site)","sequence": "ATGATG","CV": "NA","position": "72"},{"name": "EIE (ESE Site)","sequence": "ATGATG","CV": "NA","position": "72"},{"name": "IIE (ESS Site)","sequence": "TGATGT","CV": "NA","position": "73"},{"name": "Sironi_motif2 (ESS Site)","sequence": "TGATGTT","CV": "60.51","position": "73"},{"name": "ESE_9G8 (ESE Site)","sequence": "GATGTT","CV": "63.09","position": "74"},{"name": "IIE (ESS Site)","sequence": "GATGTT","CV": "NA","position": "74"},{"name": "IIE (ESS Site)","sequence": "ATGTTG","CV": "NA","position": "75"},{"name": "IIE (ESS Site)","sequence": "TGTTGG","CV": "NA","position": "76"},{"name": "Sironi_motif2 (ESS Site)","sequence": "TGTTGGA","CV": "81.69","position": "76"},{"name": "RESCUE ESE (ESE Site)","sequence": "GTTGGA","CV": "NA","position": "77"},{"name": "IIE (ESS Site)","sequence": "GTTGGA","CV": "NA","position": "77"},{"name": "RESCUE ESE (ESE Site)","sequence": "TTGGAT","CV": "NA","position": "78"},{"name": "IIE (ESS Site)","sequence": "TTGGAT","CV": "NA","position": "78"},{"name": "EIE (ESE Site)","sequence": "TGGATG","CV": "NA","position": "79"},{"name": "IIE (ESS Site)","sequence": "TGGATG","CV": "NA","position": "79"},{"name": "IIE (ESS Site)","sequence": "GGATGT","CV": "NA","position": "80"},{"name": "Sironi_motif2 (ESS Site)","sequence": "GGATGTG","CV": "69.5","position": "80"},{"name": "ESE_9G8 (ESE Site)","sequence": "GATGTG","CV": "61.08","position": "81"},{"name": "EIE (ESE Site)","sequence": "GATGTG","CV": "NA","position": "81"},{"name": "IIE (ESS Site)","sequence": "GATGTG","CV": "NA","position": "81"},{"name": "EIE (ESE Site)","sequence": "ATGTGA","CV": "NA","position": "82"},{"name": "IIE (ESS Site)","sequence": "ATGTGA","CV": "NA","position":

"82"},{"name": "EIE (ESE Site)","sequence": "TGTGAG","CV": "NA","position": "83"},{"name": "IIE (ESS Site)","sequence": "TGTGAG","CV": "NA","position": "83"},{"name": "Sironi_motif2 (ESS Site)","sequence": "TGTGAGC","CV": "73.56","position": "83"},{"name": "EIE (ESE Site)","sequence": "GAGCCC","CV": "NA","position": "86"},{"name": "ESE_SC35 (ESE Site)","sequence": "GAGCCCTG","CV": "83.17","position": "86"},{"name": "EIE (ESE Site)","sequence": "AGCCCT","CV": "NA","position": "87"},{"name": "PESE (ESE Site)","sequence": "AGCCCTGC","CV": "53.79076644864","position": "87"},{"name": "EIE (ESE Site)","sequence": "GCCCTG","CV": "NA","position": "88"},{"name": "EIE (ESE Site)","sequence": "CCCTGC","CV": "NA","position": "89"},{"name": "EIE (ESE Site)","sequence": "CCTGCC","CV": "NA","position": "90"},{"name": "Sironi_motif3 (ESS Site)","sequence": "CCTGCCAG","CV": "78.21","position": "90"},{"name": "PESE (ESE Site)","sequence": "CCTGCCAG","CV": "42.75505082974","position": "90"},{"name": "ESE_ASFB (ESE Site)","sequence": "CTGCCAG","CV": "71.23","position": "91"},{"name": "ESE_SC35 (ESE Site)","sequence": "TGCCAGTG","CV": "78.5","position": "92"},{"name": "EIE (ESE Site)","sequence": "CCAGTG","CV": "NA","position": "94"},{"name": "ESS_hnRNPA1 (ESS Site)","sequence": "CAGTGT","CV": "68.94","position": "95"},{"name": "IIE (ESS Site)","sequence": "CAGTGT","CV": "NA","position": "95"},{"name": "IIE (ESS Site)","sequence": "AGTGTA","CV": "NA","position": "96"},{"name": "Fas ESS (ESS Site)","sequence": "AGTGTA","CV": "NA","position": "96"},{"name": "IIE (ESS Site)","sequence": "GTGTAA","CV": "NA","position": "97"},{"name": "EIE (ESE Site)","sequence": "AACCTC","CV": "NA","position": "101"},{"name": "ESE_SC35 (ESE Site)","sequence": "AACCTCCA","CV": "75.24","position": "101"},{"name": "Sironi_motif3 (ESS Site)","sequence": "CCTCCATG","CV": "68.8","position": "103"},{"name": "RESCUE ESE (ESE Site)","sequence": "CTCCAT","CV": "NA","position": "104"},{"name": "ESE_9G8 (ESE Site)","sequence": "CATGGC","CV": "60.27","position": "107"},{"name": "ESE_SC35 (ESE Site)","sequence": "GCTCAGTG","CV": "75.12","position": "111"},{"name": "EIE (ESE Site)","sequence": "CTCAGT","CV": "NA","position": "112"},{"name": "ESE_ASF (ESE Site)","sequence": "CTCAGTG","CV": "73.71","position": "112"},{"name": "ESE_ASFB (ESE Site)","sequence": "CTCAGTG","CV": "77.46","position": "112"},{"name": "EIE (ESE Site)","sequence": "TCAGTG","CV": "NA","position": "113"},{"name": "IIE (ESS Site)","sequence": "TCAGTG","CV": "NA","position": "113"},{"name": "ESS_hnRNPA1 (ESS Site)","sequence": "CAGTGA","CV": "69.89","position": "114"},{"name": "EIE (ESE Site)","sequence": "CAGTGA","CV": "NA","position": "114"},{"name": "RESCUE ESE (ESE Site)","sequence": "AGTGAA","CV": "NA","position": "115"},{"name": "EIE (ESE Site)","sequence": "AGTGAA","CV": "NA","position": "115"},{"name": "PESE (ESE Site)","sequence": "AGTGAACA","CV": "37.07605589405","position": "115"},{"name": "EIE (ESE Site)","sequence": "GTGAAC","CV": "NA","position": "116"},{"name": "PESE (ESE Site)","sequence": "GTGAACAA","CV": "27.90389010324","position": "116"},{"name": "EIE (ESE Site)","sequence": "TGAACA","CV": "NA","position": "117"},{"name": "ESE_9G8 (ESE Site)","sequence": "GAACAA","CV": "65.98","position": "118"},{"name": "RESCUE ESE (ESE Site)","sequence": "GAACAA","CV": "NA","position": "118"},{"name": "EIE (ESE Site)","sequence": "GAACAA","CV": "NA","position": "118"},{"name": "ESE_Tra2 (ESE Site)","sequence": "AACAA","CV": "94.47","position": "119"},{"name": "EIE (ESE Site)","sequence": "AACAAA","CV": "NA","position": "119"},{"name": "EIE (ESE Site)","sequence": "ACAAAT","CV": "NA","position": "120"},{"name": "ESE_SRp40 (ESE Site)","sequence": "ATTCTGC","CV": "78.89","position": "124"},{"name": "Sironi_motif3 (ESS Site)","sequence": "TCTGCAAT","CV": "63.65","position": "126"},{"name": "EIE (ESE Site)","sequence": "CCTCAC","CV": "NA","position": "134"},{"name": "Sironi_motif3 (ESS Site)","sequence": "CCTCACTC","CV": "69.22","position": "134"},{"name": "ESE_ASFB (ESE Site)","sequence": "CTCACTC","CV": "71.46","position": "135"},{"name": "ESE_SRp40 (ESE Site)","sequence": "TCACTCT","CV": "82.3","position": "136"},{"name": "ESE_SRp40 (ESE Site)","sequence": "ACTCTGG","CV": "85.83","position": "138"},{"name": "ESE_ASFB (ESE Site)","sequence": "CTCTGGG","CV": "76.62","position": "139"},{"name": "IIE (ESS Site)","sequence": "TCTGGG","CV": "NA","position": "140"},{"name": "Sironi_motif2 (ESS Site)","sequence": "TCTGGGC","CV": "73.56","position": "140"},{"name": "IIE (ESS Site)","sequence": "CTGGGC","CV": "NA","position": "141"},{"name": "IIE (ESS Site)","sequence": "GGGCAG","CV": "NA","position": "143"},{"name": "ESE_SC35 (ESE Site)","sequence": "GGGCAGTG","CV": "76.9","position":

"143"},{"name": "EIE (ESE Site)","sequence": "GCAGTG","CV": "NA","position": "145"},{"name": "ESS_hnRNPA1 (ESS Site)","sequence": "CAGTGT","CV": "68.94","position": "146"},{"name": "IIE (ESS Site)","sequence": "CAGTGT","CV": "NA","position": "146"},{"name": "EIE (ESE Site)","sequence": "AGTGTG","CV": "NA","position": "147"},{"name": "IIE (ESS Site)","sequence": "AGTGTG","CV": "NA","position": "147"},{"name": "Sironi_motif2 (ESS Site)","sequence": "AGTGTGA","CV": "65.92","position": "147"},{"name": "EIE (ESE Site)","sequence": "GTGTGA","CV": "NA","position": "148"},{"name": "IIE (ESS Site)","sequence": "GTGTGA","CV": "NA","position": "148"},{"name": "EIE (ESE Site)","sequence": "TGTGAG","CV": "NA","position": "149"},{"name": "IIE (ESS Site)","sequence": "TGTGAG","CV": "NA","position": "149"},{"name": "Sironi_motif2 (ESS Site)","sequence": "TGTGAGT","CV": "71.52","position": "149"},{"name": "IIE (ESS Site)","sequence": "GTGAGT","CV": "NA","position": "150"},{"name": "IIE (ESS Site)","sequence": "TGAGTG","CV": "NA","position": "151"},{"name": "ESE_SRp40 (ESE Site)","sequence": "TGAGTGC","CV": "78.11","position": "151"},{"name": "Sironi_motif2 (ESS Site)","sequence": "TGAGTGC","CV": "70.51","position": "151"},{"name": "ESS_hnRNPA1 (ESS Site)","sequence": "GAGTGC","CV": "71.79","position": "152"},{"name": "EIE (ESE Site)","sequence": "GAGTGC","CV": "NA","position": "152"},{"name": "RESCUE ESE (ESE Site)","sequence": "GCAAAA","CV": "NA","position": "156"},{"name": "EIE (ESE Site)","sequence": "GCAAAA","CV": "NA","position": "156"},{"name": "EIE (ESE Site)","sequence": "CAAAAA","CV": "NA","position": "157"},{"name": "ESE_Tra2 (ESE Site)","sequence": "AAAAA","CV": "94.47","position": "158"},{"name": "EIE (ESE Site)","sequence": "AAAAAA","CV": "NA","position": "158"},{"name": "ESE_Tra2 (ESE Site)","sequence": "AAAAA","CV": "94.47","position": "159"},{"name": "RESCUE ESE (ESE Site)","sequence": "AAAAAG","CV": "NA","position": "159"},{"name": "EIE (ESE Site)","sequence": "AAAAAG","CV": "NA","position": "159"},{"name": "ESE_Tra2 (ESE Site)","sequence": "AAAAG","CV": "83.54","position": "160"},{"name": "RESCUE ESE (ESE Site)","sequence": "AAAAGA","CV": "NA","position": "160"},{"name": "EIE (ESE Site)","sequence": "AAAAGA","CV": "NA","position": "160"},{"name": "PESE (ESE Site)","sequence": "AAAAGAAG","CV": "36.91927382528","position": "160"},{"name": "ESE_9G8 (ESE Site)","sequence": "AAAGAA","CV": "65.98","position": "161"},{"name": "RESCUE ESE (ESE Site)","sequence": "AAAGAA","CV": "NA","position": "161"},{"name": "EIE (ESE Site)","sequence": "AAAGAA","CV": "NA","position": "161"},{"name": "Sironi_motif1 (ESS Site)","sequence": "AAAGAAGC","CV": "68.95","position": "161"},{"name": "ESE_Tra2 (ESE Site)","sequence": "AAGAA","CV": "100.34","position": "162"},{"name": "RESCUE ESE (ESE Site)","sequence": "AAGAAG","CV": "NA","position": "162"},{"name": "EIE (ESE Site)","sequence": "AAGAAG","CV": "NA","position": "162"},{"name": "PESE (ESE Site)","sequence": "AAGAAGCC","CV": "58.83143439002","position": "162"},{"name": "RESCUE ESE (ESE Site)","sequence": "AGAAGC","CV": "NA","position": "163"},{"name": "EIE (ESE Site)","sequence": "AGAAGC","CV": "NA","position": "163"},{"name": "ESE_9G8 (ESE Site)","sequence": "GAAGCC","CV": "80.41","position": "164"},{"name": "EIE (ESE Site)","sequence": "GAAGCC","CV": "NA","position": "164"},{"name": "RESCUE ESE (ESE Site)","sequence": "AAGCCA","CV": "NA","position": "165"},{"name": "EIE (ESE Site)","sequence": "AAGCCA","CV": "NA","position": "165"},{"name": "EIE (ESE Site)","sequence": "AGCCAA","CV": "NA","position": "166"},{"name": "ESE_SRp40 (ESE Site)","sequence": "CCAAAGG","CV": "88.35","position": "168"},{"name": "Sironi_motif1 (ESS Site)","sequence": "CCAAAGGA","CV": "71.6","position": "168"},{"name": "ESE_ASF (ESE Site)","sequence": "CAAAGGA","CV": "83.37","position": "169"},{"name": "ESE_ASFB (ESE Site)","sequence": "CAAAGGA","CV": "82.46","position": "169"},{"name": "ESS_hnRNPA1 (ESS Site)","sequence": "AAAGGA","CV": "67.98","position": "170"},{"name": "RESCUE ESE (ESE Site)","sequence": "AAAGGA","CV": "NA","position": "170"},{"name": "EIE (ESE Site)","sequence": "AAAGGA","CV": "NA","position": "170"},{"name": "ESE_9G8 (ESE Site)","sequence": "AAGGAC","CV": "73.23","position": "171"},{"name": "ESS_hnRNPA1 (ESS Site)","sequence": "AAGGAC","CV": "74.89","position": "171"},{"name": "RESCUE ESE (ESE Site)","sequence": "AAGGAC","CV": "NA","position": "171"},{"name": "EIE (ESE Site)","sequence": "AAGGAC","CV": "NA","position": "171"},{"name": "EIE (ESE Site)","sequence": "AGGACT","CV": "NA","position": "172"},{"name": "PESE (ESE Site)","sequence": "AGGACTTC","CV": "37.03914159744","position": "172"},{"name": "EIE (ESE Site)","sequence": "GGACTT","CV": "NA","position": "173"},{"name": "IIE

(ESS Site)","sequence": "GGACTT","CV": "NA","position": "173"},{"name": "ESE_9G8 (ESE Site)","sequence": "GACTTC","CV": "59.27","position": "174"},{"name": "ESE_SC35 (ESE Site)","sequence": "GACTTCAG","CV": "85.02","position": "174"},{"name": "RESCUE ESE (ESE Site)","sequence": "ACTTCA","CV": "NA","position": "175"},{"name": "RESCUE ESE (ESE Site)","sequence": "CTTCAG","CV": "NA","position": "176"},{"name": "IIE (ESS Site)","sequence": "TTCAGT","CV": "NA","position": "177"},{"name": "EIE (ESE Site)","sequence": "TCAGTG","CV": "NA","position": "178"},{"name": "IIE (ESS Site)","sequence": "TCAGTG","CV": "NA","position": "178"},{"name": "ESS_hnRNPA1 (ESS Site)","sequence": "CAGTGT","CV": "68.94","position": "179"},{"name": "IIE (ESS Site)","sequence": "CAGTGT","CV": "NA","position": "179"},{"name": "EIE (ESE Site)","sequence": "AGTGTG","CV": "NA","position": "180"},{"name": "IIE (ESS Site)","sequence": "AGTGTG","CV": "NA","position": "180"},{"name": "Sironi_motif2 (ESS Site)","sequence": "AGTGTGA","CV": "65.92","position": "180"},{"name": "EIE (ESE Site)","sequence": "GTGTGA","CV": "NA","position": "181"},{"name": "IIE (ESS Site)","sequence": "GTGTGA","CV": "NA","position": "181"},{"name": "ESE_9G8 (ESE Site)","sequence": "TGTGAC","CV": "60.94","position": "182"},{"name": "EIE (ESE Site)","sequence": "TGTGAC","CV": "NA","position": "182"},{"name": "IIE (ESS Site)","sequence": "TGTGAC","CV": "NA","position": "182"},{"name": "ESE_SRp40 (ESE Site)","sequence": "TGACACC","CV": "88.05","position": "184"},{"name": "ESE_9G8 (ESE Site)","sequence": "GACACC","CV": "59.27","position": "185"},{"name": "EIE (ESE Site)","sequence": "GACACC","CV": "NA","position": "185"},{"name": "ESE_ASF (ESE Site)","sequence": "GACACCT","CV": "74.17","position": "185"},{"name": "ESE_SC35 (ESE Site)","sequence": "GACACCTG","CV": "80.84","position": "185"},{"name": "EIE (ESE Site)","sequence": "ACACCT","CV": "NA","position": "186"},{"name": "PESE (ESE Site)","sequence": "ACACCTGC","CV": "35.97650757573","position": "186"},{"name": "EIE (ESE Site)","sequence": "CACCTG","CV": "NA","position": "187"},{"name": "EIE (ESE Site)","sequence": "ACCTGC","CV": "NA","position": "188"},{"name": "PESE (ESE Site)","sequence": "ACCTGCAG","CV": "56.05539633097","position": "188"},{"name": "EIE (ESE Site)","sequence": "CCTGCA","CV": "NA","position": "189"},{"name": "PESE (ESE Site)","sequence": "CCTGCAGA","CV": "48.10181709588","position": "189"},{"name": "ESE_ASFB (ESE Site)","sequence": "CTGCAGA","CV": "73.54","position": "190"},{"name": "ESE_SRp55 (ESE Site)","sequence": "TGCAGA","CV": "84.85","position": "191"},{"name": "EIE (ESE Site)","sequence": "TGCAGA","CV": "NA","position": "191"},{"name": "PESE (ESE Site)","sequence": "TGCAGAGA","CV": "27.63553560985","position": "191"},{"name": "ESE_9G8 (ESE Site)","sequence": "GCAGAG","CV": "67.52","position": "192"},{"name": "EIE (ESE Site)","sequence": "GCAGAG","CV": "NA","position": "192"},{"name": "Sironi_motif1 (ESS Site)","sequence": "GCAGAGAA","CV": "66.3","position": "192"},{"name": "PESE (ESE Site)","sequence": "GCAGAGAA","CV": "43.29258935126","position": "192"},{"name": "ESS_hnRNPA1 (ESS Site)","sequence": "CAGAGA","CV": "73.7","position": "193"},{"name": "EIE (ESE Site)","sequence": "CAGAGA","CV": "NA","position": "193"},{"name": "ESE_ASF (ESE Site)","sequence": "CAGAGAA","CV": "80.4","position": "193"},{"name": "ESE_ASFB (ESE Site)","sequence": "CAGAGAA","CV": "79.38","position": "193"},{"name": "PESE (ESE Site)","sequence": "CAGAGAAA","CV": "30.84251697435","position": "193"},{"name": "RESCUE ESE (ESE Site)","sequence": "AGAGAA","CV": "NA","position": "194"},{"name": "EIE (ESE Site)","sequence": "AGAGAA","CV": "NA","position": "194"},{"name": "ESE_9G8 (ESE Site)","sequence": "GAGAAA","CV": "62.35","position": "195"},{"name": "ESS_hnRNPA1 (ESS Site)","sequence": "GAGAAA","CV": "69.17","position": "195"},{"name": "RESCUE ESE (ESE Site)","sequence": "GAGAAA","CV": "NA","position": "195"},{"name": "EIE (ESE Site)","sequence": "GAGAAA","CV": "NA","position": "195"},{"name": "RESCUE ESE (ESE Site)","sequence": "AGAAAA","CV": "NA","position": "196"},{"name": "EIE (ESE Site)","sequence": "AGAAAA","CV": "NA","position": "196"},{"name": "ESE_9G8 (ESE Site)","sequence": "GAAAAC","CV": "79.27","position": "197"},{"name": "RESCUE ESE (ESE Site)","sequence": "GAAAAC","CV": "NA","position": "197"},{"name": "EIE (ESE Site)","sequence": "GAAAAC","CV": "NA","position": "197"},{"name": "ESE_Tra2 (ESE Site)","sequence": "AAAAC","CV": "81.36","position": "198"},{"name": "EIE (ESE Site)","sequence": "AAAACT","CV": "NA","position": "198"},{"name": "IIE (ESS Site)","sequence": "ACTTTT","CV": "NA","position": "201"},{"name": "IIE (ESS Site)","sequence":

"CTTTTA","CV": "NA","position": "202"},{"name": "IIE (ESS Site)","sequence": "TTTTAT","CV": "NA","position": "203"},{"name": "EIE (ESE Site)","sequence": "TTTATG","CV": "NA","position": "204"},{"name": "IIE (ESS Site)","sequence": "TTTATG","CV": "NA","position": "204"},{"name": "ESE_SRp40 (ESE Site)","sequence": "TTTATGG","CV": "80.86","position": "204"},{"name": "EIE (ESE Site)","sequence": "TTATGG","CV": "NA","position": "205"},{"name": "IIE (ESS Site)","sequence": "TTATGG","CV": "NA","position": "205"},{"name": "Fas ESS (ESS Site)","sequence": "TTATGG","CV": "NA","position": "205"},{"name": "Sironi_motif2 (ESS Site)","sequence": "TTATGGG","CV": "72.57","position": "205"},{"name": "ESS_hnRNPA1 (ESS Site)","sequence": "TATGGG","CV": "78.22","position": "206"},{"name": "EIE (ESE Site)","sequence": "TATGGG","CV": "NA","position": "206"},{"name": "IIE (ESS Site)","sequence": "TATGGG","CV": "NA","position": "206"},{"name": "Fas ESS (ESS Site)","sequence": "TATGGG","CV": "NA","position": "206"},{"name": "Sironi_motif2 (ESS Site)","sequence": "TATGGGT","CV": "71.52","position": "206"},{"name": "IIE (ESS Site)","sequence": "ATGGGT","CV": "NA","position": "207"},{"name": "PESS (ESS Site)","sequence": "ATGGGTTA","CV": "-29.35059871671","position": "207"},{"name": "IIE (ESS Site)","sequence": "TGGGTT","CV": "NA","position": "208"},{"name": "Fas ESS (ESS Site)","sequence": "GGGTTA","CV": "NA","position": "209"},{"name": "Fas ESS (ESS Site)","sequence": "GGTTAG","CV": "NA","position": "210"},{"name": "Sironi_motif2 (ESS Site)","sequence": "GGTTAGA","CV": "61.72","position": "210"},{"name": "IIE (ESS Site)","sequence": "GTTAGA","CV": "NA","position": "211"},{"name": "IIE (ESS Site)","sequence": "TTAGAT","CV": "NA","position": "212"},{"name": "Fas ESS (ESS Site)","sequence": "TTAGAT","CV": "NA","position": "212"},{"name": "Sironi_motif1 (ESS Site)","sequence": "TTAGATGT","CV": "73.77","position": "212"},{"name": "ESS_hnRNPA1 (ESS Site)","sequence": "TAGATG","CV": "74.41","position": "213"},{"name": "IIE (ESS Site)","sequence": "TAGATG","CV": "NA","position": "213"},{"name": "RESCUE ESE (ESE Site)","sequence": "AGATGT","CV": "NA","position": "214"},{"name": "IIE (ESS Site)","sequence": "AGATGT","CV": "NA","position": "214"},{"name": "ESE_9G8 (ESE Site)","sequence": "GATGTC","CV": "76.18","position": "215"},{"name": "ESE_SRp40 (ESE Site)","sequence": "TGTCACC","CV": "81.76","position": "217"},{"name": "Sironi_motif3 (ESS Site)","sequence": "TGTCACCA","CV": "62.29","position": "217"},{"name": "Sironi_motif3 (ESS Site)","sequence": "TCACCAAT","CV": "65.51","position": "219"},{"name": "EIE (ESE Site)","sequence": "CACCAA","CV": "NA","position": "220"},{"name": "IIE (ESS Site)","sequence": "AATTGT","CV": "NA","position": "224"},{"name": "IIE (ESS Site)","sequence": "ATTGTA","CV": "NA","position": "225"},{"name": "IIE (ESS Site)","sequence": "TTGTAA","CV": "NA","position": "226"},{"name": "ESE_SRp40 (ESE Site)","sequence": "TGTAAGG","CV": "79.31","position": "227"},{"name": "Sironi_motif2 (ESS Site)","sequence": "TGTAAGG","CV": "68.44","position": "227"},{"name": "Fas ESS (ESS Site)","sequence": "GTAAGG","CV": "NA","position": "228"},{"name": "ESE_9G8 (ESE Site)","sequence": "TAAGGC","CV": "62.08","position": "229"},{"name": "ESS_hnRNPA1 (ESS Site)","sequence": "TAAGGC","CV": "81.32","position": "229"},{"name": "ESS_hnRNPA1 (ESS Site)","sequence": "AAGGCC","CV": "73.22","position": "230"},{"name": "ESE_SC35 (ESE Site)","sequence": "GGCCTGTG","CV": "89.32","position": "232"},{"name": "EIE (ESE Site)","sequence": "GCCTGT","CV": "NA","position": "233"},{"name": "IIE (ESS Site)","sequence": "GCCTGT","CV": "NA","position": "233"},{"name": "EIE (ESE Site)","sequence": "CCTGTG","CV": "NA","position": "234"},{"name": "IIE (ESS Site)","sequence": "CCTGTG","CV": "NA","position": "234"},{"name": "EIE (ESE Site)","sequence": "CTGTGA","CV": "NA","position": "235"},{"name": "IIE (ESS Site)","sequence": "CTGTGA","CV": "NA","position": "235"},{"name": "ESE_9G8 (ESE Site)","sequence": "TGTGAC","CV": "60.94","position": "236"},{"name": "EIE (ESE Site)","sequence": "TGTGAC","CV": "NA","position": "236"},{"name": "IIE (ESS Site)","sequence": "TGTGAC","CV": "NA","position": "236"},{"name": "IIE (ESS Site)","sequence": "GTGACT","CV": "NA","position": "237"},{"name": "RESCUE ESE (ESE Site)","sequence": "TGACTG","CV": "NA","position": "238"},{"name": "IIE (ESS Site)","sequence": "TGACTG","CV": "NA","position": "238"},{"name": "ESE_SRp40 (ESE Site)","sequence": "TGACTGT","CV": "79.25","position": "238"},{"name": "EIE (ESE Site)","sequence": "ACTGTG","CV": "NA","position": "240"},{"name": "IIE (ESS Site)","sequence": "ACTGTG","CV": "NA","position": "240"},{"name": "EIE (ESE Site)","sequence": "CTGTGA","CV": "NA","position": "241"},{"name": "IIE (ESS Site)","sequence": "CTGTGA","CV": "NA","position":

"241"},{"name": "ESE_9G8 (ESE Site)","sequence": "TGTGAC","CV": "60.94","position": "242"},{"name": "EIE (ESE Site)","sequence": "TGTGAC","CV": "NA","position": "242"},{"name": "IIE (ESS Site)","sequence": "TGTGAC","CV": "NA","position": "242"},{"name": "ESE_SRp40 (ESE Site)","sequence": "TGACACA","CV": "78.89","position": "244"},{"name": "ESE_SRp40 (ESE Site)","sequence": "ACACAGC","CV": "88.53","position": "246"},{"name": "Sironi_motif1 (ESS Site)","sequence": "ACACAGCT","CV": "64.29","position": "246"},{"name": "ESE_SRp55 (ESE Site)","sequence": "CACAGC","CV": "78","position": "247"},{"name": "ESE_ASF (ESE Site)","sequence": "CACAGCT","CV": "79.82","position": "247"},{"name": "ESE_ASFB (ESE Site)","sequence": "CACAGCT","CV": "81.54","position": "247"},{"name": "PESE (ESE Site)","sequence": "ACAGCTGG","CV": "29.11791421781","position": "248"},{"name": "ESE_ASF (ESE Site)","sequence": "CAGCTGG","CV": "74.23","position": "249"},{"name": "ESE_ASFB (ESE Site)","sequence": "CAGCTGG","CV": "74.31","position": "249"},{"name": "PESE (ESE Site)","sequence": "CAGCTGGA","CV": "52.56595838224","position": "249"},{"name": "EIE (ESE Site)","sequence": "AGCTGG","CV": "NA","position": "250"},{"name": "Sironi_motif2 (ESS Site)","sequence": "AGCTGGA","CV": "62.32","position": "250"},{"name": "EIE (ESE Site)","sequence": "GCTGGA","CV": "NA","position": "251"},{"name": "RESCUE ESE (ESE Site)","sequence": "TGGATC","CV": "NA","position": "253"},{"name": "ESE_SC35 (ESE Site)","sequence": "GATCCCTC","CV": "79.98","position": "255"},{"name": "Sironi_motif3 (ESS Site)","sequence": "GATCCCTC","CV": "71.38","position": "255"},{"name": "EIE (ESE Site)","sequence": "ATCCCT","CV": "NA","position": "256"},{"name": "Sironi_motif3 (ESS Site)","sequence": "CCTCCCTG","CV": "85.98","position": "259"},{"name": "ESE_ASFB (ESE Site)","sequence": "CTCCCTG","CV": "75.92","position": "260"},{"name": "EIE (ESE Site)","sequence": "TCCCTG","CV": "NA","position": "261"},{"name": "ESE_SRp40 (ESE Site)","sequence": "TCCCTGG","CV": "84.04","position": "261"},{"name": "EIE (ESE Site)","sequence": "CCCTGG","CV": "NA","position": "262"},{"name": "ESE_ASFB (ESE Site)","sequence": "CCCTGGG","CV": "74.69","position": "262"},{"name": "IIE (ESS Site)","sequence": "CCTGGG","CV": "NA","position": "263"},{"name": "Fas ESS (ESS Site)","sequence": "CCTGGG","CV": "NA","position": "263"},{"name": "Sironi_motif2 (ESS Site)","sequence": "CCTGGGA","CV": "62.88","position": "263"},{"name": "IIE (ESS Site)","sequence": "CTGGGA","CV": "NA","position": "264"},{"name": "ESE_9G8 (ESE Site)","sequence": "TGGGAC","CV": "60.34","position": "265"},{"name": "EIE (ESE Site)","sequence": "TGGGAC","CV": "NA","position": "265"},{"name": "IIE (ESS Site)","sequence": "TGGGAC","CV": "NA","position": "265"},{"name": "IIE (ESS Site)","sequence": "GGGACT","CV": "NA","position": "266"},{"name": "IIE (ESS Site)","sequence": "CTGTCT","CV": "NA","position": "270"},{"name": "IIE (ESS Site)","sequence": "TGTCTG","CV": "NA","position": "271"},{"name": "IIE (ESS Site)","sequence": "GTCTGT","CV": "NA","position": "272"},{"name": "IIE (ESS Site)","sequence": "TCTGTA","CV": "NA","position": "273"},{"name": "IIE (ESS Site)","sequence": "TGTAAT","CV": "NA","position": "275"},{"name": "IIE (ESS Site)","sequence": "GTAATG","CV": "NA","position": "276"},{"name": "ESS_hnRNPA1 (ESS Site)","sequence": "TAATGC","CV": "66.08","position": "277"},{"name": "EIE (ESE Site)","sequence": "ATGCTA","CV": "NA","position": "279"},{"name": "EIE (ESE Site)","sequence": "TGCTAA","CV": "NA","position": "280"},{"name": "ESE_9G8 (ESE Site)","sequence": "TAAGAC","CV": "78.06","position": "283"},{"name": "ESS_hnRNPA1 (ESS Site)","sequence": "TAAGAC","CV": "72.98","position": "283"},{"name": "ESE_Tra2 (ESE Site)","sequence": "AAGAC","CV": "87.22","position": "284"},{"name": "RESCUE ESE (ESE Site)","sequence": "AAGACA","CV": "NA","position": "284"},{"name": "EIE (ESE Site)","sequence": "AAGACA","CV": "NA","position": "284"},{"name": "EIE (ESE Site)","sequence": "AGACAG","CV": "NA","position": "285"},{"name": "ESE_SRp40 (ESE Site)","sequence": "AGACAGG","CV": "88.71","position": "285"},{"name": "Sironi_motif1 (ESS Site)","sequence": "AGACAGGG","CV": "65.25","position": "285"},{"name": "ESE_ASF (ESE Site)","sequence": "GACAGGG","CV": "83.37","position": "286"},{"name": "Sironi_motif2 (ESS Site)","sequence": "ACAGGGC","CV": "63.68","position": "287"},{"name": "ESE_9G8 (ESE Site)","sequence": "CAGGGC","CV": "59.67","position": "288"},{"name": "ESS_hnRNPA1 (ESS Site)","sequence": "CAGGGC","CV": "83.22","position": "288"},{"name": "IIE (ESS Site)","sequence": "GGGCAG","CV": "NA","position": "290"},{"name": "ESE_SC35 (ESE Site)","sequence": "GGGCAGTG","CV": "76.9","position": "290"},{"name": "EIE (ESE Site)","sequence":

"GCAGTG","CV": "NA","position": "292"},{"name": "ESS_hnRNPA1 (ESS Site)","sequence": "CAGTGC","CV": "67.98","position": "293"},{"name": "EIE (ESE Site)","sequence": "CAGTGC","CV": "NA","position": "293"},{"name": "PESE (ESE Site)","sequence": "GTGCATCT","CV": "29.81596771451","position": "295"},{"name": "ESE_SRp55 (ESE Site)","sequence": "TGCATC","CV": "92.4","position": "296"},{"name": "PESE (ESE Site)","sequence": "TGCATCTG","CV": "30.49162377281","position": "296"},{"name": "PESE (ESE Site)","sequence": "GCATCTGC","CV": "46.71690882169","position": "297"},{"name": "Sironi_motif3 (ESS Site)","sequence": "TCTGCAAG","CV": "66.83","position": "300"},{"name": "PESE (ESE Site)","sequence": "TCTGCAAG","CV": "31.08349682081","position": "300"},{"name": "EIE (ESE Site)","sequence": "TGCAAG","CV": "NA","position": "302"},{"name": "EIE (ESE Site)","sequence": "GCAAGC","CV": "NA","position": "303"},{"name": "ESE_9G8 (ESE Site)","sequence": "CAAGCC","CV": "61.08","position": "304"},{"name": "EIE (ESE Site)","sequence": "CAAGCC","CV": "NA","position": "304"},{"name": "EIE (ESE Site)","sequence": "AAGCCC","CV": "NA","position": "305"},{"name": "Sironi_motif3 (ESS Site)","sequence": "AAGCCCAA","CV": "67.49","position": "305"},{"name": "EIE (ESE Site)","sequence": "GCCCAA","CV": "NA","position": "307"},{"name": "ESE_SC35 (ESE Site)","sequence": "GCCCAATG","CV": "76.41","position": "307"},{"name": "IIE (ESS Site)","sequence": "AATGTT","CV": "NA","position": "311"},{"name": "IIE (ESS Site)","sequence": "ATGTTG","CV": "NA","position": "312"},{"name": "IIE (ESS Site)","sequence": "TGTTGA","CV": "NA","position": "313"},{"name": "Sironi_motif2 (ESS Site)","sequence": "TGTTGAA","CV": "64.79","position": "313"},{"name": "ESE_9G8 (ESE Site)","sequence": "GTTGAA","CV": "66.31","position": "314"},{"name": "IIE (ESS Site)","sequence": "GTTGAA","CV": "NA","position": "314"},{"name": "RESCUE ESE (ESE Site)","sequence": "TTGAAG","CV": "NA","position": "315"},{"name": "IIE (ESS Site)","sequence": "TTGAAG","CV": "NA","position": "315"},{"name": "RESCUE ESE (ESE Site)","sequence": "TGAAGG","CV": "NA","position": "316"},{"name": "EIE (ESE Site)","sequence": "TGAAGG","CV": "NA","position": "316"},{"name": "Sironi_motif2 (ESS Site)","sequence": "TGAAGGG","CV": "82.29","position": "316"},{"name": "ESE_9G8 (ESE Site)","sequence": "GAAGGG","CV": "68.73","position": "317"},{"name": "ESS_hnRNPA1 (ESS Site)","sequence": "GAAGGG","CV": "68.94","position": "317"},{"name": "Sironi_motif2 (ESS Site)","sequence": "GAAGGGA","CV": "66.62","position": "317"},{"name": "ESS_hnRNPA1 (ESS Site)","sequence": "AAGGGA","CV": "85.13","position": "318"},{"name": "EIE (ESE Site)","sequence": "AAGGGA","CV": "NA","position": "318"},{"name": "EIE (ESE Site)","sequence": "GGGAGA","CV": "NA","position": "320"},{"name": "ESE_9G8 (ESE Site)","sequence": "GGAGAC","CV": "86.92","position": "321"},{"name": "EIE (ESE Site)","sequence": "GGAGAC","CV": "NA","position": "321"},{"name": "ESS_hnRNPA1 (ESS Site)","sequence": "GAGACA","CV": "67.51","position": "322"},{"name": "EIE (ESE Site)","sequence": "GAGACA","CV": "NA","position": "322"},{"name": "EIE (ESE Site)","sequence": "AGACAG","CV": "NA","position": "323"},{"name": "EIE (ESE Site)","sequence": "GACAGT","CV": "NA","position": "324"},{"name": "EIE (ESE Site)","sequence": "ACAGTG","CV": "NA","position": "325"},{"name": "ESS_hnRNPA1 (ESS Site)","sequence": "CAGTGC","CV": "67.98","position": "326"},{"name": "EIE (ESE Site)","sequence": "CAGTGC","CV": "NA","position": "326"},{"name": "ESE_Tra2 (ESE Site)","sequence": "AATAA","CV": "98.87","position": "332"},{"name": "PESS (ESS Site)","sequence": "ATAAATGT","CV": "-38.91389014471","position": "333"},{"name": "PESS (ESS Site)","sequence": "TAAATGTT","CV": "-42.91515103754","position": "334"},{"name": "PESS (ESS Site)","sequence": "AAATGTTT","CV": "-44.3062808224","position": "335"},{"name": "IIE (ESS Site)","sequence": "AATGTT","CV": "NA","position": "336"},{"name": "IIE (ESS Site)","sequence": "ATGTTT","CV": "NA","position": "337"},{"name": "IIE (ESS Site)","sequence": "TGTTTG","CV": "NA","position": "338"},{"name": "Sironi_motif2 (ESS Site)","sequence": "TGTTTGG","CV": "75.62","position": "338"},{"name": "IIE (ESS Site)","sequence": "GTTTGG","CV": "NA","position": "339"},{"name": "Fas ESS (ESS Site)","sequence": "GTTTGG","CV": "NA","position": "339"},{"name": "Sironi_motif2 (ESS Site)","sequence": "GTTTGGA","CV": "61.72","position": "339"},{"name": "RESCUE ESE (ESE Site)","sequence": "TTTGGA","CV": "NA","position": "340"},{"name": "EIE (ESE Site)","sequence": "TTTGGA","CV": "NA","position": "340"},{"name": "IIE (ESS Site)","sequence": "TTTGGA","CV": "NA","position": "340"},{"name": "Sironi_motif2 (ESS Site)","sequence": "TTTGGAG","CV": "66.68","position": "340"},{"name": "EIE (ESE Site)","sequence": "TTGGAG","CV": "NA","position":

"341"},{"name": "IIE (ESS Site)","sequence": "TTGGAG","CV": "NA","position": "341"},{"name": "Sironi_motif1 (ESS Site)","sequence": "TTGGAGGG","CV": "66.66","position": "341"},{"name": "EIE (ESE Site)","sequence": "TGGAGG","CV": "NA","position": "342"},{"name": "IIE (ESS Site)","sequence": "TGGAGG","CV": "NA","position": "342"},{"name": "Sironi_motif2 (ESS Site)","sequence": "TGGAGGG","CV": "72.8","position": "342"},{"name": "IIE (ESS Site)","sequence": "GGAGGG","CV": "NA","position": "343"},{"name": "Fas ESS (ESS Site)","sequence": "GGAGGG","CV": "NA","position": "343"},{"name": "Sironi_motif2 (ESS Site)","sequence": "GGAGGGA","CV": "83.53","position": "343"},{"name": "ESE_9G8 (ESE Site)","sequence": "GAGGGA","CV": "66.92","position": "344"},{"name": "ESS_hnRNPA1 (ESS Site)","sequence": "GAGGGA","CV": "88.93","position": "344"},{"name": "IIE (ESS Site)","sequence": "GAGGGA","CV": "NA","position": "344"},{"name": "EIE (ESE Site)","sequence": "GGGAAA","CV": "NA","position": "346"},{"name": "ESE_9G8 (ESE Site)","sequence": "GGAAAC","CV": "66.38","position": "347"},{"name": "RESCUE ESE (ESE Site)","sequence": "GGAAAC","CV": "NA","position": "347"},{"name": "EIE (ESE Site)","sequence": "GGAAAC","CV": "NA","position": "347"},{"name": "PESE (ESE Site)","sequence": "GGAAACTT","CV": "37.14698111564","position": "347"},{"name": "RESCUE ESE (ESE Site)","sequence": "GAAACT","CV": "NA","position": "348"},{"name": "EIE (ESE Site)","sequence": "GAAACT","CV": "NA","position": "348"},{"name": "RESCUE ESE (ESE Site)","sequence": "AACTTC","CV": "NA","position": "350"},{"name": "ESE_SC35 (ESE Site)","sequence": "AACTTCTA","CV": "77.76","position": "350"},{"name": "ESE_SRp40 (ESE Site)","sequence": "CTTCTAC","CV": "79.6","position": "352"},{"name": "EIE (ESE Site)","sequence": "TTCTAC","CV": "NA","position": "353"},{"name": "EIE (ESE Site)","sequence": "TCTACC","CV": "NA","position": "354"},{"name": "Sironi_motif3 (ESS Site)","sequence": "TCTACCTA","CV": "78.32","position": "354"},{"name": "EIE (ESE Site)","sequence": "CTACCT","CV": "NA","position": "355"},{"name": "ESE_SRp55 (ESE Site)","sequence": "TACCTA","CV": "75.31","position": "356"},{"name": "EIE (ESE Site)","sequence": "TACCTA","CV": "NA","position": "356"},{"name": "EIE (ESE Site)","sequence": "ACCTAC","CV": "NA","position": "357"},{"name": "EIE (ESE Site)","sequence": "CCTACG","CV": "NA","position": "358"},{"name": "ESE_ASFB (ESE Site)","sequence": "CCTACGG","CV": "73.15","position": "358"},{"name": "EIE (ESE Site)","sequence": "CTACGG","CV": "NA","position": "359"},{"name": "ESE_9G8 (ESE Site)","sequence": "TACGGC","CV": "61.48","position": "360"},{"name": "ESE_SRp55 (ESE Site)","sequence": "TACGGC","CV": "87.22","position": "360"},{"name": "ESS_hnRNPA1 (ESS Site)","sequence": "TACGGC","CV": "79.17","position": "360"},{"name": "EIE (ESE Site)","sequence": "TACGGC","CV": "NA","position": "360"},{"name": "EIE (ESE Site)","sequence": "ACGGCA","CV": "NA","position": "361"},{"name": "RESCUE ESE (ESE Site)","sequence": "GCAAAA","CV": "NA","position": "364"},{"name": "EIE (ESE Site)","sequence": "GCAAAA","CV": "NA","position": "364"},{"name": "ESE_Tra2 (ESE Site)","sequence": "AAAAT","CV": "81.36","position": "366"},{"name": "EIE (ESE Site)","sequence": "AAAATA","CV": "NA","position": "366"},{"name": "ESE_Tra2 (ESE Site)","sequence": "AATAA","CV": "98.87","position": "368"},{"name": "PESS (ESS Site)","sequence": "AATTCTTT","CV": "-35.56132543063","position": "371"},{"name": "IIE (ESS Site)","sequence": "ATTCTT","CV": "NA","position": "372"},{"name": "IIE (ESS Site)","sequence": "TTCTTT","CV": "NA","position": "373"},{"name": "IIE (ESS Site)","sequence": "TCTTTC","CV": "NA","position": "374"},{"name": "IIE (ESS Site)","sequence": "TTTCCT","CV": "NA","position": "376"},{"name": "ESE_SC35 (ESE Site)","sequence": "TTCCTCTG","CV": "78.32","position": "377"},{"name": "IIE (ESS Site)","sequence": "CTCTGT","CV": "NA","position": "380"},{"name": "IIE (ESS Site)","sequence": "TCTGTC","CV": "NA","position": "381"},{"name": "IIE (ESS Site)","sequence": "CTGTCT","CV": "NA","position": "382"},{"name": "IIE (ESS Site)","sequence": "TGTCTG","CV": "NA","position": "383"},{"name": "ESE_SRp40 (ESE Site)","sequence": "TGTCTGC","CV": "82.12","position": "383"},{"name": "Sironi_motif3 (ESS Site)","sequence": "TCTGCCTT","CV": "71.43","position": "385"},{"name": "EIE (ESE Site)","sequence": "CTGCCT","CV": "NA","position": "386"},{"name": "ESE_ASFB (ESE Site)","sequence": "CTGCCTT","CV": "74.54","position": "386"},{"name": "IIE (ESS Site)","sequence": "TGCCTT","CV": "NA","position": "387"},{"name": "IIE (ESS Site)","sequence": "GCCTTG","CV": "NA","position": "388"},{"name": "Sironi_motif3 (ESS Site)","sequence": "CCTTGCAA","CV": "64.74","position": "389"},{"name": "ESE_SRp55 (ESE Site)","sequence":

"TGCAAC","CV": "75.76","position": "392"},{"name": "RESCUE ESE (ESE Site)","sequence": "TGCAAC","CV": "NA","position": "392"},{"name": "EIE (ESE Site)","sequence": "TGCAAC","CV": "NA","position": "392"},{"name": "EIE (ESE Site)","sequence": "GCAACT","CV": "NA","position": "393"},{"name": "EIE (ESE Site)","sequence": "CAACTG","CV": "NA","position": "394"},{"name": "EIE (ESE Site)","sequence": "ACTGTG","CV": "NA","position": "396"},{"name": "IIE (ESS Site)","sequence": "ACTGTG","CV": "NA","position": "396"},{"name": "EIE (ESE Site)","sequence": "CTGTGA","CV": "NA","position": "397"},{"name": "IIE (ESS Site)","sequence": "CTGTGA","CV": "NA","position": "397"},{"name": "IIE (ESS Site)","sequence": "TGTGAT","CV": "NA","position": "398"},{"name": "IIE (ESS Site)","sequence": "GTGATA","CV": "NA","position": "399"},{"name": "ESE_9G8 (ESE Site)","sequence": "GATAAG","CV": "59.94","position": "401"},{"name": "ESE_9G8 (ESE Site)","sequence": "TAAGAC","CV": "78.06","position": "403"},{"name": "ESS_hnRNPA1 (ESS Site)","sequence": "TAAGAC","CV": "72.98","position": "403"},{"name": "ESE_Tra2 (ESE Site)","sequence": "AAGAC","CV": "87.22","position": "404"},{"name": "RESCUE ESE (ESE Site)","sequence": "AAGACT","CV": "NA","position": "404"},{"name": "EIE (ESE Site)","sequence": "AAGACT","CV": "NA","position": "404"},{"name": "ESE_SRp40 (ESE Site)","sequence": "AGACTGG","CV": "87.21","position": "405"},{"name": "Sironi_motif2 (ESS Site)","sequence": "GACTGGG","CV": "60.01","position": "406"},{"name": "Sironi_motif2 (ESS Site)","sequence": "ACTGGGA","CV": "65.92","position": "407"},{"name": "IIE (ESS Site)","sequence": "CTGGGA","CV": "NA","position": "408"},{"name": "ESE_9G8 (ESE Site)","sequence": "TGGGAC","CV": "60.34","position": "409"},{"name": "EIE (ESE Site)","sequence": "TGGGAC","CV": "NA","position": "409"},{"name": "IIE (ESS Site)","sequence": "TGGGAC","CV": "NA","position": "409"},{"name": "EIE (ESE Site)","sequence": "GGACAA","CV": "NA","position": "411"},{"name": "ESE_SC35 (ESE Site)","sequence": "GGACAATA","CV": "79.91","position": "411"},{"name": "ESE_9G8 (ESE Site)","sequence": "GACAAT","CV": "65.57","position": "412"},{"name": "RESCUE ESE (ESE Site)","sequence": "GACAAT","CV": "NA","position": "412"},{"name": "EIE (ESE Site)","sequence": "GACAAT","CV": "NA","position": "412"},{"name": "ESE_Tra2 (ESE Site)","sequence": "AATAA","CV": "98.87","position": "415"},{"name": "EIE (ESE Site)","sequence": "AAATGG","CV": "NA","position": "418"},{"name": "PESS (ESS Site)","sequence": "AAATGGCT","CV": "-29.87154654312","position": "418"},{"name": "ESE_SC35 (ESE Site)","sequence": "GCTCTCTG","CV": "78.93","position": "423"},{"name": "Sironi_motif3 (ESS Site)","sequence": "GCTCTCTG","CV": "67.22","position": "423"},{"name": "IIE (ESS Site)","sequence": "TCTCTG","CV": "NA","position": "425"},{"name": "ESE_SRp40 (ESE Site)","sequence": "TCTCTGC","CV": "87.03","position": "425"},{"name": "ESE_SC35 (ESE Site)","sequence": "CTCTGCTG","CV": "76.23","position": "426"},{"name": "PESE (ESE Site)","sequence": "CTCTGCTG","CV": "60.98200324348","position": "426"},{"name": "IIE (ESS Site)","sequence": "TCTGCT","CV": "NA","position": "427"},{"name": "IIE (ESS Site)","sequence": "CTGCTG","CV": "NA","position": "428"},{"name": "ESE_ASFB (ESE Site)","sequence": "CTGCTGT","CV": "72.54","position": "428"},{"name": "PESE (ESE Site)","sequence": "CTGCTGTG","CV": "52.11552101004","position": "428"},{"name": "IIE (ESS Site)","sequence": "TGCTGT","CV": "NA","position": "429"},{"name": "Sironi_motif2 (ESS Site)","sequence": "TGCTGTG","CV": "63.08","position": "429"},{"name": "EIE (ESE Site)","sequence": "GCTGTG","CV": "NA","position": "430"},{"name": "IIE (ESS Site)","sequence": "GCTGTG","CV": "NA","position": "430"},{"name": "IIE (ESS Site)","sequence": "CTGTGT","CV": "NA","position": "431"},{"name": "ESE_SRp55 (ESE Site)","sequence": "TGTGTA","CV": "84.02","position": "432"},{"name": "IIE (ESS Site)","sequence": "TGTGTA","CV": "NA","position": "432"},{"name": "IIE (ESS Site)","sequence": "GTGTAA","CV": "NA","position": "433"},{"name": "ESE_Tra2 (ESE Site)","sequence": "AACAA","CV": "94.47","position": "437"},{"name": "EIE (ESE Site)","sequence": "AACAAA","CV": "NA","position": "437"},{"name": "EIE (ESE Site)","sequence": "ACAAAT","CV": "NA","position": "438"},{"name": "EIE (ESE Site)","sequence": "AAATCA","CV": "NA","position": "440"},{"name": "RESCUE ESE (ESE Site)","sequence": "AATCAA","CV": "NA","position": "441"},{"name": "EIE (ESE Site)","sequence": "AATCAA","CV": "NA","position": "441"},{"name": "EIE (ESE Site)","sequence": "ATCAAC","CV": "NA","position": "442"},{"name": "EIE (ESE Site)","sequence": "TCAACA","CV": "NA","position": "443"},{"name": "EIE (ESE Site)","sequence": "CAACAG","CV": "NA","position": "444"},{"name": "ESE_SRp40 (ESE Site)","sequence":

"CAACAGG","CV": "84.22","position": "444"},{"name": "Sironi_motif1 (ESS Site)","sequence": "CAACAGGA","CV": "73.82","position": "444"},{"name": "ESE_Tra2 (ESE Site)","sequence": "AACAG","CV": "83.54","position": "445"},{"name": "ESE_ASF (ESE Site)","sequence": "AACAGGA","CV": "82.21","position": "445"},{"name": "ESE_ASFB (ESE Site)","sequence": "AACAGGA","CV": "71.92","position": "445"},{"name": "ESE_9G8 (ESE Site)","sequence": "CAGGAC","CV": "75.64","position": "447"},{"name": "ESS_hnRNPA1 (ESS Site)","sequence": "CAGGAC","CV": "74.89","position": "447"},{"name": "RESCUE ESE (ESE Site)","sequence": "AGGACA","CV": "NA","position": "448"},{"name": "EIE (ESE Site)","sequence": "AGGACA","CV": "NA","position": "448"},{"name": "EIE (ESE Site)","sequence": "GGACAA","CV": "NA","position": "449"},{"name": "ESE_SC35 (ESE Site)","sequence": "GGACAATG","CV": "82.68","position": "449"},{"name": "ESE_9G8 (ESE Site)","sequence": "GACAAT","CV": "65.57","position": "450"},{"name": "RESCUE ESE (ESE Site)","sequence": "GACAAT","CV": "NA","position": "450"},{"name": "EIE (ESE Site)","sequence": "GACAAT","CV": "NA","position": "450"},{"name": "EIE (ESE Site)","sequence": "ACAATG","CV": "NA","position": "451"},{"name": "IIE (ESS Site)","sequence": "TGTCCT","CV": "NA","position": "455"},{"name": "Sironi_motif3 (ESS Site)","sequence": "TGTCCTTG","CV": "60.28","position": "455"},{"name": "IIE (ESS Site)","sequence": "GTCCTT","CV": "NA","position": "456"},{"name": "IIE (ESS Site)","sequence": "TCCTTG","CV": "NA","position": "457"},{"name": "Sironi_motif3 (ESS Site)","sequence": "CCTTGCAA","CV": "64.74","position": "458"},{"name": "EIE (ESE Site)","sequence": "AAATTA","CV": "NA","position": "464"},{"name": "Fas ESS (ESS Site)","sequence": "ATTAGG","CV": "NA","position": "466"},{"name": "Sironi_motif2 (ESS Site)","sequence": "ATTAGGG","CV": "61.61","position": "466"},{"name": "PESS (ESS Site)","sequence": "ATTAGGGG","CV": "-30.29502403577","position": "466"},{"name": "IIE (ESS Site)","sequence": "TTAGGG","CV": "NA","position": "467"},{"name": "Fas ESS (ESS Site)","sequence": "TTAGGG","CV": "NA","position": "467"},{"name": "Sironi_motif2 (ESS Site)","sequence": "TTAGGGG","CV": "80.53","position": "467"},{"name": "Sironi_motif1 (ESS Site)","sequence": "TTAGGGGT","CV": "75.08","position": "467"},{"name": "ESS_hnRNPA1 (ESS Site)","sequence": "TAGGGG","CV": "97.51","position": "468"},{"name": "Fas ESS (ESS Site)","sequence": "TAGGGG","CV": "NA","position": "468"},{"name": "PESS (ESS Site)","sequence": "TAGGGGTA","CV": "-26.29998465361","position": "468"},{"name": "IIE (ESS Site)","sequence": "AGGGGT","CV": "NA","position": "469"},{"name": "Fas ESS (ESS Site)","sequence": "AGGGGT","CV": "NA","position": "469"},{"name": "Fas ESS (ESS Site)","sequence": "GGGGTA","CV": "NA","position": "470"},{"name": "Fas ESS (ESS Site)","sequence": "GGGTAA","CV": "NA","position": "471"},{"name": "ESE_9G8 (ESE Site)","sequence": "GGTAAC","CV": "62.15","position": "472"},{"name": "ESE_SRp40 (ESE Site)","sequence": "TAACAGG","CV": "86.37","position": "474"},{"name": "Sironi_motif1 (ESS Site)","sequence": "TAACAGGT","CV": "72.64","position": "474"},{"name": "ESE_Tra2 (ESE Site)","sequence": "AACAG","CV": "83.54","position": "475"},{"name": "ESE_ASF (ESE Site)","sequence": "AACAGGT","CV": "80.17","position": "475"},{"name": "ESS_hnRNPA1 (ESS Site)","sequence": "CAGGTC","CV": "71.55","position": "477"},{"name": "IIE (ESS Site)","sequence": "GGTCTT","CV": "NA","position": "479"},{"name": "ESE_SC35 (ESE Site)","sequence": "GGTCTTCG","CV": "76.9","position": "479"},{"name": "IIE (ESS Site)","sequence": "GTCTTC","CV": "NA","position": "480"},{"name": "ESE_ASFB (ESE Site)","sequence": "CGCTGTA","CV": "73.54","position": "485"},{"name": "IIE (ESS Site)","sequence": "GCTGTA","CV": "NA","position": "486"},{"name": "IIE (ESS Site)","sequence": "TGTAAT","CV": "NA","position": "488"},{"name": "RESCUE ESE (ESE Site)","sequence": "AATCAG","CV": "NA","position": "491"},{"name": "EIE (ESE Site)","sequence": "AATCAG","CV": "NA","position": "491"},{"name": "EIE (ESE Site)","sequence": "TCAGTG","CV": "NA","position": "493"},{"name": "IIE (ESS Site)","sequence": "TCAGTG","CV": "NA","position": "493"},{"name": "ESS_hnRNPA1 (ESS Site)","sequence": "CAGTGT","CV": "68.94","position": "494"},{"name": "IIE (ESS Site)","sequence": "CAGTGT","CV": "NA","position": "494"},{"name": "EIE (ESE Site)","sequence": "AGTGTG","CV": "NA","position": "495"},{"name": "IIE (ESS Site)","sequence": "AGTGTG","CV": "NA","position": "495"},{"name": "Sironi_motif2 (ESS Site)","sequence": "AGTGTGA","CV": "65.92","position": "495"},{"name": "EIE (ESE Site)","sequence": "GTGTGA","CV": "NA","position": "496"},{"name": "IIE (ESS Site)","sequence": "GTGTGA","CV":

"NA","position": "496"},{"name": "EIE (ESE Site)","sequence": "TGTGAG","CV": "NA","position": "497"},{"name": "IIE (ESS Site)","sequence": "TGTGAG","CV": "NA","position": "497"},{"name": "Sironi_motif2 (ESS Site)","sequence": "TGTGAGC","CV": "73.56","position": "497"},{"name": "EIE (ESE Site)","sequence": "GAGCCT","CV": "NA","position": "500"},{"name": "EIE (ESE Site)","sequence": "CCTCAC","CV": "NA","position": "503"},{"name": "Sironi_motif3 (ESS Site)","sequence": "CCTCACAG","CV": "78.21","position": "503"},{"name": "ESE_ASF (ESE Site)","sequence": "CTCACAG","CV": "74.64","position": "504"},{"name": "ESE_ASFB (ESE Site)","sequence": "CTCACAG","CV": "77.31","position": "504"},{"name": "ESE_SRp40 (ESE Site)","sequence": "TCACAGG","CV": "99.9","position": "505"},{"name": "Sironi_motif1 (ESS Site)","sequence": "TCACAGGT","CV": "70.43","position": "505"},{"name": "ESE_ASF (ESE Site)","sequence": "CACAGGT","CV": "94.79","position": "506"},{"name": "ESE_ASFB (ESE Site)","sequence": "CACAGGT","CV": "94.23","position": "506"},{"name": "ESS_hnRNPA1 (ESS Site)","sequence": "CAGGTA","CV": "73.46","position": "508"},{"name": "EIE (ESE Site)","sequence": "GTACAA","CV": "NA","position": "511"},{"name": "EIE (ESE Site)","sequence": "TACAAT","CV": "NA","position": "512"},{"name": "IIE (ESS Site)","sequence": "AATTTG","CV": "NA","position": "515"},{"name": "EIE (ESE Site)","sequence": "ATTTGA","CV": "NA","position": "516"},{"name": "IIE (ESS Site)","sequence": "ATTTGA","CV": "NA","position": "516"},{"name": "EIE (ESE Site)","sequence": "TTTGAC","CV": "NA","position": "517"},{"name": "IIE (ESS Site)","sequence": "TTTGAC","CV": "NA","position": "517"},{"name": "ESE_9G8 (ESE Site)","sequence": "GACCAT","CV": "64.37","position": "520"},{"name": "EIE (ESE Site)","sequence": "GACCAT","CV": "NA","position": "520"},{"name": "ESE_SC35 (ESE Site)","sequence": "GACCATTG","CV": "83.91","position": "520"},{"name": "IIE (ESS Site)","sequence": "CCATTG","CV": "NA","position": "522"},{"name": "EIE (ESE Site)","sequence": "ATTGAC","CV": "NA","position": "524"},{"name": "EIE (ESE Site)","sequence": "TGACAA","CV": "NA","position": "526"},{"name": "ESE_9G8 (ESE Site)","sequence": "GACAAT","CV": "65.57","position": "527"},{"name": "RESCUE ESE (ESE Site)","sequence": "GACAAT","CV": "NA","position": "527"},{"name": "EIE (ESE Site)","sequence": "GACAAT","CV": "NA","position": "527"},{"name": "IIE (ESS Site)","sequence": "AATTTT","CV": "NA","position": "530"},{"name": "IIE (ESS Site)","sequence": "ATTTTC","CV": "NA","position": "531"},{"name": "IIE (ESS Site)","sequence": "TTTTCA","CV": "NA","position": "532"},{"name": "ESE_SRp40 (ESE Site)","sequence": "TTTCAAC","CV": "83.26","position": "533"},{"name": "EIE (ESE Site)","sequence": "TCAACA","CV": "NA","position": "535"},{"name": "EIE (ESE Site)","sequence": "CAACAC","CV": "NA","position": "536"},{"name": "ESE_SC35 (ESE Site)","sequence": "CAACACTG","CV": "75.8","position": "536"},{"name": "ESE_Tra2 (ESE Site)","sequence": "AACAC","CV": "81.36","position": "537"},{"name": "ESE_SRp40 (ESE Site)","sequence": "ACACTGC","CV": "87.03","position": "538"},{"name": "Sironi_motif3 (ESS Site)","sequence": "ACTGCCAG","CV": "75.36","position": "540"},{"name": "ESE_ASFB (ESE Site)","sequence": "CTGCCAG","CV": "71.23","position": "541"},{"name": "EIE (ESE Site)","sequence": "GCCAGA","CV": "NA","position": "543"},{"name": "EIE (ESE Site)","sequence": "CCAGAT","CV": "NA","position": "544"},{"name": "Sironi_motif1 (ESS Site)","sequence": "CCAGATGT","CV": "80.07","position": "544"},{"name": "EIE (ESE Site)","sequence": "CAGATG","CV": "NA","position": "545"},{"name": "ESE_ASF (ESE Site)","sequence": "CAGATGT","CV": "82.21","position": "545"},{"name": "ESE_ASFB (ESE Site)","sequence": "CAGATGT","CV": "79","position": "545"},{"name": "RESCUE ESE (ESE Site)","sequence": "AGATGT","CV": "NA","position": "546"},{"name": "IIE (ESS Site)","sequence": "AGATGT","CV": "NA","position": "546"},{"name": "Sironi_motif2 (ESS Site)","sequence": "AGATGTG","CV": "65.74","position": "546"},{"name": "ESE_9G8 (ESE Site)","sequence": "GATGTG","CV": "61.08","position": "547"},{"name": "EIE (ESE Site)","sequence": "GATGTG","CV": "NA","position": "547"},{"name": "IIE (ESS Site)","sequence": "GATGTG","CV": "NA","position": "547"},{"name": "IIE (ESS Site)","sequence": "ATGTGT","CV": "NA","position": "548"},{"name": "ESE_SRp55 (ESE Site)","sequence": "TGTGTG","CV": "75.05","position": "549"},{"name": "EIE (ESE Site)","sequence": "TGTGTG","CV": "NA","position": "549"},{"name": "IIE (ESS Site)","sequence": "TGTGTG","CV": "NA","position": "549"},{"name": "Sironi_motif2 (ESS Site)","sequence": "TGTGTGA","CV": "72.75","position": "549"},{"name": "EIE (ESE Site)","sequence": "GTGTGA","CV": "NA","position": "550"},{"name": "IIE (ESS Site)","sequence": "GTGTGA","CV": "NA","position":

"550"},{"name": "EIE (ESE Site)","sequence": "TGTGAG","CV": "NA","position": "551"},{"name": "IIE (ESS Site)","sequence": "TGTGAG","CV": "NA","position": "551"},{"name": "Sironi_motif2 (ESS Site)","sequence": "TGTGAGT","CV": "71.52","position": "551"},{"name": "IIE (ESS Site)","sequence": "GTGAGT","CV": "NA","position": "552"},{"name": "IIE (ESS Site)","sequence": "TGAGTG","CV": "NA","position": "553"},{"name": "Sironi_motif2 (ESS Site)","sequence": "TGAGTGT","CV": "68.47","position": "553"},{"name": "ESS_hnRNPA1 (ESS Site)","sequence": "GAGTGT","CV": "72.74","position": "554"},{"name": "EIE (ESE Site)","sequence": "GAGTGT","CV": "NA","position": "554"},{"name": "IIE (ESS Site)","sequence": "GAGTGT","CV": "NA","position": "554"},{"name": "EIE (ESE Site)","sequence": "AGTGTG","CV": "NA","position": "555"},{"name": "IIE (ESS Site)","sequence": "AGTGTG","CV": "NA","position": "555"},{"name": "Sironi_motif2 (ESS Site)","sequence": "AGTGTGA","CV": "65.92","position": "555"},{"name": "EIE (ESE Site)","sequence": "GTGTGA","CV": "NA","position": "556"},{"name": "IIE (ESS Site)","sequence": "GTGTGA","CV": "NA","position": "556"},{"name": "IIE (ESS Site)","sequence": "TGTGAT","CV": "NA","position": "557"},{"name": "IIE (ESS Site)","sequence": "GTGATT","CV": "NA","position": "558"},{"name": "ESE_SC35 (ESE Site)","sequence": "GATTCCTT","CV": "78.56","position": "560"},{"name": "IIE (ESS Site)","sequence": "TTCCTT","CV": "NA","position": "562"},{"name": "Fas ESS (ESS Site)","sequence": "TTCCTT","CV": "NA","position": "562"},{"name": "IIE (ESS Site)","sequence": "TCCTTG","CV": "NA","position": "563"},{"name": "IIE (ESS Site)","sequence": "CCTTGG","CV": "NA","position": "564"},{"name": "Sironi_motif2 (ESS Site)","sequence": "CCTTGGG","CV": "65.75","position": "564"},{"name": "IIE (ESS Site)","sequence": "CTTGGG","CV": "NA","position": "565"},{"name": "Fas ESS (ESS Site)","sequence": "CTTGGG","CV": "NA","position": "565"},{"name": "Sironi_motif2 (ESS Site)","sequence": "CTTGGGG","CV": "73.71","position": "565"},{"name": "Sironi_motif1 (ESS Site)","sequence": "CTTGGGGA","CV": "60.65","position": "565"},{"name": "IIE (ESS Site)","sequence": "TTGGGG","CV": "NA","position": "566"},{"name": "Fas ESS (ESS Site)","sequence": "TTGGGG","CV": "NA","position": "566"},{"name": "Sironi_motif2 (ESS Site)","sequence": "TTGGGGA","CV": "60.21","position": "566"},{"name": "EIE (ESE Site)","sequence": "TGGGGA","CV": "NA","position": "567"},{"name": "IIE (ESS Site)","sequence": "TGGGGA","CV": "NA","position": "567"},{"name": "Fas ESS (ESS Site)","sequence": "TGGGGA","CV": "NA","position": "567"},{"name": "Sironi_motif2 (ESS Site)","sequence": "TGGGGAC","CV": "61.01","position": "567"},{"name": "ESE_9G8 (ESE Site)","sequence": "GGGGAC","CV": "82.08","position": "568"},{"name": "EIE (ESE Site)","sequence": "GGGGAC","CV": "NA","position": "568"},{"name": "IIE (ESS Site)","sequence": "GGGGAC","CV": "NA","position": "568"},{"name": "EIE (ESE Site)","sequence": "GGACAT","CV": "NA","position": "570"},{"name": "ESE_SC35 (ESE Site)","sequence": "GGACATTA","CV": "77.15","position": "570"},{"name": "EIE (ESE Site)","sequence": "GACATT","CV": "NA","position": "571"},{"name": "EIE (ESE Site)","sequence": "ACATTA","CV": "NA","position": "572"},{"name": "EIE (ESE Site)","sequence": "CATTAC","CV": "NA","position": "573"},{"name": "EIE (ESE Site)","sequence": "ATTACC","CV": "NA","position": "574"},{"name": "EIE (ESE Site)","sequence": "TTACCT","CV": "NA","position": "575"},{"name": "IIE (ESS Site)","sequence": "TTACCT","CV": "NA","position": "575"},{"name": "EIE (ESE Site)","sequence": "TACCTG","CV": "NA","position": "576"},{"name": "EIE (ESE Site)","sequence": "ACCTGG","CV": "NA","position": "577"},{"name": "IIE (ESS Site)","sequence": "CCTGGG","CV": "NA","position": "578"},{"name": "Fas ESS (ESS Site)","sequence": "CCTGGG","CV": "NA","position": "578"},{"name": "Sironi_motif2 (ESS Site)","sequence": "CCTGGGA","CV": "62.88","position": "578"},{"name": "IIE (ESS Site)","sequence": "CTGGGA","CV": "NA","position": "579"},{"name": "ESE_9G8 (ESE Site)","sequence": "TGGGAC","CV": "60.34","position": "580"},{"name": "EIE (ESE Site)","sequence": "TGGGAC","CV": "NA","position": "580"},{"name": "IIE (ESS Site)","sequence": "TGGGAC","CV": "NA","position": "580"},{"name": "EIE (ESE Site)","sequence": "GGGACC","CV": "NA","position": "581"},{"name": "EIE (ESE Site)","sequence": "GGACCA","CV": "NA","position": "582"},{"name": "PESE (ESE Site)","sequence": "GGACCATT","CV": "29.23778198997","position": "582"},{"name": "ESE_9G8 (ESE Site)","sequence": "GACCAT","CV": "64.37","position": "583"},{"name": "EIE (ESE Site)","sequence": "GACCAT","CV": "NA","position": "583"},{"name": "IIE (ESS Site)","sequence": "CATTTG","CV": "NA","position": "586"},{"name": "IIE (ESS Site)","sequence": "ATTTGT","CV": "NA","position": "587"},{"name": "IIE (ESS Site)","sequence": "TTTGTG","CV":

"NA","position": "588"},{"name": "IIE (ESS Site)","sequence": "TTGTGA","CV": "NA","position": "589"},{"name": "ESE_9G8 (ESE Site)","sequence": "TGTGAC","CV": "60.94","position": "590"},{"name": "EIE (ESE Site)","sequence": "TGTGAC","CV": "NA","position": "590"},{"name": "IIE (ESS Site)","sequence": "TGTGAC","CV": "NA","position": "590"},{"name": "EIE (ESE Site)","sequence": "GTGACC","CV": "NA","position": "591"},{"name": "EIE (ESE Site)","sequence": "TGACCC","CV": "NA","position": "592"},{"name": "Sironi_motif3 (ESS Site)","sequence": "TGACCCAA","CV": "75.26","position": "592"},{"name": "EIE (ESE Site)","sequence": "GACCCA","CV": "NA","position": "593"},{"name": "EIE (ESE Site)","sequence": "ACCCAA","CV": "NA","position": "594"},{"name": "RESCUE ESE (ESE Site)","sequence": "CAATCA","CV": "NA","position": "597"},{"name": "RESCUE ESE (ESE Site)","sequence": "AATCAG","CV": "NA","position": "598"},{"name": "EIE (ESE Site)","sequence": "AATCAG","CV": "NA","position": "598"},{"name": "EIE (ESE Site)","sequence": "TCAGTG","CV": "NA","position": "600"},{"name": "IIE (ESS Site)","sequence": "TCAGTG","CV": "NA","position": "600"},{"name": "ESE_SRp40 (ESE Site)","sequence": "TCAGTGG","CV": "88.11","position": "600"},{"name": "Sironi_motif2 (ESS Site)","sequence": "TCAGTGG","CV": "63.63","position": "600"},{"name": "Sironi_motif1 (ESS Site)","sequence": "TCAGTGGC","CV": "65.23","position": "600"},{"name": "ESS_hnRNPA1 (ESS Site)","sequence": "CAGTGG","CV": "67.03","position": "601"},{"name": "EIE (ESE Site)","sequence": "CAGTGG","CV": "NA","position": "601"},{"name": "EIE (ESE Site)","sequence": "AGTGGC","CV": "NA","position": "602"},{"name": "Sironi_motif2 (ESS Site)","sequence": "AGTGGCC","CV": "66.73","position": "602"},{"name": "EIE (ESE Site)","sequence": "GTGGCC","CV": "NA","position": "603"},{"name": "IIE (ESS Site)","sequence": "GTGGCC","CV": "NA","position": "603"},{"name": "ESE_SC35 (ESE Site)","sequence": "GGCCAGTG","CV": "91.1","position": "605"},{"name": "EIE (ESE Site)","sequence": "CCAGTG","CV": "NA","position": "607"},{"name": "ESE_SRp40 (ESE Site)","sequence": "CCAGTGC","CV": "80.86","position": "607"},{"name": "Sironi_motif1 (ESS Site)","sequence": "CCAGTGCC","CV": "65.92","position": "607"},{"name": "ESS_hnRNPA1 (ESS Site)","sequence": "CAGTGC","CV": "67.98","position": "608"},{"name": "EIE (ESE Site)","sequence": "CAGTGC","CV": "NA","position": "608"},{"name": "EIE (ESE Site)","sequence": "AGTGCC","CV": "NA","position": "609"},{"name": "EIE (ESE Site)","sequence": "GTGCCT","CV": "NA","position": "610"},{"name": "IIE (ESS Site)","sequence": "GTGCCT","CV": "NA","position": "610"},{"name": "EIE (ESE Site)","sequence": "TGCCTG","CV": "NA","position": "611"},{"name": "IIE (ESS Site)","sequence": "TGCCTG","CV": "NA","position": "611"},{"name": "ESE_SC35 (ESE Site)","sequence": "TGCCTGTG","CV": "76.72","position": "611"},{"name": "EIE (ESE Site)","sequence": "GCCTGT","CV": "NA","position": "612"},{"name": "IIE (ESS Site)","sequence": "GCCTGT","CV": "NA","position": "612"},{"name": "EIE (ESE Site)","sequence": "CCTGTG","CV": "NA","position": "613"},{"name": "IIE (ESS Site)","sequence": "CCTGTG","CV": "NA","position": "613"},{"name": "IIE (ESS Site)","sequence": "CTGTGT","CV": "NA","position": "614"},{"name": "ESE_SRp55 (ESE Site)","sequence": "TGTGTG","CV": "75.05","position": "615"},{"name": "EIE (ESE Site)","sequence": "TGTGTG","CV": "NA","position": "615"},{"name": "IIE (ESS Site)","sequence": "TGTGTG","CV": "NA","position": "615"},{"name": "Sironi_motif2 (ESS Site)","sequence": "TGTGTGT","CV": "71.52","position": "615"},{"name": "IIE (ESS Site)","sequence": "GTGTGT","CV": "NA","position": "616"},{"name": "ESE_SRp55 (ESE Site)","sequence": "TGTGTG","CV": "75.05","position": "617"},{"name": "EIE (ESE Site)","sequence": "TGTGTG","CV": "NA","position": "617"},{"name": "IIE (ESS Site)","sequence": "TGTGTG","CV": "NA","position": "617"},{"name": "Sironi_motif2 (ESS Site)","sequence": "TGTGTGC","CV": "73.56","position": "617"},{"name": "EIE (ESE Site)","sequence": "GTGTGC","CV": "NA","position": "618"},{"name": "IIE (ESS Site)","sequence": "GTGTGC","CV": "NA","position": "618"},{"name": "EIE (ESE Site)","sequence": "TGTGCC","CV": "NA","position": "619"},{"name": "IIE (ESS Site)","sequence": "TGTGCC","CV": "NA","position": "619"},{"name": "Sironi_motif3 (ESS Site)","sequence": "TGTGCCTA","CV": "63.99","position": "619"},{"name": "EIE (ESE Site)","sequence": "GTGCCT","CV": "NA","position": "620"},{"name": "IIE (ESS Site)","sequence": "GTGCCT","CV": "NA","position": "620"},{"name": "ESE_SRp55 (ESE Site)","sequence": "TGCCTA","CV": "79.22","position": "621"},{"name": "EIE (ESE Site)","sequence": "TGCCTA","CV": "NA","position": "621"},{"name": "EIE (ESE Site)","sequence": "GCCTAA","CV": "NA","position": "622"},{"name": "EIE (ESE Site)","sequence": "CCTAAT","CV": "NA","position":

"623"},{"name": "ESE_SRp40 (ESE Site)","sequence": "CTAATCG","CV": "83.14","position": "624"},{"name": "PESE (ESE Site)","sequence": "TCGTCAAG","CV": "34.9030896022","position": "628"},{"name": "ESE_SRp40 (ESE Site)","sequence": "CGTCAAG","CV": "83.14","position": "629"},{"name": "RESCUE ESE (ESE Site)","sequence": "GTCAAG","CV": "NA","position": "630"},{"name": "EIE (ESE Site)","sequence": "GTCAAG","CV": "NA","position": "630"},{"name": "EIE (ESE Site)","sequence": "TCAAGG","CV": "NA","position": "631"},{"name": "ESS_hnRNPA1 (ESS Site)","sequence": "CAAGGA","CV": "67.98","position": "632"},{"name": "EIE (ESE Site)","sequence": "CAAGGA","CV": "NA","position": "632"},{"name": "ESE_9G8 (ESE Site)","sequence": "AAGGAA","CV": "61.15","position": "633"},{"name": "ESS_hnRNPA1 (ESS Site)","sequence": "AAGGAA","CV": "76.79","position": "633"},{"name": "RESCUE ESE (ESE Site)","sequence": "AAGGAA","CV": "NA","position": "633"},{"name": "EIE (ESE Site)","sequence": "AAGGAA","CV": "NA","position": "633"},{"name": "PESE (ESE Site)","sequence": "AAGGAAGA","CV": "26.49326625162","position": "633"},{"name": "RESCUE ESE (ESE Site)","sequence": "AGGAAG","CV": "NA","position": "634"},{"name": "EIE (ESE Site)","sequence": "AGGAAG","CV": "NA","position": "634"},{"name": "PESE (ESE Site)","sequence": "AGGAAGAA","CV": "42.4655432001","position": "634"},{"name": "RESCUE ESE (ESE Site)","sequence": "GGAAGA","CV": "NA","position": "635"},{"name": "EIE (ESE Site)","sequence": "GGAAGA","CV": "NA","position": "635"},{"name": "PESE (ESE Site)","sequence": "GGAAGAAG","CV": "57.72483502628","position": "635"},{"name": "ESE_9G8 (ESE Site)","sequence": "GAAGAA","CV": "87.72","position": "636"},{"name": "RESCUE ESE (ESE Site)","sequence": "GAAGAA","CV": "NA","position": "636"},{"name": "EIE (ESE Site)","sequence": "GAAGAA","CV": "NA","position": "636"},{"name": "Sironi_motif1 (ESS Site)","sequence": "GAAGAAGG","CV": "64.41","position": "636"},{"name": "PESE (ESE Site)","sequence": "GAAGAAGG","CV": "42.58209283323","position": "636"},{"name": "ESE_Tra2 (ESE Site)","sequence": "AAGAA","CV": "100.34","position": "637"},{"name": "RESCUE ESE (ESE Site)","sequence": "AAGAAG","CV": "NA","position": "637"},{"name": "EIE (ESE Site)","sequence": "AAGAAG","CV": "NA","position": "637"},{"name": "RESCUE ESE (ESE Site)","sequence": "AGAAGG","CV": "NA","position": "638"},{"name": "EIE (ESE Site)","sequence": "AGAAGG","CV": "NA","position": "638"},{"name": "Sironi_motif2 (ESS Site)","sequence": "AGAAGGT","CV": "63.4","position": "638"},{"name": "PESE (ESE Site)","sequence": "AGAAGGTG","CV": "28.3568160797","position": "638"},{"name": "ESE_9G8 (ESE Site)","sequence": "GAAGGT","CV": "70.74","position": "639"},{"name": "ESS_hnRNPA1 (ESS Site)","sequence": "GAAGGT","CV": "70.84","position": "639"},{"name": "Sironi_motif2 (ESS Site)","sequence": "GAAGGTG","CV": "60.55","position": "639"},{"name": "ESS_hnRNPA1 (ESS Site)","sequence": "AAGGTG","CV": "70.6","position": "640"},{"name": "IIE (ESS Site)","sequence": "GGTGTA","CV": "NA","position": "642"},{"name": "IIE (ESS Site)","sequence": "GTGTAA","CV": "NA","position": "643"},{"name": "IIE (ESS Site)","sequence": "TGTAAT","CV": "NA","position": "644"},{"name": "RESCUE ESE (ESE Site)","sequence": "AATCAG","CV": "NA","position": "647"},{"name": "EIE (ESE Site)","sequence": "AATCAG","CV": "NA","position": "647"},{"name": "EIE (ESE Site)","sequence": "TCAGTG","CV": "NA","position": "649"},{"name": "IIE (ESS Site)","sequence": "TCAGTG","CV": "NA","position": "649"},{"name": "ESS_hnRNPA1 (ESS Site)","sequence": "CAGTGT","CV": "68.94","position": "650"},{"name": "IIE (ESS Site)","sequence": "CAGTGT","CV": "NA","position": "650"},{"name": "ESE_SRp55 (ESE Site)","sequence": "AGTGTC","CV": "74.35","position": "651"},{"name": "IIE (ESS Site)","sequence": "GTGTCA","CV": "NA","position": "652"},{"name": "ESE_SRp40 (ESE Site)","sequence": "TGTCAAC","CV": "80.2","position": "653"},{"name": "ESE_9G8 (ESE Site)","sequence": "GTCAAC","CV": "61.48","position": "654"},{"name": "EIE (ESE Site)","sequence": "GTCAAC","CV": "NA","position": "654"},{"name": "EIE (ESE Site)","sequence": "TCAACC","CV": "NA","position": "655"},{"name": "Sironi_motif3 (ESS Site)","sequence": "TCAACCAG","CV": "68.69","position": "655"},{"name": "PESE (ESE Site)","sequence": "TCAACCAG","CV": "29.02085865143","position": "655"},{"name": "EIE (ESE Site)","sequence": "CAACCA","CV": "NA","position": "656"},{"name": "RESCUE ESE (ESE Site)","sequence": "AACCAG","CV": "NA","position": "657"},{"name": "EIE (ESE Site)","sequence": "AACCAG","CV": "NA","position": "657"},{"name": "ESS_hnRNPA1 (ESS Site)","sequence": "CAGGTA","CV": "73.46","position": "660"},{"name": "Fas ESS (ESS Site)","sequence": "AGGTAA","CV": "NA","position": "661"},{"name":

"Fas ESS (ESS Site)","sequence": "GGTAAG","CV": "NA","position": "662"},{"name": "ESE_9G8 (ESE Site)","sequence": "TAAGAA","CV": "65.98","position": "664"},{"name": "ESS_hnRNPA1 (ESS Site)","sequence": "TAAGAA","CV": "74.89","position": "664"},{"name": "EIE (ESE Site)","sequence": "TAAGAA","CV": "NA","position": "664"},{"name": "ESE_Tra2 (ESE Site)","sequence": "AAGAA","CV": "100.34","position": "665"},{"name": "ESS_hnRNPA1 (ESS Site)","sequence": "AAGAAA","CV": "65.36","position": "665"},{"name": "RESCUE ESE (ESE Site)","sequence": "AAGAAA","CV": "NA","position": "665"},{"name": "EIE (ESE Site)","sequence": "AAGAAA","CV": "NA","position": "665"},{"name": "PESE (ESE Site)","sequence": "AAGAAAGA","CV": "27.3945557634","position": "665"},{"name": "RESCUE ESE (ESE Site)","sequence": "AGAAAG","CV": "NA","position": "666"},{"name": "EIE (ESE Site)","sequence": "AGAAAG","CV": "NA","position": "666"},{"name": "PESE (ESE Site)","sequence": "AGAAAGAA","CV": "38.43193045181","position": "666"},{"name": "RESCUE ESE (ESE Site)","sequence": "GAAAGA","CV": "NA","position": "667"},{"name": "EIE (ESE Site)","sequence": "GAAAGA","CV": "NA","position": "667"},{"name": "ESE_9G8 (ESE Site)","sequence": "AAAGAA","CV": "65.98","position": "668"},{"name": "RESCUE ESE (ESE Site)","sequence": "AAAGAA","CV": "NA","position": "668"},{"name": "EIE (ESE Site)","sequence": "AAAGAA","CV": "NA","position": "668"},{"name": "Sironi_motif1 (ESS Site)","sequence": "AAAGAAAT","CV": "62.71","position": "668"},{"name": "ESE_Tra2 (ESE Site)","sequence": "AAGAA","CV": "100.34","position": "669"},{"name": "ESS_hnRNPA1 (ESS Site)","sequence": "AAGAAA","CV": "65.36","position": "669"},{"name": "RESCUE ESE (ESE Site)","sequence": "AAGAAA","CV": "NA","position": "669"},{"name": "EIE (ESE Site)","sequence": "AAGAAA","CV": "NA","position": "669"},{"name": "EIE (ESE Site)","sequence": "AGAAAT","CV": "NA","position": "670"},{"name": "EIE (ESE Site)","sequence": "GAAATG","CV": "NA","position": "671"},{"name": "PESS (ESS Site)","sequence": "GAAATGTA","CV": "-31.0092534602","position": "671"},{"name": "PESS (ESS Site)","sequence": "AATGTATT","CV": "-34.86327193394","position": "673"},{"name": "IIE (ESS Site)","sequence": "ATGTAT","CV": "NA","position": "674"},{"name": "IIE (ESS Site)","sequence": "TGTATT","CV": "NA","position": "675"}]},"Splice site signals":{"matrix": [{"signal": "Acceptor splice site","matrix": [{"name": "MaxEnt Acceptor site","sequence": "TAAATATATTTTATCTTTAGGGC","CV": "8.95","position": "1","WT": "true"}]},{"signal": "Acceptor splice site","matrix": [{"name": "HSF Acceptor site (matrix AG)","sequence": "TTTTATCTTTAGGG","CV": "89.93","position": "9","WT": "true"}]},{"signal": "Acceptor splice site","matrix": [{"name": "HSF Acceptor site (matrix AG)","sequence": "TTTAGGGCTTAGGT","CV": "79.16","position": "16"}]},{"signal": "Donor splice site","matrix": [{"name": "HSF Donor site (matrix GT)","sequence": "TAGGTGTGA","CV": "85.78","position": "25"}]},{"signal": "Donor splice site","matrix": [{"name": "HSF Donor site (matrix GT)","sequence": "GGTGTGATC","CV": "66.8","position": "27"}]},{"signal": "Acceptor splice site","matrix": [{"name": "MaxEnt Acceptor site","sequence": "GGATTTAAATTTCTCCGAAGCTT","CV": "4.08","position": "47"}]},{"signal": "Acceptor splice site","matrix": [{"name": "HSF Acceptor site (matrix AG)","sequence": "ATTTCTCCGAAGCT","CV": "80.16","position": "55"}]},{"signal": "Donor splice site","matrix": [{"name": "HSF Donor site (matrix GT)","sequence": "GATGTTGGA","CV": "72.31","position": "74"}]},{"signal": "Acceptor splice site","matrix": [{"name": "HSF Acceptor site (matrix AG)","sequence": "GTTGGATGTGAGCC","CV": "69.88","position": "77"}]},{"signal": "Donor splice site","matrix": [{"name": "HSF Donor site (matrix GT)","sequence": "GATGTGAGC","CV": "82.6","position": "81"}]},{"signal": "Donor splice site","matrix": [{"name": "MaxEnt Donor site","sequence": "GATGTGAGC","CV": "4.72","position": "81"}]},{"signal": "Acceptor splice site","matrix": [{"name": "HSF Acceptor site (matrix AG)","sequence": "GAGCCCTGCCAGTG","CV": "73.81","position": "86"}]},{"signal": "Donor splice site","matrix": [{"name": "HSF Donor site (matrix GT)","sequence": "AGTGTAACC","CV": "70.31","position": "96"}]},{"signal": "Acceptor splice site","matrix": [{"name": "HSF Acceptor site (matrix AG)","sequence": "TCCATGGCTCAGTG","CV": "76.92","position": "105"}]},{"signal": "Donor splice site","matrix": [{"name": "HSF Donor site (matrix GT)","sequence": "TCAGTGAAC","CV": "66.9","position": "113"}]},{"signal": "Acceptor splice site","matrix": [{"name": "MaxEnt Acceptor site","sequence": "GCAATCCTCACTCTGGGCAGTGT","CV": "4.72","position": "129"}]},{"signal": "Acceptor splice site","matrix": [{"name": "HSF Acceptor site (matrix AG)","sequence": "CACTCTGGGCAGTG","CV":

"75.6","position": "137"}]},{"signal": "Donor splice site","matrix": [{"name": "HSF Donor site (matrix GT)","sequence": "GCAGTGTGA","CV": "69.92","position": "145"}]},{"signal": "Donor splice site","matrix": [{"name": "HSF Donor site (matrix GT)","sequence": "AGTGTGAGT","CV": "80.4","position": "147"}]},{"signal": "Donor splice site","matrix": [{"name": "MaxEnt Donor site","sequence": "AGTGTGAGT","CV": "6.96","position": "147"}]},{"signal": "Acceptor splice site","matrix": [{"name": "HSF Acceptor site (matrix AG)","sequence": "AGTGCAAAAAAGAA","CV": "65.24","position": "153"}]},{"signal": "Acceptor splice site","matrix": [{"name": "HSF Acceptor site (matrix AG)","sequence": "AAGAAGCCAAAGGA","CV": "65.27","position": "162"}]},{"signal": "Acceptor splice site","matrix": [{"name": "HSF Acceptor site (matrix AG)","sequence": "AAAGGACTTCAGTG","CV": "71.57","position": "170"}]},{"signal": "Acceptor splice site","matrix": [{"name": "MaxEnt Acceptor site","sequence": "CTTCAGTGTGACACCTGCAGAGA","CV": "4.17","position": "176"}]},{"signal": "Donor splice site","matrix": [{"name": "HSF Donor site (matrix GT)","sequence": "TCAGTGTGA","CV": "69.53","position": "178"}]},{"signal": "Donor splice site","matrix": [{"name": "HSF Donor site (matrix GT)","sequence": "AGTGTGACA","CV": "67.72","position": "180"}]},{"signal": "Acceptor splice site","matrix": [{"name": "HSF Acceptor site (matrix AG)","sequence": "TGACACCTGCAGAG","CV": "80.18","position": "184"}]},{"signal": "Acceptor splice site","matrix": [{"name": "HSF Acceptor site (matrix AG)","sequence": "ACACCTGCAGAGAA","CV": "68.42","position": "186"}]},{"signal": "Acceptor splice site","matrix": [{"name": "HSF Acceptor site (matrix AG)","sequence": "TTTATGGGTTAGAT","CV": "78.05","position": "204"}]},{"signal": "Donor splice site","matrix": [{"name": "HSF Donor site (matrix GT)","sequence": "TGGGTTAGA","CV": "83.53","position": "208"}]},{"signal": "Donor splice site","matrix": [{"name": "HSF Donor site (matrix GT)","sequence": "GATGTCACC","CV": "69.21","position": "215"}]},{"signal": "Acceptor splice site","matrix": [{"name": "HSF Acceptor site (matrix AG)","sequence": "ACCAATTGTAAGGC","CV": "70.94","position": "221"}]},{"signal": "Donor splice site","matrix": [{"name": "HSF Donor site (matrix GT)","sequence": "ATTGTAAGG","CV": "81","position": "225"}]},{"signal": "Donor splice site","matrix": [{"name": "MaxEnt Donor site","sequence": "ATTGTAAGG","CV": "4.89","position": "225"}]},{"signal": "Acceptor splice site","matrix": [{"name": "MaxEnt Acceptor site","sequence": "GGCCTGTGACTGTGACACAGCTG","CV": "3.08","position": "232"}]},{"signal": "Donor splice site","matrix": [{"name": "HSF Donor site (matrix GT)","sequence": "CCTGTGACT","CV": "70.46","position": "234"}]},{"signal": "Donor splice site","matrix": [{"name": "HSF Donor site (matrix GT)","sequence": "ACTGTGACA","CV": "67.65","position": "240"}]},{"signal": "Acceptor splice site","matrix": [{"name": "HSF Acceptor site (matrix AG)","sequence": "ACTGTGACACAGCT","CV": "76.73","position": "240"}]},{"signal": "Donor splice site","matrix": [{"name": "HSF Donor site (matrix GT)","sequence": "ACTGTCTGT","CV": "69.76","position": "269"}]},{"signal": "Donor splice site","matrix": [{"name": "HSF Donor site (matrix GT)","sequence": "TCTGTAATG","CV": "69.51","position": "273"}]},{"signal": "Acceptor splice site","matrix": [{"name": "HSF Acceptor site (matrix AG)","sequence": "TGTAATGCTAAGAC","CV": "71.99","position": "275"}]},{"signal": "Acceptor splice site","matrix": [{"name": "HSF Acceptor site (matrix AG)","sequence": "ATGCTAAGACAGGG","CV": "76.97","position": "279"}]},{"signal": "Acceptor splice site","matrix": [{"name": "HSF Acceptor site (matrix AG)","sequence": "AAGACAGGGCAGTG","CV": "65.62","position": "284"}]},{"signal": "Donor splice site","matrix": [{"name": "HSF Donor site (matrix GT)","sequence": "CAGGGCAGT","CV": "66.64","position": "288"}]},{"signal": "Acceptor splice site","matrix": [{"name": "HSF Acceptor site (matrix AG)","sequence": "TGCATCTGCAAGCC","CV": "72.39","position": "296"}]},{"signal": "Acceptor splice site","matrix": [{"name": "HSF Acceptor site (matrix AG)","sequence": "CCAATGTTGAAGGG","CV": "75.27","position": "309"}]},{"signal": "Acceptor splice site","matrix": [{"name": "HSF Acceptor site (matrix AG)","sequence": "TGTTGAAGGGAGAC","CV": "69.45","position": "313"}]},{"signal": "Acceptor splice site","matrix": [{"name": "HSF Acceptor site (matrix AG)","sequence": "AAATGTTTGGAGGG","CV": "74.72","position": "335"}]},{"signal": "Donor splice site","matrix": [{"name": "HSF Donor site (matrix GT)","sequence": "AATGTTTGG","CV": "73.37","position": "336"}]},{"signal": "Donor splice site","matrix": [{"name": "HSF Donor site (matrix GT)","sequence": "TCTGTCTGC","CV": "65.47","position": "381"}]},{"signal": "Acceptor splice site","matrix": [{"name": "HSF Acceptor site

(matrix AG)","sequence": "AACTGTGATAAGAC","CV": "66.93","position": "395"}]},{"signal": "Donor splice site","matrix": [{"name": "HSF Donor site (matrix GT)","sequence": "ACTGTGATA","CV": "67.96","position": "396"}]},{"signal": "Acceptor splice site","matrix": [{"name": "MaxEnt Acceptor site","sequence": "GCTGTGTAACAAATCAACAGGAC","CV": "3.97","position": "430"}]},{"signal": "Donor splice site","matrix": [{"name": "HSF Donor site (matrix GT)","sequence": "TGTGTAACA","CV": "69.19","position": "432"}]},{"signal": "Acceptor splice site","matrix": [{"name": "HSF Acceptor site (matrix AG)","sequence": "ACAAATCAACAGGA","CV": "74.95","position": "438"}]},{"signal": "Acceptor splice site","matrix": [{"name": "MaxEnt Acceptor site","sequence": "ACAATGTCCTTGCAAATTAGGGG","CV": "5.26","position": "451"}]},{"signal": "Donor splice site","matrix": [{"name": "HSF Donor site (matrix GT)","sequence": "AATGTCCTT","CV": "65.7","position": "453"}]},{"signal": "Acceptor splice site","matrix": [{"name": "HSF Acceptor site (matrix AG)","sequence": "CTTGCAAATTAGGG","CV": "76.07","position": "459"}]},{"signal": "Acceptor splice site","matrix": [{"name": "HSF Acceptor site (matrix AG)","sequence": "TAGGGGTAACAGGT","CV": "76.72","position": "468"}]},{"signal": "Donor splice site","matrix": [{"name": "HSF Donor site (matrix GT)","sequence": "GGGGTAACA","CV": "80.1","position": "470"}]},{"signal": "Donor splice site","matrix": [{"name": "MaxEnt Donor site","sequence": "GGGGTAACA","CV": "3.2","position": "470"}]},{"signal": "Donor splice site","matrix": [{"name": "HSF Donor site (matrix GT)","sequence": "CAGGTCTTC","CV": "73.95","position": "477"}]},{"signal": "Acceptor splice site","matrix": [{"name": "MaxEnt Acceptor site","sequence": "CAGGTCTTCGCTGTAATCAGTGT","CV": "4.08","position": "477"}]},{"signal": "Acceptor splice site","matrix": [{"name": "HSF Acceptor site (matrix AG)","sequence": "CGCTGTAATCAGTG","CV": "74.79","position": "485"}]},{"signal": "Donor splice site","matrix": [{"name": "HSF Donor site (matrix GT)","sequence": "GCTGTAATC","CV": "69.57","position": "486"}]},{"signal": "Acceptor splice site","matrix": [{"name": "HSF Acceptor site (matrix AG)","sequence": "AATCAGTGTGAGCC","CV": "66.51","position": "491"}]},{"signal": "Donor splice site","matrix": [{"name": "HSF Donor site (matrix GT)","sequence": "TCAGTGTGA","CV": "69.53","position": "493"}]},{"signal": "Donor splice site","matrix": [{"name": "HSF Donor site (matrix GT)","sequence": "AGTGTGAGC","CV": "77.48","position": "495"}]},{"signal": "Donor splice site","matrix": [{"name": "MaxEnt Donor site","sequence": "AGTGTGAGC","CV": "3.73","position": "495"}]},{"signal": "Acceptor splice site","matrix": [{"name": "HSF Acceptor site (matrix AG)","sequence": "TGAGCCTCACAGGT","CV": "82.64","position": "499"}]},{"signal": "Donor splice site","matrix": [{"name": "HSF Donor site (matrix GT)","sequence": "CAGGTACAA","CV": "80.04","position": "508"}]},{"signal": "Donor splice site","matrix": [{"name": "MaxEnt Donor site","sequence": "CAGGTACAA","CV": "7.09","position": "508"}]},{"signal": "Acceptor splice site","matrix": [{"name": "MaxEnt Acceptor site","sequence": "ACAATTTTCAACACTGCCAGATG","CV": "4.95","position": "528"}]},{"signal": "Acceptor splice site","matrix": [{"name": "HSF Acceptor site (matrix AG)","sequence": "CAACACTGCCAGAT","CV": "75.92","position": "536"}]},{"signal": "Acceptor splice site","matrix": [{"name": "HSF Acceptor site (matrix AG)","sequence": "CAGATGTGTGAGTG","CV": "65.37","position": "545"}]},{"signal": "Donor splice site","matrix": [{"name": "HSF Donor site (matrix GT)","sequence": "GATGTGTGA","CV": "75.66","position": "547"}]},{"signal": "Donor splice site","matrix": [{"name": "HSF Donor site (matrix GT)","sequence": "TGTGTGAGT","CV": "79.04","position": "549"}]},{"signal": "Donor splice site","matrix": [{"name": "MaxEnt Donor site","sequence": "TGTGTGAGT","CV": "5.84","position": "549"}]},{"signal": "Donor splice site","matrix": [{"name": "HSF Donor site (matrix GT)","sequence": "TGAGTGTGA","CV": "69.6","position": "553"}]},{"signal": "Donor splice site","matrix": [{"name": "HSF Donor site (matrix GT)","sequence": "AGTGTGATT","CV": "70.7","position": "555"}]},{"signal": "Acceptor splice site","matrix": [{"name": "MaxEnt Acceptor site","sequence": "ACCATTTGTGACCCAATCAGTGG","CV": "3.56","position": "584"}]},{"signal": "Donor splice site","matrix": [{"name": "HSF Donor site (matrix GT)","sequence": "TTTGTGACC","CV": "66.44","position": "588"}]},{"signal": "Acceptor splice site","matrix": [{"name": "HSF Acceptor site (matrix AG)","sequence": "TGACCCAATCAGTG","CV": "74.13","position": "592"}]},{"signal": "Acceptor splice site","matrix": [{"name": "HSF Acceptor site (matrix AG)","sequence": "ATCAGTGGCCAGTG","CV": "73.45","position": "599"}]},{"signal": "Donor splice site","matrix": [{"name": "HSF Donor site (matrix GT)","sequence": "CCTGTGTGT","CV": "73.28","position":

"613"}]},{"signal": "Donor splice site","matrix": [{"name": "HSF Donor site (matrix GT)","sequence": "TGTGTGTGC","CV": "68.91","position": "615"}]},{"signal": "Acceptor splice site","matrix": [{"name": "MaxEnt Acceptor site","sequence": "GTGTGTGCCTAATCGTCAAGGAA","CV": "3.07","position": "616"}]},{"signal": "Acceptor splice site","matrix": [{"name": "HSF Acceptor site (matrix AG)","sequence": "CTAATCGTCAAGGA","CV": "76.84","position": "624"}]},{"signal": "Acceptor splice site","matrix": [{"name": "HSF Acceptor site (matrix AG)","sequence": "TCGTCAAGGAAGAA","CV": "69.93","position": "628"}]},{"signal": "Acceptor splice site","matrix": [{"name": "HSF Acceptor site (matrix AG)","sequence": "TCAAGGAAGAAGGT","CV": "67.06","position": "631"}]},{"signal": "Donor splice site","matrix": [{"name": "HSF Donor site (matrix GT)","sequence": "AAGGTGTAA","CV": "77.58","position": "640"}]},{"signal": "Acceptor splice site","matrix": [{"name": "HSF Acceptor site (matrix AG)","sequence": "AGGTGTAATCAGTG","CV": "71.35","position": "641"}]},{"signal": "Donor splice site","matrix": [{"name": "HSF Donor site (matrix GT)","sequence": "GGTGTAATC","CV": "69.64","position": "642"}]},{"signal": "Acceptor splice site","matrix": [{"name": "HSF Acceptor site (matrix AG)","sequence": "AGTGTCAACCAGGT","CV": "78.22","position": "651"}]},{"signal": "Donor splice site","matrix": [{"name": "HSF Donor site (matrix GT)","sequence": "CAGGTAAGA","CV": "97.33","position": "660","WT": "true"}]},{"signal": "Donor splice site","matrix": [{"name": "MaxEnt Donor site","sequence": "CAGGTAAGA","CV": "10.77","position": "660","WT": "true"}]},{"signal": "Donor splice site","matrix": [{"name": "HSF Donor site (matrix GT)","sequence": "AATGTATTA","CV": "69.78","position": "673"}]}]},"Branch point signals":{"matrix": [{"name": "BP","sequence": "GGCTTAG","CV": "79","position": "21"},{"name": "BP","sequence": "CTCCGAA","CV": "75.81","position": "59"},{"name": "BP","sequence": "TCCGAAG","CV": "68.6","position": "60"},{"name": "BP","sequence": "CCCTGCC","CV": "65.2","position": "89"},{"name": "BP","sequence": "GGCTCAG","CV": "90.83","position": "110"},{"name": "BP","sequence": "TCCTCAC","CV": "98.24","position": "133"},{"name": "BP","sequence": "GCCAAAG","CV": "68.49","position": "167"},{"name": "BP","sequence": "ACTTCAG","CV": "68.95","position": "175"},{"name": "BP","sequence": "TGCAGAG","CV": "70.91","position": "191"},{"name": "BP","sequence": "ATGTCAC","CV": "66.42","position": "216"},{"name": "BP","sequence": "CACCAAT","CV": "68.14","position": "220"},{"name": "BP","sequence": "CTGTGAC","CV": "65.74","position": "235"},{"name": "BP","sequence": "CTGTGAC","CV": "65.74","position": "241"},{"name": "BP","sequence": "GACACAG","CV": "73.45","position": "245"},{"name": "BP","sequence": "CCCTCCC","CV": "70.14","position": "258"},{"name": "BP","sequence": "TGCTAAG","CV": "79.11","position": "280"},{"name": "BP","sequence": "AGCCCAA","CV": "75.28","position": "306"},{"name": "BP","sequence": "GCCCAAT","CV": "73.38","position": "307"},{"name": "BP","sequence": "TACCTAC","CV": "70.51","position": "356"},{"name": "BP","sequence": "TCCTCTG","CV": "65.88","position": "378"},{"name": "BP","sequence": "GCCTCAC","CV": "98.13","position": "502"},{"name": "BP","sequence": "CTCACAG","CV": "78.58","position": "504"},{"name": "BP","sequence": "GCCAGAT","CV": "74.22","position": "543"},{"name": "BP","sequence": "GACCCAA","CV": "75.61","position": "593"},{"name": "BP","sequence": "ACCCAAT","CV": "70.76","position": "594"},{"name": "BP","sequence": "TGCCTAA","CV": "66.18","position": "621"},{"name": "BP","sequence": "GCCTAAT","CV": "82.41","position": "622"},{"name": "BP","sequence": "TCGTCAA","CV": "65.89","position": "628"}]}}
